# High-resolution computational modeling of immune responses in the gut

**DOI:** 10.1093/gigascience/giz062

**Published:** 2019-06-11

**Authors:** Meghna Verma, Josep Bassaganya-Riera, Andrew Leber, Nuria Tubau-Juni, Stefan Hoops, Vida Abedi, Xi Chen, Raquel Hontecillas

**Affiliations:** 1 Nutritional Immunology and Molecular Medicine Laboratory, Biocomplexity Institute of Virginia Tech, 1015 Life Science Circle, Blacksburg, VA 24061, USA; 2 Graduate Program in Translational Biology, Medicine and Health, Virginia Tech, Blacksburg, 1 Riverside Circle, Roanoke, VA 24016, USA; 3 Grado Department of Industrial and Systems Engineering, Virginia Tech, 250 Perry St, Blacksburg, VA 24061, USA

**Keywords:** agent-based model, ordinary differential equation, Gaussian process, Helicobacter pylori, high-performance computing, metamodel, sensitivity analysis, immune system, dendritic cells, macrophages

## Abstract

**Background:**

*Helicobacter pylori* causes gastric cancer in 1–2% of cases but is also beneficial for protection against allergies and gastroesophageal diseases. An estimated 85% of *H. pylori*–colonized individuals experience no detrimental effects. To study the mechanisms promoting host tolerance to the bacterium in the gastrointestinal mucosa and systemic regulatory effects, we investigated the dynamics of immunoregulatory mechanisms triggered by *H. pylori* using a high-performance computing–driven ENteric Immunity SImulator multiscale model. Immune responses were simulated by integrating an agent-based model, ordinary, and partial differential equations.

**Results:**

The outputs were analyzed using 2 sequential stages: the first used a partial rank correlation coefficient regression–based and the second a metamodel-based global sensitivity analysis. The influential parameters screened from the first stage were selected to be varied for the second stage. The outputs from both stages were combined as a training dataset to build a spatiotemporal metamodel. The Sobol indices measured time-varying impact of input parameters during initiation, peak, and chronic phases of infection. The study identified epithelial cell proliferation and epithelial cell death as key parameters that control infection outcomes. *In silico* validation showed that colonization with *H. pylori* decreased with a decrease in epithelial cell proliferation, which was linked to regulatory macrophages and tolerogenic dendritic cells.

**Conclusions:**

The hybrid model of *H. pylori* infection identified epithelial cell proliferation as a key factor for successful colonization of the gastric niche and highlighted the role of tolerogenic dendritic cells and regulatory macrophages in modulating the host responses and shaping infection outcomes.

## Background

Computational modeling of immune response dynamics can provide novel insights and facilitate systems-level understanding of the interactions at the gastric mucosa during infection. Ordinary differential equation (ODE)-based methods are deterministic and based on the average response of cells over time. Dynamical models are used in immunology for system-level analyses of CD4^+^ T cell differentiation [1], macrophage differentiation [2], immune responses elicited by *Clostridium difficile* infection [3], co-infections [4], and in cancer and immunotherapy [5]. However, ODE-based models lack the spatial aspects and the features to study the organ and immune cell topology over time. Agent-based models (ABM) employ a bottom-up approach that focuses on the spatial and temporal aspects of individual immune cells, unlike the ODE-based methods. This rule-based method includes agents that act as local entities which - interact locally with other agents, move in space, follow a set of rules representing their role in a given system and contribute towards generating an emergent behavior. Because the immune system is a complex dynamical system [6] whose components, *i.e*., the immune cells, move in space and time changing their location, ABMs are useful tools that can be used to understand biological mechanisms and reveal hidden insights.

*Helicobacter pylori* is a gram-negative bacterium that has persistently colonized the human stomach since early evolution [7, 8] and is currently found in >50% [9] of the global population. *H. pylori* has co-evolved with humans for thousands of years, such that an estimated 85% of *H. pylori*−colonized individuals do not present any detrimental effects. Thus, the vast majority of carriers (i.e., up to 75%) remain asymptomatic, while only 15% develop ulcers and <3% develop cancer. Further, growing and sometimes contradictory evidence from recent experimental, clinical studies and epidemiological studies suggest that *H. pylori* might provide protection against obesity-related inflammation, type 2 diabetes [10], esophageal and cardiac pathologies, childhood asthma and allergies [11], and autoimmune diseases. In this context, it is crucial to understand the mechanisms that promote host tolerance to the bacterium in the gastrointestinal mucosa and its systemic regulatory effects because these have been linked to the beneficial commensal aspects of *H. pylori*−human host interaction. Computational models provide a cost-effective and predictive way to study the complex and dynamic immune system interactions and form a non-intuitive novel hypothesis. Solving the complex puzzle of immunoregulatory mechanisms that include large spatiotemporal scales ranging from cellular, intracellular, and tissue- to organ-level scales is a major unsolved challenge that requires the application of computational modeling and data analytics.

An advanced hybrid model used to study the mucosal immune response during gut inflammation highlighted the mechanisms by which effector CD4^+^ T-cell responses contributed to tissue damage in the gut mucosa following immune dysregulation [12]. Other hybrid models with the integration of ABM, ODE, and partial differential equation (PDE) technologies were developed to elucidate the dynamics of tumor development [13] and tumor growth models [14]. These combined techniques have been used to develop multi-organ models in various situations, including the study of granuloma formation [15] and pressure-driven ulcer formation in patients with spinal cord injury [16]. The different agent-based simulators with immunology-related applications are discussed and summarized by Cappuccio et al. [17] and Bassaganya-Riera et al. [18]. The different multiscale modeling tools and agent-based immune simulators are compared by Mei et al. [12] and An et al. [19].

In this study, we utilize a high-resolution ENteric Immunity SImulator (ENISI)-based model of the stomach for simulating the mucosal immune responses to *H. pylori* infection. The advanced hybrid multiscale modeling platform ENISI multiscale model (MSM) is capable of scaling up to 10^12^ agents [20]. The host immune responses initiated during *H. pylori* infection and the underlying immunoregulatory mechanisms are captured using the ENISI multiscale hybrid model. The underlying intracellular mechanisms that control cytokine production, signaling, and differentiation of macrophages and T cells are modeled by using ODEs; the diffusion of cytokine values is modeled using PDEs; and the location and interactions among the immune cells, bacteria, and epithelial cells are modeled using ABMs. The hybrid model thereby represents a high-performance computing (HPC)-driven large-scale simulation of the massively interacting cells and molecules in the immune system, integrating the multiple modeling technologies from molecules to systems across multiple spatiotemporal scales.

To understand the dynamics and emergent immunological patterns described by this hybrid model, we employed sensitivity analysis (SA), an important part of the model analysis used to explore the influence of varying model parameters on the simulation outputs. The influence of the effects of changes in parameter values on the model output explains the model dynamics that underlay the outputs [21, 22]. Furthermore, SA examines the robustness of the model output at a different range of parameter values that correspond to a range of different assumptions. We employed global SA and conducted a 2-stage spatiotemporal global SA approach. First, we used a regression-based method such as the partial rank correlation coefficient (PRCC) and screened the important input parameters that were shown to have the most influence on the output cell populations obtained from the hybrid model. Second, the screened input parameters from the first stage were varied to build a second-stage parameter design matrix, and the computer simulations were again run using the hybrid ENISI model. The outputs from both analytic stages were combined and used as a 'training dataset' to build a spatiotemporal Gaussian process (GP)-based metamodel. Finally, variance-based decomposition global SA was used to compute the Sobol’ indices and the most influential parameters over the course of infection were identified. The data analytics methods conducted on the hybrid model identified the epithelial cell parameters such as epithelial cell proliferation as the most influential ones, required for the successful colonization of *H. pylori* in the gastric microenvironment.

## Methods

### Hybrid multiscale *H. pylori* infection model

We developed a multicompartment, high-resolution, hybrid ABM/ODE/PDE model to capture the dynamics of the immune response during *H. pylori* colonization of the gastric mucosa. The model has a spatial discretization such that the dimension of the entire (2D) grid is 30 mm x 10 mm. An individual lattice site for our simulation is 1 mm x 1 mm; however, this is a configurable run parameter and can be changed without modifying the model. An individual lattice site is a unit wherein all the agents located within that location have the same cytokine environment, i.e., for all the agents in that location, ENISI-MSM would send the same concentration of cytokines to COPASI. The entire grid is divided within into 4 functionally and anatomically distinct sized compartments: lumen, epithelium, lamina propria (LP), and gastric lymph node (GLN). In the model, there are multiple cells and cell types (i.e., agents) within this dimensional grid. At the beginning of each simulation cycle, the cells (agents) are randomly placed within the 2D grid. The separation of different types of agents, corresponding to different cell types, into compartments within the grid is based on the conceptual framework that underlines the model, which is based on the authors' expertise and available information. Currently the individual agents do not have any physical size meaning such that there is no limit of agents within each individual spatial grid. The model is initialized with the concentration of different cell types (i.e., agents for, e.g., macrophages) at the beginning of the simulation by the user.

The use of a border implementation permits the migration of agents (cells) across compartments and facilitates the unidirectional and bidirectional movement of the agents. At the cellular scale, ENISI-MSM, simulated epithelial cells, macrophages, dendritic cells (DCs), CD4^+^ T cells, and bacteria which are implemented as agents in the model. At the intracellular scale, calibrated ODE-based models of T cells [23] and macrophages [2] were used to represent the intracellular pathways controlling cytokine production. The CD4^+^ T-cell ODE model was calibrated using the experimental data provided in Table S1 of Carbo et al. [23]. The Particle Swarm algorithm implemented in COPASI was used to determine unknown model parameter values and fully calibrate the CD4^+^ T-cell ODE model (details are described in Carbo et al. [23]). The intracellular macrophage ODE model was calibrated using a combination of sourced and new data generated from *in vitro* macrophage differentiation studies, which were compiled into a dataset provided in the S2 file of Leber et al. [2]. The parameter values are specified within the previously published articles—CD4^+^ T-cell ODE model [23] and macrophages [2]. The parameters of the calibrated ODEs were kept unchanged, and the ABM parameters were calibrated by approximating the output simulations such that they qualitatively resembled the patterns observed in a mouse model of *H. pylori* infection [24], also described in detail in Results (see Results section, *Hybrid model simulations produce similar immune dynamics observed in previously published experimental data*). Cytokines secreted by immune cells and their change in concentration were modeled by PDE. The degradation value of the cytokines and the diffusion constant determines the spread of the cytokine value of one lattice site to its neighboring lattice site similar to as described in our previous work [12]. The features of ABM, ODE, and PDE were combined to create a multiscale modeling environment that spanned across different orders of spatiotemporal scales. The model output contains information about the x and y coordinates of the agents at every time point. The cytokines and internal signaling pathways that drive functional fates of cells are well mixed within a cell; i.e., we have only temporal resolution within the cell during a time step. However, because the model is capable of providing information regarding spatial coordinates over time, we claim the model to be a spatiotemporal model.

**Figure 1:**
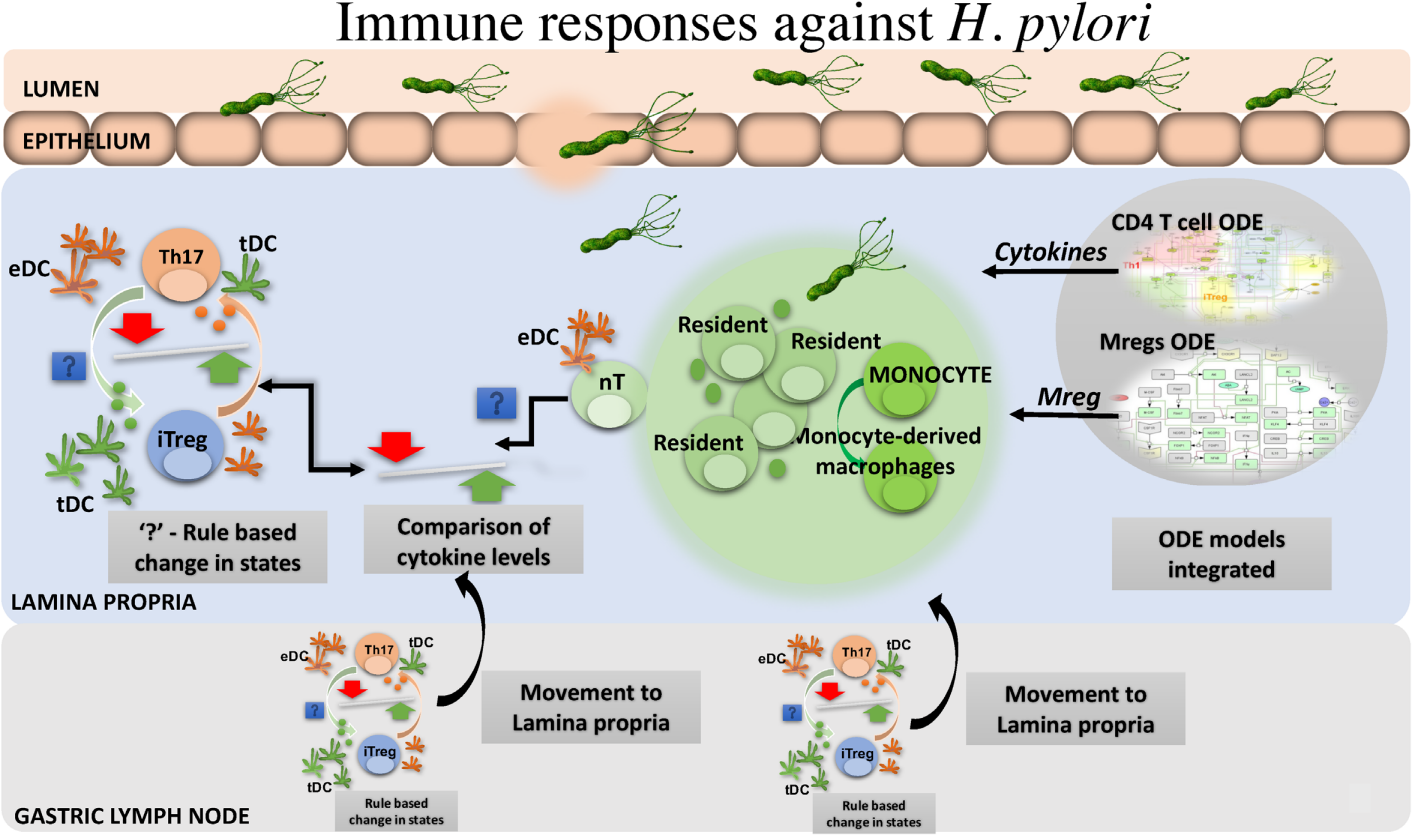
*Helicobacter pylori* infection schematic diagram of the hybrid ABM ODE model. The model comprises of 4 compartments, (i) the lumen that contains *H. pylori* and bacteria; (ii) epithelium that contains epithelial cells and dendritic cells; (iii) lamina propria (LP) that contains a variety of immune cells including the infiltrating effector (eDCs) and tolerogenic dendritic cells (tDCs), monocytes, regulatory macrophages (Mreg; both resident and monocyte-derived macrophages), T helper (Th) cells, and naive CD4^+^ T cells (nT), Th1, iTreg, Th17, regulatory T (Treg) cells; and (iv) gastric lymph node compartment that contains eDCs, tDCs, Th1, Th17, iTreg, and nT. The Treg cells in the LP are the type 1 regulatory (Tr1) T cells with regulatory function whose expansion is largely dependent on environmental IL-10. These are different from iTreg, which are T cells differentiated from naive T cells in the presence of tDCs and TGF-β cytokines. The 2 calibrated ODEs for T cells and regulatory macrophages are integrated as the ODE components in the hybrid model. The cellular agents are simulated in a 2D grid space with their behavior defined by a set of rules during a course of *H. pylori* infection..

The code for the hybrid model is freely accessible and can be downloaded from the GitHub repository [ 25]. The detailed use instructions on “how to run a simulation,” and codes for creating specific examples presented here are described in Additional file S1. The SciCrunch database assigned RRID for ENISI-MSM is RRID:SCR_016918. The design implementation of the code structure is depicted in the Additional file Fig. S1. The hybrid model is implemented in C++ and utilizes the Repast HPC library [ 26, 27]. For the ODEs, we utilized COPASI [28], an ODE-based modeling tool used in computational biology. The rules in the model that described the interaction of *H. pylori* with the gastric mucosa and immune responses resulting from the infection are derived from the findings in our previously published studies [ 1, 2]. Specifically, this hybrid model reproduced the immune responses generated by the interaction of *H. pylori* and the resident macrophages as shown in the mouse model of *H. pylori* infection [24]. The rules for each cell type in the *H. pylori* infection are summarized in Table 1. A pictorial representation of the rules is depicted in Figure.   1. These cell types, represented as agents, act according to the rules (as in Table 1), which are updated at each discrete simulation cycle.

**Table 1:** A list of rules for all the agent types implemented in the hybrid model

Name of agent	Agent type	Rules
*Helicobacter pylori*	*H. pylori*	Moves across the epithelial cell border if near damaged epithelial layer; proliferates in the lumen and LP; dies in LP and in the lumen due to the damage of epithelial cells by Th1 or Th17 cells
Macrophages	Monocyte	Proliferates in presence of effector DCs or damaged epithelial cells; proliferates in the LP; differentiates to regulatory macrophage based on the output from the macrophage ODE; differentiates to inflammatory macrophages in presence of IFN-γ; dies naturally
	Resident	Proliferates in the presence of *H. pylori*; secretes IL-10; dies naturally; dies due to Th1 and Treg cells
	Regulatory	Proliferates and removes bacteria; dies; secretes IL-10
	Inflammatory	Proliferates in the presence of damaged epithelial cells; dies naturally
Dendritics	Immature	Moves from LP to epithelium compartment and from the epithelium to the LP; differentiates to tolerogenic DC in the presence of tolerogenic bacteria, both in epithelium and LP; differentiates to effector DC in the presence of *H. pylori*; proliferates in LP and GLN; dies naturally
	Effector	Moves from LP to GLN; moves from epithelium to LP; secretes IL-6 and IL-12; dies naturally
	Tolerogenic	Moves from LP to GLN; moves from epithelium to LP; secretes TGF-β; dies naturally
T cells	Naive	In the presence of effector DCs: differentiates to Th1 in the presence of IFN-γ or IL-12; differentiates to Th17 in the presences of IL-6 or TGF-β; in the presence of tolerogenic DCs: differentiates to iTreg in the presence of TGF-β; differentiates to Treg in the presences of IL-10; dies naturally
	Th1	Secretes IFN-γ; moves from GLN to LP; proliferates in LP and GLN; dies naturally
	Th17	Secretes IL-17; in the presence of tolerogenic DC, transitions to iTreg cells; moves from GLN to LP; proliferates in LP and GLN;dies naturally
	iTreg	Secretes IL-10; in the presence of tolerogenic DC, transitions to iTreg cells;moves from GLN to LP; proliferates in LP and GLN; dies naturally
	Tr	Secretes IL-10;dies naturally;proliferates in the LP
Epithelial	Healthy	Damaged by infectious bacteria;damaged by Th1 and Th17 cells; proliferates;secretes IL-6 and IL-12; dies naturally
	Damaged	Transitions to healthy state in the presence of IL-10; dies naturally
Bacteria	Infectious	Dies due to Th1 or Th17 or inflammatory macrophages or damaged epithelial cells;dies naturally;proliferates in the LP
	Tolerogenic	Moves from lumen to the epithelium in the presence of damaged epithelial cells;becomes infectious if moves in the LP compartment; proliferates in lumen and LP; dies naturally

Dies = is removed from the simulation; DC: dendritic cell; GLN: gastric lymph node; IFN-γ: interferon γ; IL: interleukin; iTreg: induced regulatory T cell; LP: lamina propria; TGF-β: transforming growth factor β; Th: T helper; Treg: regulatory T cell.

### Model description

ENISI-MSM is a multiscale ABM platform for computational immunology that was built on our previous work, ENISI-MSM [12], which integrated COPASI, the ODE solver, and ENISI, an agent-based simulator.

### Spatial discretization

The model has a spatial discretization such that we define the area being simulated as a simulation environment with a 2D grid of size 30 mm x 10 mm. Each individual lattice site is 1 mm x 1 mm; however, this is a configurable run parameter and can be changed without modifying the model. We further want to clarify that the aforementioned units in the model are annotations and purely aesthetic. The scales described in Table 1 in the previous version of ENISI-MSM [12] were kept unchanged .

**Figure 2:**
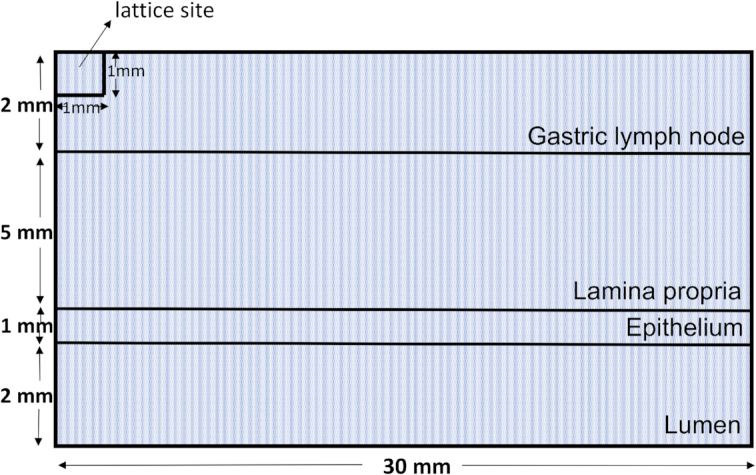
A pictorial representation of the spatial discretization of the 2D grid.

The 4 functionally and anatomically distinct sized compartments are separated by border implementation such that their dimensions are lumen (2 mm), epithelium (1 mm), LP (5 mm), and GLN (2 mm). The following compartments are adjacent to each other: lumen to epithelium, epithelium to LP, and LP to GLN. The spatial discretization is described in Figure.   2.

The parameters that define the initial concentration of the agents and the diffusivity of cytokines are obtained from a properties file ('model.props' in the 'Howtorunasimulation' folder in the GitHub repository). All the values of the parameters are listed in Table S1 alongwith detailed mechanism that each parameter corresponds to is described in the second column, "parameter description," of Table S1. We demonstrate below how we obtain a count of thousands of resident macrophages. For example, if the initial concentration of resident macrophages in the LP is 30, the total number of these resident macrophages can be calculated as

\begin{eqnarray*}
&&{n}({\rm{resident}}\ \ {\rm{macrophages}}) = {\rm{siz}}{{\rm{e}}_{{\rm{compartment}}}}\ \ ({\rm{lamina}}\,{\rm{propria}})\nonumber \\
&& \times {\rm{concentratio}}{{\rm{n}}_{{\rm{intial}}}}({\rm{resident}}\ \ {\rm{macrophages}}).
\end{eqnarray*}

\begin{equation*}
{n}({\rm{resident}}\ \ {\rm{macrophages}}) = (30 \times 5) \times 30 = 4,500.
\end{equation*}

### Time step size

**Figure 3:**
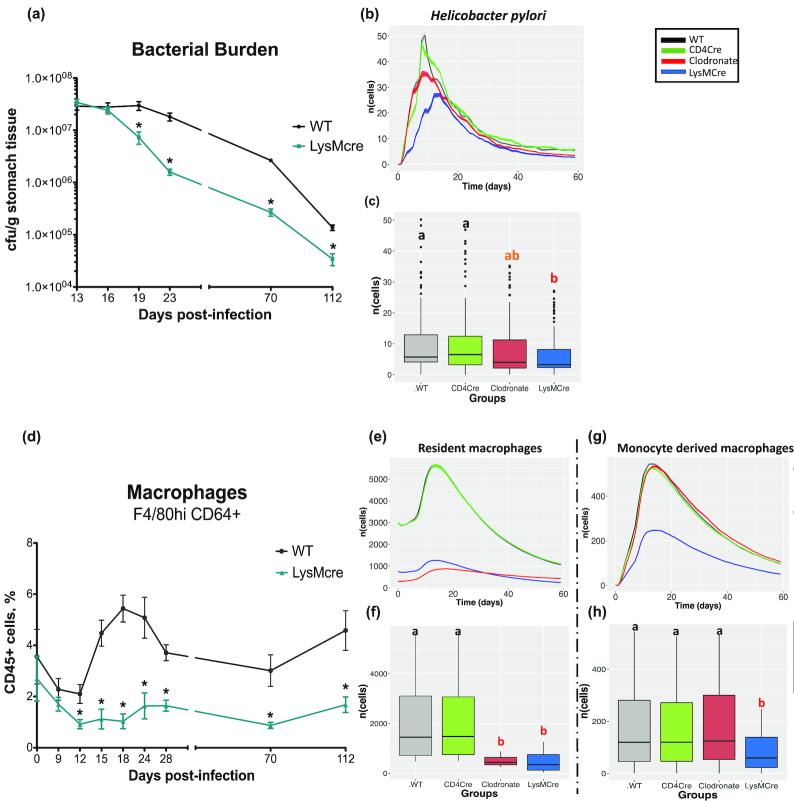
Time course simulations representing the immune response during *Helicobacter pylori* infection. The upper half of the plot in both panels shows the dynamics of the population cells over time representing the number of cells (y-axis) vs time (x-axis) in wild type (WT) (black), CD4Cre (green), clodronate (red), and LysMCre (blue) simulated *in silico* groups during *H. pylori* infection (see Results section, *Hybrid model simulations produce similar immune dynamics observed in previously published experimental data*for details about the groups). A side-by-side comparison with the bacterial load and macrophage population as observed in the mouse model of *H. pylori* infection is also included (***a*** and ***d***). The cell populations include (***b***) *H. pylori*, (***e***) the resident macrophages, and (***g***) monocyte-derived macrophages in the lamina propria compartment. The graphs in the lower half (***c, f, h***) of these panels show the results for statistical comparison between the groups using ANOVA with the post hoc analysis. The letters “a,” “ab,” and “b” represent statistically significant differences (*P <* 0.05) between the groups obtained after running the Tukey honestly significant difference. The groups with letter "a" are statistically significant different than the group with letter "b", groups with same letter are not statistically significantly different than each other. The group with letter "ab" is not statistically significantly different than group "a" and "b". In the box plots, the horizontal line is the median of the respective cell population, the box contains the interquartile range, the whiskers show the 95% confidence interval, and the dots are the outliers. cfu: colony-forming units.

The time step size is 1 tick ∼ 1 day, which was obtained during the process of qualitatively comparing the output to the results from the mouse model of *H. pylori* infection. For example, the peak of resident macrophages in the LP (see Figure.   **3e** and f) is observed at ∼21 days, which is similar to the results obtained in Fig. 2A described in [24] (also described in detail in Results section, *Hybrid model simulations produce similar immune dynamics observed in previously published experimental data*).

### Updating

Each agent has an “act” function within the code that describes the rules implemented for each of the agent groups. At every simulation cycle, each agent inspects its location and updates its state. If the agents were T cells and macrophages, they obtained the cytokine concentration from the ValueLayers and sent that information to COPASI, which calculated the differentiation subtype of the agent and cytokines to be secreted into the environment [12]. The input to the ODEs was the cytokine values at the agent's location. Thus, the intracellular ODE models were used to determine and update the state. Each agent proliferated, died, changed its state, and moved across the compartment, following the set of rules defined for them.

The COPASI set-up for the solver used the LSODA (Livermore Solver for Ordinary Differential Equations) differential equation solver. The default values for the set-up such as the relative tolerance (1e−6), absolute tolerance (1e−12), and maximum internal steps of 10,000 were maintained. The ENISI MSM sends the current concentrations of the cytokines to COPASI. COPASI uses those values to integrate the deterministic model for 1 tick, i.e., 1 day. The resulting time series of cytokine concentrations are used to update the cytokine value in the ABM/PDE system. COPASI simulates a different model for each relevant cell type.

The ENISI-MSM PDE solver uses a simple numerical scheme to solve the PDEs [29] and process the distributed value layer [30]. The ValueLayer stores the value for a grid space and provides methods to change the values of each individual lattice site. The Diffuser is used to diffuse the values of the ValueLayer using diffusion (d) and degradation (δ) constants as described by Mei el al. [12]. The diffusion constant determines the migration of values of a lattice site to its neighboring lattice site. The table below shows the constants used for each immediate neighbor during the PDE integration. Lets index the neighbors based on their position relative to the current lattice position, where the following table specifies the integration constants *K* for each neighbor relative to any lattice point. For example, here (0,0) is a lattice point and (-1,-1), (-1,0), (-1,1), (0,-1), (0,1), (1,-1),(1,0) and (1,1) are its relative neighbors.

**Table:** 

Relative location	-1	0	1
-1	0.3	1.2	0.3
0	1.2	−6.0	1.2
1	0.3	1.2	0.3

This leads to the equation shown below for each lattice point. Note that summing over the neighbors includes the lattice point (0,0). Here, *v_n_* is the value of the lattice site itself at step n, *deltaT*is the integration step, the values of *c*_δ_ and *c*_d_ are the degradation and diffusion constant, respectively. The PDE solver uses the above number scheme $K^\mathrm{neighbor}$ for the diffusion process. The step size is automatically adjusted at the beginning of the simulation based on the degradation and diffusion constants to avoid underflow errors; *i.e*., multiple PDE steps are in general executed per tick. The grid size is identical with the spatial discretization for the agents.

\begin{eqnarray*}
v_{n} = v_{ n-1} + deltaT * \lbrace c_{ d}*\sum[K^{ neighbor}*v_{ n-1}^{ neighbor}]-c_{ \delta}*v_{ n-1}\rbrace
\end{eqnarray*}

### Movement

The cells and bacterial agents presented in the model have Brownian motion and move randomly within the compartment. Brownian movement is an inherent property of a cell. Depending on cell phenotypes the movement can vary, but all cells with the same phenotype exhibit similar movements. Additionally, chemokine-driven movement is dependent on chemokine concentration in a tissue site. The capability of chemokine-driven movement exists in ENISI-MSM if the right chemokines are represented in the model. However, the focus of this model was to investigate changes in cell phenotype and not chemokine-driven movement of cells. Thus, the chemokines driving the movement are not represented in the present model. Cell migration is implemented in the code as the move() function for each of the cells and agents, which calls the moveRandom() function from the [31] file.

The hybrid model simulations were run on an Ivy Bridge-EX E7-4890 v2 2.80 GHz (3.40 GHz Turbo) with quad processor nodes. The code was parallelized such that the simulation time on a single node with 4 parallel tasks varied between 9 and 10 minutes. This runtime was based on the model parameters at the initiation stage, which included the numbers of immune cells, bacteria, epithelial cells, and time steps and the size of the 2D grid. To facilitate the investigation of the mechanisms underlying host responses during *H. pylori* infection, anatomical and functional compartments were spatially linked such that the agents had both unidirectional and bidirectional movement. All the agents worked in a synchronous format wherein the 2 agent populations (macrophages and T cells) made function calls to their respective ODE models [2, 23]. These agents used the varying cytokine concentration (i.e., environment variable) in their grid spaces as inputs to the ODE model, and these models were run using COPASI [28].

Table 2 shows information on the agents and the states that they can acquire.

**Table 2:** List of all the agents and the states they can acquire

Name of agent	States it can acquire	Name of the states in the hybrid model
*Helicobacter pylori*	0	*H. pylori*
Macrophage	0	Monocyte
	1	Resident
	2	Regulatory
	3	Inflammatory
Dendritic cell	0	Immature
	1	Effector
	2	Tolerogenic
T cell	0	Naive
	1	Th1
	2	Th17
	3	iTreg
	4	Tr
Epithelial cell	0	Healthy
	1	Damaged
Bacteria	1	Infectious
	2	Tolerogenic

All agents can acquire ≥1 and at most 5 states. The names chosen for the acquired states are closely related to their functional properties based on the underlying “rules".

Furthermore, we included the screenshots of 1 actual in silico simulation of *H. pylori* infection to highlight the spatiotemporal aspects of the modeling outputs. The time snapshots were created using VisIt version 2.12 [32], an interactive visualization and analysis tool. As shown in Additional file Fig. S2, the screenshots at time points 2, 4, 5, and 6 represent the spatial distribution of different agent cells over time across the 2D grid.

### Global sensitivity analysis

To conduct the global SA, we determined a list of 38 parameters to be varied that were selected on the basis of the calibration process (wherein the parameters that did not show much variation were not included). A range of values (maximum and minimum) was specified for each of the parameters (refer to Additional file Table S1) by expert judgment, summarized by bounded intervals. The practice of using expert judgment is recognized in the SA field [33]. As discussed by Thorne et al. [34], one of the challenges encountered in using ABM is the process of determining the parameter values; e.g., this may include the lack of the availability of experimental techniques to measure such parameters. The values of the parameters for the model presented here are obtained via the best guess based on the qualitative comparison of the computer model outputs with that of the experimental results obtained from the mouse model of *H. pylori* infection [24] (as described in detail in Results section, *Hybrid model simulations produce similar immune dynamics observed in previously published experimental data*). Because the source of the parameters is not known, we estimated the values to fit the data obtained from the mouse model of infection.

The values of these parameters were normalized within the range of 0 to 1 for SA purposes. We used a 2-stage metamodeling methodology to determine the influence of each input parameter on the model output, in a high dimensional screening setting inspired by Moon et al. [35]. The step-wise procedure is described in Additional file Fig. S3. All the files for global SA are freely accessible and can be downloaded from GitHub [36].

The 2-stage global SA is described in detail in the next section. To summarize, for the first stage the input parameter matrix was designed using the method described by Moon et al. [35] and simulations were run using the hybrid computer model. The simulation output from the first stage was analyzed using PRCC because it was computationally efficient, and the active inputs (significant effect) were screened to reduce the input parameter space. Second, the active parameters were varied whereas the inactive parameters from the first stage were maintained at a nominal value for the input parameter matrix design to be used for the second stage. Third, the simulation outputs from both stages were combined and used as a training dataset to fit a spatiotemporal metamodel. Fourth, the unknown model parameters for the spatiotemporal metamodel were estimated using the maximum log-likelihood function. The spatiotemporal metamodel was used as a substitute for the hybrid computer model, and the variance decomposition method was used to compute the Sobol’ total and first-order indices. Overall, we employed both approaches, PRCC based (for screening) and Sobol’ indices calculation, to perform a complete global SA of the hybrid computer model. The following sections explain the procedure step by step.

#### Design of 2-stage experiments and analysis

The inputs for the hybrid model are varying parameter values obtained from the design matrix and the outputs are the number of cells (agents) that vary over time. The first-stage experiment was focused on the screening of the input variables to reduce the number of input parameters to vary for the SA and to limit the computational cost. Computational costs are often a limiting factor that play an important role in the inclusion of model parameters in the SA [21]. For the design, we assumed the total number of input parameters under consideration *d* (in our case, 38). With an assumption of a maximum of 50% active inputs that is aimed to improve the screening performance, the number of runs for stage 1 was fixed to *n*_1_ = 4*d*, such that *n*_1_ > 5 × *d*× 0.5 = 2.5*d* as in Moon et al. [35]. To construct a *n*_1_ × (*n*_1_ − 1) preliminary input parameter design matrix, X* needed to be constructed ([35]). The input parameter design matrix for first-stage sampling was drawn from X*.

The algorithm for the first-stage design generated a design matrix *X*^(1)^ that satisfied the following 3 properties as in [35]:

- The columns of X^*^were uncorrelated, thereby facilitating the independent assessment of the effects due to the input parameters.
- The maximum and minimum value in each input parameter column were ensured to be 0 and 1, respectively, thereby preventing any input values with larger values to have a larger influence on the response, induced by the design.
- The designs defined by X^*^had “space-filling” properties such that all the regions of the input space were exhaustively explored.

#### First-stage sampling plan

The first-stage input parameter design matrix *X*^(1)^ was obtained by selecting the first *d* columns of X*, i.e., ${X^{( 1 )}} = ( {{\xi _1},\ \ldots .,\ {\xi _d}} )$. The hybrid computer model was run and the simulation outputs at these *n*_1_ design points were obtained.

In our case, the model comprised *d =* 38 input variables. The total number of distinct input parameter design points obtained using the above procedure was *n*_1_ = 152 = 4 ×*d*= 4 × 38. To account for the variability in the output, we ran 20 replicates *r*. Thus, the total number of simulations run using the hybrid model computer simulator with *X*^(1)^ as input parameter design matrix was *r*x*n*_1_*=* 20 × 152 = 3,040.

#### First-stage analysis

We analyzed the outputs from first-stage analysis and screened the active inputs from using PRCC. To measure the effect of the input parameters on output, we performed both PRCC and Spearman rank correlation coefficient (SRCC) analysis. PRCC and SRCC were chosen because they were computationally efficient (accounting for the low computational budget). A correlation analysis provides a measure of the strength of linear association between input and output variables [37]. The correlation coefficient between *x_j_* and *y* is calculated as follows:

\begin{equation*}
{r_{{x_j}y}} = \frac{{\mathrm{cov}\left( {{x_j},y} \right)}}{{\sqrt {\mathrm{var}\left( {{x_j}} \right)\mathrm{var}\left( y \right)} }} = \frac{{\mathop \sum \nolimits_{i = 1}^N \left( {{x_{ij}} - \bar{x}} \right)({y_i} - \bar{y})}}{{\sqrt {\mathop \sum \nolimits_{i = 1}^N {{\left( {{x_{ij}} - \bar{x}} \right)}^2}\mathop \sum \nolimits_{i = 1}^N {{\left( {{y_i} - \bar{y}} \right)}^2}} }},\
\end{equation*}

\begin{equation*}
j = 1,2, \ldots ,k.
\end{equation*}

where cov(*x_j_, y*) stands for the covariance between *x_j_* and *y* and var(*x_j_*) and var(*y*) are the variance of *x_j_* and *y*,respectively.

PRCC is performed when (i) a non-linear but monotonic relation exists between the input and outputs, and (ii) when little or no correlation exists between the input variables (which is guaranteed by the property [i] of our input parameter matrix, *X*^(1)^ described above). As described in Marino et al. [37], the PRCC between rank-transformed *x_j_* and *y* is the correlation coefficient between the 2 residuals $({x_j} - \widehat {{x_j})}$ and (${y_j} - \widehat {{y_j})}$, where $\widehat {{x_j}}\ $and $\widehat {{y_j}}$ are rank transformed and follow the linear regression models as follows:

\begin{eqnarray*}
\widehat {{x_j}} = {c_o} + \sum\nolimits_{\begin{array}{@{}*{1}{c}@{}} {p\ = \ j}\\ {p \ne j} \end{array}}^k {{c_p}{x_p}} \quad {\rm and} \quad \widehat {{y_j}} = {c_o} + \sum\nolimits_{\begin{array}{@{}*{1}{c}@{}} {p\ = \ j}\\ {p \ne j} \end{array}}^k {{c_p}{x_p}}.
\end{eqnarray*}

**Figure 4:**
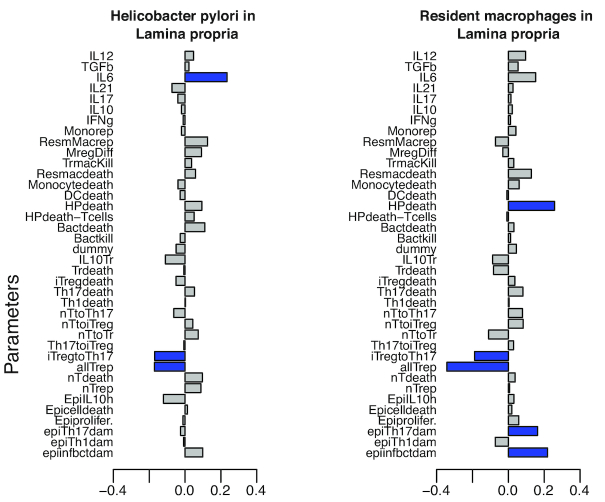
Bar plots for the partial rank correlation coefficients. The magnitude of the bar-plot indicates the value of the partial rank correlation coefficient. Blue bars indicate input parameters shown to be significantly different than 0 at *P <* 0.05 as influential, whereas the gray bars indicate the non-influential parameters on (a) *H. pylori* and (b) resident macrophages, in the lamina propria compartment. The detailed explanation of the abbreviations for the parameters are in Table S1.

We performed the PRCC analysis on the outputs obtained from the hybrid computer model with *X*^(1)^ as an input, using the “epi.prcc” package in R [38]. The significance test evaluated the strength of influence of each input parameter and assessed whether the PRCC coefficients were significantly different from zero [37]. We ran the PRCC analysis for 13 output cell populations ( Figure.   4 shows data for 2 output populations; the rest of the data are not shown) and identified the active input parameters using the significance test. PRCC and SRCC produced identical outputs; hence, results from SRCC are not shown here. If an input parameter was shown to be significant (*P*< 0.05) in 1 of the 13 output cell populations, it was considered as an active input for the second-stage input parameter design matrix. Additionally, domain expert knowledge was employed to include additional parameters, based on the biological significance, that were otherwise shown to be non-significant. In all, based on the PRCC analysis performed on the outputs obtained from the first-stage simulations and domain expert knowledge, we chose 23 input parameters as active inputs for the second stage (see Additional file Fig. S4). Thus, PRCC-screened inputs at significance level *P*< 0.05 and inputs based on expert knowledge were selected as active inputs to be varied for the second-stage sampling plan.

#### Second-stage sampling plan

The number of active inputs obtained from the first-stage analysis amounted to 23 parameters out of the initial set of 38 parameters. We followed the design described by Moon et al. [35] for the second stage and the number of design points amounted to *n*_2_= 100% × 5 × *a*, where *a* stands for the number of active inputs from the first stage. This resulted in *n*_2_= 23 × 5 = 115 parameter combinations for the second-stage input parameter design matrix. Because outputs from both stages are to be combined for second-stage analysis [35], the design for the second-stage was chosen to build on top of *X*^(1)^. The sampling phase design algorithm ensured that the columns satisfied the properties (i) (uncorrelated design points) and (ii) (between values 0 and 1) as listed in the previous section. We constructed the 115 × 38 (115 parameter settings and 38 parameters) design matrix for the second-stage that incorporated the 23 active inputs obtained from the PRCC screening in the first-stage output analysis. After combining the design points from both stages, the parameter design matrix *X* with space-filling properties contained 267 (152 from the first stage and 115 from the second stage) design points.

#### Second-stage analysis

We ran the computer code for the hybrid model with the second-stage input parameter design matrix (with 115 [*n*_2_] design points) for 20 (*r*) replicates, which amounted to 115 × 20 = 2,300 runs. The outputs from the first stage (152 × 20 runs) and second stage (115 × 20 runs) were combined to provide the training data to build a spatiotemporal metamodel. For the second-stage analyses, we utilized a metamodeling-based approach. Metamodels are surrogate models that can be used as a substitute for the simulation model [39]. The use of metamodels reduces the computational budget and cost of analysis and is a useful option in cases when the simulation model is expensive to run (in our case 9–10 minutes for 1 design point) [39]. The various metamodeling techniques used to build surrogates for a computer model output include linear regression models, neural networks, high dimensional model representation methods, GP regression models, polynomial chaos expansion, and more that are discussed in length by Rasmussen and Williams [40] and Santner et al. [41]. Among these, GPs are one of the most popular emulators because this method allows modeling of fairly complex functional forms. The GPs provide not only prediction at a new point but also an estimate of the uncertainty in that prediction [40]. A GP is a stochastic process for which any finite set of y-variables has a joint multivariate Gaussian distribution [42, 40]. Suppose ${y_j}( w )$, the simulation response obtained on the *j*th simulation replicate, at a design point $w\ = {( {{X^T},\ t} )^T}\ \in \ \chi \ $x ${\rm{{\rm T}}}$. It can be described as follows:

(1) \begin{eqnarray*}
{y_j}\left( w \right) = Y\left( w \right) + \ {\varepsilon _j}\ \left( w \right) = {\beta _0} + M\left( w \right) + {\varepsilon _j}\left( w \right),
\end{eqnarray*}

where *Y(w)* represents the mean function of ${y_j}( w )$, the quantity of interest that we intend to estimate at any design point *w*. The ${\beta _0}$ is a constant trend term and is assumed to be unknown. The input parameter $X \in \ \chi \subset \ {\mathbb{R}^d}$ and the time $t\ \in \ {\rm{{\rm T}}} \subset \ {\mathbb{R}_ + }$; and *X* is independent of *t*. The ${\varepsilon _j}( w )$ represents the sampling variability inherent in a stochastic simulation, which is assumed to be independent and identically distributed across the replications at any given design point [43].

The term $M( w )$ represents a stationary GP with mean = 0, and covariance between any points was modeled as the Gaussian covariance defined in [44]. Thus, the covariance between any design points ${w_a} = {( {{X_a}^T,\ {t_a}} )^T}\ $and ${w_b} = {( {{X_b}^T,\ {t_b}} )^T}\ $ in the random field can be modeled as

(2) \begin{eqnarray*}
\mathrm{cov}\left( {M\left( {{w_a}} \right),\ M\left( {{w_b}} \right)} \right) = {{\rm{\Gamma }}^2}{\rm{\ exp}}\left( - \mathop \sum \nolimits_{r\ = \ 1}^d {\theta _r}{\left( {{X_{ar}} - \ {X_{br}}} \right)^2}\right)R\left( {{t_a} - \ {t_b};{\rm{\gamma }}} \right),\nonumber \\
\end{eqnarray*}

where exp($- \mathop \sum \nolimits_{r\ = \ 1}^d {\theta _r}{( {{X_{ar}} - \ {X_{br}}} )^2}$) models the spatial correlation between 2 input design points $\ {X_a}$ and $\ {X_b}$ in the input parameter space, whereas $R( {{t_a} - \ {t_b};\ {\rm{\gamma }}} )\ $ also given by exp($- \mathop \sum \nolimits_{r\ = \ 1}^d {{\rm{\gamma }}_r}\ {( {{t_{ar}} - {t_{br}}} )^2}\ $ ) models the temporal correlation between time points ${t_a}\ $and ${t_b}.\ $The parameters $\theta $ and $\gamma $ represent the rate at which (i) spatial correlation decreases as the points move farther in space with the same time index and (ii) temporal correlation decreases as the time points are farther apart in time at the same input vector, respectively. Both the spatial correlation and temporal correlation are modeled using the Gaussian covariance. The parameter ${{\rm{\Gamma }}^2}$ can be interpreted as the variance of *M*(*w*) for all *w*. The input parameter design consists of $( {{w_a},\ {n_i}} )_{i\ = \ 1}^k$ design points to run independent simulations with replicates applied to each of the design points. Let $k\ \times \ 1$ denote a vector of sample averages of simulation responses given by $\bar{y} = {( {\bar{y}( {{w_1}} ),\ \bar{y}( {{w_2}} ),\ \ldots .,\ \bar{y}( {{w_k}} )} )^T}\ $, where $\bar{y}( {{w_i}} )$ is the resulting estimate of performance measure obtained at design point ${w_i}$ and $\bar{\varepsilon}( {{w_i}} )$ is the sampling variability inherent in a stochastic simulation [43]. The equations associated with $\bar{y}( {{w_i}} )$ and $\bar{\varepsilon }( {{w_i}} )$ are described in equation (3):

(3) \begin{eqnarray*}
&&\bar{y}({w_i}) = \frac{1}{{{n_i}}}\sum\nolimits_{j = 1}^{{n_i}} {{y_j}({w_i}) = Y({w_i}) + \bar{\varepsilon}({w_i})} \quad {\rm{and}}\nonumber \\
&& \bar{\varepsilon}({w_i}) = \frac{1}{{{n_i}}}\sum\nolimits_{j = 1}^{{n_i}} {{\varepsilon _j}({w_i})} ,\,i = 1,2, \ldots ,k.
\end{eqnarray*}

Similar as in Ankenman et al. [43], shown in equation (4), let ${\mathop \sum \nolimits_M}$ be the *k*x*k* covariance matrix across all design points and let $\mathop \sum \nolimits_M ( {{w_o},\ .} )\ $ be the *k*x 1 vector, (cov[*M*(*w*_0_, *w*_1_)], cov[*M*(*w*_0_, *w*_2_)], ..., cov[*M*(*w*_0_, *w_k_*)]^*T*^, that contains spatial covariance between the *k* design points and a given prediction point ${w_o}$. Also, let ${\mathop \sum \nolimits_\varepsilon }$ be the *k*x*k* covariance matrix of the vector of simulation errors associated with the vector of point estimates $\bar{y}$, across all design points. As described by Ankenman et al. [43], the best linear predictor $Y( {{w_o}} )$ that has the minimum mean squared error (MSE) among all linear predictors at a given point ${w_{o\ }} = \ {( {{X_o}^T,\ {t_o}} )^T}\ $ can be given by equation (4):

(4) \begin{equation*} \hat{Y}\ \left( {{w_o}} \right) = \widehat {{\beta _o}} + \ \mathop \sum \nolimits_M {\left( {{w_0},.} \right)^T}{\left( {{{\mathop \sum \nolimits_M}} + \ {{\mathop \sum \nolimits_\varepsilon} }} \right)^{ - 1}}(\bar{y} - {1_k}\widehat {{\beta _0}}), \end{equation*}

where 1_*k*_ is the *k*x 1 vector of ones and $\widehat {{\beta _o}}$ is estimated to be 1. The corresponding optimal MSE as in Ankenman et al. [43] is given by equation (5):

(5) \begin{eqnarray*}
\mathrm{MSE}\ \left( {\hat{Y}\left( {{w_o}} \right)} \right) = \mathop \sum \nolimits_M {X_0},{w_o} - \mathop \sum \nolimits_M {\left( {{w_{0,}}.} \right)^T}{\left( {{{\mathop \sum \nolimits_M}} + \ {{\mathop \sum \nolimits_\varepsilon} }} \right)^{ - 1}}\mathop \sum \nolimits_M \left( {{w_o},.} \right).\nonumber \\
\end{eqnarray*}

To implement the metamodeling approach as described above, the unknown model parameters are estimated through maximizing the log-likelihood function. The underlying standard assumption is that ${( {Y( {{w_o}} ),\ {{\bar{y}}^T}} )^T}$ follows a multivariate normal distribution (e.g., see [43] and [45]). The function implemented in the mlegp package in R [46] is used for the estimation of the parameters. Once the parameters are estimated the prediction then follows equations (4) and (5).

#### Sensitivity index calculation

To determine the effect of input variables on the output, we employed the variance decomposition method. These methods involve the decomposition of the variance of the output as a sum of the variance produced by each input parameter [42].

We independently generated 10,000 × 38 sampling matrices, such that the parameter combinations were generated via Latin hypercube sampling as described by Saltelli et al. [47]. Simulations were performed using the GP spatiotemporal model as described in the previous section *Second-stage analysis*, and the Sobol’ indices were computed as described in [48, 47]. The Sobol’ method quantitatively measured the contribution of each input parameter by computing the first order and total order index [47]. For output *Y*, input parameter matrix ${X_i}$, where *i* is the input parameters of the model, the Sobol’ indices are computed as follows:

\begin{equation*}
\mathrm{SI}_1^{Xi} = \frac{{V[E(Y|{X_i)}]}}{{V\left( Y \right)}}\ ,
\end{equation*}

and

\begin{equation*}
\mathrm{SI}_{\mathrm{tot}}^{Xi} = \frac{{V[E(Y|{X_{\sim i})}]}}{{V\left( Y \right)}}.
\end{equation*}

The Sobol’ first-order sensitivity index $\mathrm{SI}_1^{Xi}$ measures the impact of a single parameter on the model output, whereas the Sobol’ total order index measures the influence of ${X_i}$ including all the interactions with other parameters. The first-order indices were computed using the Sobol-Saltelli method [47, 49], whereas the total order indices were computed using Sobol-Jansen [47, 50].

## Results

### Hybrid model simulations produce similar immune response dynamics observed in previously published experimental data

We first aimed to simulate the findings observed in previous gut models [24] to ensure that we obtained similar response dynamics from the hybrid ENISI model of *H. pylori* infection. As in Viladomiu et al. [24], to demonstrate that the gastric mucosa harbors a system of macrophages that contribute to the outcome of *H. pylori* infection, we created an *in silico* peroxisome proliferator-activated receptor γ (PPARγ) macrophage-specific knockout (KO) model. PPARγ is an important transcription factor that controls the expression of genes that contribute to the inflammatory response once this is initiated. To disrupt the downregulation of pro-inflammatory responses, we simulated a PPARγ KO system in either macrophage or T-cell populations and compared the response to a wild-type (WT) system. In the model, we created 4 different macrophage populations, comprising “resident” macrophage agents that mimic the properties of the F4/80hi CD11b^+^CD64^+^CXCR1^+^ macrophages reported by Viladomiu et al. [24], monocyte-derived (infiltrating) macrophage populations with regulatory properties (M2, or alternatively activated), pro-inflammatory function (M1, or classically activated) and monocytes (see Table 2).

We simulated an *in silico H. pylori* infection by creating 4 groups, (i) a control—WT (representing a WT group), (ii) CD4Cre (T-cell−specific PPARγ KO—lacks PPARγ gene in all CD4 T cells), (iii) LysMCre (myeloid cell−specific PPARγ KO—lacks PPARγ gene in all macrophages), and (iv) clodronate group (simulating the removal of macrophages by chemical depletion via clodronate treatment). To simulate the CD4Cre group, the probabilities of a naive T cell transitioning to an iTreg cell (p_nTtoiTreg) and Th17 cell differentiating to iTreg (p_Th17toiTreg) were reduced to 5% and 10% of the control value, respectively (refer to Table S1). As described by Carbo et al. [23], to simulate the LysMCre experimental conditions, the probabilities of (i) a monocyte transitioning to a regulatory macrophage (p_Mregdiff) and (ii) immature DCs switching to tolerogenic DCs (p_iDCtotDC) were reduced to ∼60% and ∼30% of the control value, respectively (refer to Table S1). A complete set of parameters for each of the biological KOs are included as separate columns in Table S1. Last, the removal of macrophages by clodronate was simulated by decreasing the initial numbers of the macrophage population including the resident macrophages. The rationale to include the clodronate group (macrophage removal) was to evaluate whether depletion of phagocytic cells (terminology with respect to model, i.e., monocytes, residents, monocyte-derived regulatory , and inflammatory macrophages) would affect *H. pylori* colonization levels, as we have previously reported in an *in vivo* model [24]. Furthermore, to simulate the myeloid cell PPARγ KO system, the initial population of resident macrophages were also reduced.

All the groups were initialized with equal loads of *H. pylori* agents. Ten replicates of the simulations were performed for each of the input parameter settings specific to each group. The outputs were averaged, and standard errors of the means were plotted as ribbons (shaded regions) across the graphs. After running the 10 replicates of the time series *in silico* simulation, the hybrid model showed significantly (*P* < 0.05) higher levels of *H. pylori* in the WT and CD4Cre groups as compared to LysMCre KO and macrophage-depleted groups ( Figure.   3b and c).

In addition to the increase in *H. pylori*, the WT and CD4Cre *in silico* experimental groups had a higher population of resident as well as monocyte-derived regulatory macrophages as compared to the clodronate (macrophage-depleted) and LysMCre groups ( Figure.   3e, f, g, and h). The results in the mouse model indicated that between weeks 2 and 3 after infection a decrease in bacterial burden in the stomach of LysMcre mice was observed as shown in Fig. 1A of Viladomiu et al. [ 24]. The decrease in bacterial burden led to significant and sustained lower colonization levels when compared to WT and CD4Cre. Similar to the results observed in the mouse model, we observed a significant decrease ( Figure.   3b and c) in the bacterial burden in the simulated LysMcre group as compared to the simulated WT and CD4cre groups. Furthermore, the results from the mouse model indicated that a significant increase in numbers of F4/80 ^hi^CD11b^+^CD64^+^CX3CR1^+^ cells (herein referred to as resident macrophages) was observed in WT mice in comparison with LysMcre mice as shown in Fig. 2A and E of Viladomiu et al. [24]. These cells accumulated in the stomach mucosa starting on day 14 post-infection in the WT mice but not in the LysMcre mice. We observed a similar increase ( Figure.   3e, f, g, and h) in the number of resident macrophages as well as monocyte-derived macrophages in the simulated WT groups in comparison to the simulated LysMcre group. We estimated the parameter values to fit the data obtained from the mouse model of *H. pylori* infection. Thus, the observations were qualitatively similar to the findings of Viladomiu et al. [24], where the stomach of WT mice was enriched in a population of F4/80^+^CD11b^+^CD64^+^ myeloid cells, compared to LysMCre mice.

Overall, with the results in Figure.   3, we showed the ability of the hybrid model to replicate the experimental results of Viladomiu et al. [24], and these preliminary data were used as a base calibration setting for SA and other *in silico* findings.

### Partial correlation coefficient analysis screened the influential parameters

To reduce the computational complexity of varying an input parameter space of 38 parameters, we divided the SA process in 2 stages. For first-stage analysis, we utilized the PRCC regression−based SA method to screen the influential inputs and used it for the second-stage design of the experiments (see Methods section, *Global sensitivity analysis*). Using PRCC, we determined the impact of the input parameters on the output cell populations in the model. The parameters with significant correlation with *H. pylori* in the gastric LP compartment and resident macrophages are shown in Figure.   4, along with their PRCC values. The blue bars highlight the parameters that are significantly different than 0, at *P*< 0.05, compared with gray bars, which indicate no significant difference. It is important to note that at this stage the analysis using PRCC was non-temporal.

The SA from first-stage results showed that the epithelial damage due to infectious bacteria (epiinfbctdam), with a coefficient value of ∼0.2, was positively correlated with the colonization of *H. pylori* in the LP compartment, indicating the important role of epithelial cell damage during the course of infection, similar to our previous findings [51]. Another parameter included the probability of the release of interleukin-6 (IL-6) with a coefficient value in the range (0.3–0.4).

Next, the epithelial cell damage parameters [epiinftbctdam = (0.2–0.3), epiTh17dam = (0–0.2)] were shown to have positive influence on the resident macrophage cells, whereas the T-cell type transition parameters [p_iTregtoTh17 = (0.3–0.4) and p_Th17toiTreg = (0.1–0.2)] showed a negative impact on the resident macrophages. Similarly, we performed the PRCC analysis for all the cell populations under consideration during the infection (not shown).

The significant parameters (marked in blue bars) obtained from the SA of the output from the first-stage design of experiments (152 parameter settings with 20 replicates; see Methods section, *Global sensitivity analysis*) were selected to be varied for the second-stage design. All the selected inputs are shown in Additional file Fig. S4. In all, we obtained 23 active inputs from the first stage.

### Metamodel-based spatiotemporal sensitivity analysis

The outputs obtained after running the first (152 × 20 runs) and second (115 × 20 runs) stage simulations, wherein x 20 denotes the 20 replicates, were combined to be used as a training dataset. The combined output was utilized to build a GP-based spatiotemporal metamodel (see Methods section, *Global sensitivity analysis*), using the mlegp package in R [46].

**Figure 5:**
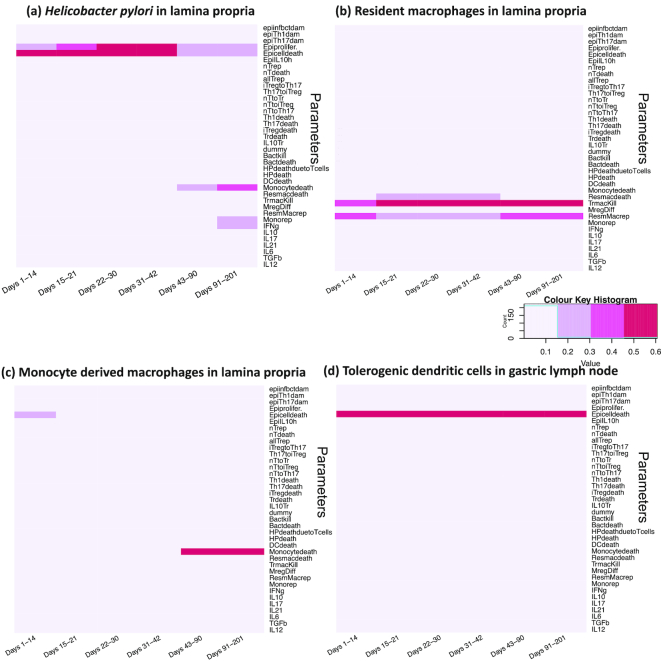
Heat maps of Sobol’ total order index for the input parameters across different output populations. The values in the heat map indicate the Sobol’ total order sensitivity index obtained from the metamodel for the 38 input parameters with respect to the cell populations. The values with darker color indicate a stronger influence on the cell population as compared to the ones with lighter shade that indicate non-influential parameters for the cell populations: (a) *H. pylori*, (b) monocyte-derived macrophages, and (c) resident macrophages in the lamina propria compartment and (d) tolerogenic DCs in the gastric lymph node compartment. The indexes are calculated over 6 time points ranging across the 3 stages of infection, including initiation (Day 1–14), peak (Days 15–42), and recovery stages (Days 43–201). The detailed explanation of abbreviations for the parameters are in Table S1.

The outputs from the training dataset were subdivided into 6 datasets, corresponding to 6 time periods (Days 1–14, 15–21, 22–30, 31–42, 43–90, 91–201), and averaged across these periods. The subdivision of output across the time periods aided the temporal analysis over the initiation (Days 1–14), peak of infection (Days 15–30), and chronic phase (after Day 31) stages [ 24]. We then fit a GP model (with nugget) and evaluated the performance of the fitting of the metamodel for *H. pylori*, resident macrophages, and monocyte-derived macrophages in the LP compartment, and tolerogenic DC in the GLN, using the diagnostic plots (see Additional file Fig. S5). After fitting the models, we performed variance-based global SA by computing the Sobol’ total order and first-order sensitivity index (see Methods section, *Global sensitivity analysis*). The estimates of the Sobol’ total order indices for the input parameters calculated over the 6 time periods are shown in Figure.   5.

As shown in Figure.   5a, the metamodel-based global SA showed that the input parameters, epithelial cell proliferation (Epiprolifer), and epithelial cell death (Epicelldeath) had the strongest impact on the population of *H. pylori* in the LP compartment. As time progressed from initiation of the infection (Days 1–14) through peak (Days 15–30), the epithelial cell proliferation had a continued impact on the colonization of *H. pylori*. Next, the influence of the probability of epithelial cell death decreased over the course of infection. Furthermore, Figure.   5b highlighted the impact of epithelial cell proliferation (Epiprolifer) and epithelial cell death (Epicelldeath) on the monocyte-derived macrophages.

For the resident macrophage population in the LP, which have emergent properties similar to those characterized in [24], we observed that the resident macrophage replication parameter (ResmMacRep) has an impact during the initiation and peak stages of the infection, which indicates that these subsets of macrophages replicate during the course of *H. pylori* infection. This result highlights the reliability of the 2-staged global SA method used here because these findings are consistent with those in [24] wherein we observed that these subsets of macrophages expand in the gastric stomach LP during the course of *H. pylori* infection.

Finally, for the tolerogenic DCs in Figure.   5d, we observed that epithelial cell death (Epicelldeath) seemed to have an impact. Another parameter that stands for the probability of naive T cell transitioning to iTreg cell (nTtoiTreg) was shown to have an impact on the tolerogenic DCs. Tolerogenic DCs are involved in the rule that transitions the naive T cells to iTreg cells in the GLN, and the stronger impact of the nTtoiTreg during the initiation and peak stages of the infection highlights the role of the tolerogenic DCs during the course of infection.

The global SA data suggested that the main contributors to the chronic colonization of *H. pylori* in the LP are epithelial cells, specifically the epithelial cell proliferation parameter.

### Effect of different ranges of epithelial cell proliferation

**Figure 6:**
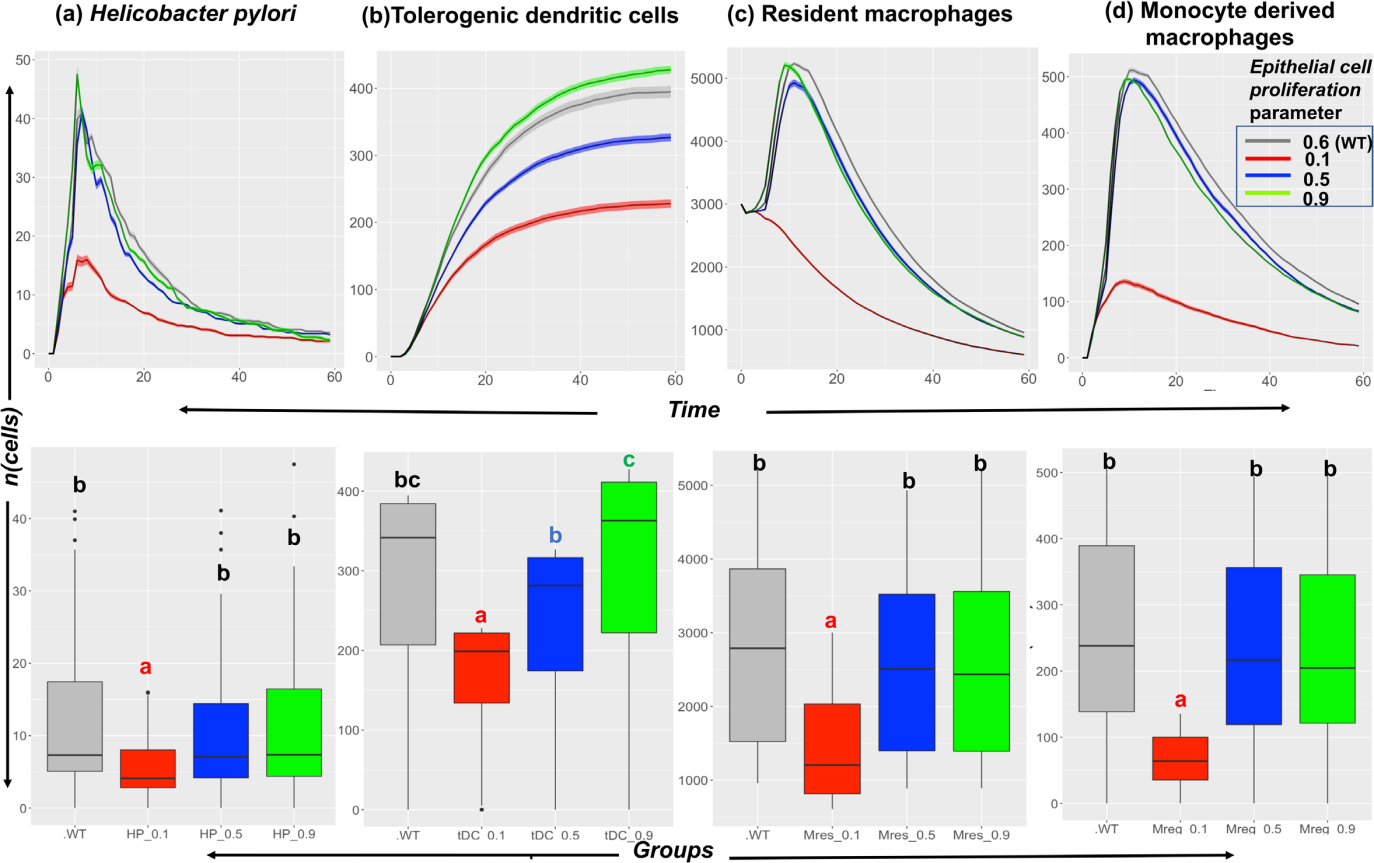
*In silico* study of the effect of the epithelial cell proliferation parameter on the cell populations. The plots show the effect of varying the epithelial cell proliferation (p_Epiprolifer) parameter (with values 0.1, 0.5, 0.6 [WT], and 0.9) on the output cell population of (a) *H. pylori*, (b) tolerogenic dendritic cells, (c) resident macrophages, and (d) monocyte-derived macrophages. The parameter has a decreasing effect on the cellular populations under consideration, wherein a decrease in the parameter value decreases the abundance of the cells over time. The lower half of each panel shows the results for statistical comparison between the groups using ANOVA with the post hoc analysis. The letters “a,” “b,” “c,” and “bc” represent statistically significant differences (*P <* 0.05) between the groups obtained after running Tukey's honestly significant difference. The groups with letter "a" are statistically significantly different than groups with letter "b" and "c". The group with letter "bc" are statistically significantly different different than group "a" but they do not differ from groups "b" and "c". The groups with same letter are not statistically significantly different than each other.

An interesting prediction derived from the metamodel-based global SA is that epithelial cell proliferation is one of the parameters that has a strong impact on the size of the *H. pylori* population. The biological hypothesis derived from this prediction is that epithelial cell proliferation is responsible for the higher colonization of *H. pylori*. Prior to conducting any experimental studies, we wanted to explore the hypothesis using our hybrid computer model *in silico* and study the model outputs obtained after we changed the epithelial cell proliferation parameter. Thus, we varied the epithelial cell proliferation parameter across different ranges (0.1–0.9, with 0.6 being the value for baseline conditions) and ran the simulations using the hybrid model and studied its effect on the different output cell population (obtained after running the simulations). These outputs were the ones obtained after running the simulation using the hybrid computer model, as we varied the epithelial cell proliferation parameter. We analyzed the outputs from the hybrid computer model and, interestingly, observed that upon decreasing the Epiprolifer from a range of values 0.9–0.1, the output cell populations with regulatory function, namely, regulatory macrophages and tolerogenic DCs, were found to vary. We observed a decreasing effect ( Figure.   6) on *H. pylori*, monocyte-derived macrophages, resident macrophages in the LP compartment, and tolerogenic DCs in the GLN. Overall, these cell populations varied due to the variation in the epithelial cell proliferation parameter.

For clarification, such connection was not embedded in the mechanisms included in Table 1, but it represents an emergent behavior from the simulations predicting the involvement of regulatory and tolerogenic DCs in the mechanisms of immunoregulation during *H. pylori* infection. Finally, the simulations targeting the epithelial cell proliferation resulted in changes in the regulatory and tolerogenic DC populations. This shows that the simulations indirectly targeted the regulatory and tolerogenic DC population. Thus, we hypothesize that epithelial cell proliferation might be responsible for the higher colonization of *H. pylori* through an immunoregulatory mechanism that involves regulatory macrophages and tolerogenic cells. This is in line with our own conclusions drawn from a previous article [24] where we show that the presence of cells with regulatory phenotype favors higher levels of *H. pylori* colonization. The results from the SA presented in this article suggest that epithelial proliferation might be a crucial part of the mechanisms by which these regulatory responses are induced and that there is a link between these parameters. The exact biological process however cannot be inferred from the current model and it will be investigated in follow-up *in vivo* studies.

The *in silico* findings suggested the involvement of regulatory macrophages (both resident as well as monocyte-derived) and tolerogenic DCs on the colonization *of H. pylori* in the gastric LP. This highlighted and validated the role of epithelial cell proliferation as one of the main factors affecting *H. pylori* levels in the gastric niche.

## Discussion

*H. pylori* is the dominant indigenous bacterium of the gastric microbiota. In the majority of individuals, *H. pylori* colonizes the stomach without causing adverse effects, with little to no activation of inflammatory pathways. However, certain members of the population lose immune tolerance to the bacterium thereby contributing to the development of chronic gastric diseases. The immunological mechanisms underlying its ability to persist in a harsh acidic gastric environment and its dual role as a pathogen and beneficial organism remain unknown. A subset of macrophages helps create a regulatory microenvironment that promotes the chronic colonization of *H. pylori* [24]. However, the immune regulatory mechanisms are incompletely understood. Computational models of the immune system featuring immune responses are powerful tools for testing the different “what-if” scenarios. Multiscale models of the immune response are attractive in terms of modeling the responses at different spatiotemporal scales [52].

In this study, we developed a HPC-driven hybrid, high-resolution, multiscale model to simulate the complex immunoregulatory mechanisms during *H. pylori* infection. The hybrid model was integrated with 2 intracellular ODEs capturing the dynamics of CD4^+^ T cells and regulatory macrophages. The inputs to the hybrid model are the set of parameters whose variation governs the immune system dynamics during infection. The obtained outputs were emergent patterns of different cell types, cytokines, and bacterial levels, e.g., the levels of *H. pylori*, and that qualitatively matched the patterns observed in an *in vivo* infection model [1, 24]. We presented an *in silico* framework that evaluated the global SA of the hybrid model and studied how the variation in the biological parameters affected the simulation outputs. The 2-stage global SA indicated that epithelial cell parameters, specifically, the proliferation of epithelial cells, affected the colonization of *H. pylori* in the gastric mucosa. These results were validated *in silico* and highlighted the involvement of regulatory macrophages and tolerogenic DCs in facilitating *H. pylori* colonization of the gastric mucosa. Previous studies highlighted that *H. pylori* inhabits the apical surfaces of the epithelial cells and maintains a persistent infection [53].

Furthermore, Mimuro et al. [54] demonstrated that *H. pylori* promotes epithelial gastric cell survival by attenuating apoptosis. These events showed how *H. pylori* regulated the gastric niche and utilized epithelial cells to facilitate its persistence within the stomach [54, 55]. Thus, the findings in the present study are in line with the literature that suggests that epithelial cell proliferation favors the colonization of *H. pylori* in the stomach.

Our group also showed another mechanism used by *H. pylori* to create a gut microenvironment that involved the induction of an IL-10−driven regulatory mechanism mediated by CD11b^+^F4/80^hi^CD64^+^CX_3_CR1^+^ mononuclear phagocytes, which facilitated bacterial colonization [24]. Additionally, in this article, we reported that regulatory macrophages were involved in the process of colonization with *H. pylori* when we varied the epithelial cell proliferation parameter *in silico*. Zhang et al. [56] demonstrated that *H. pylori* directed active tolerogenic programming of DCs that favored chronic bacterial colonization, by altering the balance of Th17/Treg cells. Rizzuti et al. [57] demonstrated that *H. pylori*−mediated IL-10 release caused the activation of signal transducer and activator of transcription 3 (STAT3) in DCs. This activation of STAT3 via IL-10 release was shown to induce the production of the tolerogenic DC phenotype. The findings from this article also indicated the involvement of tolerogenic DCs in affecting the mucosal levels of *H. pylori*. Therefore, the literature, combined with the results from this study, collectively suggests that during *H. pylori* infection, the epithelial cell favors the colonization of *H. pylori* by creating a regulatory microenvironment. This process is mediated by the regulatory macrophages and tolerogenic programming of DCs. Based on the results from this article and findings from the literature, this leads us to propose that the induction of IL-10 by the regulatory macrophages is potentially involved in directing the tolerogenic programming of DCs. All experimental evidence combined with our model prediction suggests the action of an underlying biological mechanism that links the presence of *H. pylori* in the gastric mucosa with changes in the rates of epithelial cell proliferation, which ultimately affects the levels of colonization. Our prediction points towards a link between epithelial cell proliferation and the action of tolerogenic DCs and regulatory macrophages. The exact cellular mechanism induced during this process however cannot be inferred from the current model, and it will be investigated in follow-up *in vivo* studies.

At its current stage, the hybrid ENISI model reproduces the overall immune system dynamics observed during an *H. pylori* infection. The parameters of calibrated ODEs were kept unchanged, whereas the ABM parameters were calibrated by qualitatively matching the patterns of the output simulations as observed in an *in vivo* model of *H. pylori* infection [24]. For ABM, its calibration and validation remain the major key issues, discussed elsewhere [21, 58, 59]. Furthermore, developing targeted methods of SA has been identified as an important challenge in the field [21, 60, 61]. In this article, we highlighted the use of SA methods with a 2-stage global SA framework composed of first screening the input parameters (using PRCC) and second building a surrogate model (using GP) of the hybrid model to understand the emergent behavior of the represented system. It is important to note that each SA method known has its own merits and produces useful information; however, none provides a complete picture of the emergent model behavior [21]. First, we employed PRCC methods as the initial step in our 2-staged SA that aided the screening of active inputs and reduced the parameter space. The choice of PRCC was advantageous and justified by the low computational cost and low complexity in the computation of the coefficients. Another advantage of the regression-based PRCC method is that the complex output from our hybrid model was condensed into a descriptive relationship that can be described by statistical measures such as *R^2^*[21]. As described by ten Broeke et al. [21], the results from PRCC are good descriptors of the outputs produced if the regression function constitutes a good fit to the output. However, if the function does not yield a good fit, the regression-based SA is proven to be useful in screening the influential parameters for further analysis [21], as described in our analysis.

Furthermore, the interaction effects between the parameters are not considered in regression-based methods, and hence it was followed by the use of variance-based methods in later stage analysis. Second, we employed a metamodeling-based approach and the Sobol’ method because they provided information on the interaction between the input variables and the use of metamodels allowed the sensitivity indices to be computed. One of the advantages of the Sobol’ method is that it is model-free and no fitting functions are used to decompose the output variance [39]. It considers the averaged effect of parameters over the whole parameter space but fails to explore the different patterns within the space [21]. Furthermore, the method is not suitable for quantification of output variability if the output distributions deviate from a normal distribution [21]. ten Broeke et al. [21] provide a detailed comparison of different SA methods used for the global SA of ABMs. Thus, we used both the PRCC and computation of Sobol’ indices approaches to evaluate the influence of the input parameter variation and identified the parameters involved in the successful colonization of the gastric niche by *H. pylori*.

Some limitations of the model include implementation through a 2D grid system and including all cells of the same size. Although we parallelize the computation of the hybrid model output, the large number of simulations required for the global SA compensates for the benefits of parallelization. To improve the calibration process and overall usability of the model, the data required for model calibration would include tissue biopsies from people infected with *H. pylori* that can be used to quantify the cells and take into account their spatial arrangement. The current version is also limited in terms of the interactions that are based on epithelial cells and DCs because they are strictly rule based. Building ODE models for these cells and integrating them with the ABM model will help capture the dynamics of epithelial cells and DCs more in depth. Overall, the immunoregulatory mechanisms underlying the chronic colonization of *H. pylori* and the predictive capacity of the model can be further improved by incorporating cell-specific models for epithelial cells and DCs.

In summary, a high-resolution, hybrid, multiscale spatiotemporal stochastic model of *H. pylori* infection was built and global SA was performed. The results from the global SA highlight the key role played by epithelial cells in affecting the levels of *H. pylori* colonization. The *in silico* validation of varying the epithelial cell proliferation parameter demonstrated the involvement of regulatory macrophages and the tolerogenic DCs. The next steps aimed to enrich the model will involve the validation of the findings *in vivo* to study the underlying mechanisms involved in the successful immune evasion by *H. pylori*. The computational modeling predictions will be further validated experimentally and clinically.

## Potential Implications

The computational model of the gut contains high-resolution information processing representations of immune responses that are generalizable for other infectious and autoimmune diseases. Complex diseases such as autoimmune disorders, infectious diseases, and cancer all require integration of multiscale-level data, information, and knowledge, ranging from genes, proteins, cells, and tissues to the organ level. The ENISI model of the gut presented here can be generalized to other diseases by implementing the agents and rules specific to that disease, plus recalibrating the model based on data that are specific to the new indication. Because ABMs have modular architectures, new agent types can be added and rules can be modified without restructuring the entire simulation set-up [19]. The use of ABM in such hybrid models not only facilitates the implementation of already known mechanisms but also helps validate and predict any unforeseen new mechanisms using data analytics methods such as global SA to analyze emerging behaviors at the systems level. The finer details regarding intracellular and intercellular interactions that contribute towards the nonlinear and complex behavior of the gut can also be studied by integrating the intracellular ODE models as implemented here.

## Availability of supporting data and materials

The datasets and files supporting the results of this article are available in the ENISI-MSM GitHub repository, RRID:SCR_016918 [25]. Further data supporting this work and snapshots of our code are available in the *GigaScience* repository, GigaDB [62].

## Availability of source code and requirements

- Project Name: ENISI MSM
- Project homepage: https://github.com/NIMML/ENISI-MSM
- Operating system(s): Linux, Mac OSX
- Programming language: C++, R, MATLAB
- Other requirements: CMake 3.7.2
- ENISI Dependencies: https://github.com/NIMML/ENISI-Dependencies
- License: Apache License 2.0
- RRID:SCR_016918

## Additional files

**File S1—**The detailed instructions to install ENISI MSM (Step I), run a simulation (Step II), and conduct SA (Step III) are described.

**Fig S1**. Design implementation of the hybrid multiscale model used to simulate *Helicobacter pylori* infection. The figure shows the class structure used in the ENISI MSM hybrid agent-based ODE model. Each group consists of an act() function that includes the implemented rule for each agent. The previously published ODE models for T cells and macrophages are used to integrate in the ABM code.

**Table S1** Table describing the input parameters used in the sensitivity analysis and their ranges used.

**Fig S2**. Time screenshots of an *H. pylori* infection modeled in a 30 mm (length) x 10 mm (width) 2D grid. The thickness of the compartment is shown on the y-axis, such that the lumen spans 0–2 units, epithelium spans 2–3 units, lamina propria spans 3–8 units, and gastric lymph node spans 8–10 units on the scale. The 2D distribution of different cell subsets over the time steps (ticks) 2, 4 (top panels), 5, and 6 (bottom panels) is shown. The insets in each image show a zoomed-in portion of the respective grids across the time steps 2, 4, 5, and 6. The agents represented in the screenshots below are only for visual representation and do not represent the actual size of the biological cells.

**Fig S3**. Flow chart for the 2-staged global sensitivity analysis.

**Fig S4**. The active and inactive inputs selected from the stage 1 analysis. The rows represent the input parameters and columns represent the output cell populations. The green boxes highlight the “active” input parameters (row) that are shown to have a significant influence (calculated based on the results obtained from partial correlation coefficient analysis) on an output cell (columns) under consideration.

**Fig S5**. Diagnostic and residual plots obtained for the Gaussian processes fitted metamodels. The upper panel represents the diagnostic Q-Q plots, where the open circles represent the cross-validated predictions and solid black lines represent observed response. The “observed simulations” data in the first half of the lower panel refer to the observed output values of the simulations obtained after running the hybrid computer model, whereas the y-axis refers to the predicted simulation values obtained from the cross-validated model. Each point represents 1 output point obtained as an output from the simulation. The second half of the lower panel refers to the standard residual plot wherein the x-axis represents the observed simulation values obtained from the simulation and the y-axis refers to the residual error [error (predicted values – observed values)/standard deviation (error)] obtained. The diagnostic plots denote the black circles, which are the cross-validated prediction. Cross-validation is in the sense that for predictions made at design point x, all observations at design point x are removed from the training set. The lower panel represents the residual plots for the cell populations: (a) *H. pylori*, (b) resident macrophages, (c) monocyte-derived macrophages in the LP, and (d) tolerogenic DCs in the GLN compartment.

## Abbreviations

ABM: agent-based model; DC: dendritic cell; ENISI MSM: Enteric Immunity Simulator multiscale modeling; GLN: gastric lymph node; GP: Gaussian process; HPC: high-performance computing; IFN-γ: interferon γ; IL: interleukin; iTreg: induced regulatory T; KO: knockout: LP: lamina propria; LSODA: Livermore Solver for Ordinary Differential Equations; ODE: ordinary differential equation; PDE: partial differential equation; PPARγ: peroxisome proliferator-activated receptor γ; PRCC: partial rank correlation coefficient; RRID: research identification initiative ID; SA: sensitivity analysis; SRCC: Spearman rank correlation coefficient; STAT3: signal transducer and activator of transcription 3; TGF-β: transforming growth factor β; Th: T helper; Treg: regulatory T; WT: wild type.

## Competing interests

The authors declare that they have no competing interests.

## Funding

This work was supported by the Defense Threat Reduction Agency (DTRA) grant HDTRA1–18-1–0008 to J.B.R. and R.H. and funds from the Nutritional Immunology and Molecular Medicine Laboratory (www.nimml.org). The funding body had no role in the design of the study, data collection, analysis, interpretation of data, and writing of the manuscript.

## Authors’ contributions

M.V., R.H., and J.B.R. formulated the model, implemented, performed the simulations, analyzed model-generated outputs, made the figures, and wrote the manuscript. M.V., A.L., J.B.R., R.H., and S.H. formulated the model. S.H., A.L., and V.A. implemented the code architecture and benchmarked the parallel version of the hybrid model. X.C. and M.V. wrote the codes for global sensitivity analysis and generated the design matrices. N.T.J. generated macrophage and *H. pylori* experimental data. J.B.R., V.A., and R.H. supervised the project. J.B.R. and R.H. edited the manuscript. J.B.R., A.L., N.T.J., S.H., V.A., X.C., and R.H. participated in discussions on the model and results. All authors provided critical feedback on the project.

## Supplementary Material

giz062_GIGA-D-18-00435_Original_Submission

giz062_GIGA-D-18-00435_Revision_1

giz062_GIGA-D-18-00435_Revision_2

giz062_GIGA-D-18-00435_Revision_3

giz062_GIGA-D-18-00435_Revision_4

giz062_Response_to_Reviewer_Comments_Original_Submission

giz062_Response_to_Reviewer_Comments_Revision_1

giz062_Response_to_Reviewer_Comments_Revision_2

giz062_Response_to_Reviewer_Comments_Revision_3

giz062_Reviewer_1_Report_Original_Submission Chang Gong -- 12/9/2018 Reviewed

giz062_Reviewer_1_Report_Revision_1 Chang Gong -- 1/28/2019 Reviewed

giz062_Reviewer_2_Report_Original_Submission Elsje Pienaar -- 12/17/2018 Reviewed

giz062_Reviewer_2_Report_Revision_1 Elsje Pienaar -- 1/31/2019 Reviewed

giz062_Reviewer_2_Report_Revision_2 Elsje Pienaar -- 2/18/2019 Reviewed

giz062_Reviewer_3_Report_Revision_2 Paul Macklin, Ph.D. -- 2/25/2019 Reviewed

giz062_Reviewer_3_Report_Revision_3 Paul Macklin, Ph.D. -- 3/21/2019 Reviewed

giz062_Reviewer_3_Report_Revision_4 Paul Macklin, Ph.D. -- 4/7/2019 Reviewed

giz062_Supplement_Files
