# Supplementary material for: High-resolution computational modeling of immune responses in the gut
Source: Gigascience. 2019 Jun 11;8(6):giz062. doi: 10.1093/gigascience/giz062 (PMC6559340; doi:10.1093/gigascience/giz062)
Supplement: giz062_GIGA-D-18-00435_Original_Submission [file giz062_giga-d-18-00435_original_submission.pdf]

GigaScience

High-Resolution Computational Modeling of Immune Responses in the Gut

--Manuscript Draft--

**Manuscript Number:**	GIGA-D-18-00435
**Full Title:**	High-Resolution Computational Modeling of Immune Responses in the Gut
**Article Type:**	Research
**Funding Information:**	
**Abstract:**	Background: Helicobacter pylori (H. pylori) causes gastric cancer in 1-2% of cases, but it exerts beneficial health effects including protection against allergies and gastroesophageal diseases. To examine the double edge sword of H. pylori as a pathogen and a commensal, we investigated the dynamics of immunoregulatory mechanisms triggered by H. pylori infection using a high-performance computing driven ENteric Immunity Simulator (ENISI) multiscale model. The immune responses were simulated in a high-resolution model integrating agent based model, ordinary and partial differential equations.<br>Results: The outputs were analyzed using two sequential stages wherein the first stage used a partial rank correlation coefficient regression based and the second employed a metamodel-based global sensitivity analysis. The influential parameters screened from the first stage were selected to be varied for the second stage. The outputs from both stages were combined as a 'training dataset' to build a spatiotemporal metamodel. The Sobol' indices measured the time-varying impact of input parameters during the initiation, peak and chronic phases of infection. The data analytics methods identified epithelial cell proliferation and epithelial cell death as key parameters that control infection outcomes. In-silico validation showed the colonization of H. pylori decreased with a decrease in epithelial cell proliferation mediated by regulatory macrophages and tolerogenic dendritic cells.<br>Conclusion: The hybrid model of H. pylori infection identified epithelial cell proliferation as a key factor for successful colonization of the gastric niche and highlighted the role of tolerogenic dendritic cells and regulatory macrophages in both modulating the host responses and shaping infection outcomes.
**Corresponding Author:**	Raquel Hontecillas, Ph.D.<br>Virginia Polytechnic Institute and State University<br>Blacksburg, Virginia UNITED STATES
**Corresponding Author Secondary Information:**	
**Corresponding Author's Institution:**	Virginia Polytechnic Institute and State University
**Corresponding Author's Secondary Institution:**	
**First Author:**	Meghna Verma, M.S.
**First Author Secondary Information:**	
**Order of Authors:**	Meghna Verma, M.S.
	Josep Bassaganya-Riera, Ph.D.
	Andrew Leber, Ph.D.
	Nuria Tubau-Juni, M.S.
	Stefan Hoops, Ph.D.
	Vida Abedi, Ph.D.
	Xi Chen, Ph.D.
	Raquel Hontecillas, Ph.D.
**Order of Authors Secondary Information:**	

Powered by Editorial Manager® and ProduXion Manager® from Aries Systems Corporation

Additional Information:	
**Question**	**Response**
Are you submitting this manuscript to a special series or article collection?	No
**Experimental design and statistics**<br><br>Full details of the experimental design and statistical methods used should be given in the Methods section, as detailed in our Minimum Standards Reporting Checklist. Information essential to interpreting the data presented should be made available in the figure legends.<br><br>Have you included all the information requested in your manuscript?	Yes
**Resources**<br><br>A description of all resources used, including antibodies, cell lines, animals and software tools, with enough information to allow them to be uniquely identified, should be included in the Methods section. Authors are strongly encouraged to cite Research Resource Identifiers (RRIDs) for antibodies, model organisms and tools, where possible.<br><br>Have you included the information requested as detailed in our Minimum Standards Reporting Checklist?	Yes
**Availability of data and materials**<br><br>All datasets and code on which the conclusions of the paper rely must be either included in your submission or deposited in publicly available repositories (where available and ethically appropriate), referencing such data using a unique identifier in the references and in the "Availability of Data and Materials" section of your manuscript.	Yes

Have you have met the above requirement as detailed in our Minimum Standards Reporting Checklist?	

Manuscript
Click here to download Manuscript Manuscript.docx

High-Resolution Computational Modeling of Immune Responses in the Gut

Meghna Verma[1,2], Josep Bassaganya-Riera[1], Andrew Leber[1], Nuria Tubau-Juni[1], Stefan Hoops[1], Vida Abedi[1], Xi Chen[3], Raquel Hontecillas[1,*]

[1]Nutritional Immunology and Molecular Medicine Laboratory, Biocomplexity Institute of Virginia Tech, Blacksburg, VA 24060, USA.
[2]Graduate Program in Translational Biology, Medicine and Health, Virginia Tech, Blacksburg, VA, 24061, USA.
[3]Grado Department of Industrial and Systems Engineering, Virginia Tech, Blacksburg, VA, USA.

*** Correspondence:**
Dr. Raquel Hontecillas
Email: rmagarzo@vt.edu

Keywords: agent-based model, dendritic cells, ordinary differential equation, Gaussian process, *Helicobacter pylori*, high performance computing, immune system, macrophages, metamodel, sensitivity analysis.

Email address for all authors:

Meghna Verma: meghna89@vt.edu
Josep Bassaganya-Riera: jbassaga@vt.edu
Andrew Leber: ajleber@vt.edu
Nuria Tubau-Juni: nuriaj@vt.edu
Stefan Hoops: shoops@vt.edu
Vida Abedi: vidaabedi@gmail.com
Xi Chen: xchen6@vt.edu
Raquel Hontecillas: rmagarzo@vt.edu

Abstract

Background: *Helicobacter pylori* (*H. pylori*) causes gastric cancer in 1-2% of cases, but it exerts beneficial health effects including protection against allergies and gastroesophageal diseases. To examine the double edge sword of *H. pylori* as a pathogen and a commensal, we investigated the dynamics of immunoregulatory mechanisms triggered by *H. pylori* infection using a high-performance computing driven **EN**teric **I**mmunity **Si**mulator (ENISI) multiscale model. The immune responses were simulated in a high-resolution model integrating agent based model, ordinary and partial differential equations.

Results: The outputs were analyzed using two sequential stages wherein the first stage used a partial rank correlation coefficient regression based and the second employed a metamodel-based global sensitivity analysis. The influential parameters screened from the first stage were selected to be varied for the second stage. The outputs from both stages were combined as a 'training dataset' to build a spatiotemporal metamodel. The Sobol' indices measured the time-varying impact of input parameters during the initiation, peak and chronic phases of infection. The data analytics methods identified epithelial cell proliferation and epithelial cell death as key parameters that control infection outcomes. *In-silico* validation showed the colonization of *H. pylori* decreased with a decrease in epithelial cell proliferation mediated by regulatory macrophages and tolerogenic dendritic cells.

Conclusion: The hybrid model of *H. pylori* infection identified epithelial cell proliferation as a key factor for successful colonization of the gastric niche and highlighted the role of tolerogenic dendritic cells and regulatory macrophages in both modulating the host responses and shaping infection outcomes.

1. Background

Computational modeling of the immune response dynamics can provide novel insights and facilitate the systems level understanding of the interactions at the gastric mucosa during infection. Ordinary differential equation (ODE-based methods are deterministic and based on the average response of cells over time. Dynamical models are used in immunology for system-level analyses of CD4+ T cell differentiation (Carbo, Bassaganya-Riera et al. 2013), macrophage differentiation (Leber, Bassaganya-Riera et al. 2016), immune responses elicited by *Clostridium difficile* infection (Leber, Viladomiu et al. 2015), co-infections (Verma, Erwin et al. 2017), and in cancer and immunotherapy (Qomlaqi, Bahrami et al. 2017). However, ODE based models lack the spatial aspects and the features to study the movement of immune cells over time. Agent-based models (ABM) employ a bottom-up approach that focuses on the spatial and temporal aspects of individual immune cells unlike the ODE-based methods. This rule-based method includes agents that act as local entities which interact locally with other agents, move in space, and follow set of rules representing their role in a given system and contribute towards generating an emergent behavior. Since, the immune system is a complex dynamical system (Vodovotz, Xia et al. 2017) wherein the components *i.e.* the immune cells move in space and time, ABMs are useful tools that can be employed to understand biological mechanisms and the hidden insights.

Helicobacter pylori is a gram-negative bacterium that has persistently colonized the human stomach since early evolution (Kusters, van Vliet et al. 2006) (Mane, Dominguez-Bello et al. 2010) and is currently found in over 50% (Cover and Blaser 2009) of the global population. It is known to be a major risk

factor in the development of peptic and gastric cancers in the form of adenocarcinoma, and gastric mucosa associated lymphoid tissue (MALT) lymphoma (Blaser 1992, Moss and Calam 1992, Asghar and Parsonnet 2001). Nonetheless, the vast majority of carriers (*i.e.* up to 75%) remain asymptomatic, only 15% develop ulcers, and less than 3% develop cancer. Further, recent experimental, clinical studies and epidemiological evidence suggest that *H. pylori* might provide protection against obesity-related inflammation and type 2 diabetes (Bassaganya-Riera, Dominguez-Bello et al. 2012), esophageal, cardiac pathologies, childhood asthma and allergies (Oertli, Sundquist et al. 2012) and autoimmune diseases. Due to the risk involved in developing cancer, the recommended international guidelines employ the “test and treat” policy wherein a person tested positive for *H. pylori* is recommended to be treated with antibiotics. Antibiotic resistance against *H. pylori* has risen significantly leading to therapy failure, for example, about 30% of *H. pylori* isolates in the US are resistant to at least one antimicrobial and 5% are multidrug resistant. Thus, it is crucial to understand the underlying immunoregulatory mechanisms and develop innovative approaches to design alternative therapeutics by investigating the dual role of *H. pylori* as commensal and pathogen. Computational models provide a cost-effective and predictive way to study the complex and dynamic immune system interactions and form non-intuitive novel hypothesis. Solving the complex puzzle of immunoregulatory mechanisms that include large spatiotemporal scales ranging from cellular, intracellular, tissue and organ level scales is a major unsolved challenge that requires applying computational modeling and data analytics.

An advanced hybrid model used to study the mucosal immune response during gut inflammation highlighted the mechanisms by which effector CD4+ T

cell responses, contributed to tissue damage in the gut mucosa following immune dysregulation (Mei, Abedi et al. 2015). Other hybrid models with integration of ABM, ODE and PDE technologies, were developed to understand the dynamics of tumor development (Gong, Milberg et al. 2017) and tumor growth models (Wang, Birch et al. 2009). These combined techniques have been used to develop multi-organ models in various situations, including the study of granuloma formation (Marino, El-Kebir et al. 2011) and pressure driven ulcer formation in post spinal cord injury patients (Solovyev, Mi et al. 2013). The summary of different agent-based simulators with immunology related applications are discussed and summarized in (Bassaganya-Riera 2015, Cappuccio, Tieri et al. 2016). The comparison between different multiscale modeling tools, agent-based immune simulators, are discussed in (An, Mi et al. 2009, Mei, Abedi et al. 2015).

In this study, we utilize a high-resolution **EN**teric **I**mmunity **SI**mulator (ENISI)-based model of the stomach for simulating the mucosal immune responses to *H pylori* infection. The advanced hybrid multiscale modeling platform ENISI multiscale model (MSM) is capable of scaling up to 10^{12} agents (Abedi, Hontecillas et al. 2015). The host immune responses initiated during *H. pylori* infection and the underlying immunoregulatory mechanisms are captured using the ENISI multiscale hybrid model. The underlying intracellular mechanisms that control cytokine production, signaling and differentiation of macrophages and T cells are modeled by using ODEs, the diffusion of cytokine values is modeled using PDEs and the location and interactions among the immune cells, bacteria and epithelial cells are modeled by using ABMs. The hybrid model thereby represents a high-performance computing (HPC)-driven large-scale simulation of the massively interacting cells and molecules in the immune system,

integrating the multiple modeling technologies from molecules to systems across multiple spatiotemporal scales.

To understand the dynamics and emergent immunological patterns described by this hybrid model, we employed sensitivity analysis *(SA)*, an important part of the model analysis used to explore the influence of varying model parameters on the simulation outputs. The influence of the effects of changes in parameter values on the model output explains the model dynamics that underlay the outputs (Ligmann-Zielinska, Kramer et al. 2014, Ten Broeke, Van Voorn et al. 2016). Furthermore, *SA* examines the robustness of the model output at different range of parameter values that correspond to a range of different assumptions. We employed global *SA* and conducted a two-stage spatiotemporal global *SA* approach. First, we used a regression-based method such as the partial rank correlation coefficient (PRCC) and screened the important input parameters that were shown to have the most influence on the output cell populations obtained from the hybrid model. Second, the screened input parameters from the first stage were varied to build a second stage parameter design matrix, and the computer simulations were again run using the hybrid ENISI model. The outputs from both analytics stages were combined and used as a 'training dataset' to build a spatiotemporal Gaussian process based metamodel. Finally, variance-based decomposition global *SA* was used to compute the Sobol' indices and the most influential parameters over the course of infection were identified. The data analytics methods conducted on the hybrid model identified the epithelial cell parameters such as epithelial cell proliferation as the most influential ones, required for the successful colonization of *H.* pylori in the gastric microenvironment.

2. Methods

2.1 Hybrid multiscale *Helicobacter pylori* infection model

We developed a multi-compartment, high-resolution, hybrid ABM/ODE/PDE model to capture the dynamics of the immune response during *H. pylori* colonization of the gastric mucosa. The model was defined in an area with 30 * 10 two-dimensional grid cell. The grid was divided into 4 functionally and anatomically distinct compartments: lumen, epithelium, lamina propria and gastric lymph node. These compartments were spatially linked through the use of a border implementation, that permitted the migration of agents (cells) across compartments. This facilitated the unidirectional and bidirectional movement of agents. At the cellular scale, ENISI MSM, simulated epithelial cells, macrophages, dendritic cells (DC), CD4+ T cells and bacteria that are implemented as agents in the model. At the intracellular scale, calibrated ODE based models of T cells (Carbo, Hontecillas et al. 2013) and macrophages (Leber, Bassaganya-Riera et al. 2016) were used to represent the intracellular pathways controlling cytokine production. Cytokines secreted by immune cells and their change in concentration were modeled by PDE. The evaporation constant for the cytokines determined the degradation value and the diffusion constant determined the spread of the cytokine value of one grid cell to its neighboring grid cell similar as in (Mei, Abedi et al. 2015). The features of ABM, ODE and PDE were combined to create a multiscale modeling environment which spanned across different orders of spatiotemporal scales. The parameters of the calibrated ODEs were kept unchanged, and the ABM parameters were calibrated by approximating the output simulations such that they qualitatively

resembled the patterns observed in an *in vivo* model of *H. pylori* infection (Viladomiu, Bassaganya-Riera et al. 2017).

The code for the hybrid model is freely accessible and can be downloaded at https://github.com/NIMML/ENISI-MSM. The design of the implementation of the code structure is depicted in the Additional file ***Fig S1***. The hybrid model is implemented in C++ and utilized the Repast HPC library (Collier and North 2011). For the ODEs, we utilized COPASI (Hoops, Sahle et al. 2006), an ODE-based modeling tool used in computational biology. The rules in the model that described the interaction of *H.* pylori with the gastric mucosa and the immune responses resulting from the infection are derived from the findings in our previously published studies (Carbo, Bassaganya-Riera et al. 2013, Leber, Bassaganya-Riera et al. 2016). Specifically, this hybrid model reproduced the immune responses generated by the interaction *H. pylori* and the resident macrophages as shown in (Viladomiu, Bassaganya-Riera et al. 2017). The rules for each cell type in the *H. pylori* infection are summarized in ***Table 1****.* A pictorial representation of the rules is depicted in *Fig 1.* These cell types represented as agents, act according to the rules (as in ***Table 1*)** that are updated at discrete simulation cycle. At the beginning of each simulation cycle, these agents were randomly placed separated by the four compartments within the two-dimensional grid. Each agent has an *'act'* function within the code, that describes the rules implemented for each of the agent groups. At every simulation cycle each agent inspected its location, obtained the cytokine concentration and updated its state. If the agents were T cells and macrophages, the intracellular ODE models were utilized to determine and update the state. The input to the ODEs were the cytokine values at the agent's location. Each agent proliferated, died, changed

its state and moved across the compartment, following the set of rules defined for them.

Fig 1. ***Helicobacter pylori*** **infection schematic diagram of the hybrid ABM ODE model**

The model comprises of four compartments, i) the lumen that contains H. pylori and bacteria, ii) epithelium that contains epithelial cells and dendritic cells, iii) lamina propria that contains variety of immune cells including the infiltrating effector (eDCs) and tolerogenic (tDCs) dendritic cells, monocytes, regulatory macrophages (both resident and monocyte derived macrophages), T helper cells and naïve CD4+ T cells (nT) and iv) gastric lymph node compartment that contains eDCs, tDCs, Th1, Th17, iTreg and nT. Two calibrated ODEs for T cells and regulatory macrophages are integrated as the ODE components in the hybrid model. The cellular agents are simulated in a two-dimensional grid space with their behavior defined by a set of rules during a course of H. pylori infection.

The hybrid model simulations were run on an Ivy Bridge-EX E7-4890 v2 2.80 GHz (3.40 GHz Turbo) quad processor nodes. The code was parallelized such that the simulation time on a single node with 4 parallel tasks, varied between 9-10 minutes. This run time was based on the model parameters at the initiation stage, which included the number of immune cell, bacteria, epithelial cells, number of time steps, and size of the two-dimensional grid. To facilitate the investigation of the mechanisms underlying host responses during *H. pylori* infection, anatomical and functional compartments were spatially linked such that

the agents had both unidirectional and bidirectional movement. All the agents worked in a synchronous format wherein the two agent populations (macrophages and T cells) made function calls to their respective ODE models (Leber, Bassaganya-Riera et al. 2016) (Carbo, Hontecillas et al. 2013). These agents used the varying cytokine concentration (*i.e.* environment variable) in their grid spaces as inputs to the ODE model and these models were run using COPASI (Hoops, Sahle et al. 2006). ***Table 2*** shows information on the agents and the states that they can acquire.

**Name of agents**	**States it can acquire**	**Name of the states in the hybrid model**
Helicobacter pylori	0	*H. pylori*
Macrophages	0	Monocyte
	1	Resident
	2	Regulatory
	3	Inflammatory
Dendritic cells	0	Immature
	1	Effector
	2	Tolerogenic
T cell	0	Naïve
	1	Th1
	2	Th17
	3	iTreg
	4	Tr
Epithelial	0	Healthy
	1	Damaged
Bacteria	1	Infectious
	2	Tolerogenic

Table 2. List of all the agents and the states they can acquire.

All the agents can acquire at least 1 and at the most 5 states. The names chosen for the acquired states, are closely related to their functional properties based on the underlying "rules".

2.2 Global sensitivity analysis

To conduct the global *SA,* we determined a list of 38 parameters to be varied that were selected based on the calibration process (wherein the parameters that did not show a lot of variation were not included). A range of values (maximum and minimum) was specified for each of the parameters by expert judgement, summarized by bounded intervals (Saltelli, Tarantola et al. 2000) (refer to Additional File ***Table S1***). The values of these parameters were normalized within the range of 0 and 1 for *SA* purposes. We employed a two-stage metamodeling methodology to determine the influence of each input parameter to the model output, in a high dimensional screening setting inspired by (Moon, Dean et al. 2012). The step-wise procedure is described in the Additional file, *Fig S2.* All the files for global *SA* are freely accessible and can be downloaded at https://github.com/NIMML/Sensitivity-Analysis.

The two-stage global *SA* is described in detail in the below section. To summarize, for the first stage the input parameter matrix was designed using the method described in Moon, Dean et al. 2012 and simulations were run using the hybrid computer model. The simulation output from first stage, was analyzed using PRCC as it was computationally efficient, and the active inputs (significant effect) were screened to reduce the input parameter space. Second, the active parameters were varied whereas the inactive parameters from the first stage, were maintained at a nominal value for the input parameter matrix design to be employed for the second stage. Third, the simulation outputs from both stages were combined and used as a ‘training dataset’ to fit a spatio-temporal metamodel. Fourth, the unknown model parameters for the spatio-temporal metamodel were estimated using maximum log-likelihood function. The spatio-temporal metamodel was used as a substitute for the hybrid computer model and the variance-decomposition method was used to compute the Sobol’ total and

first order indices. Overall, we employed both approaches, PRCC based (for screening) and Sobol' indices calculation to perform a complete global *SA* of the hybrid computer model. The following sections below, describe detailed step by step explanation of the procedure.

<u>*Design of two-stage experiments and analysis*</u>

The input for the hybrid model are varying parameter values obtained from the design matrix and the output are the number of cells (agents) that vary over time. The first stage experiment was focused on the screening of the input variables in order to reduce the number of input parameters to vary for the *SA* and to limit the computational cost. Computational costs are often a limiting factor that play an important role in the inclusion of model parameters in the *SA (Ten Broeke, Van Voorn et al. 2016).* For the design, we assumed the total number of input parameters under consideration as *d* (in our case, 38). With an assumption of a maximum of 50% active inputs that is aimed to improve the screening performance, the number of runs for stage 1, were fixed to $n_1 = 4d$, such that $n_1 > 5*d*0.5 = 2.5d$ as in (Moon, Dean et al. 2012). To construct a $n_1 * (n_1-1)$ preliminary input parameter design matrix, X^*, needed to be constructed ((Moon, Dean et al. 2012)). The input parameter design matrix for first stage sampling was drawn from X^*.

The algorithm for the first stage design, generated a design matrix $X^{(1)}$ that satisfied the below three listed properties as in (Moon, Dean et al. 2012)

i) The columns of X^* were uncorrelated thereby facilitating the independent assessments of the effects due to the input parameters.

ii) The maximum and minimum value in each input parameter column were ensured to be 0 and 1 respectively, thereby preventing any input values with larger values to have a larger influence on the response, induced by the design.

iii) The designs defined by X^* had "space-filling" properties such that all the regions of the input space were exhaustively explored.

First stage sampling plan:

The first stage input parameter design matrix $X^{(1)}$ was obtained by selecting the first *d* columns of X^*, *i.e.* $X^{(1)} = (\xi_1, \dots., \xi_d)$. The hybrid computer model was run and the simulation outputs at these $n_1$design points were obtained.

In our case, the model comprised of *d* = 38 input variables. The total number of distinct input parameter design points obtained using the above procedure was n_1 = 152 (4**d* = 4* 38). To account for the variability in the output, we run 20 replicates (*r)*. Thus, the total number of simulations run using the hybrid model computer simulator with $X^{(1)}$ as input parameter design matrix, were $r \times n_1$ = 20 x 152 = 3040.

First stage analysis

We analyzed the outputs from first stage analysis and screened the active inputs from using PRCC. To measure the effect of input parameter on output, we performed both PRCC and the spearman rank correlation coefficient (SRCC) analysis. PRCC and SRCC were chosen because they were computationally efficient (accounting for the low computational budget). A correlation analysis provides a measure of the strength of linear association between input and

output variable (Marino, Hogue et al. 2008). A correlation coefficient between x_j and y is calculated as follows:

$$r_{x_j y} = \frac{Cov(x_j, y)}{\sqrt{Var(x_j)Var(y)}} = \frac{\sum_{i=1}^{N}(x_{ij} - \overline{x})(y_i - \overline{y})}{\sqrt{\sum_{i=1}^{N}(x_{ij} - \overline{x})^2 \sum_{i=1}^{N}(y_i - \overline{y})^2}}$$

$$j = 1,2, \dots, k.$$

where, $Cov(x_j, y)$ stands for the covariance between x_j and y, and *Var* (x_j) and *Var (y)* are the variance of x_j and *y respectively*.

PRCC is performed when i) a non-linear but monotonic relation exists between the input and outputs, and ii) when little or no correlation exists between the input variables (which is guaranteed by the property (i) of our input parameter matrix, $X^{(1)}$ described above). As described in Marino, Hogue et al. 2008, the PRCC between rank transformed x_j and y is the CC between the two residuals $(x_j - \widehat{x_j})$ and $(y_j - \widehat{y_j})$ where $\widehat{x_j}$ and $\widehat{y_j}$ are rank transformed and follow the linear regression models as follows:

$$\widehat{x_j} = c_o + \sum_{\substack{p=j \\ p\neq j}}^{k} c_p x_p \text{ and } \widehat{y_j} = c_o + \sum_{\substack{p=j \\ p\neq j}}^{k} c_p x_p \text{ .}$$

We performed the PRCC analysis on the outputs obtained from the hybrid computer model with $X^{(1)}$ as an input, using '*epi.prcc'* package in R (https://cran.r-project.org/web/packages/epiR/epiR.pdf). The significance test evaluated the strength of influence each input parameters and assessed if the PRCC coefficients were significantly different that zero (Marino, Hogue et al. 2008). We run the PRCC analysis for 13 output cell populations (*Fig 3* shows data for 2

output populations and the rest of the data not shown), and identified the active input parameters using the significance test. PRCC and SRCC produced identical outputs, hence results from SRCC are not shown here. If an input parameter was shown to be significant (*P < 0.05)* in one of the 13 output cell populations, it was considered as an active input for the second stage input parameter design matrix. Additionally, domain expert knowledge was employed to include additional parameter based on biological significance that were otherwise shown to be non-significant. In all, based on the PRCC analysis performed on the outputs obtained from the first stage simulations and domain expert knowledge, we chose 23 input parameters as active inputs for the second stage (see Additional file S3). Thus, PRCC screened inputs at significance level $p < 0.05$ and inputs based on expert knowledge were selected as active inputs to be varied for the second stage sampling plan.

Second stage sampling plan:

The number of active inputs obtained from the first stage analysis amounted to 23 parameters out of the initial set of 38 parameters. We followed the design described in (Moon, Dean et al. 2012) for the second stage and the number of design points amounted to, n_2= 100%* 5*a* where '*a'* stands for the number of active inputs from the first stage. This resulted into n_2= 23*5 = 115 parameters combinations for the second stage input parameter design matrix. Since outputs from both stages are to be combined for second stage analysis, per (Moon, Dean et al. 2012), the design for the second stage was chosen to build on top of $X^{(1)}$. The sampling phase design algorithm ensured that the columns satisfied the properties (i) (uncorrelated design points) and (ii) (between values 0 and 1) as

listed in the previous section. We constructed the 115 x 38 (115 parameter setting and 38 parameters) design matrix for the second stage that incorporated the 23 active inputs obtained from the PRCC screening in the first stage output analysis. After combining the design points from both the stages, the parameter design matrix *X* with space filling properties contained 267 (152 from first stage and 115 from second stage) design points.

<u>Second stage analysis</u>

We run the computer code for the hybrid model with second stage input parameter design matrix (with 115 (n_2) design points), for 20 (r) replicates, which amounted to 115 x 20 (2300) runs. The outputs from the first stage (152 x 20 runs) and second stage (115 x 20 runs) were combined to provide the ‘training data’ to build a spatio-temporal metamodel. For the second stage analyses we utilized a metamodeling based approach. Metamodels are surrogate models that can be used as a substitute for the simulation model (Saltelli, Ratto et al. 2008). The use of metamodels reduces the computational budget, cost of analysis, and are useful options in cases when the simulation model is expensive to run (in our case 9-10 minutes for 1 design point) (Saltelli, Ratto et al. 2008). The various metamodeling techniques used to build surrogates for a computer model output include linear regression models, neural networks, high dimensional model representation methods, Gaussian process (GP) regression models, polynomial chaos expansion and more that are discussed in length in (Rasmussen and Williams 2006, Santner, Williams et al. 2013). Amongst these, GPs are one of the most popular emulators as it allows modeling of fairly complex functional form and not only provides a prediction at a new point but also an estimate of the

uncertainty in that prediction (Rasmussen and Williams 2006). A GP is a stochastic process for which any finite set of y-variables has a joint multivariate Gaussian distribution (Thiele, Kurth et al. 2014) (Rasmussen and Williams 2006). Suppose, $y_j(w)$, the simulation response obtained on the *j*th simulation replicate, at a design point $w = (X^T, t)^T \in \chi \times \mathrm{T}$, it can be described as follows:

$$y_j(w) = Y(w) + \varepsilon_j(w) = \beta_0 + M(w) + \varepsilon_j(w), \quad (1)$$

where *Y(w)* represents the mean function of $y_j(w)$, the quantity of interest that we intend to estimate at any design point *w.* The β_0 is a constant trend term and is assumed to be unknown. The input parameter $X \in \chi \subset \mathbb{R}^d$ and the time $t \in \mathrm{T} \subset \mathbb{R}_+$; and X is independent of t. The $\varepsilon_j(w)$ are represents the sampling variability inherent in a stochastic simulation, that are that are assumed to be independent and identically distributed across the replications at any given design point (Ankenman, Nelson et al. 2010).

The term $M(w)$ represents a stationary Gaussian process with mean = 0 and covariance between any points was modeled as the Gaussian covariance defined in (Lamoureux, Mechbal et al. 2014). Thus, the covariance between any design points $w_a = ({X_a}^T, t_a)^T$and $w_b = ({X_b}^T, t_b)^T$in the random field can be modeled as-

$$Cov\big(M(w_a), M(w_b)\big) = \Gamma^2 \exp(-\sum_{r=1}^{d} \theta_r (X_{ar} - X_{br})^2 \, R(t_a - t_b; \gamma), \quad (2)$$

wherein, $\exp(-\sum_{r=1}^{d} \theta_r (X_{ar} - X_{br})^2$) models the spatial correlation between two input design points X_a and X_b in the input parameter space, whereas $R(t_a - t_b; \gamma)$ also given by exp $(-\sum_{r=1}^{d} \gamma_r \, (t_{ar} - t_{br})^2)$ models the temporal

correlation between time points t_a and t_b. The parameters θ and γ represents the rate at which i) spatial correlation decreases as the points move farther in space with the same time index, and ii) temporal correlation decreases as the time points are farther apart in time at the same input vector, respectively. Both the spatial correlation and temporal correlation are modeled using the Gaussian covariance. The parameter Γ^2 can be interpreted as variance of *M (w)* for all *w.* The input parameter design consists of $((w_a, n_i)_{i=1}^{k})$ design points to run independent simulations with replicates applied to each of the design points. Let, $k \times 1$ denote a vector of sample averages of simulation responses given by $\bar{y} = (\bar{y}(w_1), \bar{y}(w_2), \ldots., \bar{y}(w_k))^T$, where in $\bar{y}(w_i)$ is the resulting estimate of performance measure obtained at design point w_i and $\bar{\varepsilon}(w_i)$ is the sampling variability inherent in a stochastic simulation (Ankenman, Nelson, & Staum, 2010). The equations associated with $\bar{y}(w_i)$ and $\bar{\varepsilon}(w_i)$ are described below in equation (3):

$$\bar{y}(w_i) = \frac{1}{n_i}\sum_{j=1}^{n_i} y_j(w_i) = Y(w_i) + \bar{\varepsilon}(w_i) \text{ and } \bar{\varepsilon}(w_i) = \frac{1}{n_i}\sum_{j=1}^{n_i} \varepsilon_j(w_i), i = 1,2,\ldots,k.$$

(3)

Similar as in (Ankenman, Nelson, & Staum, 2010), shown below in equation (4), let $\sum_M$ be the *k x k* covariance matrix across all design points and let $\sum_M(w_o,.)$ be the *k x 1* vector, (Cov[M(w_0,w_1)], Cov[M(w_0,w_2)],..,Cov[M(w_0,w_k)]^T that contains spatial covariance between the *k* design points and a given prediction point w_o. Also, let $\sum_\varepsilon$ be the *k x k* covariance matrix of vector of simulation errors associated with vector of point estimates $\bar{y}$, across all design points. As described in (Ankenman, Nelson et al. 2010), the best linear predictor $Y(w_o)$ that

has the minimum mean squared error (MSE) among all linear predictors at a given point $w_o = (X_o{}^T, t_o)^T$ can be given by equation (4):

$$\hat{Y}(w_o) = \widehat{\beta_o} + \textstyle\sum_M (w_0,\ .)^T\, [\sum_M + \sum_\varepsilon]^{-1} (\bar{y} - 1_k \widehat{\beta_0}), \qquad (4)$$

where, 1_k is the *k x 1* vector of ones and $\widehat{\beta_o}$ is estimated to be 1. The corresponding optimal MSE as in (Ankenman, Nelson et al. 2010) is given by equation (5):

$$MSE\left(\hat{Y}(w_o)\right) = \textstyle\sum_M X_0, w_o - \sum_M \left(w_{0,}.\right)^T [\sum_M + \sum_\varepsilon]^{-1} \sum_M (w_o, .) \qquad (5).$$

In order to implement the metamodeling approach as described above, the unknown model parameters are estimated through maximizing the log-likelihood function. The underlying standard assumption is that $(Y(w_o),\ \bar{y}^T)^T$ follows a multivariate normal distribution, for e.g. see (Ankenman, Nelson et al. 2010) and (Chen and Kim 2014). The function implemented in *mlegp* package in R (Dancik and Dorman 2008) is used for the estimation of the parameters. Once the parameters are estimated the prediction then follows equations (4) and (5).

Sensitivity index calculation

In order to determine the effect of input variables on the output, we employed the variance decomposition method. These methods involve the decomposition of the variance of the output as a sum of the variance produced by each input parameter (Thiele, Kurth et al. 2014).

We independently generated 10,000 x 38 sampling matrices, such that the parameter combinations are generated via Latin Hypercube sampling and as

described in (Saltelli, Annoni et al. 2010). Simulations were performed using the GP spatio-temporal model as described in the previous section and the Sobol' indices were computed as described in (Sobol 1993) (Saltelli, Annoni et al. 2010). The Sobol' method quantitatively measured the contribution of each input parameter by computing the first order and total order index (Saltelli, Annoni et al. 2010). For output *Y,* input parameter matrix X_i where, *i* is the input parameters of the model, the Sobol' indices are computed as follows:

$$SI_1^{Xi} = \frac{V[E(Y|X_i]}{V(Y)} ,$$

and

$$SI_{tot}^{Xi} = \frac{V[E(Y|X_{\sim i}]}{V(Y)}.$$

The Sobol' first order sensitivity index SI_1^{Xi} measures the impact of one single parameter on the model output, whereas the Sobol' total order index measures the influence of X_i including all the interactions with other parameters. The First-order indices were computed using the Sobol-Saltelli's method as described in (Saltelli, Annoni et al. 2010) (Sobol', Tarantola et al. 2007) whereas, the total order indices were computed using Sobol-Jansen as in (Jansen 1999, Saltelli, Annoni et al. 2010).

3. Results

3. 1 Hybrid model simulations produce similar immune system dynamics observed in previously published experimental data

We first aimed to simulate the findings observed in previous gut models (Viladomiu, Bassaganya-Riera et al. 2017) to ensure that we obtained similar response dynamics from the hybrid ENISI model of *H. pylori* infection. As in (Viladomiu, Bassaganya-Riera et al. 2017), to demonstrate that the gastric mucosa harbors a system of macrophages that contribute to the outcome of *H. pylori* infection, we created an *in-silico* Peroxisome proliferator-activated receptor gamma (PPARγ) macrophage specific knockout (KO) model. PPARγ is an important transcription factor that controls the expression of genes that contribute to the inflammatory response once this is initiated. To disrupt the downregulation of pro-inflammatory responses, we simulated a PPARg KO system in either macrophage or T cell populations and compared the response to a wild type system. In the model, we created three different macrophage populations, comprised of, "resident" macrophage agents that mimic the properties of the F4/80hi CD11b+ CD64+ CXCR1+ macrophages reported in (Viladomiu, Bassaganya-Riera et al. 2017), monocyte derived (infiltrating) and macrophage populations with regulatory (M2, or alternatively activated) and pro-inflammatory function (M1 or classically activated) (see *Table 2*).

We simulated an *in-silico H. pylori* infection by creating four groups, i) a control - WT (representing a wild type group), ii) CD4Cre (T cell specific PPARγ KO), iii) LysMCre (Myeloid cell specific PPARγ KO) and clodronate group (simulating the removal of macrophages by chemical depletion via clodronate treatment). To

simulate the CD4Cre group, the probability of a naive T cell transitioning to an iTreg cell (*nTtoiTreg*) and the rate of Th17 cell differentiation to iTreg (*Th17toiTreg*) were reduced (from control). As described in (Carbo, Hontecillas et al. 2013), to simulate the LysMCre experimental conditions, the probabilities of i) a monocyte transitioning to a regulatory macrophage (*MonotoMreg*) and ii) immature dendritic cells switching to tolerogenic dendritic cells(*iDCtotDC*) were reduced. Lastly, the removal of macrophages by clodronate were simulated by decreasing the initial numbers of the macrophage population including the resident macrophages. The rationale to include the clodronate group (macrophage removal) was to evaluate if depletion of phagocytic cells (terminology with respect to model, *i.e.* monocytes, resident, monocyte-derived macrophages and inflammatory macrophages) would affect *H. pylori* colonization levels, as we have previously reported in an *in vivo* model (Viladomiu, Bassaganya-Riera et al. 2017). Further, in order to simulate the myeloid cell PPARγ KO system, the initial population of resident macrophages were also reduced.

All the groups were initialized with equal loads of *H. pylori* agents. Ten replicates of the simulations were performed for each of the input parameter settings specific to each group. The outputs were averaged and standard error of mean were plotted as ribbons (shaded regions) across the graphs. After running the 10 replicates of the time series *in-silico* simulation, the hybrid model showed significantly ($p < 0.05$) higher levels of *H. pylori* in the WT and CD4Cre groups as compared to LysMCre KO and macrophage-depleted groups (*Fig. 2*, panel *a* and *d*).

Fig 2. Time course simulations representing the immune system during Helicobacter pylori infection.

The upper half of the plot shows the dynamics of the population cells over time representing the number of cells (y-axis) versus time (x-axis) in a WT (black), CD4Cre (green), clodronate (red) and LysMCre (blue) simulated in-silico groups during H. pylori infection. The cell populations include - a) H. pylori; b) the resident macrophages and, c) monocyte-derived macrophages in the lamina propria compartment. The figures in the lower half (d-f), show the results for statistical comparison between the groups using ANOVA with the post-hoc analysis. The letters 'a', 'ab' and 'b' represent statistically significant differences (P<0.05) between the groups obtained after running the Tukey's Honestly Significant Difference.

In addition to the increase in *H. pylori,* WT and CD4Cre *in-silico* experimental groups had higher resident as well as monocyte derived regulatory macrophages as compared to clodronate (macrophage depleted) and LysMCre groups (*Fig. 2b-c, e-f*). These observations were qualitatively similar to the findings in (Viladomiu, Bassaganya-Riera et al. 2017), where the stomach of WT mice was enriched in a population of F4/80+CD11b+CD64+ myeloid cells, compared to LysMCre mice.

Overall, with the results in *Fig 2,* we showed the ability of the hybrid model to replicate the experimental results in (Viladomiu, Bassaganya-Riera et al. 2017), and this preliminary data was used as a base calibration setting for *SA* and other *in-silico* findings.

3.2 Partial correlation coefficient analysis screened the influential parameters

To reduce the computational complexity of varying an input parameter space of 38 parameters, we divided the SA process in two stages. For first stage analysis, we utilized the PRCC regression-based *SA* method to screen the influential inputs and used it for the second stage design of the experiments (refer Methods 2.2). Using PRCC, we determined the impact of the input parameters on the output cell populations in the model. The parameters with significant correlation with *H. pylori* in the gastric lamina propria compartment and resident macrophages are shown in *Fig 3*, along with their PRCC values. The bars in blue, highlight the parameters that are significantly different than 0, at $P < 0.05$ compared to grey bars which are not significant. It is important to note that at this stage the analysis using PRCC was non-temporal.

The *SA* from first stage results showed that the epithelial damage due to infectious bacteria (*epiinfbctdam*) with a coefficient value of (~0.2), was positively correlated with the colonization of *H. pylori* in the lamina propria compartment, indicating the important role of epithelial cell damage during the course of infection, similar to our findings obtained in (Alam, Deng et al. 2015). Another parameter included the probability of the release of IL-6 (*IL6)* with a coefficient value within the range (0.3-0.4).

Next, the epithelial cell damage parameters (*epiinftbctdam* = (0.2-0.3), *epiTh17dam* = 0-0.2) were shown to have positive influence on the resident macrophage cells whereas, the T cell type transition parameters (*iTregtoTh17* = (0.3 - 0.4) and *Th17toiTreg* = (0.1 - 0.2)) showed a negative impact on the

resident macrophages. Similarly, we performed the PRCC analysis for all the cell populations under consideration during the infection (not shown).

Fig 3. Bar plots for the partial rank correlation coefficients.

The magnitude of the bar-plot indicates the value of the partial rank correlation coefficient. The blue bar indicated the input parameters shown to be significantly different than 0, at P <0.05 as influential whereas the grey bars indicate the non-influential parameters on a) H. pylori and b) resident macrophages, in the lamina propria compartment.

The significant parameters (marked in blue bars) obtained from the SA of the output from first stage design of experiments (152 parameter settings with 20 replicates, refer Methods 2.2), were selected to be varied for the second stage design. All the selected inputs are shown in Additional *Fig S3*. In all, we obtained 23 active inputs from the first stage.

3.3 Metamodel based spatio-temporal sensitivity analysis

The outputs obtained after running the first (152 x 20 runs) and second (115 x 20 runs) stage simulations, wherein x20 denotes the 20 replicates, were combined to be used as a 'training dataset'. The combined output was utilized to build a Gaussian process based spatiotemporal metamodel (refer Methods 2.2), using *mlegp* package in R (Dancik and Dorman 2008).

The outputs from the 'training dataset' were sub-divided into 6 datasets, corresponding to six time periods (Days 1-14, 15-21, 22-30, 31-42, 43-90, 91-

201) and averaged across these periods. The sub-division of output across the time periods, aided the temporal analysis over the initiation (Day 1-14), peak of infection (Days 15-30) and chronic phase (post Day 31) stages as in (Viladomiu, Bassaganya-Riera et al. 2017). We then fit a Gaussian process model (with nugget) and evaluated the performance of the fitting of the metamodel for *H. pylori,* resident macrophages and monocyte derived macrophages in lamina propria compartment, and tolerogenic DC in the gastric lymph node, using the diagnostic plots (see figures in Additional file, Fig *S4*). After fitting the models, we performed variance based global *SA* by computing the Sobol' total order and first order sensitivity index (refer Methods 2.2). The estimates of the Sobol' total order indices for the input parameters calculated over the six time periods are shown in *Fig 4* (a-d).

Fig 4*. *Heat-maps of Sobol' total order index for the input parameters across different output populations.

The values in the heat-map indicate the Sobol' total order sensitivity index obtained from the metamodel, for the 38 input parameters with respect to the cell populations. The values with darker color indicate a stronger influence on the cell population as compared to the ones with lighter shade that indicate non-influential parameters for the cell populations - a) H. pylori, b) monocyte derived macrophages, c) resident macrophages, in the lamina propria compartment and d) tolerogenic DCs, in the gastric lymph node compartment. The indexes are calculated over six time points ranging across the three stages of infection, including initiation (Day 1-14), peak (Days 15-42) and recovery stages (Days 43-201).

As shown in *Fig* 4a, the metamodel based global SA showed that the input parameters, epithelial cell proliferation (*Epiprolifer*) and epithelial cell death (*Epicelldeath*) had the strongest impact on the population of *H. pylori* in lamina propria compartment. As time progressed from initiation of the infection (Days 1-14), through peak (Days 15-30), the epithelial cell proliferation had a continued impact on the colonization of *H. pylori*. Next, the influence of the probability of epithelial cell death decreased over the course of infection. Further, *Fig 4b* highlighted the impact of epithelial cell proliferation (*Epiprolifer*) and epithelial cell death (*Epicelldeath*) on the monocyte-derived macrophages.

For the resident macrophage population in the lamina propria, that have emergent properties similar to the one characterized in (Viladomiu, Bassaganya-Riera et al. 2017), we observed that the resident macrophage replication parameter (*ResmMacRep*) has an impact during the initiation and peak stages of the infection which indicates that these subsets of macrophages replicate during the course of *H. pylori* infection. This highlights the reliability of the two-staged global SA method used here, as these findings are consistent with the ones in (Viladomiu, Bassaganya-Riera et al. 2017) wherein we observed that these subsets of macrophages are recruited to the stomach lamina propria during the course of *H. pylori* infection.

Finally, for the tolerogenic DCs in *Fig 4d*, we observed that the epithelial cell death (*Epicelldeath*) seemed to have an impact. Another parameter that stands for the probability of naive T cell transitioning to iTreg cell (*nTtoiTreg*) was shown to have an impact on the tolerogenic dendritic cells. Tolerogenic dendritic cells are involved in the rule that transitions the naive T cells to iTreg cells in the gastric lymph node, and the stronger impact of the *nTtoiTreg* during the initiation and peak stages

of the infection highlights the role of the tolerogenic dendritic cells during the course of infection.

The global *SA* data suggested that the main contributors of the chronic colonization of *H. pylori* in the lamina propria are the epithelial cells, specifically the epithelial cell proliferation parameter.

3.4 Effect of different ranges of epithelial cell proliferation

Based on the results obtained from the model prediction (the epithelial cell proliferation parameter shown to have a strong impact on the *H. pylori* population), derived from the metamodel based global *SA;* we hypothesized that the epithelial cell proliferation is responsible for the higher colonization of *H. pylori*. To validate the hypothesis, we studied the effect of different ranges of epithelial cell proliferation on the model outputs, *in-silico*. To study this effect, we altered the parameter values governing epithelial cell proliferation (*Epiprolifer*) across different ranges (0.1-0.9, with 0.6 being the value for control conditions) and run the simulations using the hybrid model. Interestingly, upon decreasing the *Epiprolifer* from a range of values 0.9-0.1, we observed a decreasing effect (*Fig 5a-d)* on *H. pylori,* monocyte-derived macrophages, resident macrophages in the lamina propria compartment and tolerogenic dendritic cells in gastric lymph node.

***Fig 5. In-silico* study of the effect of epithelial cell proliferation parameter on the cell populations.**

The plots show the effect of varying epithelial cell proliferation (Epiprolifer) parameter (with values 0.1, 0.5, 0.6(WT), and 0.9) on the output cell population of a) H. pylori, b) tolerogenic dendritic cells, c) resident macrophages and d) monocyte derived

macrophages. The parameter has a decreasing effect on the cellular populations under consideration, wherein a decrease in the parameter value, decreases the abundance of the cells over time. *The lower half of the figures (a-d), show the results for statistical comparison between the groups using ANOVA with the post-hoc analysis. The letters 'a', 'b', 'c', and 'bc' represent statistically significant differences (P<0.05) between the groups obtained after running the Tukey's Honestly Significant Difference*.

The *in-silico* findings suggested the involvement of regulatory macrophages (both resident as well as monocyte derived) and tolerogenic DC on the colonization *of H. pylori* in the gastric lamina propria. This highlighted and validated the role of epithelial cell proliferation as one of the main factor affecting *H. pylori* levels in the gastric niche.

4. Discussion

H. pylori eludes the immune response and is able to persist in the gastric mucosa and exhibit both pathogenic and commensal roles. However, the immunological mechanisms underlying its ability to persist in a harsh acidic gastric environment and its dual role as pathogen and beneficial organism remain unknown. A subset of macrophages helps create a regulatory microenvironment that promotes the chronic colonization of *H. pylori* (Viladomiu, Bassaganya-Riera et al. 2017). However, the immune regulatory mechanisms are incompletely understood. Computational models of the immune system featuring immune responses are powerful tools for testing the different 'what-if' scenarios. Multiscale models of the immune response

are attractive in terms of modeling the responses at different spatiotemporal scales (Heiner and Gilbert 2013).

In this study, we developed a HPC-driven hybrid, high-resolution, multiscale model to simulate the complex immunoregulatory mechanisms during *H. pylori* infection. The hybrid model was integrated with two intracellular ODEs capturing the dynamics of CD4+ T cells and regulatory macrophages. The inputs to the hybrid model are the set of parameters whose variation governs the immune systems dynamics during infection. The obtained outputs were emergent patterns of different cell types, cytokines and bacterial levels for *e.g.* the levels of *H. pylori*, and that qualitatively matched the patterns observed in an *in vivo* infection model (Carbo, Bassaganya-Riera et al. 2013, Viladomiu, Bassaganya-Riera et al. 2017). We presented an *in-silico* framework that evaluated the global *SA* of the hybrid model and studied how the variation in the biological parameters affected the simulation outputs. The two-staged global SA indicated that epithelial cell parameters, specifically, the proliferation of epithelial cells affected the colonization of *H. pylori* in the gastric mucosa. These results were validated *in-silico*, and highlighted the involvement of regulatory macrophages and tolerogenic DC in facilitating *H. pylori* colonization of the gastric mucosa. Previous studies highlighted *H. pylori* inhabits the apical surfaces of the epithelial cells and maintains a persistent infection (Alzahrani, Lina et al. 2014). Further, Mimuro et al. demonstrated that *H. pylori* promotes epithelial gastric cell survival by attenuating apoptosis. These events showed how *H. pylori* regulated the gastric niche and utilized epithelial cells to facilitate its persistence within the stomach (Mimuro, Suzuki et al. 2007) (Wroblewski and Peek 2007).Thus, the findings in the current study are in line with the literature that suggest epithelial cell proliferation favor the colonization of *H. pylori* in the stomach. Our group also showed another mechanism used by *H. pylori* to create a gut

microenvironment that involved the induction of IL-10-driven regulatory mechanism mediated by $CD11b^{+}F4/80^{hi}CD64^{+}CX_{3}CR1^{+}$ mononuclear phagocytes, which facilitated its colonization (Viladomiu, Bassaganya-Riera et al. 2017). Additionally, in this paper, we reported that regulatory macrophages were involved in the process of colonization of *H. pylori* when we varied the epithelial cell proliferation parameter *in-silico*. Zhang et al., demonstrated that *H. pylori* directed active tolerogenic programming of DCs that favored chronic bacterial colonization, by altering the balance of Th17/Treg cells (Zhang, Liu et al. 2010). Rizzuti, Ang et al., demonstrated *H. pylori* mediated IL-10 release caused the activation of signal transducer and activator of transcription 3 (STAT3) in DC. This activation of STAT3 via IL-10 release was shown to induce the production of tolerogenic DC phenotype (Rizzuti, Ang et al. 2015). The findings from this paper also indicated the involvement of tolerogenic DCs in affecting the mucosal levels of *H. pylori*. Therefore, the literature combined with the results from this study, collectively suggest that during *H. pylori* infection, the epithelial cell favors the colonization of *H. pylori* by creating a regulatory microenvironment. This process is mediated by the regulatory macrophages and tolerogenic programming of DC. Based on the results from this paper and findings from the literature, this leads us to propose that the induction of IL-10 by the regulatory macrophages is potentially involved in directing the tolerogenic programming of DC.

At its current stage, the hybrid ENISI model reproduces the overall immune system dynamics observed during a *H. pylori* infection. The parameters of calibrated ODEs were kept unchanged, whereas the ABM parameters were calibrated by qualitatively matching the patterns of the output simulations as observed in an *in vivo* model of *H. pylori* infection (Viladomiu, Bassaganya-Riera et al. 2017). For ABM, its calibration and validation remain the major key issues, discussed elsewhere (Ten

Broeke, Van Voorn et al. 2016) (Windrum, Fagiolo et al. 2007) (Fagiolo, Moneta et al. 2007). Further, developing targeted methods of *SA* have been identified as a key challenge in the field (Crooks, Castle et al. 2008, Filatova, Verburg et al. 2013, Ten Broeke, Van Voorn et al. 2016). In this paper, we highlighted the use of *SA* methods with a two-stage global *SA* framework comprised of first, screening the input parameters (using PRCC) and second, building of a surrogate model (using GP) of the hybrid model to understand the emergent behavior of the represented system. It is important to note that each *SA* method known, has its own merits and produces useful information however none provide a complete picture of the emergent model behavior (Ten Broeke, Van Voorn et al. 2016). First, we employed PRCC methods as the initial step in our two staged *SA* that aided the screening of active inputs and reduced the parameter space. The choice of PRCC was advantageous and justified by the low computational cost and low complexity in the computation of the coefficients. Another advantage of the regression-based PRCC method is that the complex output from our hybrid model was condensed into descriptive relationship that can be described by statistical measures such as R^2 (Ten Broeke, Van Voorn et al. 2016). As described in (Ten Broeke, Van Voorn et al. 2016) the results from PRCC are good descriptors of the outputs produced if the regression function constitutes a good fit to the output (Ten Broeke, Van Voorn et al. 2016). However, if the function does not yield a good fit, the regression based *SA* are proven to be useful in screening the influential parameters for further analysis (Ten Broeke, Van Voorn et al. 2016), as described in our analysis. Further, the interaction effects between the parameters are not considered in regression based methods and hence it was followed by the use of variance based methods in later stage analysis. Second, we employed metamodeling-based approach and Sobol' method as they provided information on the interaction between input variable and the use of

metamodels allowed to compute the sensitivity indices. One of the advantages of the Sobol' method is that the it is model-free and no fitting functions are used to decompose the output variance (Saltelli, Ratto et al. 2008). It considers the averaged effect of parameters over the whole parameter space but fails to explore the different patterns within the space (Ten Broeke, Van Voorn et al. 2016). Further, the method is not suitable for quantification of output variability if the output distributions deviate from normal distribution (Ten Broeke, Van Voorn et al. 2016). The detailed comparison of different *SA* methods used for the global *SA* of ABMs are described in detail in (Ten Broeke, Van Voorn et al. 2016). Thus, we performed both PRCC and computation of Sobol' indices approaches to evaluate the influence of the input parameter variation and identified the parameters involved in the successful colonization of the gastric niche by *H.* pylori.

Some limitations of the model include implementation through a two-dimensional grid system and including all cells of the same size. Although, we parallelize the computation of the hybrid model output, the large number of simulations required for the global *SA* compensates for the benefits of parallelization. To improve the calibration process and overall usability of the model, the data required for model calibration would include tissue biopsies from people infected with *H. pylori* that can be used to quantify the cells and take into account their spatial arrangement. The current version is also limited in terms of the interactions based on epithelial cells and DC as they are strictly rule based. The building of ODE models for these cells and integrating them with the ABM model will help capture the dynamics of epithelial cells and DC more in depth. Overall the immunoregulatory mechanisms underlying the chronic colonization of *H. pylori* and the predictive capacity of the model can be further improved by incorporating cell specific models for epithelial cells and DC.

In summary, a high-resolution, hybrid, multiscale spatiotemporal stochastic model of *H. pylori* infection was built and global *SA* was performed. The results from the global *SA* highlight the key role played by epithelial cells in affecting the levels of *H. pylori* colonization. The *in-silico* validation of varying the epithelial cell proliferation parameter, demonstrated the involvement of regulatory macrophages and the tolerogenic DC. The next steps aimed to enrich the model will involve the validation of the findings *in vivo* in order to study the underlying mechanisms involved in the successful immune evasion by *H. pylori*.

5. Potential Implications

The computational model of the gut contains high-resolution information processing representations of immune responses that are generalizable for other infectious and autoimmune diseases. Complex diseases such as autoimmune disorders, infectious diseases and cancer all require integration of the multiscale level data, information and knowledge, ranging from genes, proteins, cells, tissue to organ level. The ENISI model of the gut presented here can be generalized to other diseases by implementing the agents and rules specific to that disease, plus recalibrating the model based on data specific to the new indication. Since ABMs have modular architectures, addition of new agent-types and modification of rules can be done without restructuring the entire simulation setup (An, Mi et al. 2009). The use of ABM in such hybrid models not only facilitates the implementation of already known mechanisms but also helps validate and predict any unforeseen new mechanisms using data analytics methods such as global *SA*. The finer details regarding intracellular and intercellular interactions that contribute towards the nonlinear and complex behavior of the gut can also be studied by integrating the intracellular ODE models as implemented here.

Tables

Name of Agent	Agent Type	Rules
Helicobacter pylori	*H. pylori*	- Moves across the epithelial cell border if near damaged epithelial layer<br>- Proliferates in the lumen and lamina propria<br>- Dies (removed from the simulation) in lamina propria and in the lumen due to the damage of epithelial cells by Th1 or Th17 cells
Macrophages	Monocyte	- Proliferates in presence of effector dendritic cells or damaged epithelial cells<br>- Proliferates in the lamina propria<br>- Differentiates to regulatory macrophage in based on the output from the Macrophage ODE<br>- Differentiates to inflammatory macrophages in presence of IFN-γ<br>- Dies naturally (removed from the model)
	Resident	- Proliferates in presence of *H. pylori*<br>- Secretes IL10<br>- Dies naturally<br>- Dies due to Th1 and Tr cells
	Regulatory	- Proliferates and removes bacteria<br>- Dies<br>- Secretes IL10
	Inflammatory	-Proliferates in presence of damaged epithelial cell<br>-Dies naturally
Dendritics	Immature	-Moves from lamina propria to epithelium compartment and from the epithelium to the lamina propria<br>- Differentiates to tolerogenic dendritic cell in presence of tolerogenic bacteria, both in epithelium and lamina propria<br>- Differentiates to effector dendritic cell in presence of *H. pylori*<br>- Proliferates in lamina propria and gastric lymph node<br>- Dies naturally
	Effector	- Moves from lamina propria to gastric lymph node<br>- Moves form epithelium to lamina propria<br>- Secretes IL6 and IL12<br>- Dies naturally
	Tolerogenic	- Moves from lamina propria to gastric lymph node<br>- Moves from epithelium to lamina propria<br>- Secretes TGF-β<br>- Dies naturally
T cells	Naïve	In presence of effector dendritic cells:<br>- Differentiates to Th1 in presence of IFN-γ or IL12<br>- Differentiates to Th17 in presences of IL6 or TGF-β<br>In presence of tolerogenic dendritic cells:<br>- Differentiates to iTreg in presence of TGF-β

		- Differentiates to Tr in presences of IL10<br>- Dies naturally
	Th1	- Secretes IFN-γ<br>- Moves from gastric lymph node to lamina propria<br>- Proliferates in lamina propria and gastric lymph node<br>- Dies naturally
	Th17	- Secretes IL17<br>- In presence of tolerogenic dendritic cell, transition to iTreg cells<br>- Moves from gastric lymph node to lamina propria<br>- Proliferates in lamina propria and gastric lymph node<br>- Dies naturally
	iTreg	- Secretes IL10<br>- In presence of tolerogenic dendritic cell, transition to iTreg cells<br>- Moves from gastric lymph node to lamina propria<br>- Proliferates in lamina propria and gastric lymph node<br>- Dies naturally
	Tr	- Secretes IL10<br>- Dies naturally<br>- Proliferates in the lamina propria
Epithelial	Healthy	-Damaged due to infectious bacteria<br>-Damaged due to Th1 and Th17 cells<br>-Proliferates<br>-Secretes IL6 and IL12<br>-Dies naturally
	Damaged	-Healed to heathy state in presence of IL10<br>-Dies naturally
Bacteria	Infectious	- Dies due to Th1 or Th17 or inflammatory macrophages or damaged epithelial cells<br>- Dies naturally<br>- Proliferates in the lamina propria
	Tolerogenic	- Moves from lumen to the epithelium in presence of damaged epithelial cells<br>- Becomes infectious if moves in the lamina propria compartment<br>- Proliferates in lumen and lamina propria<br>- Dies naturally

Table 1. A list of rules for all the agent types implemented in the hybrid model

Additional Files

Fig S1

Table S1

Fig S2

Fig S3

Fig S4

Fig S1. Design implementation of the hybrid multiscale model used to simulate Helicobacter pylori infection

The figure shows the class structure used in the ENISI MSM hybrid agent based-ODE model. Each group consists of an act() function that includes the implemented rule for each agent. The previously published ODE models for T cells and Macrophage are used to integrate in the ABM code.

Table S1 *Table describing the input parameters used in the sensitivity analysis and their ranges used.*

Fig S2. Flowchart for the two-staged global sensitivity analysis.

Fig S3. The active and inactive inputs selected from the stage 1 analysis

The rows represent the input parameters and columns represent the output cell populations. The green boxes highlight the 'active' input parameters (row) that are shown to be have a significant influence (calculated based on the results obtained from partial correlation coefficient analysis), on an output cell (columns) under consideration.

Fig S4. Diagnostic and residual plots obtained for the Gaussian processes fitted metamodels

The upper panel represents the diagnostic Q-Q plots where the open circles represent the cross validated predictions; solid black lines represent observed response. The lower panel represents the residual plots for the cell populations –(a) Helicobacter pylori; (b) Resident macrophages; (c) Monocyte-derived macrophages in the Lamina propria and (d) Tolerogenic dendritic cells in the Gastric lymph node compartment.

Availability of source code and requirements

- Project Name: ENISI MSM
- Project home page: https://github.com/NIMML/ENISI-MSM
- Programming language: C++, R

Availability of supporting data and materials

The data sets and files supporting the results of this article are available in the ENISI-MSM GitHub repository, https://github.com/NIMML/ENISI-MSM.

Declarations

List of abbreviations

ABM – Agent based model

DC – Dendritic cells

ENISI MSM – Enteric Immunity Simulator Multi-scale Modeling

GLN – gastric lymph node

GP - Gaussian process

H. pylori – *Helicobacter pylori*

HPC – High performance computing

LP – Lamina propria

ODE – Ordinary Differential Equation

PDE – Partial Differential Equation

SA – Sensitivity analysis

PRCC - Partial rank correlation coefficient

Consent for publication

Not applicable.

Competing interests

The author(s) declare that they have no competing interests.

Authors' contributions

MV formulated the model, implemented, performed the simulations, analyzed model-generate outputs, made the figures and wrote the manuscript. MV, AL and SH formulated the model. SH implemented the code architecture and benchmarked the parallel version of the hybrid model. XC and MV wrote the codes for global sensitivity analysis and generated the design matrices. JBR, VA, and RH supervised the project. JBR and RH edited the manuscript. JBR, AL, NTJ, SH, VA, XC and RH participated in discussions on the model and results. All authors provided critical feedback on the project.

Acknowledgements

Not applicable.

References

Abedi, V., R. Hontecillas, S. Hoops, N. Liles, A. Carbo, P. Lu, C. Philipson and J. ABassaganya-Riera (2015). ENISI multiscale modeling of mucosal immune responses driven by high performance computing. 2015 IEEE International Conference on Bioinformatics and Biomedicine (BIBM).

Alam, M., X. Deng, C. Philipson, J. Bassaganya-Riera, K. Bisset, A. Carbo, S. Eubank, R. Hontecillas, S. Hoops, Y. Mei, V. Abedi and M. Marathe (2015). "Sensitivity Analysis of an ENteric Immunity SImulator (ENISI)-Based Model of Immune Responses to Helicobacter pylori Infection." PLoS One **10**(9): e0136139.

Alzahrani, S., T. T. Lina, J. Gonzalez, I. V. Pinchuk, E. J. Beswick and V. E. Reyes (2014). "Effect of Helicobacter pylori on gastric epithelial cells." World Journal of Gastroenterology : WJG **20**(36): 12767-12780.

An, G., Q. Mi, J. Dutta-Moscato and Y. Vodovotz (2009). "Agent-based models in translational systems biology." Wiley Interdiscip Rev Syst Biol Med **1**(2): 159-171.

Ankenman, B., B. L. Nelson and J. Staum (2010). "Stochastic kriging for simulation metamodeling." Operations research **58**(2): 371-382.

Asghar, R. J. and J. Parsonnet (2001). "Helicobacter pylori and risk for gastric adenocarcinoma." Semin Gastrointest Dis **12**(3): 203-208.

Bassaganya-Riera, J. (2015). Computational Immunology: Models and Tools, Academic Press.

Bassaganya-Riera, J., M. G. Dominguez-Bello, B. Kronsteiner, A. Carbo, P. Lu, M. Viladomiu, M. Pedragosa, X. Zhang, B. W. Sobral, S. P. Mane, S. K. Mohapatra, W. T. Horne, A. J. Guri, M. Groeschl, G. Lopez-Velasco and R. Hontecillas (2012). "Helicobacter pylori colonization ameliorates glucose homeostasis in mice through a PPAR γ-dependent mechanism." PLoS One **7**(11): e50069.

Blaser, M. J. (1992). "Hypotheses on the pathogenesis and natural history of Helicobacter pylori-induced inflammation." Gastroenterology **102**(2): 720-727.

Cappuccio, A., P. Tieri and F. Castiglione (2016). "Multiscale modelling in immunology: a review." Brief Bioinform **17**(3): 408-418.

Carbo, A., J. Bassaganya-Riera, M. Pedragosa, M. Viladomiu, M. Marathe, S. Eubank, K. Wendelsdorf, K. Bisset, S. Hoops, X. Deng, M. Alam, B. Kronsteiner, Y. Mei and R. Hontecillas (2013). "Predictive computational modeling of the mucosal immune responses during Helicobacter pylori infection." PLoS One **8**(9): e73365.

Carbo, A., R. Hontecillas, B. Kronsteiner, M. Viladomiu, M. Pedragosa, P. Lu, C. W. Philipson, S. Hoops, M. Marathe, S. Eubank, K. Bisset, K. Wendelsdorf, A. Jarrah, Y. Mei and J. Bassaganya-Riera (2013). "Systems modeling of molecular mechanisms controlling cytokine-driven CD4+ T cell differentiation and phenotype plasticity." PLoS Comput Biol **9**(4): e1003027.

Chen, X. and K.-K. Kim (2014). "Stochastic kriging with biased sample estimates." ACM Trans. Model. Comput. Simul. **24**(2): 1-23.

Collier, N. and M. North (2011). "Repast HPC: A platform for large-scale agentbased modeling." Large-Scale Computing Techniques for Complex System Simulations: 81-110.

Cover, T. L. and M. J. Blaser (2009). "Helicobacter pylori in health and disease." Gastroenterology **136**(6): 1863-1873.

Crooks, A., C. Castle and M. Batty (2008). "Key challenges in agent-based modelling for geo-spatial simulation." Computers, Environment and Urban Systems **32**(6): 417-430.

Dancik, G. M. and K. S. Dorman (2008). "mlegp: statistical analysis for computer models of biological systems using R." Bioinformatics **24**(17): 1966-1967.

Fagiolo, G., A. Moneta and P. Windrum (2007). "A critical guide to empirical validation of agent-based models in economics: Methodologies, procedures, and open problems." Computational Economics **30**(3): 195-226.

Filatova, T., P. H. Verburg, D. C. Parker and C. A. Stannard (2013). "Spatial agent-based models for socio-ecological systems: Challenges and prospects." Environmental modelling & software **45**: 1-7.

Gong, C., O. Milberg, B. Wang, P. Vicini, R. Narwal, L. Roskos and A. S. Popel (2017). "A computational multiscale agent-based model for simulating spatio-temporal tumour immune response to PD1 and PDL1 inhibition." J R Soc Interface **14**(134).

Heiner, M. and D. Gilbert (2013). "BioModel engineering for multiscale Systems Biology." Progress in Biophysics and Molecular Biology **111**(2): 119-128.

Hoops, S., S. Sahle, R. Gauges, C. Lee, J. Pahle, N. Simus, M. Singhal, L. Xu, P. Mendes and U. Kummer (2006). "COPASI—a complex pathway simulator." Bioinformatics **22**(24): 3067-3074.

Jansen, M. J. (1999). "Analysis of variance designs for model output." Computer Physics Communications **117**(1-2): 35-43.

Kusters, J. G., A. H. van Vliet and E. J. Kuipers (2006). "Pathogenesis of Helicobacter pylori infection." Clin Microbiol Rev **19**(3): 449-490.

Lamoureux, B., N. Mechbal and J.-R. Massé (2014). "A combined sensitivity analysis and kriging surrogate modeling for early validation of health indicators." Reliability Engineering & System Safety **130**: 12-26.

Leber, A., J. Bassaganya-Riera, N. Tubau-Juni, V. Zoccoli-Rodriguez, M. Viladomiu, V. Abedi, P. Lu and R. Hontecillas (2016). "Modeling the Role of Lanthionine Synthetase C-Like 2 (LANCL2) in the Modulation of Immune Responses to Helicobacter pylori Infection." PLoS One **11**(12): e0167440.

Leber, A., M. Viladomiu, R. Hontecillas, V. Abedi, C. Philipson, S. Hoops, B. Howard and J. Bassaganya-Riera (2015). "Systems Modeling of Interactions between Mucosal Immunity and the Gut Microbiome during Clostridium difficile Infection." PLoS One **10**(7): e0134849.

Ligmann-Zielinska, A., D. B. Kramer, K. Spence Cheruvelil and P. A. Soranno (2014). "Using uncertainty and sensitivity analyses in socioecological agent-based models to improve their analytical performance and policy relevance." PLoS One **9**(10): e109779.

Mane, S., M. Dominguez-Bello, M. Blaser, B. Sobral, R. Hontecillas, J. Skoneczka, S. Mohapatra, O. Crasta, C. Evans and T. Modise (2010). "Host-interactive genes in Amerindian Helicobacter pylori diverge from their Old World homologs and mediate inflammatory responses." Journal of bacteriology **192**(12): 3078-3092.

Marino, S., M. El-Kebir and D. Kirschner (2011). "A hybrid multi-compartment model of granuloma formation and T cell priming in tuberculosis." J Theor Biol **280**(1): 50-62.

Marino, S., I. B. Hogue, C. J. Ray and D. E. Kirschner (2008). "A methodology for performing global uncertainty and sensitivity analysis in systems biology." J Theor Biol **254**(1): 178-196.

Mei, Y., V. Abedi, A. Carbo, X. Zhang, P. Lu, C. Philipson, R. Hontecillas, S. Hoops, N. Liles and J. Bassaganya-Riera (2015). "Multiscale modeling of mucosal immune responses." BMC Bioinformatics **16 Suppl 12**: S2.

Mimuro, H., T. Suzuki, S. Nagai, G. Rieder, M. Suzuki, T. Nagai, Y. Fujita, K. Nagamatsu, N. Ishijima, S. Koyasu, R. Haas and C. Sasakawa (2007). "Helicobacter pylori dampens gut epithelial self-renewal by inhibiting apoptosis, a bacterial strategy to enhance colonization of the stomach." Cell Host Microbe **2**(4): 250-263.

Moon, H., A. M. Dean and T. J. Santner (2012). "Two-stage sensitivity-based group screening in computer experiments." Technometrics **54**(4): 376-387.

Moss, S. and J. Calam (1992). "Helicobacter pylori and peptic ulcers: the present position." Gut **33**(3): 289-292.

Oertli, M., M. Sundquist, I. Hitzler, D. B. Engler, I. C. Arnold, S. Reuter, J. Maxeiner, M. Hansson, C. Taube, M. Quiding-Jarbrink and A. Muller (2012). "DC-derived IL-18 drives Treg differentiation, murine Helicobacter pylori-specific immune tolerance, and asthma protection." J Clin Invest **122**(3): 1082-1096.

Qomlaqi, M., F. Bahrami, M. Ajami and J. Hajati (2017). "An extended mathematical model of tumor growth and its interaction with the immune system, to be used for developing an optimized immunotherapy treatment protocol." Math Biosci **292**: 1-9.

Rasmussen, C. E. and C. K. Williams (2006). "Gaussian processes for machine learning. 2006." The MIT Press, Cambridge, MA, USA **38**: 715-719.

Rizzuti, D., M. Ang, C. Sokollik, T. Wu, M. Abdullah, L. Greenfield, R. Fattouh, C. Reardon, M. Tang, J. Diao, C. Schindler, M. Cattral and N. L. Jones (2015). "Helicobacter pylori inhibits

dendritic cell maturation via interleukin-10-mediated activation of the signal transducer and activator of transcription 3 pathway." J Innate Immun **7**(2): 199-211.

Saltelli, A., P. Annoni, I. Azzini, F. Campolongo, M. Ratto and S. Tarantola (2010). "Variance based sensitivity analysis of model output. Design and estimator for the total sensitivity index." Computer Physics Communications **181**(2): 259-270.

Saltelli, A., M. Ratto, T. Andres, F. Campolongo, J. Cariboni, D. Gatelli, M. Saisana and S. Tarantola (2008). Global sensitivity analysis: the primer, John Wiley & Sons.

Saltelli, A., S. Tarantola and F. Campolongo (2000). "Sensitivity anaysis as an ingredient of modeling." Statistical Science **15**(4): 377-395.

Santner, T. J., B. J. Williams and W. I. Notz (2013). The design and analysis of computer experiments, Springer Science & Business Media.

Sobol, I. M. (1993). "Sensitivity estimates for nonlinear mathematical models." Mathematical modelling and computational experiments **1**(4): 407-414.

Sobol', I. M., S. Tarantola, D. Gatelli, S. S. Kucherenko and W. Mauntz (2007). "Estimating the approximation error when fixing unessential factors in global sensitivity analysis." Reliability Engineering & System Safety **92**(7): 957-960.

Solovyev, A., Q. Mi, Y. T. Tzen, D. Brienza and Y. Vodovotz (2013). "Hybrid equation/agent-based model of ischemia-induced hyperemia and pressure ulcer formation predicts greater propensity to ulcerate in subjects with spinal cord injury." PLoS Comput Biol **9**(5): e1003070.

Ten Broeke, G., G. Van Voorn and A. Ligtenberg (2016). "Which sensitivity analysis method should I use for my agent-based model?" Journal of Artificial Societies and Social Simulation **19**(1): 5.

Thiele, J. C., W. Kurth and V. Grimm (2014). "Facilitating parameter estimation and sensitivity analysis of agent-based models: A cookbook using NetLogo and R." Journal of Artificial Societies and Social Simulation **17**(3): 11.

Verma, M., S. Erwin, V. Abedi, R. Hontecillas, S. Hoops, A. Leber, J. Bassaganya-Riera and S. M. Ciupe (2017). "Modeling the Mechanisms by Which HIV-Associated Immunosuppression Influences HPV Persistence at the Oral Mucosa." PLoS One **12**(1): e0168133.

Viladomiu, M., J. Bassaganya-Riera, N. Tubau-Juni, B. Kronsteiner, A. Leber, C. W. Philipson, V. Zoccoli-Rodriguez and R. Hontecillas (2017). "Cooperation of Gastric Mononuclear Phagocytes with Helicobacter pylori during Colonization." J Immunol **198**(8): 3195-3204.

Vodovotz, Y., A. Xia, E. L. Read, J. Bassaganya-Riera, D. A. Hafler, E. Sontag, J. Wang, J. S. Tsang, J. D. Day, S. Kleinstein, A. J. Butte, M. C. Altman, R. Hammond and S. C. Sealfon (2017). "Solving Immunology?" Trends in immunology **38**(2): 116-127.

Wang, Z., C. M. Birch, J. Sagotsky and T. S. Deisboeck (2009). "Cross-scale, cross-pathway evaluation using an agent-based non-small cell lung cancer model." Bioinformatics **25**(18): 2389-2396.

Windrum, P., G. Fagiolo and A. Moneta (2007). "Empirical validation of agent-based models: Alternatives and prospects." Journal of Artificial Societies and Social Simulation **10**(2): 8.

Wroblewski, L. E. and R. M. Peek (2007). "Orchestration of Dysregulated Epithelial Turnover by a Manipulative Pathogen." Cell Host & Microbe **2**(4): 209-211.

Zhang, M., M. Liu, J. Luther and J. Y. Kao (2010). "Helicobacter pylori directs tolerogenic programming of dendritic cells." Gut Microbes **1**(5): 325-329.

Name of Agent	Agent Type	Rules
Helicobacter pylori	*H. pylori*	- Moves across the epithelial cell border if near damaged epithelial layer<br>- Proliferates in the lumen and lamina propria<br>- Dies (removed from the simulation) in lamina propria and in the lumen due to the damage of epithelial cells by Th1 or Th17 cells
Macrophages	Monocyte	- Proliferates in presence of effector dendritic cells or damaged epithelial cells<br>- Proliferates in the lamina propria<br>- Differentiates to regulatory macrophage in based on the output from the Macrophage ODE<br>- Differentiates to inflammatory macrophages in presence of IFNg<br>- Dies naturally (removed from the model)
	Resident	- Proliferates in presence of *H. pylori*<br>- Secretes IL10<br>- Dies naturally<br>- Dies due to Th1 and Tr cells
	Regulatory	- Proliferates and removes bacteria<br>- Dies<br>- Secretes IL10
	Inflammatory	-Proliferates in presence of damaged epithelial cell<br>-Dies naturally
Dendritics	Immature	-Moves from lamina propria to epithelium compartment and from the epithelium to the lamina propria<br>- Differentiates to tolerogenic dendritic cell in presence of tolerogenic bacteria, both in epithelium and lamina propria<br>- Differentiates to effector dendritic cell in presence of *H. pylori*<br>- Proliferates in lamina propria and gastric lymph node<br>- Dies naturally
	Effector	- Moves from lamina propria to gastric lymph node<br>- Moves form epithelium to lamina propria<br>- Secretes IL6 and IL12<br>- Dies naturally
	Tolerogenic	- Moves from lamina propria to gastric lymph node<br>- Moves from epithelium to lamina propria<br>- Secretes TGF-β<br>- Dies naturally
T cells	Naïve	In presence of effector dendritic cells:<br>- Differentiates to Th1 in presence of IFN-γ or IL12<br>- Differentiates to Th17 in presences of IL6 or TGF-β<br>In presence of tolerogenic dendritic cells:<br>- Differentiates to iTreg in presence of TGF-β

		- Differentiates to Tr in presences of IL10<br>- Dies naturally
	Th1	- Secretes IFN-γ<br>- Moves from gastric lymph node to lamina propria<br>- Proliferates in lamina propria and gastric lymph node<br>- Dies naturally
	Th17	- Secretes IL17<br>- In presence of tolerogenic dendritic cell, transition to iTreg cells<br>- Moves from gastric lymph node to lamina propria<br>- Proliferates in lamina propria and gastric lymph node<br>- Dies naturally
	iTreg	- Secretes IL10<br>- In presence of tolerogenic dendritic cell, transition to iTreg cells<br>- Moves from gastric lymph node to lamina propria<br>- Proliferates in lamina propria and gastric lymph node<br>- Dies naturally
	Tr	- Secretes IL10<br>- Dies naturally<br>- Proliferates in the lamina propria
Epithelial	Healthy	-Damaged due to infectious bacteria<br>-Damaged due to Th1 and Th17 cells<br>-Proliferates<br>-Secretes IL6 and IL12<br>-Dies naturally
	Damaged	-Healed to heathy state in presence of IL10<br>-Dies naturally
Bacteria	Infectious	- Dies due to Th1 or Th17 or inflammatory macrophages or damaged epithelial cells<br>- Dies naturally<br>- Proliferates in the lamina propria
	Tolerogenic	- Moves from lumen to the epithelium in presence of damaged epithelial cells<br>- Becomes infectious if moves in the lamina propria compartment<br>- Proliferates in lumen and lamina propria<br>- Dies naturally

Figure 1

Click here to download Figure Fig.1.pdf

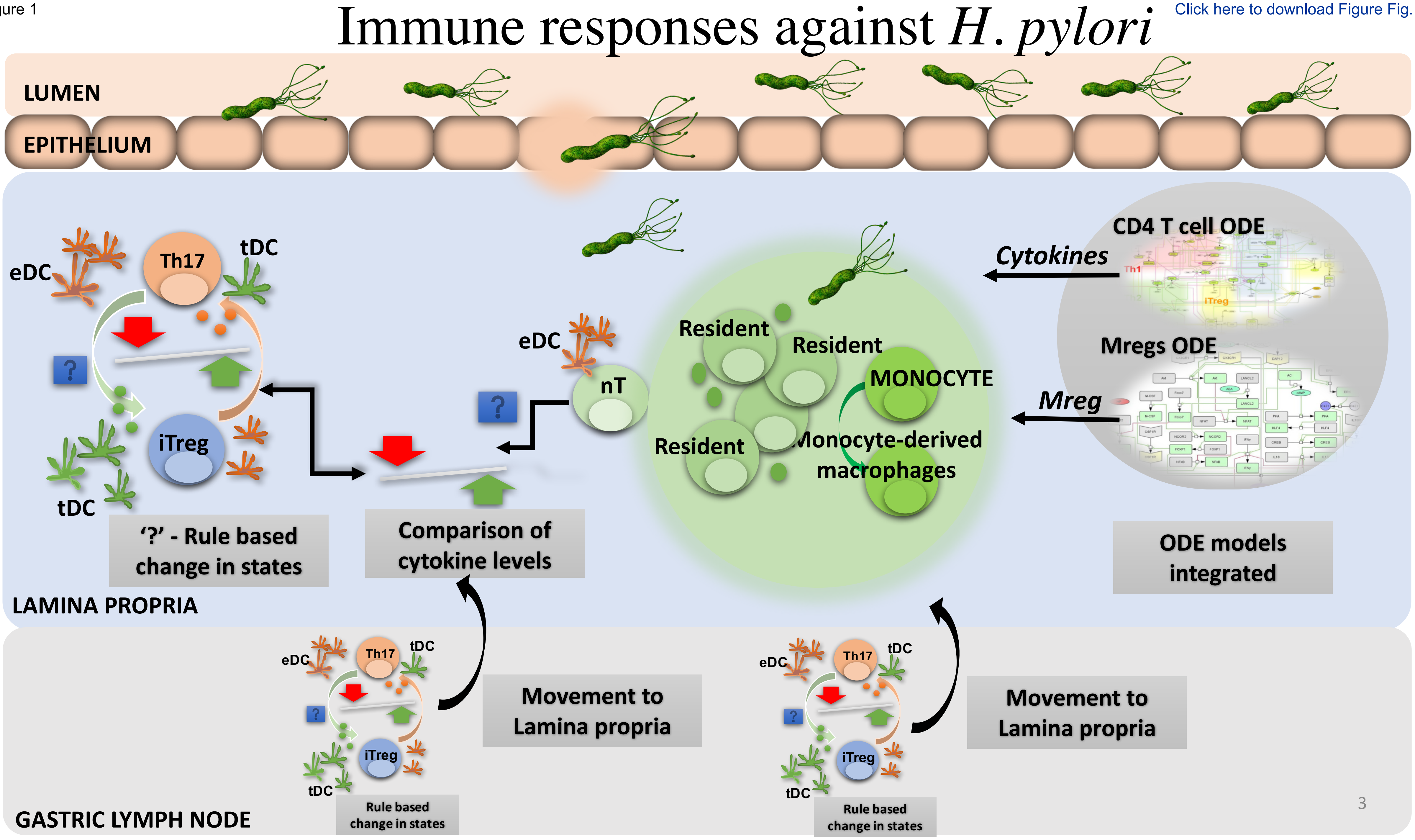

Figure 2 Click here to download Figure Fig2.pdf

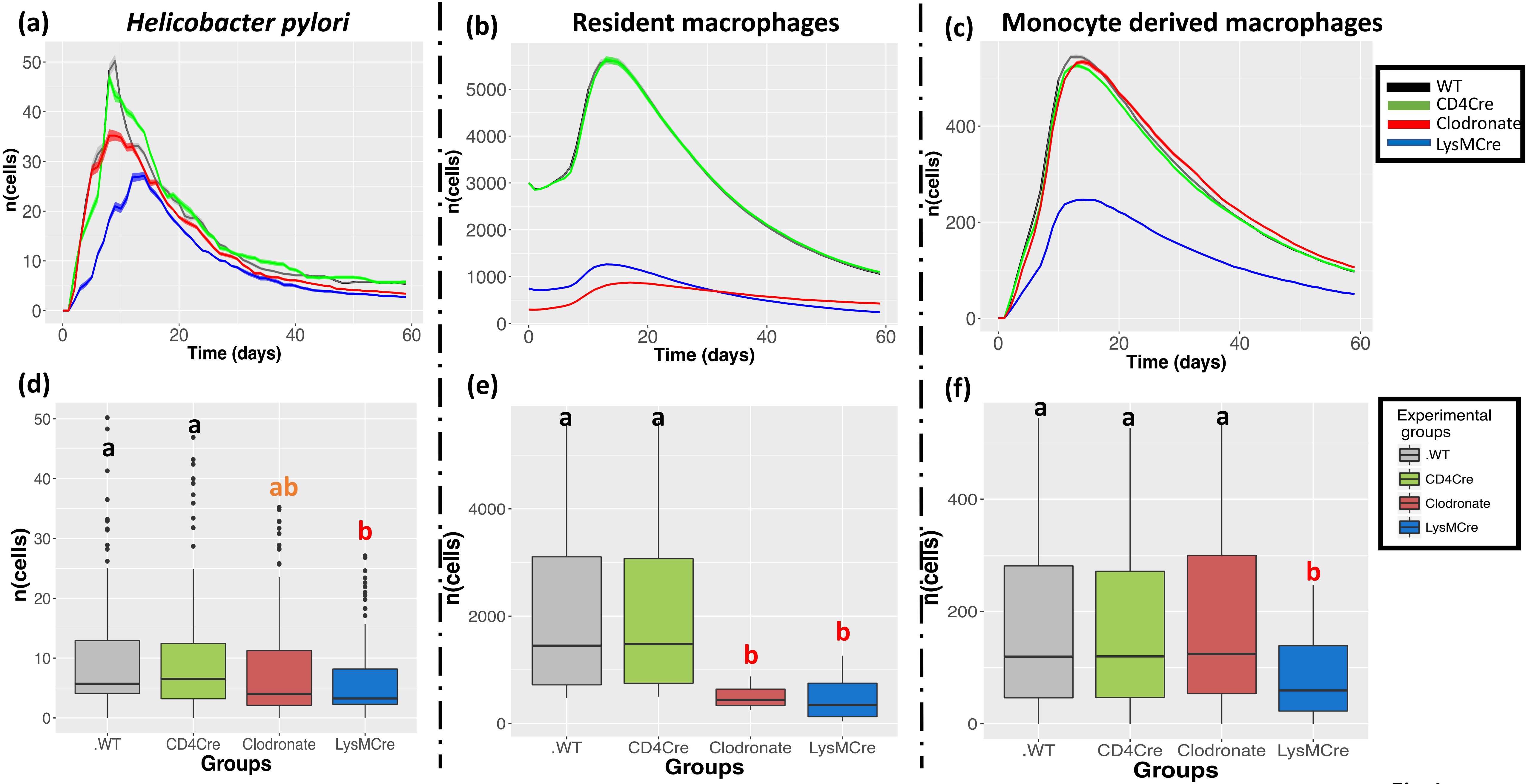

Fig 1

Figure 3

Click here to download Figure Fig3.pdf

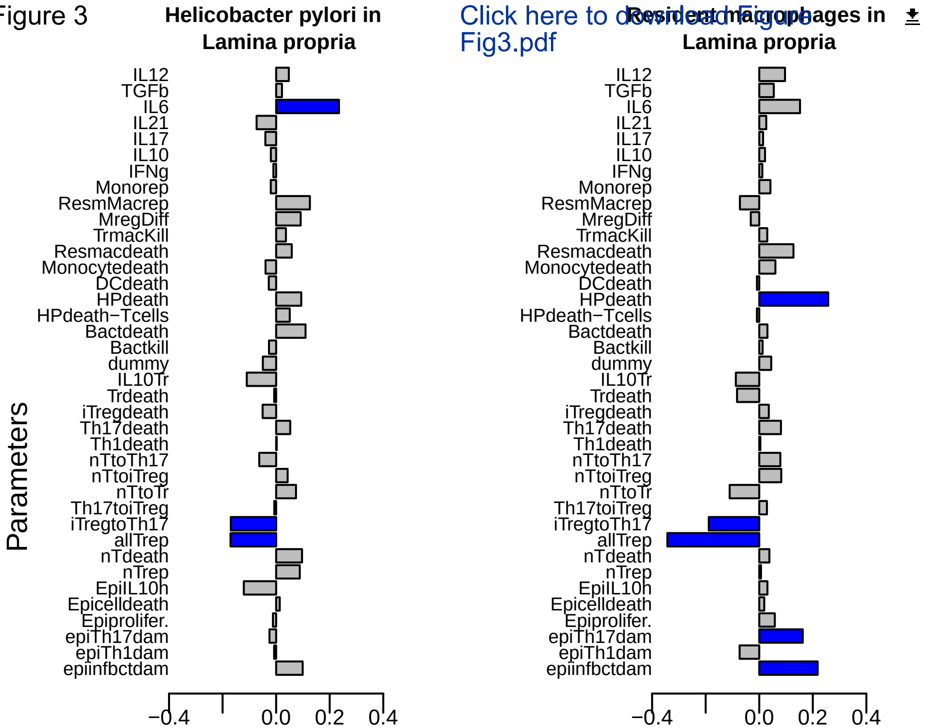

Figure 4

Click here to download Figure Fig.4.pdf

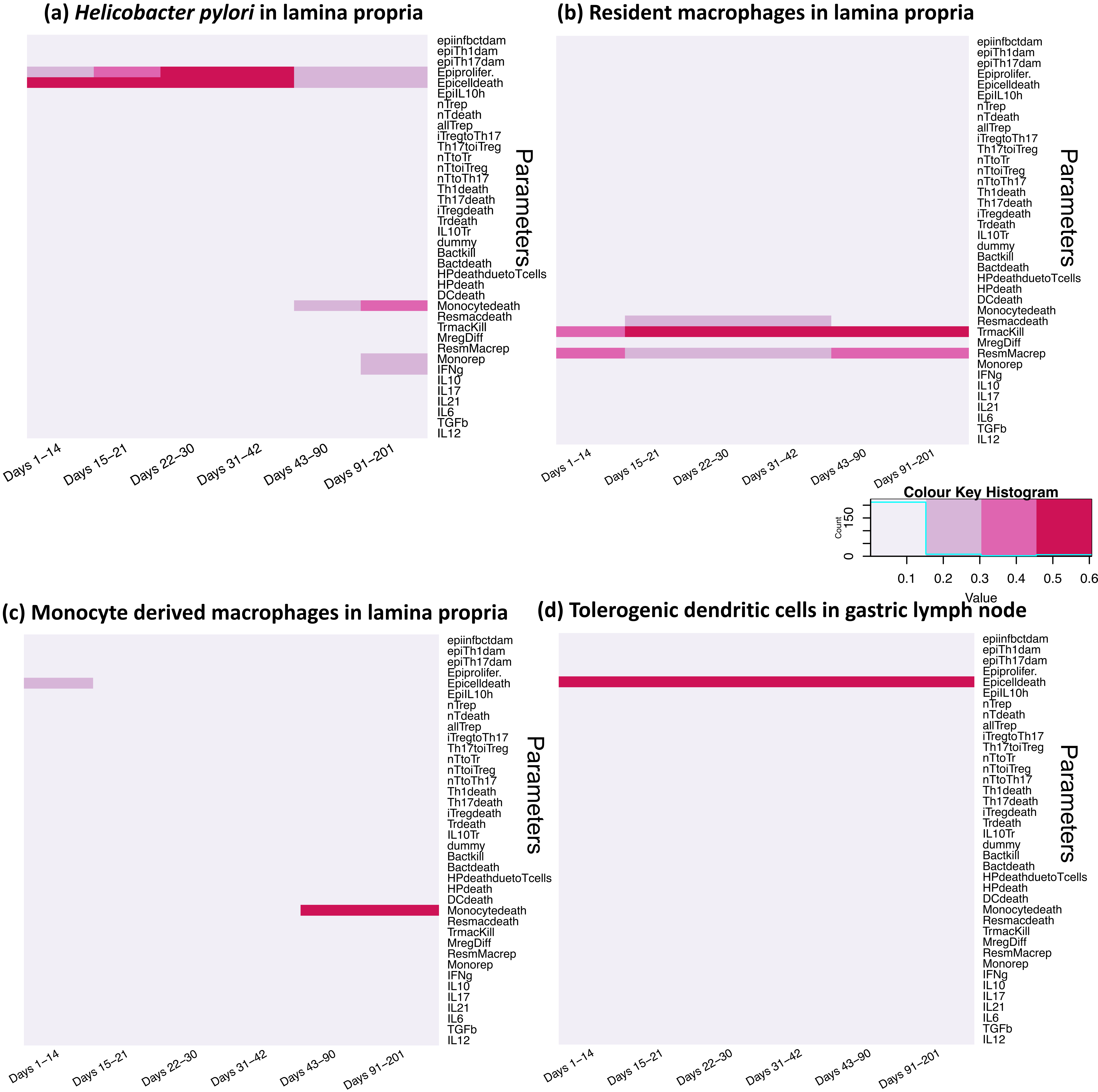

Figure 5

Click here to download Figure Fig.5.pdf

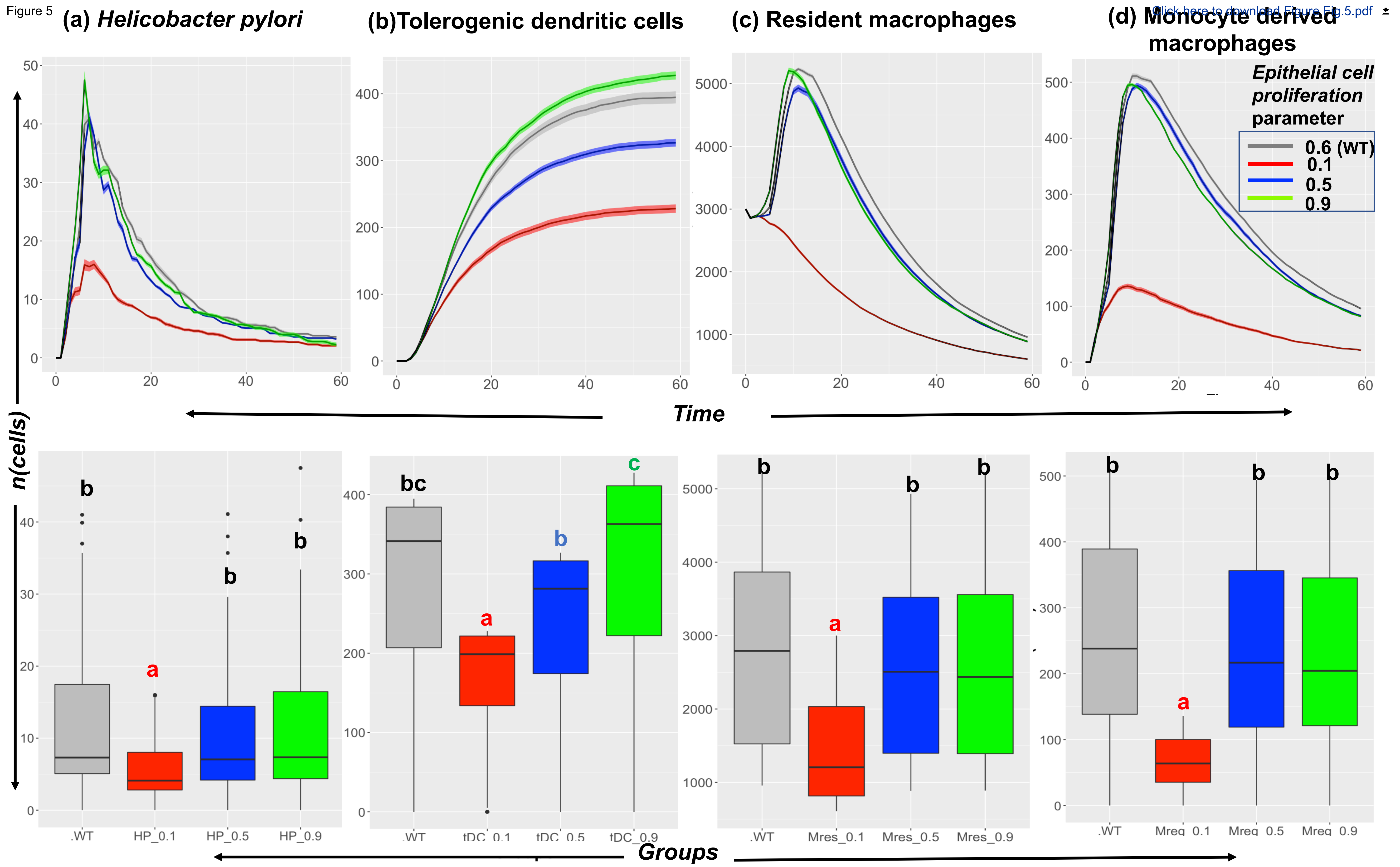

Fig S1

Click here to access/download
Supplementary Material
FigS1.png

Table S1

Click here to access/download
Supplementary Material
Table_S1.docx

Fig S2

Click here to access/download
Supplementary Material
FigS2.pdf

Fig S3

Click here to access/download
Supplementary Material
FigS3.docx

Fig S4

Click here to access/download
Supplementary Material
FigS4.pdf

Personal Cover Click here to download Personal Cover Cover-Letter.pdf

VirginiaTech
Biocomplexity Institute

Biocomplexity Institute
Washington St. (0477)
Blacksburg, Virginia 24061
540/231-7421 Fax: 540/231-2606

November 1, 2018,

Dear Editorial Board,

On behalf of me and my colleagues, enclosed please find the manuscript entitled "**High Resolution Computational Modeling of Immune Responses in the Gut**" for publication as a research article in GigaScience's new thematic series, "*Data-Driven Multicellular Systems Biology*." Our manuscript employs novel computational modeling approaches to study the immune responses in the gut.

In this manuscript, we investigated the dynamics of the immunoregulatory mechanisms triggered by *Helicobacter pylori*, using a high-performance computing driven ENteric Immunity SImulator multiscale model (ENISI MSM). The immune responses to *H. pylori* were simulated in a high-resolution hybrid model integrating various spatiotemporal scales encompassing agent-based model (tissue), ordinary differential equations (cellular) and partial differential equations (cytokine and chemokine gradients) based methods. The specific questions we address are critical host response factors for successful colonization of *H. pylori* in the gastric niche.

Our key findings using the new ENISI gut model and combined two-staged global sensitivity analysis, identified epithelial cell proliferation as a key factor in the gastric colonization of *H. pylori*. For the two-staged analysis, we used partial rank correlation coefficient regression for screening in the first stage and employed a metamodel-based variance analysis for the second stage.

The simulation studies predicted that gastric colonization with *H. pylori* decreased with a decrease in the epithelial cell proliferation. Furthermore, the process was mediated by regulatory macrophages in the gastric lamina propria and tolerogenic dendritic cells in the gastric lymph nodes. In support of our conclusions, our simulations demonstrate that decreased epithelial cell proliferation decreased the colonization of *H. pylori* and the tolerogenic dendritic cells and that regulatory macrophages mediated this process.

This high-resolution model can be applied to study infectious and autoimmune diseases. More broadly, this approach provides significant predictions regarding the factors affecting the gastric colonization of *H. pylori*. Thus, we believe this work opens up many new avenues for investigation.

Based on our knowledge of the experts in the field, we would like to suggest a list of reviewers for our study-

1. Dr. Stuart Sealfon's (admin.sealfon@mssm.edu) work is focused on integrating experimental and theoretical approaches to research on receptor structure, cell signaling and systems biology. His research targets the identification of signaling pathways activated by drugs for Parkinson's diseases and new receptor complexes implicated in neurological disorders. We believe his expertise in integrating systems biology based approaches with experimental findings would help in the enhancement of predictions obtained from the model.

2. Dr. Yoram Vodovotz's (vodovotzy@upmc.edu) research is focused on creating computational

models based on high dimensional dynamic data to study the inflammatory responses in a disease specific manner. His work focuses on building hybrid equation/agent based models to study acute inflammation, trauma and autoimmune disorder such as sepsis. We believe his expertise on the computational modeling approaches applied to inflammation will add great value to the findings from the study.

3. Dr. H. Steven Wiley's (Steven.Wiley@pnnl.gov) work combines the techniques of molecular and cellular biology with computational biology and bioinformatics. His research is focused on developing methods for quantitative analysis of receptor dynamics and he has published computer models of underlying cellular process. We strongly believe his expertise on using computational biology approaches for modeling cellular process, would be very relevant for the review process.

Any 'Data-Driven Multicellular Systems Biology' editor who deals with using modelling and simulation such as Guest Editor: Paul Macklin, through whom we learned about this journal, would be appropriate.

In compliance to authorship requirements, all authors have contributed to the work presented herein and all authors have seen and approved the content prior to submission. This manuscript has not been submitted or been accepted elsewhere.

If you have any questions, please contact us.

Sincerely,

Dr. Raquel Hontecillas
Professor of Immunology
Director, Nutritional Immunology and Molecular Medicine Laboratory
Director, Modeling Immunity to Enteric Pathogens
Biocomplexity Institute of Virginia Tech
1015 Life Science CIR, Washington Street,
Blacksburg, VA 24061
Phone: Office: 540-231-7276
