# Supplementary material for: High-resolution computational modeling of immune responses in the gut
Source: Gigascience. 2019 Jun 11;8(6):giz062. doi: 10.1093/gigascience/giz062 (PMC6559340; doi:10.1093/gigascience/giz062)
Supplement: giz062_GIGA-D-18-00435_Revision_2 [file giz062_giga-d-18-00435_revision_2.pdf]

GigaScience

High-Resolution Computational Modeling of Immune Responses in the Gut

--Manuscript Draft--

Manuscript Number:	GIGA-D-18-00435R2
Full Title:	High-Resolution Computational Modeling of Immune Responses in the Gut
Article Type:	Research
Funding Information:	Defense Threat Reduction Agency (HDTRA1-18-1-0008) — Dr. Josep Bassaganya-Riera<br>Dr. Raquel Hontecillas
Abstract:	Background: Helicobacter pylori causes gastric cancer in 1-2% of cases, but is also beneficial for protection against allergies and gastroesophageal diseases. An estimated 85% of H. pylori-colonized individuals do not present any detrimental effects. To study the mechanisms promoting host tolerance to the bacterium in the gastrointestinal mucosa and systemic regulatory effects, we investigated the dynamics of immunoregulatory mechanisms triggered by H. pylori using a high-performance computing driven ENteric Immunity Simulator multiscale model. Immune responses were simulated by integrating an agent-based model, ordinary and partial differential equations.<br>Results: The outputs were analyzed using two sequential stages: the first used a partial rank correlation coefficient regression-based and the second employed a metamodel-based global sensitivity analysis. The influential parameters screened from the first stage were selected to be varied for the second stage. The outputs from both stages were combined as a training dataset to build a spatiotemporal metamodel. The Sobol' indices measured time-varying impact of input parameters during initiation, peak and chronic phases of infection. The study identified epithelial cell proliferation and epithelial cell death as key parameters that control infection outcomes. In-silico validation showed that colonization with H. pylori decreased with a decrease in epithelial cell proliferation, which was linked to regulatory macrophages and tolerogenic dendritic cells.<br>Conclusion: The hybrid model of H. pylori infection identified epithelial cell proliferation as a key factor for successful colonization of the gastric niche and highlighted the role of tolerogenic dendritic cells and regulatory macrophages in modulating the host responses and shaping infection outcomes.
Corresponding Author:	Raquel Hontecillas, Ph.D.<br>Virginia Polytechnic Institute and State University<br>Blacksburg, Virginia UNITED STATES
Corresponding Author Secondary Information:	
Corresponding Author's Institution:	Virginia Polytechnic Institute and State University
Corresponding Author's Secondary Institution:	
First Author:	Meghna Verma, M.S.
First Author Secondary Information:	
Order of Authors:	Meghna Verma, M.S.
	Josep Bassaganya-Riera, Ph.D.
	Andrew Leber, Ph.D.
	Nuria Tubau-Juni, M.S.
	Stefan Hoops, Ph.D.
	Vida Abedi, Ph.D.
	Xi Chen, Ph.D.
	Raquel Hontecillas, Ph.D.

Powered by Editorial Manager® and ProduXion Manager® from Aries Systems Corporation

**Order of Authors Secondary Information:**	
**Response to Reviewers:**	Point by point response to the Reviewer reports<br><br>We would like to thank the reviewers and editors for taking time to review our manuscript entitled “High-Resolution Computational Modeling of Immune Responses in the Gut” and for providing valuable and constructive criticism. The review process has been helpful in the improvement of our submission. We have considered the comments that were made and have prepared the following point-by-point response. We hope that the revised version of the manuscript can now be accepted for publication. Thanks in advance.<br><br>Reviewer #1: The unit of sizes of the model grid can't be right (e.g. grid is 30nm x 10 nm). Animal cells should have measurements in the order of micrometres instead of nanometres. Please check if these are just typos, or do these errors affect any aspect of the simulation, such as diffusion.<br>Response: We thank the reviewer for pointing this out. We fixed the typos and the unit size of the model grid are 30 μm x 10 μm. These typos do not affect any aspect of the simulations as these units are only annotations and the model takes the numbers as input. We updated the manuscript and fixed the typos throughout the manuscript. Please refer to L120 – L121, and L216, L220-L221.<br><br>Reviewer #2: The authors have made significant improvements to the manuscript and thoroughly responded to reviewer comments. One major concern remains surrounding the authors' response to questions around the grid dimensions. The dimensions for the entire grid are given in nm which is smaller than a single cell. Furthermore they state that there are no limits to cell(agent) occupancy per grid compartment. This is rather confusing and calls into question how much spatial information is really contained in this model (e.g. if cytokines are diffusing over the 30nm grid what does that mean for the concentrations that individual cells (measured in micrometers)are seeing?). Based on the author responses it appears that the model is a multi-compartment model with well-mixed discrete agents in each compartment rather than a spatio-temporal model as they claim.<br>Response: We thank the reviewer for their comment.<br>We thank the reviewer for pointing out the concern regarding the dimensions of the grid. The correct dimensions of the grid are 30 μm x 10 μm. We updated the manuscript and fixed the typos. Please refer to L120 – L121, and L216, L220-L221.<br>The mention regarding no limits to cell (agent) occupancy refers to the cells (agents) having no physical size. Further, once a cell (agent) dies it is removed from the simulation to minimize the computational costs of agents that do not contribute to the biology.<br>The model output contains information about the x and y co-ordinate of the agents at every time point. The cytokines and internal signaling pathways that drive functional fates of cells are well mixed within a cell, i.e., we have only temporal resolution within the cell during a time step. However, the production, degradation, and diffusions are cell specific thus the cytokine concentration results are also spatio-temporal. Since, the model is capable of providing information regarding spatial co-ordinates over time, we claim the model to be a spatio-temporal model. We updated the manuscript, please refer to L163-L170.<br><br>Please also ensure that your revised manuscript conforms to the journal style, which can be found in the Instructions for Authors on the journal homepage.<br>Response: The revised manuscript conforms to the journal style.
**Additional Information:**	
**Question**	**Response**
Are you submitting this manuscript to a special series or article collection?	No
**Experimental design and statistics**<br><br><br>Full details of the experimental design and	Yes

Powered by Editorial Manager® and ProduXion Manager® from Aries Systems Corporation

statistical methods used should be given in the Methods section, as detailed in our Minimum Standards Reporting Checklist. Information essential to interpreting the data presented should be made available in the figure legends.<br><br>Have you included all the information requested in your manuscript?	
**Resources**<br><br>A description of all resources used, including antibodies, cell lines, animals and software tools, with enough information to allow them to be uniquely identified, should be included in the Methods section. Authors are strongly encouraged to cite Research Resource Identifiers (RRIDs) for antibodies, model organisms and tools, where possible.<br><br>Have you included the information requested as detailed in our Minimum Standards Reporting Checklist?	Yes
**Availability of data and materials**<br><br>All datasets and code on which the conclusions of the paper rely must be either included in your submission or deposited in publicly available repositories (where available and ethically appropriate), referencing such data using a unique identifier in the references and in the "Availability of Data and Materials" section of your manuscript.<br><br>Have you have met the above requirement as detailed in our Minimum Standards Reporting Checklist?	Yes

Manuscript
Click here to access/download;Manuscript;Manuscript_Revised.docx
Click here to view linked References

High-Resolution Computational Modeling of Immune Responses in the Gut

Meghna Verma[1,2], Josep Bassaganya-Riera[1], Andrew Leber[1], Nuria Tubau-Juni[1],

Stefan Hoops[1], Vida Abedi[1], Xi Chen[3], Raquel Hontecillas[1,*]

[1]Nutritional Immunology and Molecular Medicine Laboratory, Biocomplexity Institute of Virginia Tech, Blacksburg, VA 24060, USA.
[2]Graduate Program in Translational Biology, Medicine and Health, Virginia Tech, Blacksburg, VA, 24061, USA.
[3]Grado Department of Industrial and Systems Engineering, Virginia Tech, Blacksburg, VA, USA.

*** Correspondence:**
Dr. Raquel Hontecillas
Email: rmagarzo@vt.edu

Keywords: agent-based model, ordinary differential equation, Gaussian process, *Helicobacter pylori*, high-performance computing, metamodel, sensitivity analysis, immune system, dendritic cells, macrophages.

Email address for all authors:

Meghna Verma: meghna89@vt.edu
Josep Bassaganya-Riera: jbassaga@vt.edu
Andrew Leber: ajleber@vt.edu
Nuria Tubau-Juni: nuriaj@vt.edu
Stefan Hoops: shoops@vt.edu
Vida Abedi: vidaabedi@gmail.com
Xi Chen: xchen6@vt.edu
Raquel Hontecillas: rmagarzo@vt.edu

Abstract

Background: *Helicobacter pylori* causes gastric cancer in 1-2% of cases, but is also beneficial for protection against allergies and gastroesophageal diseases. An estimated 85% of *H. pylori*-colonized individuals do not present any detrimental effects. To study the mechanisms promoting host tolerance to the bacterium in the gastrointestinal mucosa and systemic regulatory effects, we investigated the dynamics of immunoregulatory mechanisms triggered by *H. pylori* using a high-performance computing driven **EN**teric **I**mmunity **Si**mulator multiscale model. Immune responses were simulated by integrating an agent-based model, ordinary and partial differential equations.

Results: The outputs were analyzed using two sequential stages: the first used a partial rank correlation coefficient regression-based and the second employed a metamodel-based global sensitivity analysis. The influential parameters screened from the first stage were selected to be varied for the second stage. The outputs from both stages were combined as a training dataset to build a spatiotemporal metamodel. The Sobol' indices measured time-varying impact of input parameters during initiation, peak and chronic phases of infection. The study identified epithelial cell proliferation and epithelial cell death as key parameters that control infection outcomes. *In-silico* validation showed that colonization with *H. pylori* decreased with a decrease in epithelial cell proliferation, which was linked to regulatory macrophages and tolerogenic dendritic cells.

Conclusion: The hybrid model of *H. pylori* infection identified epithelial cell proliferation as a key factor for successful colonization of the gastric niche and

highlighted the role of tolerogenic dendritic cells and regulatory macrophages in modulating the host responses and shaping infection outcomes.

1. Background

Computational modeling of the immune response dynamics can provide novel insights and facilitate the systems level understanding of the interactions at the gastric mucosa during infection. Ordinary differential equation (ODE-based methods are deterministic and based on the average response of cells over time. Dynamical models are used in immunology for system-level analyses of CD4+ T cell differentiation [1], macrophage differentiation [2], immune responses elicited by *Clostridium difficile* infection [3], co-infections [4], and in cancer and immunotherapy [5]. However, ODE-based models lack the spatial aspects and the features to study the organ and immune cell topology over time. Agent-based models (ABM) employ a bottom-up approach that focuses on the spatial and temporal aspects of individual immune cells, unlike the ODE-based methods. This rule-based method includes agents that act as local entities which interact locally with other agents, move in space, and follow set of rules representing their role in a given system and contribute towards generating an emergent behavior. Since, the immune system is a complex dynamical system [6] wherein the components *i.e.,* the immune cells move in space and time changing their location, ABMs are useful tools that can be employed to understand biological mechanisms and the hidden insights.

Helicobacter pylori is a gram-negative bacterium that has persistently colonized the human stomach since early evolution [7] [8] and is currently found in over 50% [9] of the global population. *H. pylori* has co-evolved with humans for thousands of years, such that an estimated 85% of the *H. pylori*-colonized

individuals, do not present any detrimental effects. Thus, the vast majority of carriers (*i.e.,* up to 75%) remain asymptomatic, while only 15% develop ulcers, and less than 3% develop cancer. Further, growing and sometimes contradictory evidence from recent experimental, clinical studies and epidemiological studies suggest that *H. pylori* might provide protection against obesity-related inflammation and type 2 diabetes [10], esophageal, cardiac pathologies, childhood asthma and allergies [11] and autoimmune diseases. In this context, it is crucial to understand the mechanisms that promote host tolerance to the bacterium in the gastrointestinal mucosa and its systemic regulatory effects since these have been linked to the beneficial commensal aspects of *H. pylori*-human host interaction. Computational models provide a cost-effective and predictive way to study the complex and dynamic immune system interactions and form a non-intuitive novel hypothesis. Solving the complex puzzle of immunoregulatory mechanisms that include large spatiotemporal scales ranging from cellular, intracellular, tissue and organ level scales is a major unsolved challenge that requires applying computational modeling and data analytics.

An advanced hybrid model used to study the mucosal immune response during gut inflammation highlighted the mechanisms by which effector CD4+ T cell responses, contributed to tissue damage in the gut mucosa following immune dysregulation [12]. Other hybrid models with the integration of ABM, ODE, and PDE technologies, were developed to understand the dynamics of tumor development [13] and tumor growth models [14]. These combined techniques have been used to develop multi-organ models in various situations, including the study of granuloma formation [15] and pressure-driven ulcer formation in post spinal cord injury patients [16]. The summary of different agent-based simulators with immunology related applications are discussed and

summarized in [17, 18]. The comparison between different multiscale modeling tools and agent-based immune simulators, are discussed in [12, 19].

In this study, we utilize a high-resolution **EN**teric **I**mmunity **SI**mulator (ENISI)-based model of the stomach for simulating the mucosal immune responses to *H pylori* infection. The advanced hybrid multiscale modeling platform ENISI multiscale model (MSM) is capable of scaling up to 10^{12} agents [20]. The host immune responses initiated during *H. pylori* infection and the underlying immunoregulatory mechanisms are captured using the ENISI multiscale hybrid model. The underlying intracellular mechanisms that control cytokine production, signaling and differentiation of macrophages and T cells are modeled by using ODEs, the diffusion of cytokine values is modeled using PDEs and the location and interactions among the immune cells, bacteria and epithelial cells are modeled by using ABMs. The hybrid model thereby represents a high-performance computing (HPC)-driven large-scale simulation of the massively interacting cells and molecules in the immune system, integrating the multiple modeling technologies from molecules to systems across multiple spatiotemporal scales.

To understand the dynamics and emergent immunological patterns described by this hybrid model, we employed sensitivity analysis *(SA)*, an important part of the model analysis used to explore the influence of varying model parameters on the simulation outputs. The influence of the effects of changes in parameter values on the model output explains the model dynamics that underlay the outputs [21, 22]. Furthermore, *SA* examines the robustness of the model output at a different range of parameter values that correspond to a range of different assumptions. We employed global *SA* and conducted a two-stage spatiotemporal global *SA* approach. First, we used a regression-based

method such as the partial rank correlation coefficient (PRCC) and screened the important input parameters that were shown to have the most influence on the output cell populations obtained from the hybrid model. Second, the screened input parameters from the first stage were varied to build a second stage parameter design matrix, and the computer simulations were again run using the hybrid ENISI model. The outputs from both analytics stages were combined and used as a ‘training dataset’ to build a spatiotemporal Gaussian process based metamodel. Finally, variance-based decomposition global *SA* was used to compute the Sobol’ indices and the most influential parameters over the course of infection were identified. The data analytics methods conducted on the hybrid model identified the epithelial cell parameters such as epithelial cell proliferation as the most influential ones, required for the successful colonization of *H.* pylori in the gastric microenvironment.

2. Methods

2.1 Hybrid multiscale *Helicobacter pylori* infection model

We developed a multi-compartment, high-resolution, hybrid ABM/ODE/PDE model to capture the dynamics of the immune response during *H. pylori* colonization of the gastric mucosa. The model has a spatial discretization such that the dimension of the entire (two-dimensional, (2D)) grid is 30 µm x 10 µm. An individual grid cell for our simulation is 1µm x 1µm, however, this is a configurable run parameter and can be changed without modifying the model. An individual grid cell is a unit wherein all the agents located within that location have the same cytokine environment, *i.e.,* for all the agents in that location, ENISI-MSM would send the same concentration of the cytokines to COPASI.

The entire grid is divided within into four functionally and anatomically distinct sized compartments: lumen, epithelium, lamina propria and gastric lymph node. In the model, there are multiple cells and cell types (i.e., agents) within this dimensional grid. At the beginning of each simulation cycle, the cells (agents) are randomly placed within the within the 2D grid. The separation of different types of agents, corresponding to different cell types, into compartments within the grid is based on the conceptual framework that underlines the model, which is based on author's expertise and available information. Currently the individual agents do not have any physical size meaning such that there is no limit of agents within each individual spatial grid. The model is initialized with the concentration of different cell types (*i.e.* agents for *e.g.* macrophages) at the beginning of the simulation by the user.

The use of a border implementation permits the migration of agents (cells) across compartments and facilitates the unidirectional and bidirectional movement of the agents. At the cellular scale, ENISI MSM, simulated epithelial cells, macrophages, dendritic cells (DC), CD4+ T cells and bacteria that are implemented as agents in the model. At the intracellular scale, calibrated ODE-based models of T cells [23] and macrophages [2] were used to represent the intracellular pathways controlling cytokine production. The CD4+ T cell ODE model was calibrated using the experimental data provided in the Table S1 of [23]. The Particle Swarm algorithm implemented in COPASI was used to determine unknown model parameter values and fully calibrate the CD4+T cell ODE model, the details are described in [23]. The intracellular macrophage ODE model was calibrated using a combination of sourced and new data generated from *in vitro* macrophage differentiation studies, that were compiled into a dataset provided within S2 file of [2]. The parameter values are specified within

the previously published manuscripts - CD4+ T cell ODE model (Carbo, Hontecillas et al. 2013) and macrophages [2]. The parameters of the calibrated ODEs were kept unchanged, and the ABM parameters were calibrated by approximating the output simulations such that they qualitatively resembled the patterns observed in a mouse model of *H. pylori* infection [24], also described in detail in section 3.1. Cytokines secreted by immune cells and their change in concentration were modeled by PDE. The degradation value of the cytokines and the diffusion constant determines the spread of the cytokine value of one grid cell to its neighboring grid cell similar to as described in [12]. The features of ABM, ODE, and PDE were combined to create a multiscale modeling environment which spanned across different orders of spatiotemporal scales. The model output contains information about the x and y co-ordinate of the agents at every time point. The cytokines and internal signaling pathways that drive functional fates of cells are well mixed within a cell, *i.e.*, we have only temporal resolution within the cell during a time step. However, the production, degradation, and diffusions are cell-specific thus the cytokine concentration results are also spatio-temporal. Since, the model is capable of providing information regarding spatial co-ordinates over time, we claim the model to be a spatio-temporal model.

The code for the hybrid model is freely accessible and can be downloaded at https://github.com/NIMML/ENISI-MSM. The detailed instructions for the usability, instructions on 'how to run a simulation' and codes for creating specific examples presented here are presented in Additional file *S1*. The SciCrunch.org database assigned research identification initiative ID (RRID) for ENISI-MSM is RRID:SCR_016918. The design of the implementation of the code structure is

depicted in the Additional file ***Fig S1***. The hybrid model is implemented in C++ and utilized the Repast HPC library (https://repast.github.io/repast_hpc.html) [25]. For the ODEs, we utilized COPASI [26], an ODE-based modeling tool used in computational biology. The rules in the model that described the interaction of *H.* pylori with the gastric mucosa and the immune responses resulting from the infection are derived from the findings in our previously published studies [1, 2]. Specifically, this hybrid model reproduced the immune responses generated by the interaction *H. pylori* and the resident macrophages as shown in the mouse model of *H. pylori* infection [24]. The rules for each cell type in the *H. pylori* infection are summarized in ***Table 1****.* A pictorial representation of the rules is depicted in *Fig 1.* These cell types represented as agents, act according to the rules (as in ***Table 1*****)** that are updated at discrete simulation cycle.

Fig 1. ***Helicobacter pylori* infection schematic diagram of the hybrid ABM ODE model**

The model comprises four compartments, i) the lumen that contains H. pylori and bacteria, ii) epithelium that contains epithelial cells and dendritic cells, iii) lamina propria that contains variety of immune cells including the infiltrating effector (eDCs) and tolerogenic (tDCs) dendritic cells, monocytes, regulatory macrophages (both resident and monocyte-derived macrophages), T helper cells and naïve CD4+ T cells (nT), Th1, iTreg, Th17, Tr cells. and iv) gastric lymph node compartment that contains eDCs, tDCs, Th1, Th17, iTreg and nT. The Tr cells in the lamina propria are the type 1 regulatory (Tr1) T cells with regulatory function whose expansion is largely dependent on environmental IL-10. These are different than iTreg which are T cells differentiated from naïve T

cell in presence of tolerogenic dendritic cells and TGF-β cytokine The two calibrated ODEs for T cells and regulatory macrophages are integrated as the ODE components in the hybrid model. The cellular agents are simulated in a two-dimensional grid space with their behavior defined by a set of rules during a course of H. pylori infection.

Model description

ENISI MSM is a multiscale agent-based modeling platform for computational immunology which was built on our previous works, ENISI-MSM [12] that integrated COPASI, the ODE solver, ENISI, an agent based simulator.

Spatial discretization

The model has a spatial discretization such that the dimension of the entire (two dimensional) grid is 30 μm x 10 μm. An individual grid cell is 1μm x 1μm, however, this is a configurable run parameter and can be changed without modifying the model. The four functionally and anatomically distinct sized compartments are separated by border implementation such that the dimensions of the four compartments are lumen (2 μm), epithelium (1 μm), lamina propria (5 μm) and gastric lymph node (2 μm). The following compartments are adjacent to each other: lumen – epithelium, epithelium - lamina propria and lamina propria – gastric lymph node. *A figure describing the spatial discretization is shown in the* Additional file ***Fig S2***.

The parameters that define the initial concentration of the agents and the diffusivity of cytokines are obtained from a properties file (*model.props* in the Howtorunasimulation folder in the GitHub repository). All the value of the parameters as listed in *Table S1*. The detailed mechanism that each parameter

corresponds to is described in the second column, *parameter description,* of *Table S1*. We demonstrate below how we obtain a count of thousands resident macrophages. For *e.g.,* if the initial concentration of resident macrophages in the lamina propria is 30, the total number of these resident macrophages can be calculated by the equation described below -

n(resident macrophages) = $\text{size}_{\text{compartment}}$(lamina propria) x $\text{concentration}_{\text{intial}}$ (resident macrophages)

n(resident macrophages) = (30 x 5) x 30 = 4500.

Time Step size

The time step size is 1 tick ~ 1 day which was obtained during the process of qualitatively comparing the output to the results from the mouse model of *H. pylori* infection. For *e.g.,* the peak of resident macrophages in lamina propria (refer *Fig 2b, d*) is observed at ~21 days which is similar to the results obtained in *Fig 2A* described in [24] (also described in detail in section 3.1).

Updating

Each agent has an *'act'* function within the code that describes the rules implemented for each of the agent groups. At every simulation cycle, each agent inspects its location and updates its state. If the agents were T cells and macrophages, they obtained the cytokine concentration from the ValueLayers, sent that information to COPASI that calculated the differentiation subtype of the agent and cytokines to be secreted that into the environment [12]. The input to the ODEs were the cytokine values at the agent's location. Thus, the intracellular ODE models were utilized to determine and update the state. Each agent

proliferated, died, changed its state and moved across the compartment, following the set of rules defined for them.

The COPASI setup for the solver used the LSODA (Livermore Solver for Ordinary Differential Equations) differential equation solver. The default values for the setup such as the - relative tolerance (1e-6), absolute tolerance (1e-12) and maximum internal steps of 10000 were maintained. The ENISI MSM sends the current concentrations of the cytokines to COPASI. COPASI uses those values to integrate the deterministic model for one tick, i.e., 1 day. The resulting time series of cytokine concentrations are used to update the cytokine value in the ABM/PDE system. COPASI simulates different model for each relevant cell type.

The ENISI MSM PDE solver uses a simple numerical scheme to solve the PDEs (https://github.com/NIMML/ENISI-MSM/tree/master/src/diffuser) and process distributed value layer (https://github.com/NIMML/ENISI-MSM/blob/master/src/grid/ValueLayer.h). The ValueLayer stores the value for a grid space and provides methods to change the values of individual grid cells. The Diffuser is used to diffuse the values of the ValueLayer using diffusion (d) and degradation (delta) constants as described in [12]. The diffusion constant determines the migration of values of a grid cell to its neighboring grid cells. As implemented in ValueLayer library, the diffusion of cytokines follows the equation shown below also described in Mei el al, 2015. Here, v_n is the value of the grid cell itself at step n. The values of c_{delta} and c_d are degradation and diffusion constant respectively.

$$v_n = v_{n-1} + c_{delta} * [\sum(c_d^{neighbor} * v_{n-1}^{neighbor}) - 6.0 * v_{n-1}]$$

0.3	1.2	0.3
1.2	-6.0	1.2
0.3	1.2	0.3

The PDE solver uses the above number scheme $c_d^{neighbor}$ for the diffusion process. The step size c_{delta} is automatically adjusted at the beginning of the simulation based on the degradation and diffusion constants to avoid underflow errors, *i.e.,* multiple PDE steps are in general executed per tick. The grid size is the identical with the spatial discretization for the agents.

Movement

The cells and bacteria agents presented in the model have Brownian motion and move randomly within the compartment. Brownian movement is an inherent property of a cell. Depending on cell phenotypes the movement can vary, but all cells with the same phenotype exhibit similar movements. Additionally, chemokine-driven movement is dependent on chemokine concentration in a tissue site. The capability of chemokine-driven movement exists in ENISI-MSM if the right chemokines are represented in the model. However, the focus of this model was to investigate changes in cell phenotype and not chemokine-driven movement of cells. Thus, the chemokines driving the movement are not represented in the current model. Cell migration is implemented in the code as the *move()* function for each of the cells and agents, which call the

moveRandom() function from the (https://github.com/NIMML/ENISI-MSM/src/compartment/Compartment.cpp) file.

The hybrid model simulations were run on an Ivy Bridge-EX E7-4890 v2 2.80 GHz (3.40 GHz Turbo) quad processor nodes. The code was parallelized such that the simulation time on a single node with four parallel tasks, varied between 9-10 minutes. This runtime was based on the model parameters at the initiation stage, which included the number of immune cell, bacteria, epithelial cells, number of time steps, and size of the two-dimensional grid. To facilitate the investigation of the mechanisms underlying host responses during *H. pylori* infection, anatomical and functional compartments were spatially linked such that the agents had both unidirectional and bidirectional movement. All the agents worked in a synchronous format wherein the two agent populations (macrophages and T cells) made function calls to their respective ODE models [2] [23]. These agents used the varying cytokine concentration (*i.e.,* environment variable) in their grid spaces as inputs to the ODE model, and these models were run using COPASI [26]. ***Table 2*** shows information on the agents and the states that they can acquire.

Name of agents	States it can acquire	Name of the states in the hybrid model
Helicobacter pylori	0	*H. pylori*
Macrophages	0	Monocyte
	1	Resident
	2	Regulatory
	3	Inflammatory
Dendritic cells	0	Immature
	1	Effector
	2	Tolerogenic
T cell	0	Naïve
	1	Th1
	2	Th17
	3	iTreg
	4	Tr
Epithelial	0	Healthy
	1	Damaged
Bacteria	1	Infectious
	2	Tolerogenic

Table 2*. *List of all the agents and the states they can acquire.

All the agents can acquire at least 1 and at the most 5 states. The names chosen for the acquired states are closely related to their functional properties based on the underlying "rules".

2.2 Global sensitivity analysis

To conduct the global *SA,* we determined a list of 38 parameters to be varied that were selected based on the calibration process (wherein the parameters that did not show a lot of variation were not included). A range of values (maximum and minimum) was specified for each of the parameters (refer to Additional file ***Table S1***) by expert judgment, summarized by bounded intervals. The practice of using expert judgment is known in the SA field as supported in [27]. As discussed in [28], one of the challenges encountered using ABM is the

process of determining the parameter values, for *e.g.* this may include the lack of the availability of experimental techniques to measure such parameters. The values of the parameters for the model presented here are obtained via the best guess based on the qualitative comparison of the computer model outputs with that of the experimental results obtained from the mouse model of *H. pylori* infection (Viladomiu, Bassaganya-Riera et al. 2017) (as described in detail in Section 3.1). Since, the source of the parameters is not known we estimated the values to fit the data obtained from the mouse model of infection.

The values of these parameters were normalized within the range of 0 and 1 for *SA* purposes. We employed a two-stage metamodeling methodology to determine the influence of each input parameter to the model output, in a high dimensional screening setting inspired by [29]. The step-wise procedure is described in the Additional file, *Fig S3.* All the files for global *SA* are freely accessible and can be downloaded at https://github.com/NIMML/Sensitivity-Analysis.

The two-stage global *SA* is described in detail in the below section. To summarize, for the first stage the input parameter matrix was designed using the method described in Moon, Dean et al. 2012 and simulations were run using the hybrid computer model. The simulation output from the first stage was analyzed using PRCC as it was computationally efficient, and the active inputs (significant effect) were screened to reduce the input parameter space. Second, the active parameters were varied whereas the inactive parameters from the first stage were maintained at a nominal value for the input parameter matrix design to be employed for the second stage. Third, the simulation outputs from both stages were combined and used as a training dataset to fit a spatio-temporal metamodel. Fourth, the unknown model parameters for the spatio-temporal

metamodel were estimated using the maximum log-likelihood function. The spatio-temporal metamodel was used as a substitute for the hybrid computer model, and the variance-decomposition method was used to compute the Sobol' total and first-order indices. Overall, we employed both approaches, PRCC based (for screening) and Sobol' indices calculation to perform a complete global *SA* of the hybrid computer model. The following sections, describe a detailed step by step explanation of the procedure.

Design of two-stage experiments and analysis

The input for the hybrid model are varying parameter values obtained from the design matrix and the output are the number of cells (agents) that vary over time. The first stage experiment was focused on the screening of the input variables to reduce the number of input parameters to vary for the *SA* and to limit the computational cost. Computational costs are often a limiting factor that play an important role in the inclusion of model parameters in the *SA [21].* For the design, we assumed the total number of input parameters under consideration as *d* (in our case, 38). With an assumption of a maximum of 50% active inputs that is aimed to improve the screening performance, the number of runs for stage 1, was fixed to $n_1 = 4d$, such that $n_1 > 5*d*0.5 = 2.5d$ as in [29]. To construct a $n_1 * (n_1-1)$ preliminary input parameter design matrix, X^*, needed to be constructed ([29]). The input parameter design matrix for first stage sampling was drawn from X^*.

The algorithm for the first stage design generated a design matrix $X^{(1)}$ that satisfied the below three listed properties as in [29]

i) The columns of X^* were uncorrelated thereby facilitating the independent assessments of the effects due to the input parameters.

ii) The maximum and minimum value in each input parameter column were ensured to be 0 and 1 respectively, thereby preventing any input values with larger values to have a larger influence on the response, induced by the design.

iii) The designs defined by X^* had “space-filling” properties such that all the regions of the input space were exhaustively explored.

First stage sampling plan:

The first stage input parameter design matrix $X^{(1)}$ was obtained by selecting the first *d* columns of X^*, *i.e.* $X^{(1)} = (\xi_1, \ldots., \xi_d)$. The hybrid computer model was run and the simulation outputs at these $n_1$design points were obtained.

In our case, the model comprised of d = 38 input variables. The total number of distinct input parameter design points obtained using the above procedure was n_1 = 152 (4*d = 4* 38). To account for the variability in the output, we run 20 replicates (*r)*. Thus, the total number of simulations run using the hybrid model computer simulator with $X^{(1)}$ as input parameter design matrix, were $r \; x \; n_1$ = 20 x 152 = 3040.

First stage analysis

We analyzed the outputs from first stage analysis and screened the active inputs from using PRCC. To measure the effect of input parameter on output, we performed both PRCC and the spearman rank correlation coefficient (SRCC) analysis. PRCC and SRCC were chosen because they were computationally

efficient (accounting for the low computational budget). A correlation analysis provides a measure of the strength of linear association between input and output variable [30]. A correlation coefficient between x_j and y is calculated as follows:

$$r_{x_j y} = \frac{Cov(x_j, y)}{\sqrt{Var(x_j)Var(y)}} = \frac{\sum_{i=1}^{N}(x_{ij} - \overline{x})(y_i - \overline{y})}{\sqrt{\sum_{i=1}^{N}(x_{ij} - \overline{x})^2 \sum_{i=1}^{N}(y_i - \overline{y})^2}}$$

$$j = 1,2, \dots, k.$$

where $Cov(x_j, y)$ stands for the covariance between x_j and y, and *Var* (x_j) and *Var (y)* are the variance of x_j and *y respectively*.

PRCC is performed when i) a non-linear but monotonic relation exists between the input and outputs, and ii) when little or no correlation exists between the input variables (which is guaranteed by the property (i) of our input parameter matrix, $X^{(1)}$ described above). As described in Marino, Hogue et al. 2008, the PRCC between rank transformed x_j and y is the CC between the two residuals $(x_j - \widehat{x_j})$ and $(y_j - \widehat{y_j})$ where $\widehat{x_j}$ and $\widehat{y_j}$ are rank transformed and follow the linear regression models as follows:

$$\widehat{x_j} = c_o + \sum_{\substack{p=j \\ p \neq j}}^{k} c_p x_p \text{ and } \widehat{y_j} = c_o + \sum_{\substack{p=j \\ p \neq j}}^{k} c_p x_p \,.$$

We performed the PRCC analysis on the outputs obtained from the hybrid computer model with $X^{(1)}$ as an input, using '*epi.prcc'* package in R (https://cran.r-project.org/web/packages/epiR/epiR.pdf). The significance test evaluated the strength of influence each input parameters and assessed if the PRCC

coefficients were significantly different than zero [30]. We run the PRCC analysis for 13 output cell populations (*Fig 3* shows data for two output populations and the rest of the data not shown) and identified the active input parameters using the significance test. PRCC and SRCC produced identical outputs, hence results from SRCC are not shown here. If an input parameter was shown to be significant (*$P < 0.05$)* in one of the 13 output cell populations, it was considered as an active input for the second stage input parameter design matrix. Additionally, domain expert knowledge was employed to include additional parameters, based on the biological significance, that were otherwise shown to be non-significant. In all, based on the PRCC analysis performed on the outputs obtained from the first stage simulations and domain expert knowledge, we chose 23 input parameters as active inputs for the second stage (see Additional file *Fig S4*). Thus, PRCC screened inputs at significance level $p < 0.05$ and inputs based on expert knowledge were selected as active inputs to be varied for the second stage sampling plan.

<u>Second stage sampling plan</u>:

The number of active inputs obtained from the first stage analysis amounted to 23 parameters out of the initial set of 38 parameters. We followed the design described in [29] for the second stage and the number of design points amounted to, n_2= 100%* 5*a where '*a'* stands for the number of active inputs from the first stage. This resulted into n_2= 23*5 = 115 parameters combinations for the second stage input parameter design matrix. Since outputs from both stages are to be combined for second stage analysis, per [29], the design for the second stage was chosen to build on top of $X^{(1)}$. The sampling phase design algorithm ensured

that the columns satisfied the properties (i) (uncorrelated design points) and (ii) (between values 0 and 1) as listed in the previous section. We constructed the 115 x 38 (115 parameter setting and 38 parameters) design matrix for the second stage that incorporated the 23 active inputs obtained from the PRCC screening in the first stage output analysis. After combining the design points from both the stages, the parameter design matrix *X* with space filling properties contained 267 (152 from the first stage and 115 from the second stage) design points.

Second stage analysis

We run the computer code for the hybrid model with the second stage input parameter design matrix (with 115 (n_2) design points), for 20 (r) replicates, which amounted to 115 x 20 (2300) runs. The outputs from the first stage (152 x 20 runs) and second stage (115 x 20 runs) were combined to provide the training data to build a spatio-temporal metamodel. For the second stage analyses, we utilized a metamodeling-based approach. Metamodels are surrogate models that can be used as a substitute for the simulation model [31]. The use of metamodels reduces the computational budget, cost of analysis, and are useful options in cases when the simulation model is expensive to run (in our case 9-10 minutes for 1 design point) [31]. The various metamodeling techniques used to build surrogates for a computer model output include linear regression models, neural networks, high dimensional model representation methods, Gaussian process (GP) regression models, polynomial chaos expansion and more that are discussed in length in [32, 33]. Amongst these, GPs are one of the most popular emulators as it allows modeling of fairly complex functional forms. The GPs not only provide prediction at a new point but also an estimate of the uncertainty in

that prediction [32]. A GP is a stochastic process for which any finite set of y-variables has a joint multivariate Gaussian distribution [34] [32]. Suppose, $y_j(w)$, the simulation response obtained on the *j*th simulation replicate, at a design point $w = (X^T, t)^T \in \chi \times \mathrm{T}$, it can be described as follows:

$$y_j(w) = Y(w) + \varepsilon_j(w) = \beta_0 + M(w) + \varepsilon_j(w), \qquad (1)$$

where *Y(w)* represents the mean function of $y_j(w)$, the quantity of interest that we intend to estimate at any design point *w.* The β_0 is a constant trend term and is assumed to be unknown. The input parameter $X \in \chi \subset \mathbb{R}^d$ and the time $t \in \mathrm{T} \subset \mathbb{R}_+$; and X is independent of t. The $\varepsilon_j(w)$ are represents the sampling variability inherent in a stochastic simulation, that are that are assumed to be independent and identically distributed across the replications at any given design point [35].

The term $M(w)$ represents a stationary Gaussian process with mean = 0 and covariance between any points was modeled as the Gaussian covariance defined in [36]. Thus, the covariance between any design points $w_a = ({X_a}^T, t_a)^T$ and $w_b = ({X_b}^T, t_b)^T$ in the random field can be modeled as-

$$Cov\big(M(w_a), M(w_b)\big) = \Gamma^2 \exp(-\textstyle\sum_{r=1}^{d} \theta_r (X_{ar} - X_{br})^2\, R(t_a - t_b; \gamma), \qquad (2)$$

wherein, $\exp(-\sum_{r=1}^{d} \theta_r (X_{ar} - X_{br})^2\,)$ models the spatial correlation between two input design points X_a and X_b in the input parameter space, whereas $R(t_a - t_b; \gamma)$ also given by exp $(-\sum_{r=1}^{d} \gamma_r\, (t_{ar} - t_{br})^2)$ models the temporal correlation between time points t_a and t_b. The parameters θ and γ represents the rate at which i) spatial correlation decreases as the points move farther in

space with the same time index, and ii) temporal correlation decreases as the time points are farther apart in time at the same input vector, respectively. Both the spatial correlation and temporal correlation are modeled using the Gaussian covariance. The parameter Γ^2 can be interpreted as the variance of *M (w)* for all *w.* The input parameter design consists of $((w_a, n_i)_{i=1}^{k})$ design points to run independent simulations with replicates applied to each of the design points. Let, $k \times 1$ denote a vector of sample averages of simulation responses given by $\bar{y} = (\bar{y}(w_1), \bar{y}(w_2), \ldots, \bar{y}(w_k))^T$, where in $\bar{y}(w_i)$ is the resulting estimate of performance measure obtained at design point w_i and $\bar{\varepsilon}(w_i)$ is the sampling variability inherent in a stochastic simulation (Ankenman, Nelson, & Staum, 2010). The equations associated with $\bar{y}(w_i)$ and $\bar{\varepsilon}(w_i)$ are described below in equation (3):

$$\bar{y}(w_i) = \frac{1}{n_i}\sum_{j=1}^{n_i} y_j(w_i) = Y(w_i) + \bar{\varepsilon}(w_i) \text{ and } \bar{\varepsilon}(w_i) = \frac{1}{n_i}\sum_{j=1}^{n_i} \varepsilon_j(w_i), i = 1,2,\ldots,k. \quad (3)$$

Similar as in (Ankenman, Nelson, & Staum, 2010), shown below in equation (4), let $\sum_M$ be the *k x k* covariance matrix across all design points and let $\sum_M(w_o, .)$ be the *k x 1* vector, $(\mathrm{Cov}[M(w_0,w_1)], \mathrm{Cov}[M(w_0,w_2)],..,\mathrm{Cov}[M(w_0,w_k)]^T$ that contains spatial covariance between the *k* design points and a given prediction point w_o. Also, let $\sum_\varepsilon$ be the *k x k* covariance matrix of the vector of simulation errors associated with the vector of point estimates $\bar{y}$, across all design points. As described in [35], the best linear predictor $Y(w_o)$ that has the minimum mean squared error (MSE) among all linear predictors at a given point $w_o = ({X_o}^T, t_o)^T$ can be given by equation (4):

$$\hat{Y}(w_o) = \widehat{\beta_o} + \textstyle\sum_M (w_0,\ .)^T \left[\sum_M + \sum_\varepsilon \right]^{-1} (\overline{y} - 1_k \widehat{\beta_0}), \qquad (4)$$

where, 1_k is the *k x 1* vector of ones and $\widehat{\beta_o}$ is estimated to be 1. The corresponding optimal MSE as in [35] is given by equation (5):

$$MSE\left(\hat{Y}(w_o)\right) = \textstyle\sum_M X_0, w_o - \sum_M \left(w_{0,}.\right)^T \left[\sum_M + \sum_\varepsilon \right]^{-1} \sum_M (w_o,.) \qquad (5).$$

To implement the metamodeling approach as described above, the unknown model parameters are estimated through maximizing the log-likelihood function. The underlying standard assumption is that $(Y(w_o),\ \bar{y}^T)^T$ follows a multivariate normal distribution, for e.g., see [35] and [37]. The function implemented in the *mlegp* package in R [38] is used for the estimation of the parameters. Once the parameters are estimated the prediction then follows equations (4) and (5).

Sensitivity index calculation

To determine the effect of input variables on the output, we employed the variance decomposition method. These methods involve the decomposition of the variance of the output as a sum of the variance produced by each input parameter [34].

We independently generated 10,000 x 38 sampling matrices, such that the parameter combinations are generated via Latin Hypercube sampling and as described in [39]. Simulations were performed using the GP spatio-temporal model as described in the previous section, and the Sobol' indices were computed as described in [40] [39]. The Sobol' method quantitatively measured the contribution of each input parameter by computing the first order and total

order index [39]. For output *Y,* input parameter matrix X_i where, *i* is the input parameters of the model, the Sobol' indices are computed as follows:

$$SI_1^{Xi} = \frac{V[E(Y|X_i]}{V(Y)},$$

and

$$SI_{tot}^{Xi} = \frac{V[E(Y|X_{\sim i}]}{V(Y)}.$$

The Sobol' first order sensitivity index SI_1^{Xi} measures the impact of one single parameter on the model output, whereas the Sobol' total order index measures the influence of X_i including all the interactions with other parameters. The First-order indices were computed using the Sobol-Saltelli's method as described in [39] [41] whereas, the total order indices were computed using Sobol-Jansen as in [39, 42].

3. Results

3. 1 Hybrid model simulations produce similar immune response dynamics observed in previously published experimental data

We first aimed to simulate the findings observed in previous gut models [24] to ensure that we obtained similar response dynamics from the hybrid ENISI model of *H. pylori* infection. As in [24], to demonstrate that the gastric mucosa

harbors a system of macrophages that contribute to the outcome of *H. pylori* infection, we created an *in-silico* Peroxisome proliferator-activated receptor gamma (PPARγ) macrophage-specific knockout (KO) model. PPARγ is an important transcription factor that controls the expression of genes that contribute to the inflammatory response once this is initiated. To disrupt the downregulation of pro-inflammatory responses, we simulated a PPARg KO system in either macrophage or T cell populations and compared the response to a wild-type system. In the model, we created three different macrophage populations, comprised of, “resident” macrophage agents that mimic the properties of the F4/80hi CD11b+ CD64+ CXCR1+ macrophages reported in [24], monocyte-derived (infiltrating) and macrophage populations with regulatory (M2, or alternatively activated) and pro-inflammatory function (M1 or classically activated) (see *Table 2*).

We simulated an *in-silico H. pylori* infection by creating four groups, i) a control - WT (representing a wild-type group), ii) CD4Cre (T cell specific PPARγ KO-lacks PPARγ gene in all CD4 T cells), iii) LysMCre (Myeloid cell specific PPARγ KO-lacks PPARγ gene in all macrophages) and clodronate group (simulating the removal of macrophages by chemical depletion via clodronate treatment). To simulate the CD4Cre group, the probabilities of a naive T cell transitioning to an iTreg cell (*p_nTtoiTreg*) and Th17 cell differentiating to iTreg (*p_Th17toiTreg*) were reduced to 5% and 10% of the control value, respectively (refer to Table S1). As described in [23], to simulate the LysMCre experimental conditions, the probabilities of i) a monocyte transitioning to a regulatory macrophage (*p_Mregdiff*) and ii) immature dendritic cells switching to tolerogenic dendritic cells (*p_iDCtotDC*) were reduced approximately to 60% and 30% of the control

value, respectively (refer to Table S1). A complete set of parameter for each of the biological KOs are included as separate columns in Table S1. Lastly, the removal of macrophages by clodronate were simulated by decreasing the initial numbers of the macrophage population including the resident macrophages. The rationale to include the clodronate group (macrophage removal) was to evaluate if depletion of phagocytic cells (terminology with respect to model, *i.e.,* monocytes, resident, monocyte-derived macrophages and inflammatory macrophages) would affect *H. pylori* colonization levels, as we have previously reported in an *in vivo* model [24]. Further, to simulate the myeloid cell PPARγ KO system, the initial population of resident macrophages were also reduced.

All the groups were initialized with equal loads of *H. pylori* agents. Ten replicates of the simulations were performed for each of the input parameter settings specific to each group. The outputs were averaged, and standard error of the means were plotted as ribbons (shaded regions) across the graphs. After running the ten replicates of the time series *in-silico* simulation, the hybrid model showed significantly (*p< 0.05*) higher levels of *H. pylori* in the WT and CD4Cre groups as compared to LysMCre KO and macrophage-depleted groups (*Fig. 2*, panel *a* and *d*).

Fig 2. Time course simulations representing the immune response during Helicobacter pylori infection.

The upper half of the plot in both the panels shows the dynamics of the population cells

over time representing the number of cells (y-axis) versus time (x-axis) in a WT (black), CD4Cre (green), clodronate (red) and LysMCre (blue) simulated in-silico

groups during H. pylori infection. The cell populations include - a) H. pylori; b) the resident macrophages and, c) monocyte-derived macrophages in the lamina propria compartment. The figures in the lower half (d-f) of both the panels, show the results for statistical comparison between the groups using ANOVA with the post-hoc analysis. The letters 'a', 'ab' and 'b' represent statistically significant differences (P<0.05) between the groups obtained after running the Tukey's Honestly Significant Difference. A side by side comparison with the bacterial load and macrophage population as observed in the mouse model of H. pylori infection are also included.

In addition to the increase in *H. pylori,* WT and CD4Cre *in-silico* experimental groups had a higher resident as well as monocyte-derived regulatory macrophages as compared to clodronate (macrophage depleted) and LysMCre groups (*Fig. 2b-c, e-f*). The results in the mouse model indicated that between weeks 2 and 3 post-infection a decrease in bacterial burden in the stomach of LysMcre mice was observed as shown in *Fig 1A* of Viladomiu, Bassaganya-Riera et al. 2017. The decrease in bacterial burden led to a significant and sustained lower colonization levels when compared to WT and CD4Cre. Similar to the results observed in the mouse model, we observed a significant decrease (*Fig 2a, d*) in the bacterial burden in the simulated LysMcre group as compared to the simulated WT and CD4cre groups. Furthermore, the results from the mouse model indicated that a significant increase in numbers of F4/80hiCD11b+ CD64+ CX3CR1+ cells (here referred to as resident macrophages in this paper), was observed in WT mice in comparison with LysMcre mice as shown in Fig. 2A, 2E of Viladomiu, Bassaganya-Riera et al. 2017. These cells accumulated in the stomach mucosa starting on day 14 post-infection in the WT mice but not in the

LysMcre mice. We observed a similar increase (*Fig 2b,e and Fig 2c,f*) in the number of resident macrophages as well as monocyte derived macrophages in the simulated WT groups in comparison to the simulated LysMcre group. We estimated the parameter values to fit the data obtained from the mouse model of *H. pylori* infection. Thus, the observations were qualitatively similar to the findings in [24], where the stomach of WT mice was enriched in a population of F4/80+CD11b+CD64+ myeloid cells, compared to LysMCre mice.

Overall, with the results in *Fig 2,* we showed the ability of the hybrid model to replicate the experimental results in [24], and this preliminary data was used as a base calibration setting for *SA* and other *in-silico* findings.

3.2 Partial correlation coefficient analysis screened the influential parameters

To reduce the computational complexity of varying an input parameter space of 38 parameters, we divided the SA process in two stages. For first-stage analysis, we utilized the PRCC regression-based *SA* method to screen the influential inputs and used it for the second stage design of the experiments (refer Methods 2.2). Using PRCC, we determined the impact of the input parameters on the output cell populations in the model. The parameters with significant correlation with *H. pylori* in the gastric lamina propria compartment and resident macrophages are shown in *Fig 3*, along with their PRCC values. The bars in blue, highlight the parameters that are significantly different than 0, at $P < 0.05$ compared to grey bars which are not significant. It is important to note that at this stage the analysis using PRCC was non-temporal.

The *SA* from first stage results showed that the epithelial damage due to infectious bacteria (*epiinfbctdam*) with a coefficient value of (~0.2), was positively correlated with the colonization of *H. pylori* in the lamina propria compartment, indicating the important role of epithelial cell damage during the course of infection, similar to our findings obtained in [43]. Another parameter included the probability of the release of IL-6 (*IL6)* with a coefficient value within the range (0.3-0.4).

Next, the epithelial cell damage parameters (*epiinftbctdam* = (0.2-0.3), *epiTh17dam* = 0-0.2) were shown to have positive influence on the resident macrophage cells whereas, the T cell type transition parameters (*p_iTregtoTh17* = (0.3 - 0.4) and *p_Th17toiTreg* = (0.1 - 0.2)) showed a negative impact on the resident macrophages. Similarly, we performed the PRCC analysis for all the cell populations under consideration during the infection (not shown).

Fig 3. Bar plots for the partial rank correlation coefficients.

The magnitude of the bar-plot indicates the value of the partial rank correlation coefficient. The blue bar indicated the input parameters shown to be significantly different than 0, at P <0.05 as influential whereas the grey bars indicate the non-influential parameters on a) H. pylori and b) resident macrophages, in the lamina propria compartment.

The significant parameters (marked in blue bars) obtained from the SA of the output from first stage design of experiments (152 parameter settings with 20 replicates, refer Methods 2.2), were selected to be varied for the second stage

design. All the selected inputs are shown in Additional file *Fig S4*. In all, we obtained 23 active inputs from the first stage.

3.3 Metamodel based spatio-temporal sensitivity analysis

The outputs obtained after running the first (152 x 20 runs) and second (115 x 20 runs) stage simulations, wherein x20 denotes the 20 replicates, were combined to be used as a training dataset. The combined output was utilized to build a Gaussian process based spatiotemporal metamodel (refer Methods 2.2), using *mlegp* package in R [38].

The outputs from the training dataset were sub-divided into 6 datasets, corresponding to six time periods (Days 1-14, 15-21, 22-30, 31-42, 43-90, 91-201) and averaged across these periods. The sub-division of output across the time periods, aided the temporal analysis over the initiation (Day 1-14), peak of infection (Days 15-30) and chronic phase (post Day 31) stages as in [24]. We then fit a Gaussian process model (with nugget) and evaluated the performance of the fitting of the metamodel for *H. pylori,* resident macrophages, and monocyte-derived macrophages in *lamina propria* compartment, and tolerogenic DC in the gastric lymph node, using the diagnostic plots (see figures in Additional file, Fig *S5*). After fitting the models, we performed variance based global *SA* by computing the Sobol' total order and first order sensitivity index (refer Methods 2.2). The estimates of the Sobol' total order indices for the input parameters calculated over the six time periods are shown in *Fig 4* (a-d).

***Fig 4*. Heat-maps of Sobol' total order index for the input parameters across different output populations.**

The values in the heat-map indicate the Sobol' total order sensitivity index obtained from the metamodel, for the 38 input parameters with respect to the cell populations. The values with darker color indicate a stronger influence on the cell population as compared to the ones with lighter shade that indicate non-influential parameters for the cell populations - a) H. pylori, b) monocyte-derived macrophages, c) resident macrophages, in the lamina propria compartment and d) tolerogenic DCs, in the gastric lymph node compartment. The indexes are calculated over six time points ranging across the three stages of infection, including initiation (Day 1-14), peak (Days 15-42) and recovery stages (Days 43-201).

As shown in *Fig* 4a, the metamodel based global SA showed that the input parameters, epithelial cell proliferation (*Epiprolifer*) and epithelial cell death (*Epicelldeath*) had the strongest impact on the population of *H. pylori* in lamina propria compartment. As time progressed from initiation of the infection (Days 1-14), through peak (Days 15-30), the epithelial cell proliferation had a continued impact on the colonization of *H. pylori*. Next, the influence of the probability of epithelial cell death decreased over the course of infection. Further, *Fig 4b* highlighted the impact of epithelial cell proliferation (*Epiprolifer*) and epithelial cell death (*Epicelldeath*) on the monocyte-derived macrophages.

For the resident macrophage population in the lamina propria, that have emergent properties similar to the one characterized in [24], we observed that the resident macrophage replication parameter (*ResmMacRep*) has an impact during the initiation and peak stages of the infection which indicates that these

subsets of macrophages replicate during the course of *H. pylori* infection. This result highlights the reliability of the two-staged global SA method used here, as these findings are consistent with the ones in [24] wherein we observed that these subsets of macrophages expand in the gastric stomach lamina propria during the course of *H. pylori* infection.

Finally, for the tolerogenic DCs in *Fig 4d*, we observed that the epithelial cell death (*Epicelldeath*) seemed to have an impact. Another parameter that stands for the probability of naive T cell transitioning to iTreg cell (*nTtoiTreg*) was shown to have an impact on the tolerogenic dendritic cells. Tolerogenic dendritic cells are involved in the rule that transitions the naive T cells to iTreg cells in the gastric lymph node, and the stronger impact of the *nTtoiTreg* during the initiation and peak stages of the infection highlights the role of the tolerogenic dendritic cells during the course of infection.

The global *SA* data suggested that the main contributors of the chronic colonization of *H. pylori* in the lamina propria are the epithelial cells, specifically the epithelial cell proliferation parameter.

3.4 Effect of different ranges of epithelial cell proliferation

An interesting prediction derived from the metamodel based global *SA* is that epithelial cell proliferation is one of the parameters that has a strong impact on the size of *H. pylori* population. The biological hypothesis derived from this prediction is that the epithelial cell proliferation is responsible for the higher colonization of *H. pylori*. Prior to conducting any experimental studies, we wanted to explore the hypothesis using our hybrid computer model *in silico* and study the model outputs obtained after we changed the epithelial cell proliferation parameter. Thus, we varied the epithelial cell proliferation parameter across

different ranges (0.1-0.9, with 0.6 being the value for baseline conditions) and ran the simulations using the hybrid model and studied its effect on the different output cell population (obtained after running the simulations). These outputs were the ones obtained after running the simulation using the hybrid computer model, as we varied the epithelial cell proliferation parameter. We analyzed the outputs from the hybrid computer model and interestingly, observed that upon decreasing the *Epiprolifer* from a range of values 0.9-0.1, the output cell populations with regulatory function, namely regulatory macrophages and tolerogenic dendritic cells were found to vary. We observed a decreasing effect (*Fig 5a-d)* on *H. pylori,* monocyte-derived macrophages, resident macrophages in the lamina propria compartment and tolerogenic dendritic cells in gastric lymph node. Overall, these cell populations varied due to the variation in the epithelial cell proliferation parameter.

For clarification, such connection was not embedded in the mechanisms included in Table 1 but it represents an emergent behavior from the simulations predicting the involvement of regulatory and tolerogenic dendritic cells in the mechanisms of immunoregulation during H. pylori infection. Finally, the simulations targeting the epithelial cell proliferation resulted in changes in regulatory and tolerogenic dendritic cell populations. This shows that the simulations indirectly targeted the regulatory and tolerogenic dendritic cell population. Thus, we hypothesize that epithelial cell proliferation might be responsible for the higher colonization of *H. pylori* through an immunoregulatory mechanism that involves regulatory macrophages and tolerogenic cells. This is in line with our own conclusions drawn from a previous paper [24] where we show that the presence of cells with regulatory phenotype favor higher levels of *H. pylori* colonization. The results from the sensitivity analysis presented in this

paper suggest that epithelial proliferation might be a crucial part of the mechanisms by which these regulatory responses are induced and that there is a link between these parameters. The exact biological process however cannot be inferred from the current model and it will be investigated in follow-up in vivo studies.

***Fig 5. In silico* study of the effect of epithelial cell proliferation parameter on the cell populations.**

The plots show the effect of varying epithelial cell proliferation (p_Epiprolifer) parameter (with values 0.1, 0.5, 0.6(WT), and 0.9) on the output cell population of a) H. pylori, b) tolerogenic dendritic cells, c) resident macrophages and d) monocyte-derived macrophages. The parameter has a decreasing effect on the cellular populations under consideration, wherein a decrease in the parameter value, decreases the abundance of the cells over time. *The lower half of the figures (a-d), show the results for statistical comparison between the groups using ANOVA with the post-hoc analysis. The letters ‘a’, ‘b’, ‘c’, and ‘bc’ represent statistically significant differences (P<0.05) between the groups obtained after running the Tukey’s Honestly Significant Difference*.

The *in silico* findings suggested the involvement of regulatory macrophages (both resident as well as monocyte-derived) and tolerogenic DC on the colonization *of H. pylori* in the gastric lamina propria. This highlighted and validated the role of epithelial cell proliferation as one of the main factor affecting *H. pylori* levels in the gastric niche.

4. Discussion

H. *pylori* is the dominant indigenous bacterium of the gastric microbiota. In the majority of individuals, *H. pylori* colonizes the stomach without causing adverse effects, with little to no activation of inflammatory pathways. However, certain members of the population lose immune tolerance to the bacterium thereby contributing to the development of chronic gastric diseases. The immunological mechanisms underlying its ability to persist in a harsh acidic gastric environment and its dual role as a pathogen and beneficial organism remain unknown. A subset of macrophages helps create a regulatory microenvironment that promotes the chronic colonization of *H. pylori* [24]. However, the immune regulatory mechanisms are incompletely understood. Computational models of the immune system featuring immune responses are powerful tools for testing the different 'what-if' scenarios. Multiscale models of the immune response are attractive in terms of modeling the responses at different spatiotemporal scales [44].

In this study, we developed a HPC-driven hybrid, high-resolution, multiscale model to simulate the complex immunoregulatory mechanisms during *H. pylori* infection. The hybrid model was integrated with two intracellular ODEs capturing the dynamics of CD4+ T cells and regulatory macrophages. The inputs to the hybrid model are the set of parameters whose variation governs the immune system dynamics during infection. The obtained outputs were emergent patterns of different cell types, cytokines, and bacterial levels for instance the levels of *H. pylori*, and that qualitatively matched the patterns observed in an *in vivo* infection model [1, 24]. We presented an *in-silico* framework that evaluated the global *SA* of the hybrid model and studied how the variation in the biological parameters affected the simulation

outputs. The two-stage global SA indicated that epithelial cell parameters, specifically, the proliferation of epithelial cells affected the colonization of *H. pylori* in the gastric mucosa. These results were validated *in silico*, and highlighted the involvement of regulatory macrophages and tolerogenic DC in facilitating *H. pylori* colonization of the gastric mucosa. Previous studies highlighted *H. pylori* inhabits the apical surfaces of the epithelial cells and maintains a persistent infection [45].

Further, Mimuro et al. demonstrated that *H. pylori* promotes epithelial gastric cell survival by attenuating apoptosis. These events showed how *H. pylori* regulated the gastric niche and utilized epithelial cells to facilitate its persistence within the stomach [46] [47]. Thus, the findings in the current study are in line with the literature that suggests epithelial cell proliferation favor the colonization of *H. pylori* in the stomach.

Our group also showed another mechanism used by *H. pylori* to create a gut microenvironment that involved the induction of IL-10-driven regulatory mechanism mediated by $CD11b^{+}F4/80^{hi}CD64^{+}CX_{3}CR1^{+}$ mononuclear phagocytes, which facilitated bacterial colonization [24]. Additionally, in this paper, we reported that regulatory macrophages were involved in the process of colonization with *H. pylori* when we varied the epithelial cell proliferation parameter *in-silico*. Zhang et al., demonstrated that *H. pylori* directed active tolerogenic programming of DCs that favored chronic bacterial colonization, by altering the balance of Th17/Treg cells [48]. Rizzuti, Ang et al., demonstrated *H. pylori*-mediated IL-10 release caused the activation of signal transducer and activator of transcription 3 (STAT3) in DC. This activation of STAT3 via IL-10 release was shown to induce the production of tolerogenic DC phenotype [49]. The findings from this paper also indicated the involvement of tolerogenic DCs in affecting the mucosal levels of *H. pylori*. Therefore, the literature combined with the results from this study, collectively

suggest that during *H. pylori* infection, the epithelial cell favors the colonization of *H. pylori* by creating a regulatory microenvironment. This process is mediated by the regulatory macrophages and tolerogenic programming of DC. Based on the results from this paper and findings from the literature, this leads us to propose that the induction of IL-10 by the regulatory macrophages is potentially involved in directing the tolerogenic programming of DC. All experimental evidence combined with our model prediction suggest the action of an underlying biological mechanism that links the presence of *H. pylori* in the gastric mucosa with changes in the rates of epithelial cell proliferation which ultimately affects the levels of colonization. Our prediction points towards a link between epithelial cell proliferation and the action of tolerogenic dendritic cells and regulatory macrophages. The exact cellular mechanism induced during this process however cannot be inferred from the current model and it will be investigated in follow-up in vivo studies.

At its current stage, the hybrid ENISI model reproduces the overall immune system dynamics observed during an *H. pylori* infection. The parameters of calibrated ODEs were kept unchanged, whereas the ABM parameters were calibrated by qualitatively matching the patterns of the output simulations as observed in an *in vivo* model of *H. pylori* infection [24]. For ABM, its calibration and validation remain the major key issues, discussed elsewhere [21] [50] [51]. Further, developing targeted methods of *SA* have been identified as an important challenge in the field [21, 52, 53]. In this paper, we highlighted the use of *SA* methods with a two-stage global *SA* framework comprised of first, screening the input parameters (using PRCC) and second, building of a surrogate model (using GP) of the hybrid model, to understand the emergent behavior of the represented system. It is important to note that each *SA* method known, has its own merits and produces

useful information however none provide a complete picture of the emergent model behavior [21]. First, we employed PRCC methods as the initial step in our two staged *SA* that aided the screening of active inputs and reduced the parameter space. The choice of PRCC was advantageous and justified by the low computational cost and low complexity in the computation of the coefficients. Another advantage of the regression-based PRCC method is that the complex output from our hybrid model was condensed into a descriptive relationship that can be described by statistical measures such as R^2 [21]. As described in [21] the results from PRCC are good descriptors of the outputs produced if the regression function constitutes a good fit to the output [21]. However, if the function does not yield a good fit, the regression-based *SA* are proven to be useful in screening the influential parameters for further analysis [21], as described in our analysis.

Further, the interaction effects between the parameters are not considered in regression-based methods, and hence it was followed by the use of variance-based methods in later stage analysis. Second, we employed metamodeling-based approach and Sobol' method as they provided information on the interaction between the input variable and the use of metamodels allowed to compute the sensitivity indices. One of the advantages of the Sobol' method is that it is model-free and no fitting functions are used to decompose the output variance [31]. It considers the averaged effect of parameters over the whole parameter space but fails to explore the different patterns within the space [21]. Further, the method is not suitable for quantification of output variability if the output distributions deviate from a normal distribution [21]. The detailed comparison of different *SA* methods used for the global *SA* of ABMs are described in detail in [21]. Thus, we performed both the PRCC and computation of Sobol' indices approaches to evaluate the influence of

the input parameter variation and identified the parameters involved in the successful colonization of the gastric niche by *H.* pylori.

Some limitations of the model include implementation through a two-dimensional grid system and including all cells of the same size. Although we parallelize the computation of the hybrid model output, the large number of simulations required for the global *SA* compensates for the benefits of parallelization. To improve the calibration process and overall usability of the model, the data required for model calibration would include tissue biopsies from people infected with *H. pylori* that can be used to quantify the cells and take into account their spatial arrangement. The current version is also limited in terms of the interactions that are based on epithelial cells and DC as they are strictly rule-based. The building of ODE models for these cells and integrating them with the ABM model will help capture the dynamics of epithelial cells and DC more in-depth. Overall the immunoregulatory mechanisms underlying the chronic colonization of *H. pylori* and the predictive capacity of the model can be further improved by incorporating cell-specific models for epithelial cells and DC.

In summary, a high-resolution, hybrid, multiscale spatiotemporal stochastic model of *H. pylori* infection was built and global *SA* was performed. The results from the global *SA* highlight the key role played by epithelial cells in affecting the levels of *H. pylori* colonization. The *in-silico* validation of varying the epithelial cell proliferation parameter demonstrated the involvement of regulatory macrophages and the tolerogenic DC. The next steps aimed to enrich the model will involve the validation of the findings *in vivo* to study the underlying mechanisms involved in the successful immune evasion by *H. pylori*. The computational modeling predictions will be further validated experimentally and clinically.

5. Potential Implications

The computational model of the gut contains high-resolution information processing representations of immune responses that are generalizable for other infectious and autoimmune diseases. Complex diseases such as autoimmune disorders, infectious diseases, and cancer all require integration of the multiscale level data, information and knowledge, ranging from genes, proteins, cells, tissue to organ level. The ENISI model of the gut presented here can be generalized to other diseases by implementing the agents and rules specific to that disease, plus recalibrating the model based on data that are specific to the new indication. Since ABMs have modular architectures, an addition of new agent-types and modification of rules can be done without restructuring the entire simulation setup [19]. The use of ABM in such hybrid models not only facilitates the implementation of already known mechanisms but also helps validate and predict any unforeseen new mechanisms using data analytics methods such as global *SA* to analyze emerging behaviors at the systems level. The finer details regarding intracellular and intercellular interactions that contribute towards the nonlinear and complex behavior of the gut can also be studied by integrating the intracellular ODE models as implemented here.

Tables

Name of Agent	Agent Type	Rules
Helicobacter pylori	*H. pylori*	- Moves across the epithelial cell border if near damaged epithelial layer<br>- Proliferates in the lumen and lamina propria<br>- Dies (removed from the simulation) in lamina propria and in the lumen due to the damage of epithelial cells by Th1 or Th17 cells
Macrophages	Monocyte	- Proliferates in presence the of effector dendritic cells or damaged epithelial cells

		- Proliferates in the lamina propria<br>- Differentiates to regulatory macrophage in based on the output from the Macrophage ODE<br>- Differentiates to inflammatory macrophages in presence of IFN-γ<br>- Dies naturally (removed from the model)
	Resident	- Proliferates in the presence of *H. pylori*<br>- Secretes IL10<br>- Dies naturally<br>- Dies due to Th1 and Tr cells
	Regulatory	- Proliferates and removes bacteria<br>- Dies<br>- Secretes IL10
	Inflammatory	-Proliferates in the presence of damaged epithelial cell<br>-Dies naturally
Dendritics	Immature	-Moves from lamina propria to epithelium compartment and from the epithelium to the lamina propria<br>- Differentiates to tolerogenic dendritic cell in the presence of tolerogenic bacteria, both in epithelium and lamina propria<br>- Differentiates to effector dendritic cell in the presence of *H. pylori*<br>- Proliferates in lamina propria and gastric lymph node<br>- Dies naturally
	Effector	- Moves from lamina propria to gastric lymph node<br>- Moves form epithelium to lamina propria<br>- Secretes IL6 and IL12<br>- Dies naturally
	Tolerogenic	- Moves from lamina propria to gastric lymph node<br>- Moves from epithelium to lamina propria<br>- Secretes TGF-β<br>- Dies naturally
T cells	Naïve	In the presence of effector dendritic cells:<br>- Differentiates to Th1 in the presence of IFN-γ or IL12<br>- Differentiates to Th17 in the presences of IL6 or TGF-β<br>In the presence of tolerogenic dendritic cells:<br>- Differentiates to iTreg in the presence of TGF-β<br>- Differentiates to Tr in the presences of IL10<br>- Dies naturally
	Th1	- Secretes IFN-γ<br>- Moves from gastric lymph node to lamina propria<br>- Proliferates in lamina propria and gastric lymph node<br>- Dies naturally
	Th17	- Secretes IL17<br>- In the presence of tolerogenic dendritic cell, transition to iTreg cells<br>- Moves from gastric lymph node to lamina propria<br>- Proliferates in lamina propria and gastric lymph node<br>- Dies naturally
	iTreg	- Secretes IL10

		- In the presence of tolerogenic dendritic cell, transition to iTreg cells<br>- Moves from gastric lymph node to lamina propria<br>- Proliferates in lamina propria and gastric lymph node<br>- Dies naturally
	Tr	- Secretes IL10<br>- Dies naturally<br>- Proliferates in the lamina propria
Epithelial	Healthy	-Damaged due to infectious bacteria<br>-Damaged due to Th1 and Th17 cells<br>-Proliferates<br>-Secretes IL6 and IL12<br>-Dies naturally
	Damaged	-Transitions to healthy state in the presence of IL10<br>-Dies naturally
Bacteria	Infectious	- Dies due to Th1 or Th17 or inflammatory macrophages or damaged epithelial cells<br>- Dies naturally<br>- Proliferates in the lamina propria
	Tolerogenic	- Moves from lumen to the epithelium in the presence of damaged epithelial cells<br>- Becomes infectious if moves in the lamina propria compartment<br>- Proliferates in lumen and lamina propria<br>- Dies naturally

Table 1. A list of rules for all the agent types implemented in the hybrid model

Additional Files

File S1

Fig S1

Table S1

Fig S2

Fig S3

Fig S4

Fig S5

File S1 – *The detailed instruction to Install ENISI MSM (Step I), Run a simulation (Step II) and Conduct Sensitivity Analysis (Step III) are described.*

Fig S1. Design implementation of the hybrid multiscale model used to simulate Helicobacter pylori infection

The figure shows the class structure used in the ENISI MSM hybrid agent based-ODE model. Each group consists of an act() function that includes the implemented rule for each agent. The previously published ODE models for T cells and Macrophage are used to integrate in the ABM code.

Table S1 *Table describing the input parameters used in the sensitivity analysis and their ranges used.*

Fig S2. A pictorial representation of the spatial discretization of the 2D grid.

Fig S3. Flowchart for the two-staged global sensitivity analysis.

Fig S4. The active and inactive inputs selected from the stage 1 analysis

The rows represent the input parameters and columns represent the output cell populations. The green boxes highlight the 'active' input parameters (row) that are shown to have a significant influence (calculated based on the results obtained from partial correlation coefficient analysis), on an output cell (columns) under consideration.

Fig S5. Diagnostic and residual plots obtained for the Gaussian processes fitted metamodels

The upper panel represents the diagnostic Q-Q plots where the open circles represent the cross-validated predictions; solid black lines represent observed response. The "observed simulations" data in the first half of the lower panel, refer to the observed output values of the simulations obtained after running the hybrid computer model, whereas the y axis refers to the predicted simulation values obtained from the Cross-validated model. Each point represents 1 output point obtained as an output from the simulation. The second half of the lower panel, refers to the standard residual plot wherein the x-axis represents the observed simulation values obtained from the simulation and the y-axis refers to the residual error ((error (predicted values – observed values) / standard deviation (error))) obtained. The diagnostic plots denote the black circles which are the cross-validated prediction. Cross-validation is in the sense that for predictions made at design point x, all observations at design point x are removed from the training set. The lower panel represents the residual plots for the cell populations –(a) Helicobacter pylori; (b) Resident macrophages; (c) Monocyte-derived macrophages in the Lamina propria and (d) Tolerogenic dendritic cells in the Gastric lymph node compartment.

Data and materials

The data sets and files supporting the results of this article are available in the ENISI-MSM GitHub repository, RRID: SCR_016918 https://github.com/NIMML/ENISI-MSM.

Availability of source code and requirements

- Project Name: ENISI MSM
- Project homepage: https://github.com/NIMML/ENISI-MSM
- Operating system(s): Linux, Mac OSX
- Programming language: C++, R, MATLAB
- Other requirements: CMake 3.7.2,
  ENISI Dependencies https://github.com/NIMML/ENISI-Dependencies
- License: Apache License 2.0
- RRID: SCR_016918

Declarations

List of abbreviations

ABM – Agent based model

DC – Dendritic cells

ENISI MSM – Enteric Immunity Simulator Multi-scale Modeling

GLN – gastric lymph node

GP - Gaussian process

H. pylori – Helicobacter pylori

HPC – High performance computing

LP – Lamina propria

ODE – Ordinary Differential Equation

PDE – Partial Differential Equation

SA – Sensitivity analysis

PRCC - Partial rank correlation coefficient

Consent for publication

Not applicable.

Competing interests

The author(s) declare that they have no competing interests.

Funding

This work was supported by the Defense Threat Reduction Agency (DTRA) grant HDTRA1-18-1-0008 to JBR and RH and funds from the Nutritional Immunology and Molecular Medicine Laboratory (www.nimml.org). The funding body had no role in the design of the study, data collection, analysis, interpretation of data and writing of the manuscript.

Authors' contributions

MV, RH and JBR formulated the model, implemented, performed the simulations, analyzed model-generate outputs, made the figures and wrote the manuscript. MV, AL, JBR, RH, and SH formulated the model. SH, AL and VA implemented the code architecture and benchmarked the parallel version of the hybrid model. XC and MV wrote the codes for global sensitivity analysis and generated the design matrices. NTJ generated macrophage and *H. pylori* experimental data. JBR, VA, and RH supervised the project. JBR and RH edited the manuscript. JBR, AL, NTJ, SH, VA, XC and RH participated in discussions on the model and results. All authors provided critical feedback on the project.

Acknowledgements

Not applicable.

References

1. Carbo A, Bassaganya-Riera J, Pedragosa M, Viladomiu M, Marathe M, Eubank S, et al. Predictive computational modeling of the mucosal immune responses during Helicobacter pylori infection. PLoS One. 2013;8(9):e73365. doi: 10.1371/journal.pone.0073365.
2. Leber A, Bassaganya-Riera J, Tubau-Juni N, Zoccoli-Rodriguez V, Viladomiu M, Abedi V, et al. Modeling the Role of Lanthionine Synthetase C-Like 2 (LANCL2) in the Modulation of

Immune Responses to Helicobacter pylori Infection. PloS one. 2016;11(12):e0167440. Epub 2016/12/10. doi: 10.1371/journal.pone.0167440. PubMed PMID: 27936058; PubMed Central PMCID: PMCPMC5147901.

3. Leber A, Viladomiu M, Hontecillas R, Abedi V, Philipson C, Hoops S, et al. Systems Modeling of Interactions between Mucosal Immunity and the Gut Microbiome during Clostridium difficile Infection. PloS one. 2015;10(7):e0134849. Epub 2015/08/01. doi: 10.1371/journal.pone.0134849. PubMed PMID: 26230099; PubMed Central PMCID: PMCPMC4521955.

4. Verma M, Erwin S, Abedi V, Hontecillas R, Hoops S, Leber A, et al. Modeling the Mechanisms by Which HIV-Associated Immunosuppression Influences HPV Persistence at the Oral Mucosa. PloS one. 2017;12(1):e0168133. Epub 2017/01/07. doi: 10.1371/journal.pone.0168133. PubMed PMID: 28060843; PubMed Central PMCID: PMCPMC5218576.

5. Qomlaqi M, Bahrami F, Ajami M, Hajati J. An extended mathematical model of tumor growth and its interaction with the immune system, to be used for developing an optimized immunotherapy treatment protocol. Mathematical biosciences. 2017;292:1-9. Epub 2017/07/18. doi: 10.1016/j.mbs.2017.07.006. PubMed PMID: 28713023.

6. Vodovotz Y, Xia A, Read EL, Bassaganya-Riera J, Hafler DA, Sontag E, et al. Solving Immunology? Trends in immunology. 2017;38(2):116-27. doi: 10.1016/j.it.2016.11.006. PubMed PMID: PMC5695553.

7. Kusters JG, van Vliet AH, Kuipers EJ. Pathogenesis of Helicobacter pylori infection. Clinical microbiology reviews. 2006;19(3):449-90. Epub 2006/07/19. doi: 10.1128/cmr.00054-05. PubMed PMID: 16847081; PubMed Central PMCID: PMCPMC1539101.

8. Mane S, Dominguez-Bello M, Blaser M, Sobral B, Hontecillas R, Skoneczka J, et al. Host-interactive genes in Amerindian Helicobacter pylori diverge from their Old World homologs and mediate inflammatory responses. Journal of bacteriology. 2010;192(12):3078-92.

9. Cover TL, Blaser MJ. Helicobacter pylori in health and disease. Gastroenterology. 2009;136(6):1863-73. doi: 10.1053/j.gastro.2009.01.073. PubMed PMID: PMC3644425.

10. Bassaganya-Riera J, Dominguez-Bello MG, Kronsteiner B, Carbo A, Lu P, Viladomiu M, et al. Helicobacter pylori colonization ameliorates glucose homeostasis in mice through a PPAR γ-dependent mechanism. PLoS One. 2012;7(11):e50069. doi: 10.1371/journal.pone.0050069.

11. Oertli M, Sundquist M, Hitzler I, Engler DB, Arnold IC, Reuter S, et al. DC-derived IL-18 drives Treg differentiation, murine Helicobacter pylori-specific immune tolerance, and asthma protection. The Journal of clinical investigation. 2012;122(3):1082-96. Epub 2012/02/07. doi: 10.1172/jci61029. PubMed PMID: 22307326; PubMed Central PMCID: PMCPMC3287234.

12. Mei Y, Abedi V, Carbo A, Zhang X, Lu P, Philipson C, et al. Multiscale modeling of mucosal immune responses. BMC bioinformatics. 2015;16 Suppl 12:S2. Epub 2015/09/04. doi: 10.1186/1471-2105-16-s12-s2. PubMed PMID: 26329787; PubMed Central PMCID: PMCPMC4705510.

13. Gong C, Milberg O, Wang B, Vicini P, Narwal R, Roskos L, et al. A computational multiscale agent-based model for simulating spatio-temporal tumour immune response to PD1 and PDL1 inhibition. Journal of the Royal Society, Interface. 2017;14(134). Epub 2017/09/22. doi: 10.1098/rsif.2017.0320. PubMed PMID: 28931635; PubMed Central PMCID: PMCPMC5636269.

14. Wang Z, Birch CM, Sagotsky J, Deisboeck TS. Cross-scale, cross-pathway evaluation using an agent-based non-small cell lung cancer model. Bioinformatics (Oxford, England). 2009;25(18):2389-96. Epub 2009/07/07. doi: 10.1093/bioinformatics/btp416. PubMed PMID: 19578172; PubMed Central PMCID: PMCPMC2735669.

15. Marino S, El-Kebir M, Kirschner D. A hybrid multi-compartment model of granuloma formation and T cell priming in tuberculosis. Journal of theoretical biology. 2011;280(1):50-62. Epub 2011/03/30. doi: 10.1016/j.jtbi.2011.03.022. PubMed PMID: 21443879; PubMed Central PMCID: PMCPMC3740747.

16. Solovyev A, Mi Q, Tzen YT, Brienza D, Vodovotz Y. Hybrid equation/agent-based model of ischemia-induced hyperemia and pressure ulcer formation predicts greater propensity to ulcerate in subjects with spinal cord injury. PLoS computational biology. 2013;9(5):e1003070. Epub 2013/05/23. doi: 10.1371/journal.pcbi.1003070. PubMed PMID: 23696726; PubMed Central PMCID: PMCPMC3656105.

17. Cappuccio A, Tieri P, Castiglione F. Multiscale modelling in immunology: a review. Briefings in bioinformatics. 2016;17(3):408-18. Epub 2015/03/27. doi: 10.1093/bib/bbv012. PubMed PMID: 25810307.

18. Bassaganya-Riera J. Computational Immunology: Models and Tools: Academic Press; 2015.

19. An G, Mi Q, Dutta-Moscato J, Vodovotz Y. Agent-based models in translational systems biology. Wiley interdisciplinary reviews Systems biology and medicine. 2009;1(2):159-71. Epub 2010/09/14. doi: 10.1002/wsbm.45. PubMed PMID: 20835989; PubMed Central PMCID: PMCPMC3640333.

20. Abedi V, Hontecillas R, Hoops S, Liles N, Carbo A, Lu P, et al., editors. ENISI multiscale modeling of mucosal immune responses driven by high performance computing. 2015 IEEE International Conference on Bioinformatics and Biomedicine (BIBM); 2015 9-12 Nov. 2015.

21. Ten Broeke G, Van Voorn G, Ligtenberg A. Which sensitivity analysis method should I use for my agent-based model? Journal of Artificial Societies and Social Simulation. 2016;19(1):5.

22. Ligmann-Zielinska A, Kramer DB, Spence Cheruvelil K, Soranno PA. Using uncertainty and sensitivity analyses in socioecological agent-based models to improve their analytical performance and policy relevance. PLoS One. 2014;9(10):e109779. Epub 2014/10/24. doi: 10.1371/journal.pone.0109779. PubMed PMID: 25340764; PubMed Central PMCID: PMCPMC4207681.

23. Carbo A, Hontecillas R, Kronsteiner B, Viladomiu M, Pedragosa M, Lu P, et al. Systems modeling of molecular mechanisms controlling cytokine-driven CD4+ T cell differentiation and

phenotype plasticity. PLoS computational biology. 2013;9(4):e1003027. Epub 2013/04/18. doi: 10.1371/journal.pcbi.1003027. PubMed PMID: 23592971; PubMed Central PMCID: PMCPMC3617204.

24. Viladomiu M, Bassaganya-Riera J, Tubau-Juni N, Kronsteiner B, Leber A, Philipson CW, et al. Cooperation of Gastric Mononuclear Phagocytes with Helicobacter pylori during Colonization. Journal of immunology (Baltimore, Md : 1950). 2017;198(8):3195-204. Epub 2017/03/08. doi: 10.4049/jimmunol.1601902. PubMed PMID: 28264969; PubMed Central PMCID: PMCPMC5380565.

25. Collier N, North M. Repast HPC: A platform for large-scale agentbased modeling. Large-Scale Computing Techniques for Complex System Simulations. 2011:81-110.

26. Hoops S, Sahle S, Gauges R, Lee C, Pahle J, Simus N, et al. COPASI—a complex pathway simulator. Bioinformatics (Oxford, England). 2006;22(24):3067-74.

27. Saltelli A, Tarantola S, Campolongo F. Sensitivity anaysis as an ingredient of modeling. Statistical Science. 2000;15(4):377-95.

28. Thorne BC, Bailey AM, Peirce SM. Combining experiments with multi-cell agent-based modeling to study biological tissue patterning. Briefings in Bioinformatics. 2007;8(4):245-57. doi: 10.1093/bib/bbm024.

29. Moon H, Dean AM, Santner TJ. Two-stage sensitivity-based group screening in computer experiments. Technometrics. 2012;54(4):376-87.

30. Marino S, Hogue IB, Ray CJ, Kirschner DE. A methodology for performing global uncertainty and sensitivity analysis in systems biology. Journal of theoretical biology. 2008;254(1):178-96. Epub 2008/06/24. doi: 10.1016/j.jtbi.2008.04.011. PubMed PMID: 18572196; PubMed Central PMCID: PMCPMC2570191.

31. Saltelli A, Ratto M, Andres T, Campolongo F, Cariboni J, Gatelli D, et al. Global sensitivity analysis: the primer: John Wiley & Sons; 2008.

32. Rasmussen CE, Williams CK. Gaussian processes for machine learning. 2006. The MIT Press, Cambridge, MA, USA. 2006;38:715-9.

33. Santner TJ, Williams BJ, Notz WI. The design and analysis of computer experiments: Springer Science & Business Media; 2013.

34. Thiele JC, Kurth W, Grimm V. Facilitating parameter estimation and sensitivity analysis of agent-based models: A cookbook using NetLogo and R. Journal of Artificial Societies and Social Simulation. 2014;17(3):11.

35. Ankenman B, Nelson BL, Staum J. Stochastic kriging for simulation metamodeling. Operations research. 2010;58(2):371-82.

36. Lamoureux B, Mechbal N, Massé J-R. A combined sensitivity analysis and kriging surrogate modeling for early validation of health indicators. Reliability Engineering & System Safety. 2014;130:12-26.

37. Chen X, Kim K-K. Stochastic kriging with biased sample estimates. ACM Trans Model Comput Simul. 2014;24(2):1-23. doi: 10.1145/2567893.

38. Dancik GM, Dorman KS. mlegp: statistical analysis for computer models of biological systems using R. Bioinformatics (Oxford, England). 2008;24(17):1966-7.

39. Saltelli A, Annoni P, Azzini I, Campolongo F, Ratto M, Tarantola S. Variance based sensitivity analysis of model output. Design and estimator for the total sensitivity index. Computer Physics Communications. 2010;181(2):259-70.

40. Sobol IM. Sensitivity estimates for nonlinear mathematical models. Mathematical modelling and computational experiments. 1993;1(4):407-14.

41. Sobol' IM, Tarantola S, Gatelli D, Kucherenko SS, Mauntz W. Estimating the approximation error when fixing unessential factors in global sensitivity analysis. Reliability Engineering & System Safety. 2007;92(7):957-60. doi: https://doi.org/10.1016/j.ress.2006.07.001.

42. Jansen MJ. Analysis of variance designs for model output. Computer Physics Communications. 1999;117(1-2):35-43.

43. Alam M, Deng X, Philipson C, Bassaganya-Riera J, Bisset K, Carbo A, et al. Sensitivity Analysis of an ENteric Immunity SImulator (ENISI)-Based Model of Immune Responses to Helicobacter pylori Infection. PloS one. 2015;10(9):e0136139. Epub 2015/09/04. doi: 10.1371/journal.pone.0136139. PubMed PMID: 26327290; PubMed Central PMCID: PMCPMC4556515.

44. Heiner M, Gilbert D. BioModel engineering for multiscale Systems Biology. Progress in Biophysics and Molecular Biology. 2013;111(2):119-28. doi: https://doi.org/10.1016/j.pbiomolbio.2012.10.001.

45. Alzahrani S, Lina TT, Gonzalez J, Pinchuk IV, Beswick EJ, Reyes VE. Effect of Helicobacter pylori on gastric epithelial cells. World Journal of Gastroenterology : WJG. 2014;20(36):12767-80. doi: 10.3748/wjg.v20.i36.12767. PubMed PMID: PMC4177462.

46. Mimuro H, Suzuki T, Nagai S, Rieder G, Suzuki M, Nagai T, et al. Helicobacter pylori dampens gut epithelial self-renewal by inhibiting apoptosis, a bacterial strategy to enhance colonization of the stomach. Cell host & microbe. 2007;2(4):250-63. Epub 2007/11/17. doi: 10.1016/j.chom.2007.09.005. PubMed PMID: 18005743.

47. Wroblewski LE, Peek RM. Orchestration of Dysregulated Epithelial Turnover by a Manipulative Pathogen. Cell Host & Microbe. 2007;2(4):209-11. doi: https://doi.org/10.1016/j.chom.2007.09.011.

48. Zhang M, Liu M, Luther J, Kao JY. Helicobacter pylori directs tolerogenic programming of dendritic cells. Gut microbes. 2010;1(5):325-9. Epub 2011/02/18. doi: 10.4161/gmic.1.5.13052. PubMed PMID: 21327041; PubMed Central PMCID: PMCPMC3023617.

49. Rizzuti D, Ang M, Sokollik C, Wu T, Abdullah M, Greenfield L, et al. Helicobacter pylori inhibits dendritic cell maturation via interleukin-10-mediated activation of the signal transducer and activator of transcription 3 pathway. Journal of innate immunity. 2015;7(2):199-211. Epub 2014/11/22. doi: 10.1159/000368232. PubMed PMID: 25412627.

50. Windrum P, Fagiolo G, Moneta A. Empirical validation of agent-based models: Alternatives and prospects. Journal of Artificial Societies and Social Simulation. 2007;10(2):8.

51. Fagiolo G, Moneta A, Windrum P. A critical guide to empirical validation of agent-based models in economics: Methodologies, procedures, and open problems. Computational Economics. 2007;30(3):195-226.

52. Crooks A, Castle C, Batty M. Key challenges in agent-based modelling for geo-spatial simulation. Computers, Environment and Urban Systems. 2008;32(6):417-30.

53. Filatova T, Verburg PH, Parker DC, Stannard CA. Spatial agent-based models for socio-ecological systems: Challenges and prospects. Environmental modelling & software. 2013;45:1-7.

Click here to access/download;Table;Table_1_Revised.docx

Name of Agent	Agent Type	Rules
Helicobacter pylori	*H. pylori*	- Moves across the epithelial cell border if near damaged epithelial layer<br>- Proliferates in the lumen and lamina propria<br>- Dies (removed from the simulation) in lamina propria and in the lumen due to the damage of epithelial cells by Th1 or Th17 cells
Macrophages	Monocyte	- Proliferates in presence the of effector dendritic cells or damaged epithelial cells<br>- Proliferates in the lamina propria<br>- Differentiates to regulatory macrophage in based on the output from the Macrophage ODE<br>- Differentiates to inflammatory macrophages in presence of IFN-γ<br>- Dies naturally (removed from the model)
	Resident	- Proliferates in the presence of *H. pylori*<br>- Secretes IL10<br>- Dies naturally<br>- Dies due to Th1 and Tr cells
	Regulatory	- Proliferates and removes bacteria<br>- Dies<br>- Secretes IL10
	Inflammatory	-Proliferates in the presence of damaged epithelial cell<br>-Dies naturally
Dendritics	Immature	-Moves from lamina propria to epithelium compartment and from the epithelium to the lamina propria<br>- Differentiates to tolerogenic dendritic cell in the presence of tolerogenic bacteria, both in epithelium and lamina propria<br>- Differentiates to effector dendritic cell in the presence of *H. pylori*<br>- Proliferates in lamina propria and gastric lymph node<br>- Dies naturally
	Effector	- Moves from lamina propria to gastric lymph node<br>- Moves form epithelium to lamina propria<br>- Secretes IL6 and IL12<br>- Dies naturally
	Tolerogenic	- Moves from lamina propria to gastric lymph node<br>- Moves from epithelium to lamina propria<br>- Secretes TGF-β<br>- Dies naturally
T cells	Naïve	In the presence of effector dendritic cells:<br>- Differentiates to Th1 in the presence of IFN-γ or IL12<br>- Differentiates to Th17 in the presences of IL6 or TGF-β<br>In the presence of tolerogenic dendritic cells:<br>- Differentiates to iTreg in the presence of TGF-β

		- Differentiates to Tr in the presences of IL10<br>- Dies naturally
	Th1	- Secretes IFN-γ<br>- Moves from gastric lymph node to lamina propria<br>- Proliferates in lamina propria and gastric lymph node<br>- Dies naturally
	Th17	- Secretes IL17<br>- in the presence of tolerogenic dendritic cell, transition to iTreg cells<br>- Moves from gastric lymph node to lamina propria<br>- Proliferates in lamina propria and gastric lymph node<br>- Dies naturally
	iTreg	- Secretes IL10<br>- In the presence of tolerogenic dendritic cell, transition to iTreg cells<br>- Moves from gastric lymph node to lamina propria<br>- Proliferates in lamina propria and gastric lymph node<br>- Dies naturally
	Tr	- Secretes IL10<br>- Dies naturally<br>- Proliferates in the lamina propria
Epithelial	Healthy	-Damaged due to infectious bacteria<br>-Damaged due to Th1 and Th17 cells<br>-Proliferates<br>-Secretes IL6 and IL12<br>-Dies naturally
	Damaged	-Transitions to healthy state in the presence of IL10<br>-Dies naturally
Bacteria	Infectious	- Dies due to Th1 or Th17 or inflammatory macrophages or damaged epithelial cells<br>- Dies naturally<br>- Proliferates in the lamina propria
	Tolerogenic	- Moves from lumen to the epithelium in the presence of damaged epithelial cells<br>- Becomes infectious if moves in the lamina propria compartment<br>- Proliferates in lumen and lamina propria<br>- Dies naturally

Figure 1

Click here to access/download;Figure;Fig.1.pdf

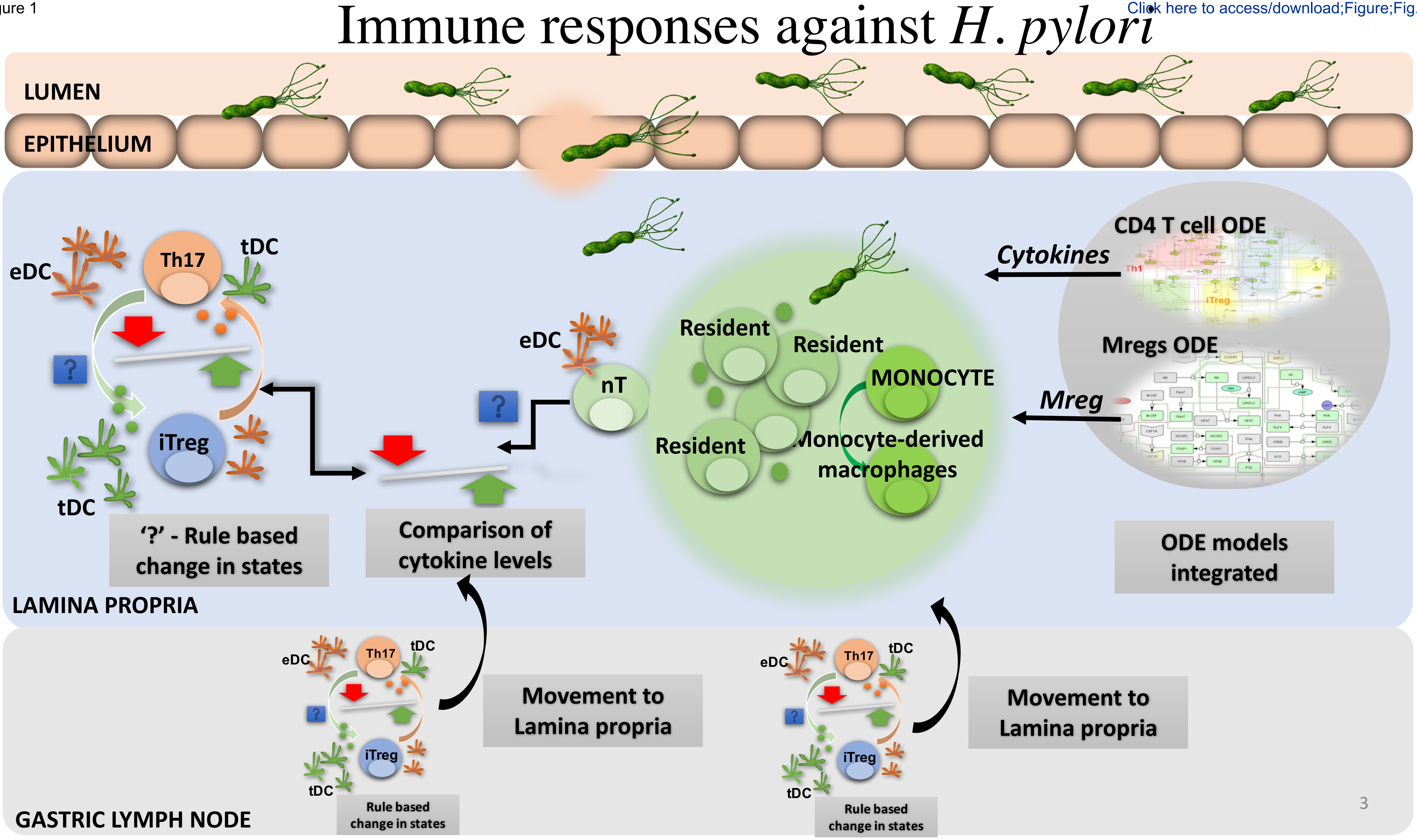

Click here to access/download;Figure;Fig2_Revised.pdf

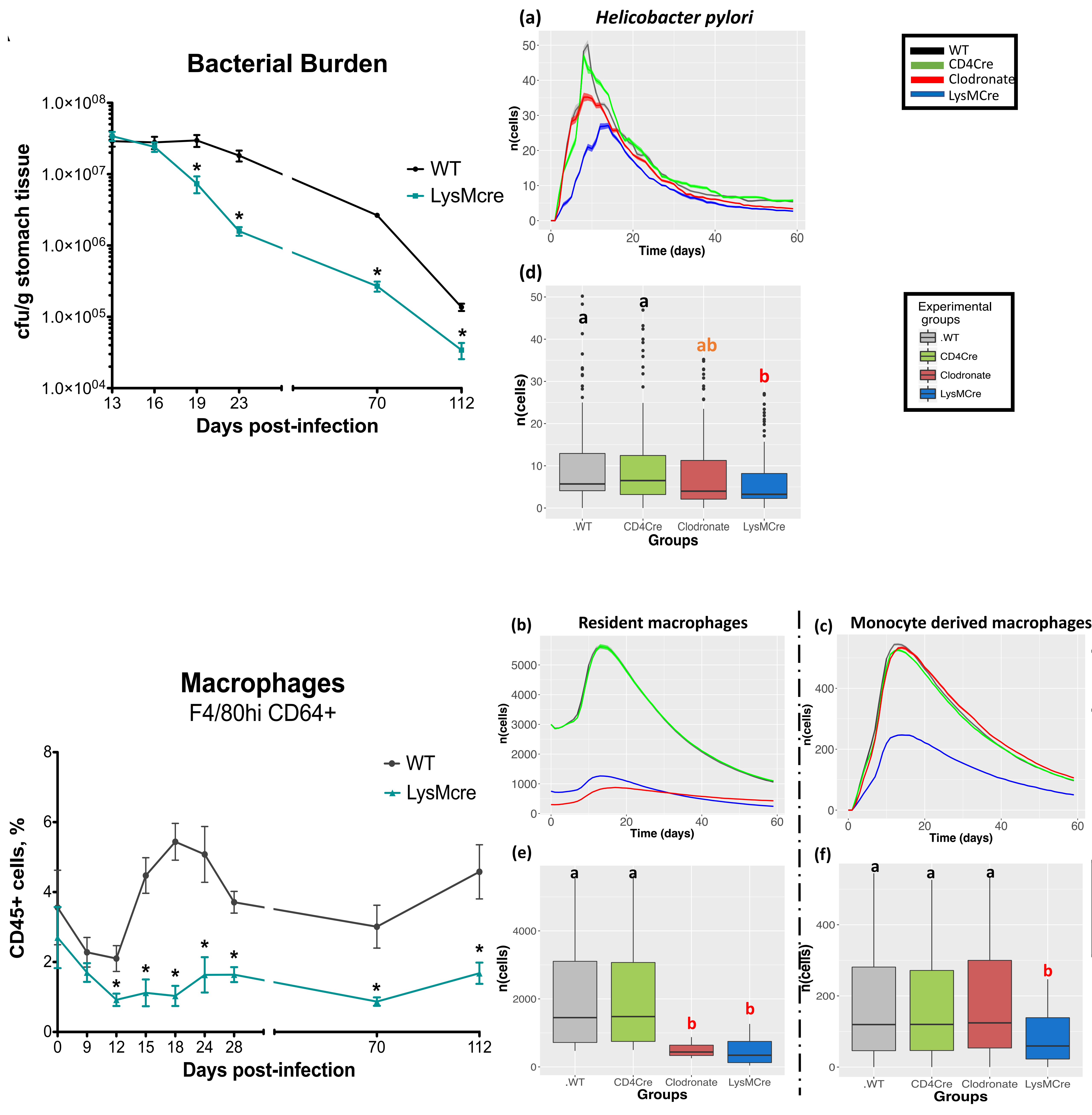

Figure 3

Click here to access/download;Figure;Fig3.pdf

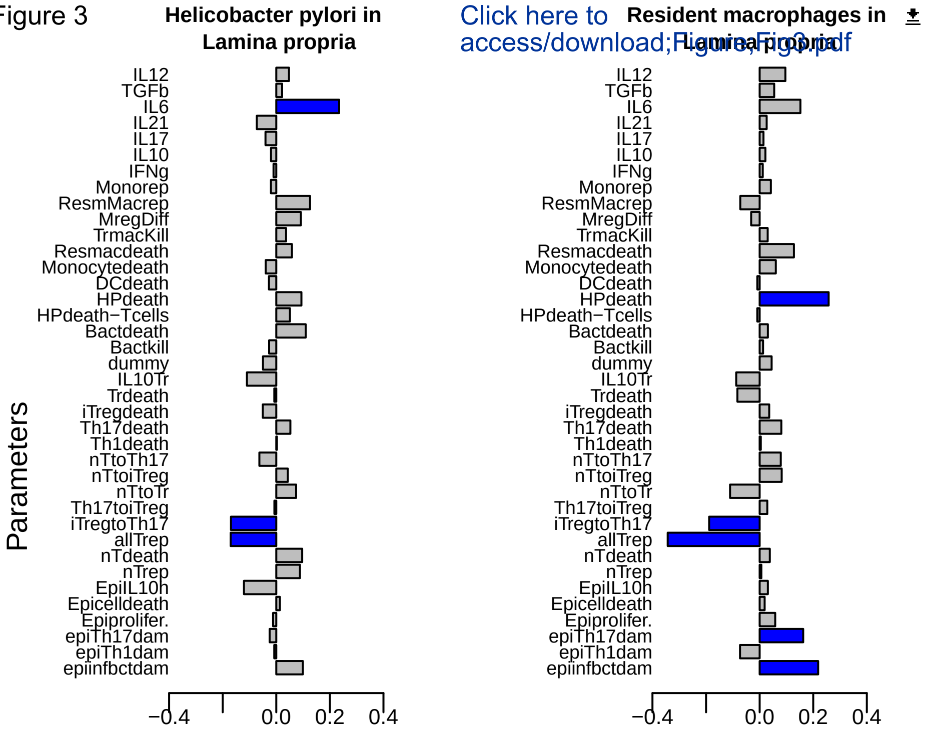

Figure 4

Click here to access/download;Figure;Fig.4.pdf

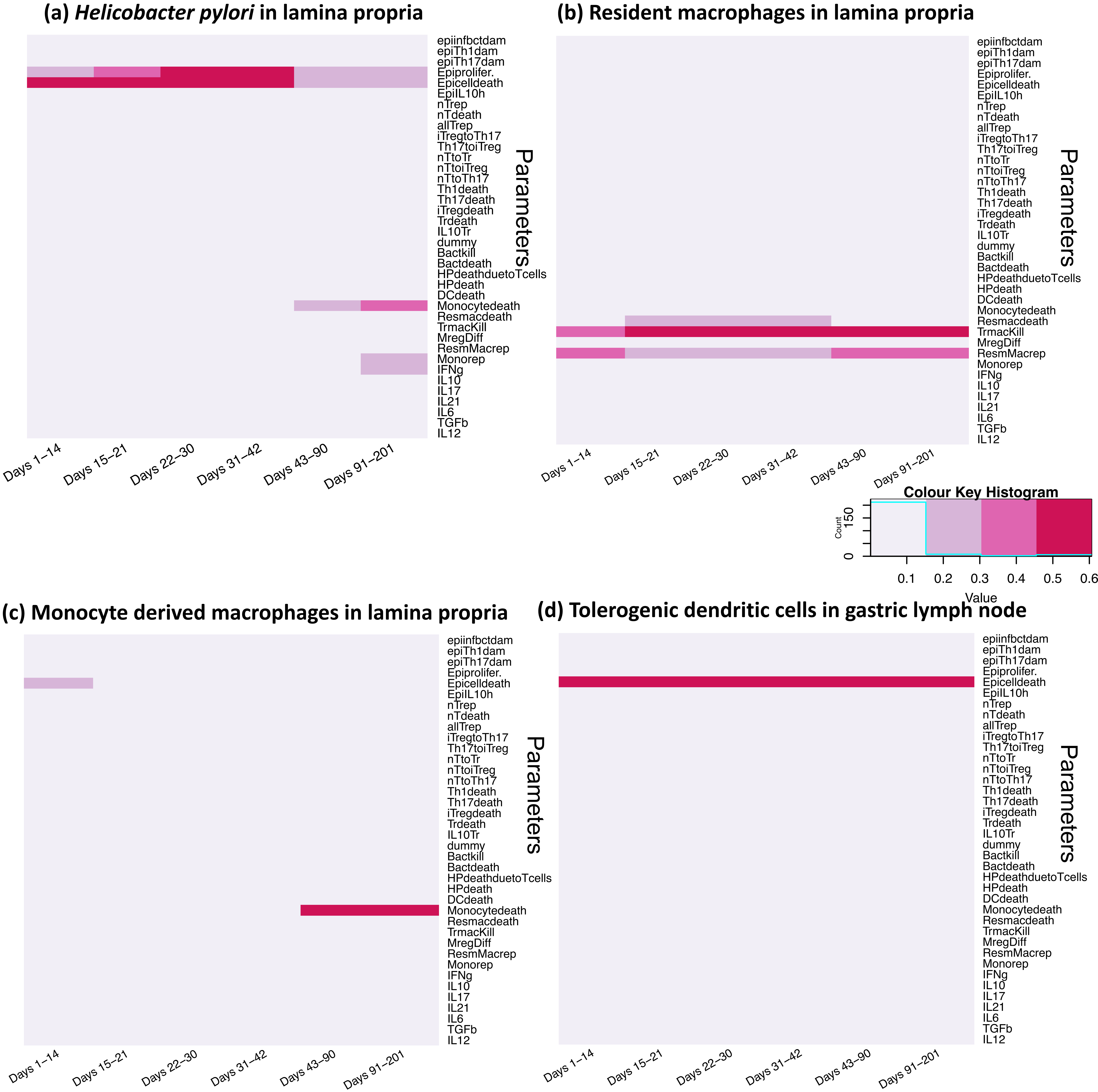

Figure 5

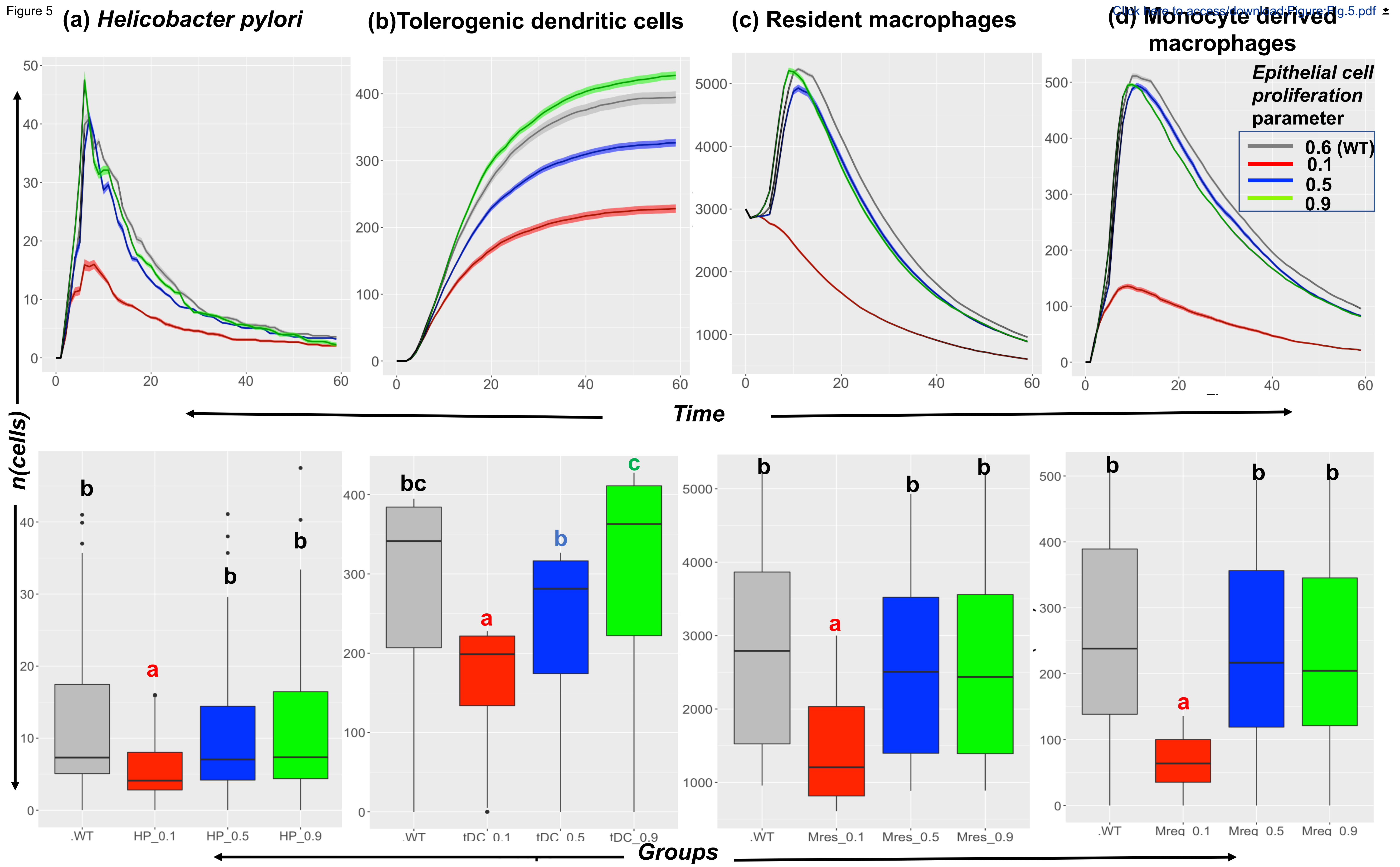
(a) Helicobacter pylori
(b)Tolerogenic dendritic cells
(c) Resident macrophages
(d) Monocyte derived macrophages
Epithelial cell proliferation parameter
0.6 (WT)
0.1
0.5
0.9
n(cells)
Time
Groups
.WT
HP_0.1
HP_0.5
HP_0.9
tDC_0.1
tDC_0.5
tDC_0.9
Mres_0.1
Mres_0.5
Mres_0.9
Mreg_0.1
Mreg_0.5
Mreg_0.9
b
a
b
b
bc
a
b
c
b
a
b
b
b
a
b
b

Click here to access/download
Supplementary Material
FileS1.pdf

Click here to access/download
Supplementary Material
FigS1.png

Click here to access/download
Supplementary Material
Table_S1_revised.docx

Click here to access/download
Supplementary Material
FigS2_revised.pdf

Click here to access/download
Supplementary Material
FigS3.pdf

Click here to access/download
Supplementary Material
FigS4.docx

Click here to access/download
Supplementary Material
FigS5.pdf

Personal Cover

Click here to access/download;Personal Cover;Point_by_point.pdf

Point by point response to the Reviewer reports

We would like to thank the reviewers and editors for taking time to review our manuscript entitled "*High-Resolution Computational Modeling of Immune Responses in the Gut*" and for providing valuable and constructive criticism. The review process has been helpful in the improvement of our submission. We have considered the comments that were made and have prepared the following point-by-point response. We hope that the revised version of the manuscript can now be accepted for publication. Thanks in advance.

Reviewer #1: The unit of sizes of the model grid can't be right (e.g. grid is 30nm x 10 nm). Animal cells should have measurements in the order of micrometres instead of nanometres. Please check if these are just typos, or do these errors affect any aspect of the simulation, such as diffusion.
Response: *We thank the reviewer for pointing this out. We fixed the typos and the unit size of the model grid are 30 µm x 10 µm. These typos do not affect any aspect of the simulations as these units are only annotations and the model takes the numbers as input. We updated the manuscript and fixed the typos throughout the manuscript. Please refer to L120 – L121, and L216, L220-L221.*

Reviewer #2: The authors have made significant improvements to the manuscript and thoroughly responded to reviewer comments. One major concern remains surrounding the authors' response to questions around the grid dimensions. The dimensions for the entire grid are given in nm which is smaller than a single cell. Furthermore they state that there are no limits to cell(agent) occupancy per grid compartment. This is rather confusing and calls into question how much spatial information is really contained in this model (e.g. if cytokines are diffusing over the 30nm grid what does that mean for the concentrations that individual cells (measured in micrometers)are seeing?). Based on the author responses it appears that the model is a multi-compartment model with well-mixed discrete agents in each compartment rather than a spatio-temporal model as they claim.
Response: *We thank the reviewer for their comment.*
We thank the reviewer for pointing out the concern regarding the dimensions of the grid. The correct dimensions of the grid are 30 µm x 10 µm. We updated the manuscript and fixed the typos. Please refer to L120 – L121, and L216, L220-L221.
The mention regarding no limits to cell (agent) occupancy refers to the cells (agents) having no physical size. Further, once a cell (agent) dies it is removed from the simulation to minimize the computational costs of agents that do not contribute to the biology.
The model output contains information about the x and y co-ordinate of the agents at every time point. The cytokines and internal signaling pathways that drive functional fates of cells are well mixed within a cell, i.e., we have only temporal resolution within the cell during a time step. However, the production, degradation, and diffusions are cell specific thus the cytokine concentration results are also spatio-temporal.
Since, the model is capable of providing information regarding spatial co-ordinates over time, we claim the model to be a spatio-temporal model. We updated the manuscript, please refer to L163-L170.

Please also ensure that your revised manuscript conforms to the journal style, which can be found in the Instructions for Authors on the journal homepage.
Response: *The revised manuscript conforms to the journal style.*
