# Supplementary material for: High-resolution computational modeling of immune responses in the gut
Source: Gigascience. 2019 Jun 11;8(6):giz062. doi: 10.1093/gigascience/giz062 (PMC6559340; doi:10.1093/gigascience/giz062)
Supplement: giz062_Supplement_Files [file giz062_supplement_files.zip › FigS4.docx]

| **Cell Population**  **(Columns)**  **Name of parameters (Rows)** | Resident macrophages - LP | Monocyte derived macrophages- LP | Inflammatory macrophages -LP | Helicobacter pylori -LP | Th1-LP | Th17-LP | iTreg-LP | Tr-LP | Th1-GLN | Th17-GLN | iTreg-GLN | Effector dendritic cells -GLN | Tolerogenic dendritic cells - GLN | Number of active inputs | Present at least once |
| --- | --- | --- | --- | --- | --- | --- | --- | --- | --- | --- | --- | --- | --- | --- | --- |
| p_epiinfbactdamage | 1 | 1 | 1 |  |  |  |  | 1 | 1 |  |  |  |  | 5 | 1 |
| p_epith1damage |  |  |  |  |  |  |  |  |  |  |  |  |  | 0 | 0 |
| p_epith17damage | 1 |  | 1 |  |  |  | 1 |  |  |  |  |  |  | 3 | 1 |
| p_EpiProliferation |  |  |  |  |  |  |  | 1 |  |  |  |  |  | 1 | 1 |
| p_EpiCellDeath |  |  |  |  | 1 |  | 1 |  | 1 | 1 | 1 |  |  | 5 | 1 |
| p_epiIL10h |  |  | 1 |  |  |  |  |  |  | 1 |  |  |  | 2 | 1 |
| p_nTrep |  |  |  |  |  |  |  |  |  |  |  |  |  | 0 | 0 |
| p_naiveTcelldeath |  |  |  |  |  |  |  | 1 |  |  |  |  |  | 1 | 1 |
| p_allTrep | 1 |  |  | 1 |  |  |  | 1 |  |  |  |  |  | 3 | 1 |
| p_iTregtoTh17 | 1 |  |  | 1 | 1 | 1 | 1 |  | 1 |  | 1 |  | 1 | 8 | 1 |
| p_Th17toiTreg |  |  |  |  |  |  |  |  |  |  |  |  |  | 0 | 0 |
| p_nTtoTr |  |  |  |  |  |  |  |  |  |  |  |  | 1 | 1 | 1 |
| p_nTtoiTreg |  |  |  |  |  |  |  |  |  |  |  |  |  | 0 | 0 |
| p_nTtoTh17 |  |  |  |  |  |  |  |  |  |  |  |  |  | 0 | 0 |
| p_Th1death |  |  |  |  |  |  |  |  |  |  |  |  |  | 0 | 0 |
| p_Th17death |  |  |  |  |  |  |  |  |  |  |  |  |  | 0 | 0 |
| p_iTregdeath |  |  |  |  |  |  |  |  | 1 | 1 |  |  |  | 2 | 1 |
| p_Trdeath |  |  |  |  |  |  |  |  |  |  |  |  |  | 0 | 0 |
| p_IL10Tr |  |  |  |  |  |  |  |  |  |  |  |  |  | 1 | 1 |
| dummy |  |  |  |  |  |  |  |  |  |  |  |  |  | 0 | 0 |
| p_BacteriaKill |  |  |  |  |  |  |  |  |  | 1 |  |  |  | 1 | 1 |
| p_BacteriaDeath |  |  |  |  |  |  |  |  |  |  |  |  |  | 0 | 0 |
| p_HPdeathduetoTcells |  |  |  |  |  |  |  |  |  |  |  |  |  | 0 | 0 |
| p_HPyloriDeath | 1 |  |  |  |  |  |  |  |  |  |  |  |  | 1 | 1 |
| p_DCDeath |  |  |  |  |  | 1 |  |  |  |  |  |  |  | 1 | 1 |
| p_Monocytedeath |  |  |  |  |  |  |  |  |  |  |  |  |  | 0 | 0 |
| p_resmacdeath |  |  |  |  |  |  |  |  |  | 1 |  |  |  | 1 | 1 |
| p_Trmackill |  |  |  |  |  |  |  |  |  |  |  |  |  | 0 | 0 |
| p_MregDiff |  |  |  |  |  |  |  |  |  |  |  |  |  | 0 | 0 |
| p_resmacrep |  |  |  |  |  |  | 1 |  |  |  |  |  |  | 1 | 1 |
| p_monorep |  |  |  |  |  | 1 | 1 |  |  | 1 |  | 1 |  | 4 | 1 |
| p_IFNg |  |  |  |  |  |  |  |  |  |  |  |  |  | 0 | 1* |
| p_IL10 |  |  |  |  |  |  |  |  |  |  |  |  |  | 0 | 1* |
| p_IL17 |  | 1 |  |  |  |  |  |  |  |  |  |  |  | 1 | 1 |
| p_IL21 |  |  |  |  |  |  |  |  |  |  |  |  |  | 0 | 0 |
| p_IL6 |  | 1 | 1 | 1 |  |  | 1 | 1 |  |  |  |  | 1 | 6 | 1 |
| p_TGFb |  |  |  |  |  | 1 |  |  |  |  |  |  |  | 1 | 1 |
| p_IL12 |  |  |  |  |  |  | 1 |  |  |  |  |  |  | 1 | 1 |
|  |  |  |  |  |  |  |  |  |  |  |  |  |  | Sum(active inputs present at least once) | =23 |
| 1 – Active input  Empty – Inactive input |  |  |  |  |  |  |  |  |  |  |  |  |  |  |  |
| ***** Recommendation from experts to include |  |  |  |  |  |  |  |  |  |  |  |  |  |  |  |
