# Supplementary material for: High-resolution computational modeling of immune responses in the gut
Source: Gigascience. 2019 Jun 11;8(6):giz062. doi: 10.1093/gigascience/giz062 (PMC6559340; doi:10.1093/gigascience/giz062)
Supplement: giz062_Supplement_Files [file giz062_supplement_files.zip › FigS5.pdf]

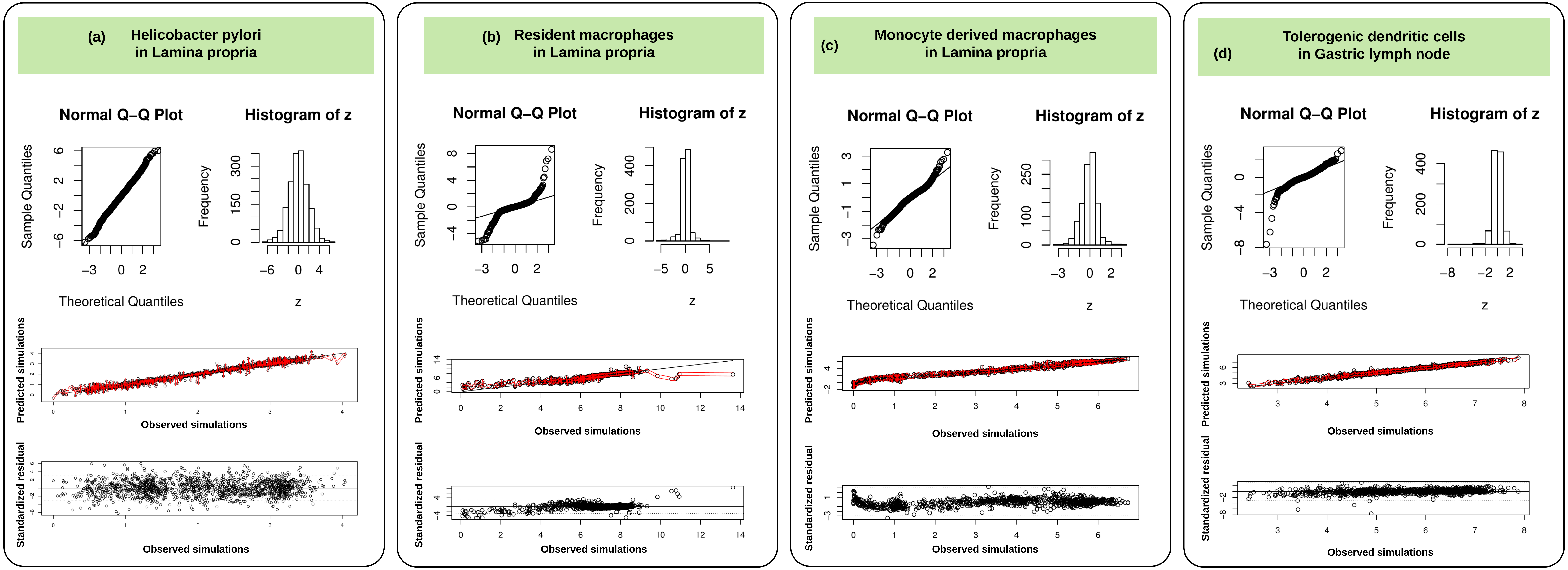

(a) Helicobacter pylori in Lamina propria
(b) Resident macrophages in Lamina propria
(c) Monocyte derived macrophages in Lamina propria
(d) Tolerogenic dendritic cells in Gastric lymph node
Normal Q–Q Plot
Histogram of z
Sample Quantiles
Theoretical Quantiles
Frequency
z
Predicted simulations
Standardized residual
Observed simulations
