# Supplementary material for: High-resolution computational modeling of immune responses in the gut
Source: Gigascience. 2019 Jun 11;8(6):giz062. doi: 10.1093/gigascience/giz062 (PMC6559340; doi:10.1093/gigascience/giz062)
Supplement: giz062_Supplement_Files [file giz062_supplement_files.zip › FileS1.pdf]

This file contains the detailed instruction to **Install** ENISI MSM (Step I), **Run** a simulation (Step II) and **Conduct Sensitivity Analysis** (Step III). The jupyter (.ipynb) notebooks (***Fig2-Code.ipynb***, ***Fig3-Code.ipynb***, ***Fig4-Code.ipynb*** and ***Fig5-Code.ipynb***) include detailed instructions on how to create the specific figures presented in the paper.

A. How to install ENISI MSM

1. Create a folder for the hybrid computer model: mkdir *ENISI*
2. Change directory to the newly created folder: cd *ENISI*
3. Clone the dependencies required from the ENISI-Dependencies from the NIMML GitHub repository -
   i. git clone --recursive https://github.com/NIMML/ENISI-Dependencies
4. Change the path to the ENISI-Dependencies folder: cd *ENISI-Dependencies*
5. Create a directory build within the folder: mkdir *build*
6. Change directory to the directory created in step 5: cd *build*
7. Start the installation: cmake ../
   make
8. Change the directory cd
9. Change the directory to the one created in step 1: cd *ENISI*
10. Clone the ENISI-MSM model from the NIMML GitHub repository –
    i. git clone —recursive https://github.com/NIMML/ENISI-MSM
11. Change the directory to ENISI-MSM: cd *ENISI-MSM*
12. Create a directory build within the folder: mkdir *build*
13. Change the directory to the directory created in step 12: cd *build*
14. Start the installation:
    cmake *-DENISI_MSM_DEPENDENCY_DIR=PATH TO ENISI-Dependencies FOLDER/install* **..**
    make

B. How to run a simulation

1. Create a folder *FolderName* to save the simulation results. It is important to place all the results of every experiment and its respective files in different folders.
2. Place the files i) *config.props* ii) *run.props* iii) *job.sh* (***required only*** if running on cluster) iv) *CD4.cps* v) *MregDiff.cps* vi) *model.props* all in the folder where you want the output files to be saved (*i.e FolderName)*.
3. *model.props* is the parameter file wherein you can change the parameters.
4. *run.props* and *config.props* are the configurable files where you can change the number of TICKS (that is a measure of computational time, *i.e* stop.at = number of TICKS) and the size of the grid (in the current model that is set to 1nm).
5. For *running locally*, use *run.sh*
6. To run on a cluster, use *job.sh.*
7. For the -output folder path, change the ***CONFIG*** variable and provide path to your folder *i.e* /home/username/*FolderName*.
8. ENISI executable to be used in the job.sh file is located in /PATH: *ENISI/ENISI-MSM/bin* folder that is created in the (installation step, Section A).

9. Run your job by typing -> *sh run.sh* (**OR**) *./ run.sh* "path of the folder where you want the results or ***sh job.sh*** (specify the ***CONFIG*** variable within).
10. After the job iscompleted, you will have *.log* files, *.tsv* files for all the compartments.
11. The *.log* file will contain debugging statements if there are any issued in the code. Additional statments can be added to the source code for confirmation and monitoring the output.

C. Sensitivity Analysis

Stage 1 Initialization

1. *Parameters.xlsx* -> Contains the maximum and minimum values of the input parameters and information about which parameters are fixed.
2. Generate the Input parameter design matrix (***P1***) using - *design_matrix_generation.m*; (NOTE: Comment out the Stage 2 part of the code).
3. Each row in P1 corresponds to the different values of the parameters to be used in the *model.props* files.
4. Run the simulation using the hybrid computer code as described in Section B.

Stage 1 Analysis

1. Run the simulations (152 x 20 replicates) for each input parameter setting obtained from **P1** (see above, step 2 in the initialization stage).
2. Convert the data into *.csv file* format:
   a. 1st column: time points information (*i.e. Ticks*),
   b. 2nd column mean values and
   c. 3rd column standard deviations

   All the information will be obtained from the ENISI-MSM output runs.

3. Run *Stage1-PRCC.ipynb* - Formats the data to be used for the PRCC analysis and calculates the PRCC coefficients. (The code generates a data frame with rows from the Parameters.xlsx file and average of the output obtained for that parameter setting in the last column).
4. Plot the PRCC graphs using *Stage1-PRCC_barplots.R*
5. Alternatively, use Fig3-Code.ipynb jupyter notebook to recreate the figures in the paper.
6. Create an excel sheet with information about the active and inactive inputs from PRCC - PRCC_activeinactiveinputs-added.xlsx.

Stage 2 Initialization

1. Generate the Input parameter design matrix (***P2***) using – i) *design_matrix_generation.m* (NOTE: Comment out the Stage 1 part of the code) and ii) information regarding the active and inactive inputs present in PRCC_activeinactiveinputs-added.xlsx file.

2. Run the simulation using the hybrid computer code as described in Section B.

Stage 2 Analysis

1. Run the simulations (115 x 20 replicates) for each input parameter setting obtained from ***P2*** (see above, step 1 in the initialization stage).
2. Convert the data into .csv file format:
    a. 1st column: time points information (*i.e. Ticks*),
    b. 2nd column mean values and
    c. 3rd column standard deviations.

    All the information will be obtained from the ENISI-MSM output runs.

3. Combine all the outputs obtained from P2 and P1. (outputs obtained after running simulation for P1 from Stage 1, Section C and for P2 from Stage 2, Section C).
   Create folders for each of the cell (cells are represented as agents in each compartment) populations and save the files from step 2, Sage 2, Section C.

4. Run *Stage2-inputfilegeneration.m* and save the output as ***.mat*** file to be used to build a temporal metamodel.
5. Build a temporal metamodel using *Stage2-BuildTempMM.R* and save the output as ***.Rdata*** dataset.
6. Calculate the Sobol Indices using *Stage2-SA-temporal6tps.R*. The input to the code includes the ***.Rdata*** obtained from the previous step 6 (stage 2 Analysis, Section C) and the datasets obtained after running SobolIndex_data_generation.m.
