# Supplementary material for: High-resolution computational modeling of immune responses in the gut
Source: Gigascience. 2019 Jun 11;8(6):giz062. doi: 10.1093/gigascience/giz062 (PMC6559340; doi:10.1093/gigascience/giz062)
Supplement: giz062_Supplement_Files [file giz062_supplement_files.zip › Table_S1_revised.docx]

|  | | **PARAMETER VALUES** | | | | | |  |
| --- | --- | --- | --- | --- | --- | --- | --- | --- |
| **Parameter** | **Description** | **Baseline (WT)** | **Min** | **Max** | **CD4 Cre** | **LysMCre** | **Clodronate** | **Unit** |
| p_epiinfbactdamage | Epithelial cell damage due to infectious bacteria | 0.9 | 0.3 | 1 | 0.9 | 0.9 | 0.9 | Per time step |
| p_epith1damage | Epithelial cell damage due to Th1 cells | 0.8 | 0.3 | 1 | 0.8 | 0.8 | 0.8 | Per time step |
| p_epith17damage | Epithelial cell damage due to Th17 cells | 0.8 | 0.3 | 0.9 | 0.8 | 0.8 | 0.8 | Per time step |
| p_EpiProliferation | Epithelial cell proliferaion | 0.6 | 0.2 | 0.9 | 0.6 | 0.6 | 0.6 | Per time step |
| p_EpiCellDeath | Epithelial cell death | 0.6 | 0.1 | 0.9 | 0.6 | 0.6 | 0.6 | Per time step |
| p_epiIL10h | Epithelial cell healing due to IL10 cytokine | 0.2 | 0.01 | 0.9 | 0.2 | 0.2 | 0.2 | Per time step |
| p_nTrep | Naïve T cell replication | 0.022 | 0.01 | 0.5 | 0.022 | 0.022 | 0.022 | Per time step |
| p_naiveTcelldeath | Naïve T cell death | 0.000025 | 0.0001 | 0.001 | 0.000025 | 0.000025 | 0.000025 | Per time step |
| p_allTrep | Replication of all the types of T helper cells | 0.2 | 0.2 | 0.5 | 0.2 | 0.2 | 0.2 | Per time step |
| p_iTregtoTh17 | Plasticity of induced Treg (iTreg) to Th17 cells | 0.001 | 0.0001 | 0.001 | 0.001 | 0.001 | 0.001 | Per time step |
| p_Th17toiTreg | Plasticity of Th17 to induced Treg (iTreg) cells | 0.001 | 0.0001 | 0.001 | ***0.0001*** | 0.0001 | 0.001 | Per time step |
| p_nTtoTr | Differentiation of naïve T cell to type 1 regulatory T cell | 0.4 | 0.04 | 0.4 | 0.4 | 0.04 | 0.4 | Per time step |
| p_nTtoiTreg | Differentiation of naïve T cell to induced Treg cell | 0.02 | 0.001 | 0.04 | ***0.001*** | 0.02 | 0.02 | Per time step |
| p_nTtoTh17 | Differentiation of naïve T cell to Th17 cell | 0.02 | 0.01 | 0.04 | 0.02 | 0.02 | 0.02 | Per time step |
| p_Th1death | Th1 cell death | 0.00001 | 0.004 | 0.03 | 0.00001 | 0.00001 | 0.00001 | Per time step |
| p_Th17death | Th17 cell death | 0.03 | 0.02 | 0.1 | 0.03 | 0.03 | 0.03 | Per time step |
| p_iTregdeath | iTreg cell death | 0.001 | 0.001 | 0.01 | 0.001 | 0.001 | 0.001 | Per time step |
| p_Trdeath | Type 1 regulatory T cell death | 0.001 | 0.001 | 0.01 | 0.001 | 0.001 | 0.001 | Per time step |
| p_IL10Tr | IL10 release by type 1 regulatory T cell | 1.2 | 1 | 2 | 1.2 | 1.2 | 1.2 | Per time step |
| dummy | Dummy | 0.3 | 0.3 | 0.6 | 0.3 | 0.3 | 0.3 | Per time step |
| p_BacteriaKill | Bacteria removed due to inflammatory responses | 0.5 | 0.05 | 0.6 | 0.5 | 0.5 | 0.5 | Per time step |
| p_BacteriaDeath | Bacteria death | 0.4 | 0.04 | 0.5 | 0.4 | 0.4 | 0.4 | Per time step |
| p_HPdeathduetoTcells | Helicobacter pylori death caused due to T cells | 0.0001 | 0.0001 | 0.001 | 0.0001 | 0.0001 | 0.0001 | Per time step |
| p_HPyloriDeath | Helicobacter pylori death | 0.0001 | 0.0001 | 0.001 | 0.0001 | 0.0001 | 0.0001 | Per time step |
| p_DCDeath | Dendritic cell death | 0.0003 | 0.0001 | 0.003 | 0.0003 | 0.0003 | 0.0003 | Per time step |
| p_Monocytedeath | Monocyte death | 0.04 | 0.01 | 0.1 | 0.04 | 0.04 | 0.04 | Per time step |
| p_resmacdeath | Resident macrophages death | 0.005 | 0.001 | 0.05 | 0.005 | 0.005 | 0.005 | Per time step |
| p_Trmackill | Macrophage killed due to type 1 regulatory T cells | 0.05 | 0.01 | 0.09 | 0.05 | 0.05 | 0.05 | Per time step |
| p_MregDiff | Differentiation of monocytes to regulatory macrophages | 0.3 | 0.1 | 0.6 | 0.3 | ***0.2*** | 0.3 | Per time step |
| p_resmacrep | Proliferation of resident macrophages | 0.2 | 0.1 | 0.6 | 0.2 | 0.2 | 0.2 | Per time step |
| p_monorep | Proliferation of monocytes | 0.04 | 0.01 | 0.5 | 0.04 | 0.04 | 0.04 | Per time step |
| p_IFNg | Release of IFNg | 0.5 | 0.1 | 0.8 | 0.5 | 0.5 | 0.5 | Per time step |
| p_IL10 | Release of IL10 | 0.2 | 0.1 | 0.8 | 0.2 | 0.2 | 0.2 | Per time step |
| p_IL17 | Release of IL17 | 0.1 | 0.1 | 0.8 | 0.1 | 0.1 | 0.1 | Per time step |
| p_IL21 | Release of IL21 (not implemented, can be considered as dummy variable 2) | 0.3 | 0.1 | 0.8 | 0.3 | 0.3 | 0.3 | Per time step |
| p_IL6 | Release of IL6 | 0.3 | 0.1 | 0.8 | 0.3 | 0.3 | 0.3 | Per time step |
| p_TGFb | Release of TGFb | 0.1 | 0.1 | 0.8 | 0.1 | 0.1 | 0.1 | Per time step |
| p_IL12 | Release of IL12 | 0.01 | 0.1 | 0.8 | 0.01 | 0.01 | 0.01 | Per time step |
| p_nTrep | Naïve T cell proliferation | 0.022 | Fixed | Fixed | 0.022 | 0.022 | 0.022 | Per time step |
| p_epicyto | Cytokine produced due to epithelial cells | 0.9 | Fixed | Fixed | 0.9 | 0.9 | 0.9 | Per time step |
| p_TotalTcap | Maximum amount of T cells allowed within a cell | 30 | Fixed | Fixed | 30 | 30 | 30 | Per time step |
| p_randomdam | Random damage of epithelial cell | 0.01 | Fixed | Fixed | 0.01 | 0.01 | 0.01 | Per time step |
| p_nTtoTh1 | Differentiation of naïve T cell to Th1 cell | 0.4 | Fixed | Fixed | 0.4 | 0.4 | 0.4 | Per time step |
| p_tcellmove | T cell movement | 0.4 | Fixed | Fixed | 0.4 | 0.4 | 0.4 | Per time step |
| p_Th17cyto | Release of cytokine from Th17 cells | 0.9 | Fixed | Fixed | 0.9 | 0.9 | 0.9 | Per time step |
| p_Th1cyto | Release of cytokines form Th1 cells | 0.9 | Fixed | Fixed | 0.9 | 0.9 | 0.9 | Per time step |
| p_TroriTregcyto | Release of cytokine from type 1 regulatory cell or induced regulatory T cell | 0.9 | Fixed | Fixed | 0.9 | 0.9 | 0.9 | Per time step |
| p_BacteriaLPProl | Proliferation of bacteria in the lamina propria | 0.3 | Fixed | Fixed | 0.3 | 0.3 | 0.3 | Per time step |
| p_BacteriaLumProl | Proliferation of bacteria in the lumen | 0.3 | Fixed | Fixed | 0.3 | 0.3 | 0.3 | Per time step |
| p_HPepitoLP | Movement of *H. pylori* from epithelium to lamina propria | 0.125 | Fixed | Fixed | 0.125 | 0.125 | 0.125 | Per time step |
| p_Hpyloricap | Maximum amount of *H. pylori* | 30 | Fixed | Fixed | 30 | 30 | 30 | Per time step |
| p_HPylorirep | Proliferation of *H. pylori* | 0.01 | Fixed | Fixed | 0.01 | 0.01 | 0.01 | Per time step |
| p_iDCtoeDCLP | Differentiation of immature dendritic cell to effector dendritic cell in lamina propria | 0.6 | Fixed | Fixed | 0.6 | 0.6 | 0.6 | Per time step |
| p_iDCtotDCLP | Differentiation of immature dendritic cell to tolerogenic dendritic cell in lamina propria | 0.3 | Fixed | Fixed | 0.3 | ***0.095*** | 0.3 | Per time step |
| p_iDCtoeDCE | Differentiation of immature dendritic cell to effector dendritic cell in epithelium | 0.6 | Fixed | Fixed | 0.6 | 0.6 | 0.6 | Per time step |
| p_iDCtotDCE | Differentiation of immature dendritic cell to tolerogenic dendritic cell in epithelium | 0.5 | Fixed | Fixed | 0.5 | 0.5 | 0.5 | Per time step |
| p_iDCrep | Proliferation of immature dendritic cell | 0.5 | Fixed | Fixed |  |  |  | Per time step |
| p_eDCcyto | Release of cytokine from effector dendritic cell | 0.9 | Fixed | Fixed |  |  |  | Per time step |
| p_tDCcyto | Release of cytokine from tolerogenic dendritic cell | 0.9 | Fixed | Fixed |  |  |  | Per time step |
| p_iDCmoveLPtoEpi | Movement of immature dendritic cells from lamina propria to epithelium | 0.6 | Fixed | Fixed |  |  |  | Per time step |
| p_iDCmoveEpitoLP | Movement of immature dendritic cells from epithelium to lamina propria | 0.6 | Fixed | Fixed |  |  |  | Per time step |
| p_DCLPtoGLN | Movement of (effector and tolerogenic) dendritic cells from lamina propria to gastric lymph node | 0.4 | Fixed | Fixed |  |  |  | Per time step |
| p_DCEpitoLP | Movement of (effector and tolerogenic) dendritic cells from epithelium to lamina propria | 0.6 | Fixed | Fixed |  |  |  | Per time step |
| p_DCbasal | Maximum amount of dendritic cells | 10 | Fixed | Fixed |  |  |  | Per time step |
| p_MinfDiff | Differentiation of monocyte to inflammatory macrophages | 0.3 | Fixed | Fixed |  |  |  | Per time step |
| p_resmaccyto | Release of cytokine from resident macrophage | 0.9 | Fixed | Fixed | 0.9 | 0.9 | 0.9 | Per time step |
| p_Monocap | Maximum amount of monocytes | 35 | Fixed | Fixed | 35 | 35 | 35 | Per time step |
| p_resmacCap | Maximum amount of resident macrophages | 35 | Fixed | Fixed | 35 | 35 | 35 | Per time step |
| p_Mregcyto | Release of cytokine from regulatory macrophage | 0.9 | Fixed | Fixed | 0.9 | 0.9 | 0.9 | Per time step |
| p_Minfcyto | Release of cytokine form inflammatory macrophage | 0.9 | Fixed | Fixed | 0.9 | 0.9 | 0.9 | Per time step |
| p_Hpregenv | *H. pylori* promotes the creation of regulatory environment | 0.7 | Fixed | Fixed | 0.7 | 0.7 | 0.7 | Per time step |
| p_EpiCap | Maximum amount of epithelial cells | 20 | Fixed | Fixed | 20 | 20 | 20 | Per time step |
| p_BacCap | Maximum amount of bacteria | 20 | Fixed | Fixed | 20 | 20 | 20 | Per time step |
| lamina_propria.eIL6.initialValue | Initial value | 10 | Fixed | Fixed | 10 | 10 | 10 |  |
| lamina_propria.eIL6.diffusion | Diffusion of IL6 cytokine | 0.6 | Fixed | Fixed | 0.6 | 0.6 | 0.6 | mm^2^/day |
| lamina_propria.eIL6.degradation | Degradation of IL6 cytokine | 0.05 | Fixed | Fixed | 0.05 | 0.05 | 0.05 | day^-1^ |
| lamina_propria.TGFb.initialValue | Initial value | 50 | Fixed | Fixed | 50 | 50 | 50 |  |
| lamina_propria.TGFb.diffusion | Diffusion of TGFb cytokine | 0.6 | Fixed | Fixed | 0.6 | 0.6 | 0.6 | mm^2^/day |
| lamina_propria.TGFb.degradation | Degradation of TGFb cytokine | 0.02 | Fixed | Fixed | 0.02 | 0.02 | 0.02 | day^-1^ |
| lamina_propria.eIL12.initialValue | Initial value | 20 | Fixed | Fixed | 20 | 20 | 20 |  |
| lamina_propria.eIL12.diffusion | Diffusion of IL12 cytokine | 0.6 | Fixed | Fixed | 0.6 | 0.6 | 0.6 | mm^2^/day |
| lamina_propria.eIL12.degradation | Degradation of IL12 cytokine | 0.02 | Fixed | Fixed | 0.02 | 0.02 | 0.02 | day^-1^ |
| lamina_propria.eIL17.initialValue | Initial value | 0.4 | Fixed | Fixed | 0.4 | 0.4 | 0.4 |  |
| lamina_propria.eIL17.diffusion | Diffusion of IL17 cytokine | 0.6 | Fixed | Fixed | 0.6 | 0.6 | 0.6 | mm^2^/day |
| lamina_propria.eIL17.degradation | Degradation of IL17 cytokine | 0.02 | Fixed | Fixed | 0.02 | 0.02 | 0.02 | day^-1^ |
| lamina_propria.eIL10.initialValue | Initial value | 4 | Fixed | Fixed | 4 | 4 | 4 |  |
| lamina_propria.eIL10.diffusion | Diffusion of IL10 cytokine | 0.6 | Fixed | Fixed | 0.6 | 0.6 | 0.6 | mm^2^/day |
| lamina_propria.eIL10.degradation | Degradation of IL10 cytokine | 0.02 | Fixed | Fixed | 0.02 | 0.02 | 0.02 | day^-1^ |
| lamina_propria.eIFNg.initialValue | Initial value | 4 | Fixed | Fixed | 4 | 4 | 4 |  |
| lamina_propria.eIFNg.diffusion | Diffusion of IFNg cytokine | 0.6 | Fixed | Fixed | 0.6 | 0.6 | 0.6 | mm^2^/day |
| lamina_propria.eIFNg.degradation | Degradation of IFNg cytokine | 0.02 | Fixed | Fixed | 0.02 | 0.02 | 0.02 | day^-1^ |
| gastric_lymph_node.eIL6.initialvalue | Initial value | 50 | Fixed | Fixed | 50 | 50 | 50 |  |
| gastric_lymph_node.eIL6.idiffusion | Diffusion of IL6 cytokine in gastric lymph node | 0.6 | Fixed | Fixed | 0.6 | 0.6 | 0.6 | mm^2^/day |
| gastric_lymph_node.eIL6.degradation | Degradation of IL6 cytokine in gastric lymph node | 0.02 | Fixed | Fixed | 0.02 | 0.02 | 0.02 | day^-1^ |
| gastric_lymph_node.TGFb.initialvalue | Initial value | 50 | Fixed | Fixed | 50 | 50 | 50 |  |
| gastric_lymph_node.TGFb.idiffusion | Diffusion of TGfb cytokine in gastric lymph node | 0.6 | Fixed | Fixed | 0.6 | 0.6 | 0.6 | mm^2^/day |
| gastric_lymph_node.TGFb.degradation | Degradation of TGFb cytokine in gastric lymph node | 0.02 | Fixed | Fixed | 0.02 | 0.02 | 0.02 | day^-1^ |
| gastric_lymph_node.eIL12.initialvalue | Initial value | 10 | Fixed | Fixed | 10 | 10 | 10 |  |
| gastric_lymph_node.eIL12.idiffusion | Diffusion of IL12 cytokine in gastric lymph node | 0.6 | Fixed | Fixed | 0.6 | 0.6 | 0.6 | mm^2^/day |
| gastric_lymph_node.eIL12.degradation | Degradation of IL12 cytokine in gastric lymph node | 0.02 | Fixed | Fixed | 0.02 | 0.02 | 0.02 | day^-1^ |
| gastric_lymph_node.eIL17.initialvalue | Initial value | 5 | Fixed | Fixed | 5 | 5 | 5 |  |
| gastric_lymph_node.eIL17.idiffusion | Diffusion of IL17 cytokine in gastric lymph node | 0.6 | Fixed | Fixed | 0.6 | 0.6 | 0.6 | mm^2^/day |
| gastric_lymph_node.eIL17.degradation | Degradation of IL17 cytokine in gastric lymph node | 0.02 | Fixed | Fixed | 0.02 | 0.02 | 0.02 | day^-1^ |
| gastric_lymph_node.eIL10.initialvalue | Initial value | 4 | Fixed | Fixed | 4 | 4 | 4 |  |
| gastric_lymph_node.eIL10.idiffusion | Diffusion of IL10 cytokine in gastric lymph node | 0.6 | Fixed | Fixed | 0.6 | 0.6 | 0.6 | mm^2^/day |
| gastric_lymph_node.eIL10.degradation | Degradation of IL10 cytokine in gastric lymph node | 0.02 | Fixed | Fixed | 0.02 | 0.02 | 0.02 | day^-1^ |
| gastric_lymph_node .eIFNg.initialValue | Initial value | 4 | Fixed | Fixed | 4 | 4 | 4 |  |
| gastric_lymph_node.eIFNg.diffusion | Diffusion of IFNg cytokine | 0.6 | Fixed | Fixed | 0.6 | 0.6 | 0.6 | mm^2^/day |
| gastric_lymph_node .eIFNg.degradation | Degradation of IFNg cytokine | 0.02 | Fixed | Fixed | 0.02 | 0.02 | 0.02 | day^-1^ |
