# Supplementary figures and images for: High-resolution computational modeling of immune responses in the gut
Source: Gigascience. 2019 Jun 11;8(6):giz062. doi: 10.1093/gigascience/giz062 (PMC6559340; doi:10.1093/gigascience/giz062)

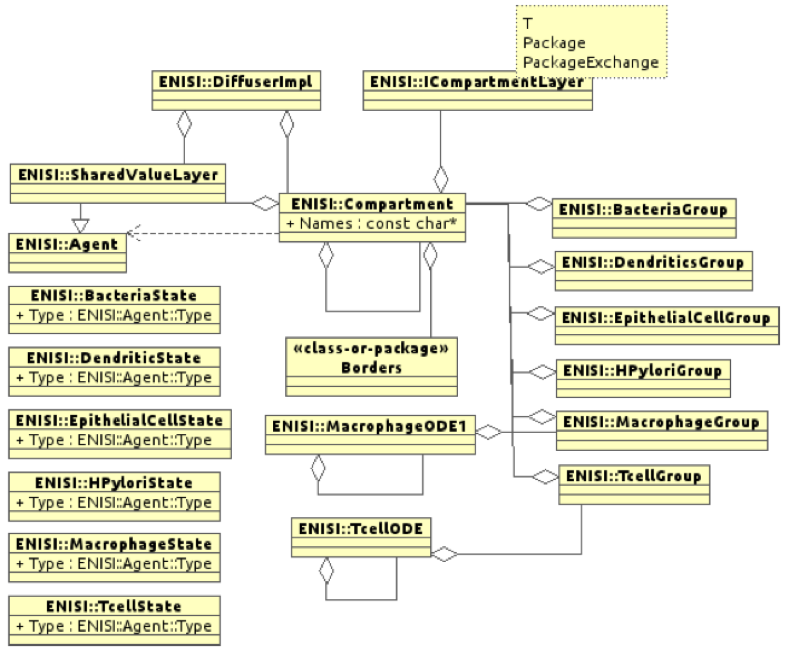

Supplement: giz062_Supplement_Files [file giz062_supplement_files.zip › FigS1.png]

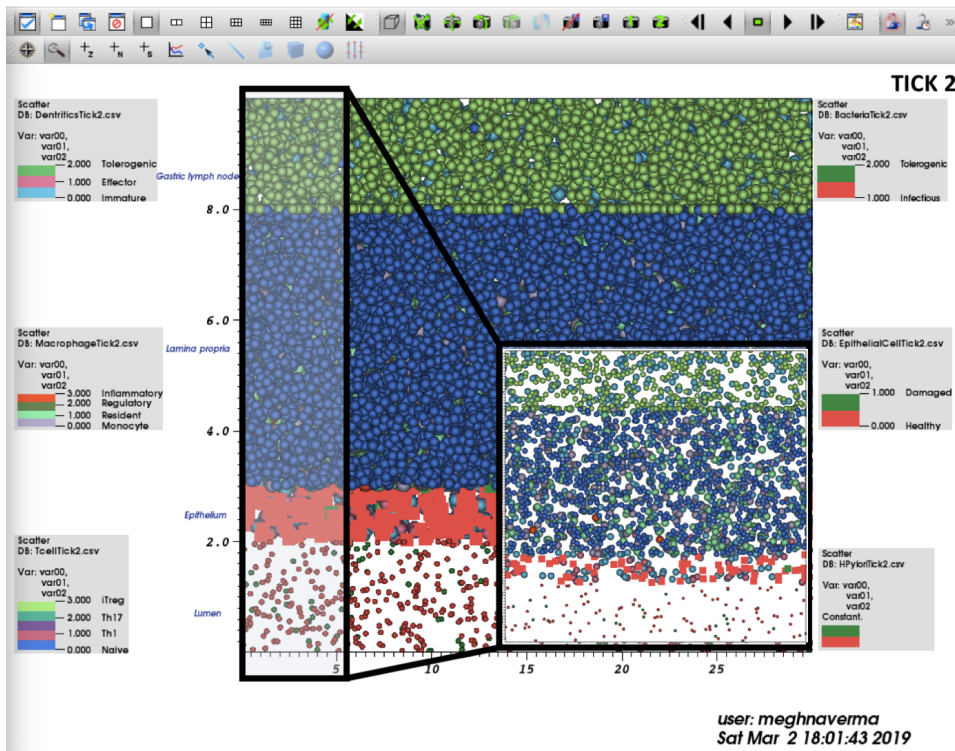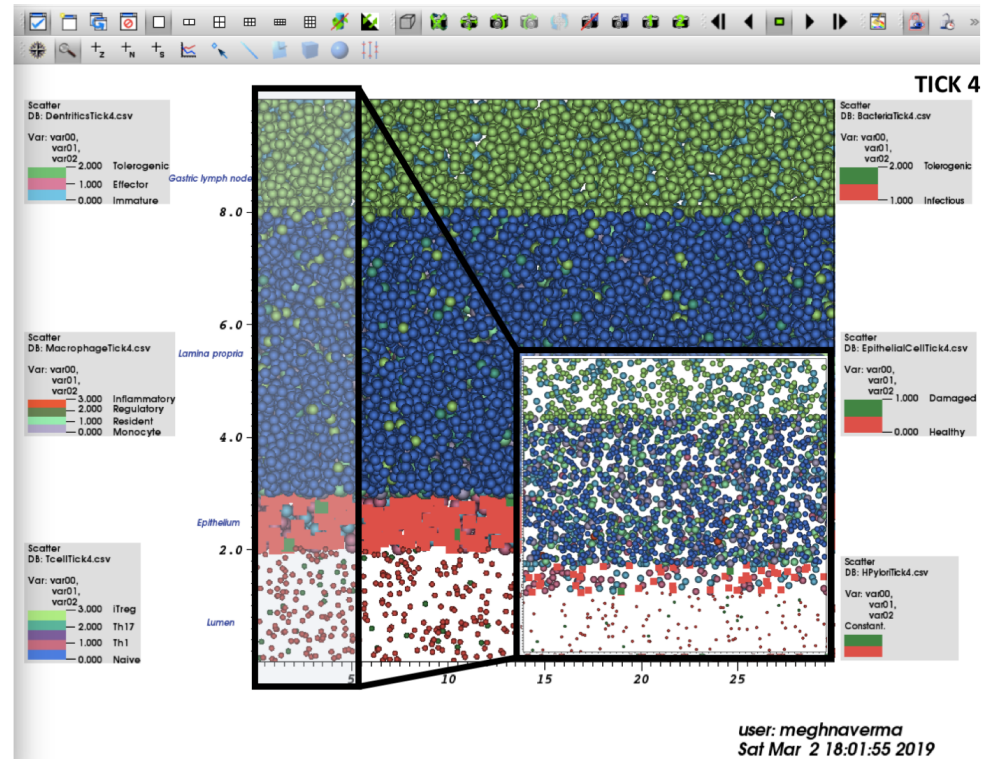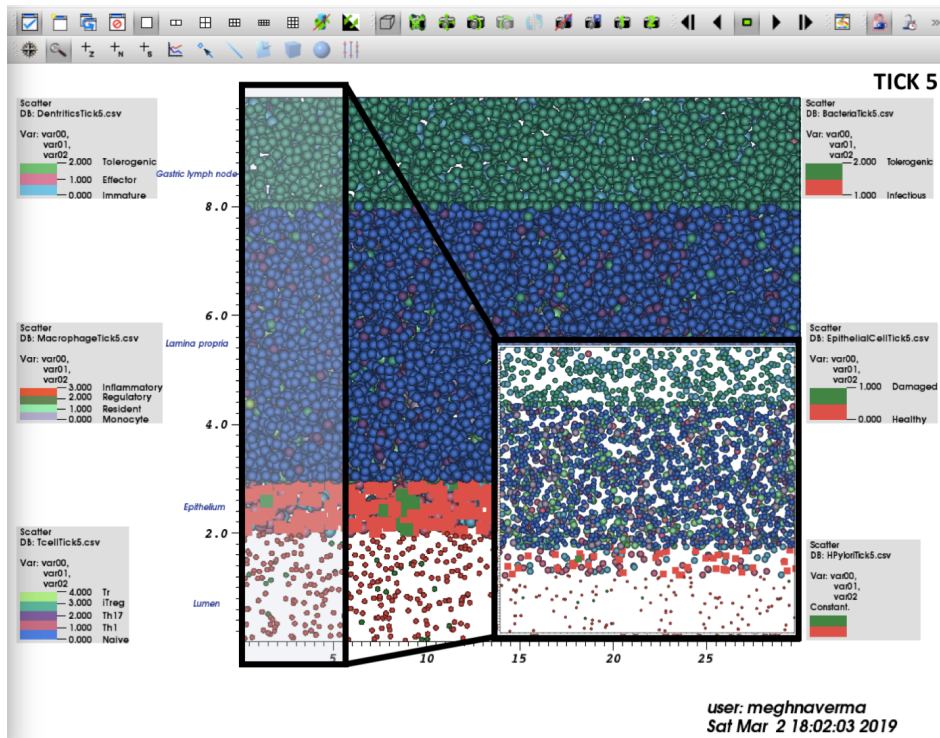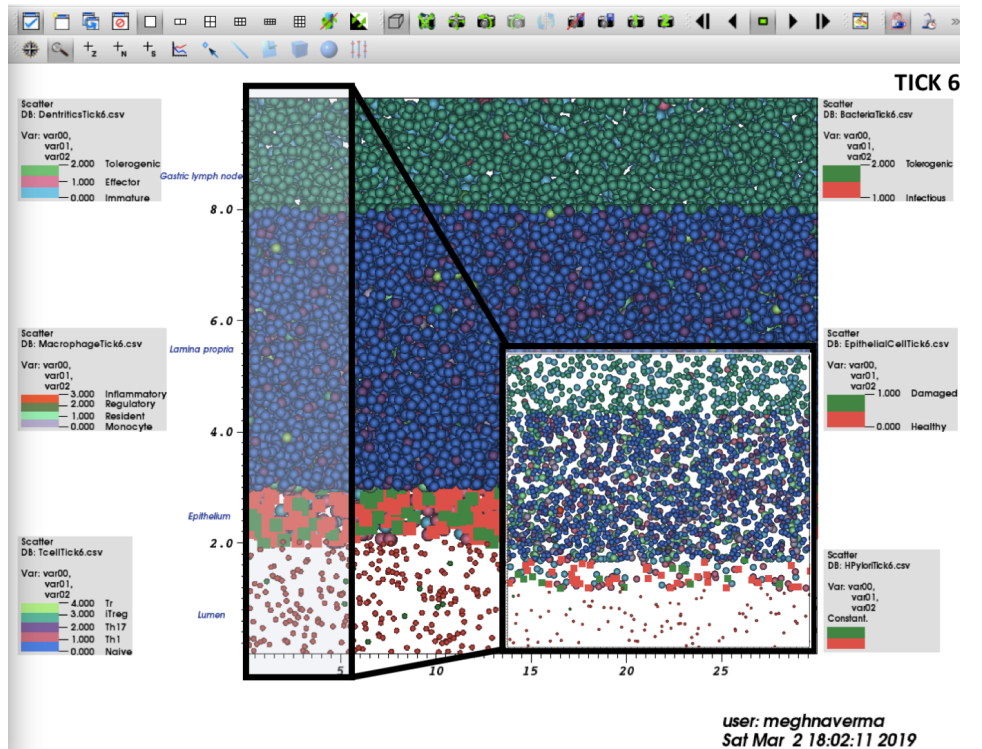

Supplement: giz062_Supplement_Files [file giz062_supplement_files.zip › FigS2.pdf]

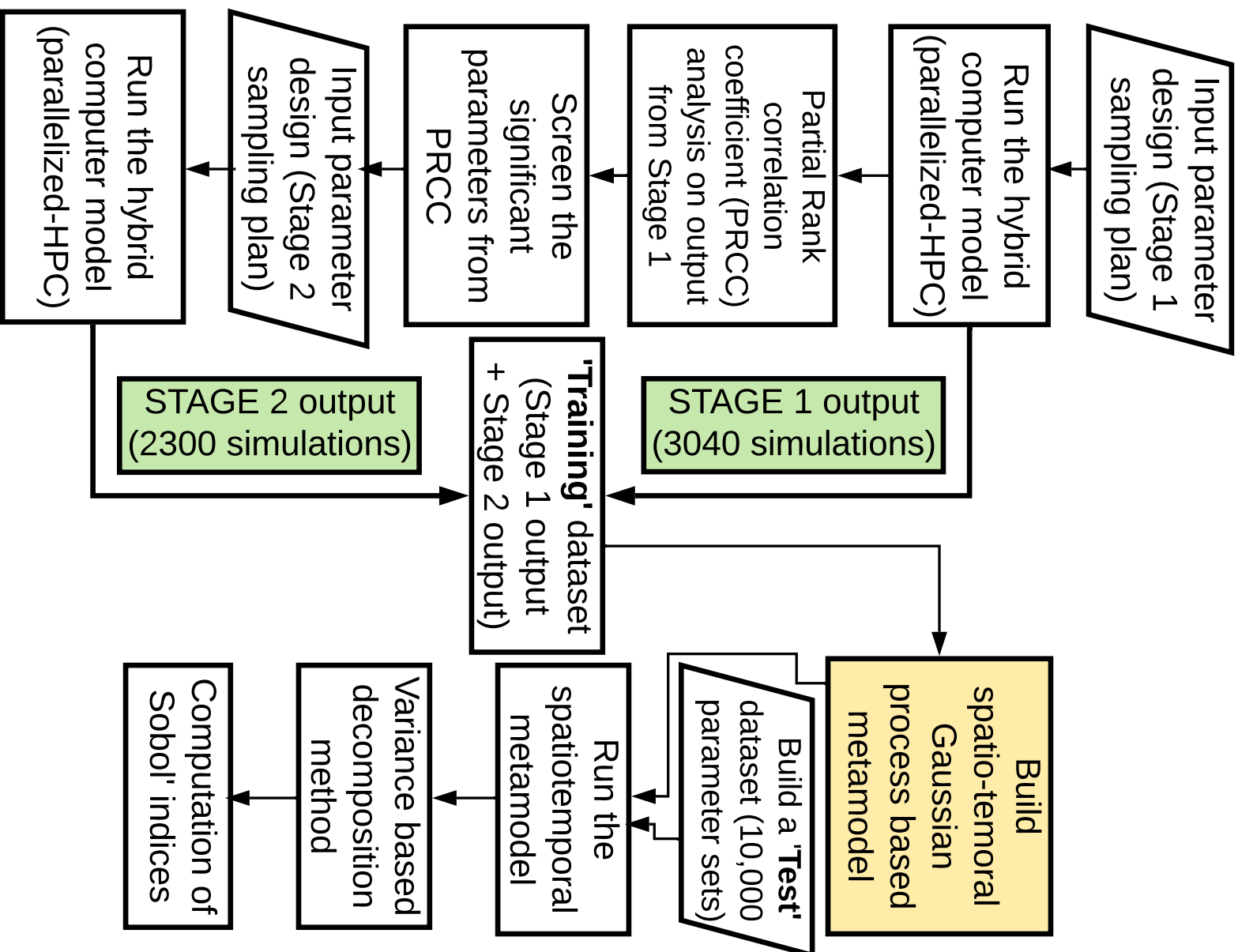

Supplement: giz062_Supplement_Files [file giz062_supplement_files.zip › FigS3.pdf]
