# Supplementary material for: High-resolution computational modeling of immune responses in the gut
Source: Gigascience. 2019 Jun 11;8(6):giz062. doi: 10.1093/gigascience/giz062 (PMC6559340; doi:10.1093/gigascience/giz062)
Supplement: giz062_Response_to_Reviewer_Comments_Original_Submission [file giz062_response_to_reviewer_comments_original_submission.pdf]

Author's Response To Reviewer Comments

Close

Point by point response to the Reviewer reports

We would like to thank the reviewers and editors involved for dedicating valuable time to review our manuscript entitled “High-Resolution Computational Modeling of Immune Responses in the Gut”. We are extremely grateful to both reviewers and the editors for their time and attention to our submission. The review was very helpful to us, and the recommended changes have allowed us to provide better documentation and clarity to the present work. The review process has been helpful in the improvement of our submission. We have considered the comments that were made and have prepared the following point-by-point response.

Reviewer #1: In this manuscript, the authors constructed a multi-scale systems biology model of Helicobacter pylori infection to study the interaction between bacterial infection and the immune system. Some modifications could be considered to improve the quality of this manuscript:

1. The model needs to be more clearly described in the text. Some details might be available from the code; nevertheless, it would be helpful for readers to understand if the authors can include more information regarding the model. For example:

We thank the reviewer for their valuable suggestion. We agree with the reviewer’s comment and accordingly we updated the manuscript with the response described below in detail.

a. Agent-based model:
i. What is the spatial discretization? The authors mentioned it's a 30*10 2D grid cell, but resident macrophages are in thousands. So multiple cells are allowed in the same grid location? How many?

Response: i) The model has a spatial discretization such that the dimension of the entire (two-dimensional) grid is 30nm x 10 nm). An individual grid cell is 1nm x 1nm, however, this is a configurable run parameter and can be changed without modifying the model. An individual grid cell is a unit wherein all the agents located within that location have the same cytokine environment, i.e., for all the agents in that location, ENISI-MSM would send the same concentration of the cytokines to COPASI. The resulting time series of cytokine concentrations will be used to update the cytokine value in the ABM/PDE system and COPASI would simulate a different model for each of the relevant cell type within that individual grid cell. Below is a figure describing the grid, also added in the Additional file Fig S2.

The entire grid is divided within into 4 functionally and anatomically distinct sized compartments such that the dimensions of the 4 compartments are lumen (2nm), epithelium (1nm), lamina propria (5nm) and gastric lymph node (2nm).
The following compartments are adjacent to each other:
• Lumen - epithelium
• Epithelium - lamina propria
• Lamina propria – gastric lymph node

In the model, there are multiple cells and cell types (i.e., agents) within this dimensional grid. At the beginning of each simulation cycle, the agents were randomly placed separated by the four compartments within the 2D grid. The separation of different types of agents, corresponding to different cell types, into compartments within the grid is based on the conceptual framework that underlines the model, which is based on author’s expertise and available information. Currently the individual agents do not have any physical size meaning that there is no limit of agents within each individual spatial grid cell. The model is initialized with the concentration of different cell types (i.e. agents for e.g. macrophages) at the beginning of the simulation by the user. We demonstrate below how we obtain a count of thousands of resident macrophages. For e.g., if the initial concentration of resident macrophages in the lamina propria is 30, the total number of these resident macrophages can be calculated by the equation (1) described below -

n(resident macrophages) = sizecompartment(lamina propria) x concentrationintial (resident macrophages) (1).

n(resident macrophages) = (30 x 5) x 30 = 4500.

The manuscript has been updated with the above addressed points, please refer to Line129 - Line147, and L217-245.

ii. What is the time step size?
Response: The time step size is 1 tick ~ 1 day which was obtained during the process of fitting the output to the results from the mouse model of H. pylori infection. For e.g. the peak of resident macrophages in lamina propria (refer Fig 2b,d) is observed at ~21 days which is similar to the results obtained in Fig 2A described in ((Viladomiu, Bassaganya-Riera et al. 2017) (also described in detail in point by point response 2.b).
The manuscript has been updated with the above addressed points, please refer to Line247 - Line253.
iii. How is migration implemented for cells and bacteria agents?
Response: The cells and bacteria agents presented in the model have Brownian motion and move randomly within the compartment. Brownian movement is an inherent property of a cell. Depending on cell phenotypes the movement can vary, but all cells with the same phenotype exhibit similar movements. Additionally, chemokine-driven movement is dependent on chemokine concentration in a tissue site. The capability of chemokine-driven movement exists in ENISI-MSM if the right chemokines are represented in the model. However, the focus of this model was to investigate changes in cell phenotype and not chemokine-driven movement of cells. Thus, the chemokines driving the movement are not represented in the current model. Cell migration is implemented in the code as the move() function for each of the cells and agents, which call the moveRandom() function from the (https://github.com/NIMML/ENISI-MSM/src/compartment/Compartment.cpp) file.

The manuscript has been updated with the above addressed points, please refer to Line294 - Line307.

b. ODE: What's the COPASI setup for the solver? How is the solver in sync with the ABM?
Response: The COPASI setup for the solver uses the LSODA (Livermore Solver for Ordinary Differential Equations) differential equation solver. The default values for the setup such as the - relative tolerance (1e-6), absolute tolerance (1e-12) and maximum internal steps of 10000 were maintained. The ENISI MSM sends the current concentrations of the cytokines
to COPASI. COPASI uses those values to integrate the deterministic
model for one tick, i.e., 1 day. The resulting time series of cytokine
concentrations are used to update the cytokine value in the ABM/PDE
system. COPASI simulates different model for each relevant cell type.

The manuscript has been updated with the above addressed points, please refer to Line266 - Line274.

c. PDE: What package and numerical scheme is used to solve the PDEs? What's the setting?
Response: ENISI MSM is a multiscale agent-based modeling platform for computational immunology which was building on our previous works, ENISI MSM that integrated COPASI, the ODE solver, ENISI, an agent based simulator (Mei, Abedi et al. 2015).
The ENISI MSM PDE solver uses a simple numerical scheme to solve the PDEs (https://github.com/NIMML/ENISI-MSM/tree/master/src/diffuser) and process distributed value layer (https://github.com/NIMML/ENISI-MSM/blob/master/src/grid/ValueLayer.h). The ValueLayer stores the value for a grid space and provides methods to change the values of individual grid cells. The Diffuser is used to diffuse the values of the ValueLayer using diffusion (d) and degradation (delta) constants as described in (Mei, Abedi et al. 2015). The diffusion constant determines the migration of values of a grid cell to its neighboring grid cells. As implemented in ValueLayer library, the diffusion of cytokines follows the equation shown below also described in Mei el al, 2015. Here, vn is the value of the grid cell itself at step n. The values of cdelta and cd are degradation and diffusion constant respectively.
vn = vn-1 + cdelta * [∑(cd neighbor *vn-1neighbor) - 6.0 * vn-1]
0.3
1.2
0.3
1.2

-6.0
1.2
0.3
1.2
0.3

The PDE solver uses the above number scheme cd neighbor for the diffusion process. The step size cdelta is automatically adjusted at the beginning of the simulation based on the degradation and diffusion constants to avoid underflow errors, i.e., multiple PDE steps are in general executed per tick. The grid size is the identical with the spatial discretization for the agents.
We updated the manuscript details to solve PDEs and the setting, please refer to L275-L293.
2. The authors listed the values of parameters in Table S1.
a. However, it's not clear what their units are (the baseline column seems to include characters such as "I^2", "#" or "d". are these units? Please clarify).
Response: We thank the reviewer for pointing this out. Those were the units for the parameters in the COPASI ODE models. The parameters described in Table S1 are probability values (ranging from 0 to 1) and hence do not have any units. The characters have been removed and the column 3 of Table_S1 has been updated.

b. Also, the sources of the parameter values are not very clear, except for the vague statement "expert judgement" (Saltelli, Tarantola et al. 2000 is cited, but this is an article on SA and does not contain parameters).
Response: The values of the parameters for the model presented here are obtained via best guess based on the qualitative comparison of the computer model outputs with that of the experimental results obtained from the mouse model of H. pylori infection (Viladomiu, Bassaganya-Riera et al. 2017) published by NIMML (described here below in the last paragraph detail).

We want to clarify the practice of using expert opinion is known in the SA field and hence we cited Saltelli, Tarantola et al. 2000 as it supports the statement. As discussed in (Thorne, Bailey et al. 2007), one of the challenges encountered using ABM is the process of determining the parameter values, for e.g. this may include the lack of the availability of experimental techniques to measure such parameters. Since, the source of the parameters is not known we estimated the values to fit the data obtained from the mouse model of infection.

The experimental results in the mouse model indicated that between weeks 2 and 3 post-infection a decrease in bacterial burden in the stomach of LysMcre mice was observed as shown in Fig 1A of Viladomiu, Bassaganya-Riera et al. 2017. The decrease in bacterial burden led to a significant and sustained lower colonization levels when compared to WT and CD4Cre. Similar to the results observed in the mouse model, we observed a significant decrease (Fig 2a,d) in the bacterial burden in the simulated LysMcre group as compared to the simulated WT and CD4cre groups.
Furthermore, the results from the mouse model indicated that a significant increase in numbers of F4/80hiCD11b+ CD64+ CX3CR1+ cells (here referred to as resident macrophages in this paper), was observed in WT mice in comparison with LysMcre mice as shown in Fig. 2A, 2E of Viladomiu, Bassaganya-Riera et al. 2017. These cells accumulated in the stomach mucosa starting on day 14 post-infection in the WT mice but not in the LysMcre mice. We observed a similar increase (Fig 2b,e and Fig 2c,f) in the number of resident macrophages as well as monocyte derived macrophages in the simulated WT groups in comparison to the simulated LysMcre group.

We updated the manuscript accordingly, please refer to L340-L350 and L654-671.

c. Please in the table explain what mechanism each parameter corresponds to. Some can be inferred from the name, but it's not very clear.
Response: We thank the reviewer for this valuable suggestion. We added column 2 in Table S1 that describes the detailed mechanism that each parameter corresponds to. We updated the manuscript accordingly, please refer to L236-L238.
For e.g. 3 rows of the Table S1 are shown below –

Table S1

Name of parameters

Description
p_epiinfbactdamage
Epithelial cell damage due to infectious bacteria
p_epith1damage
Epithelial cell damage due to Th1 cells
p_epith17damage
Epithelial cell damage due to Th17 cells

d. Some parameters are not included in the table. For example, the diffusivity of the cytokines are not listed.
We thank the reviewer for this valuable observation. We listed the diffusivity of the cytokines and updated the Table_S1.

3. In Table 1 and Table 2, there is a T cell class named "Tr", which is not explained in the text. Please clarify.
Response: We thank the reviewer for pointing this out. The Tr cells are the type 1 regulatory (Tr1) T cells that are regulatory subset of T cells, whose expansion is dependent on environmental IL-10 (produced by Mreg). These are different than iTreg which are T cells differentiated from naïve T cell in presence of tolerogenic dendritic cells and TGF-β cytokine. We clarified this point and updated the manuscript, please refer L208-211.

4. The authors used a Gaussian emulator as surrogate model for the hybrid model. In line 582, the authors mentioned that performance is evaluated using diagnostic plots in Figure S4. Please clarify what the "Observed" data refers to. Are these the same simulations from the training set which the emulator fitted to, or are these new simulations done? If these are the training set results, the authors need to run simulations and emulation on a new testing set and evaluate the performance; if it's already done, please clarify how its done (range of parameters, number of simulations, etc.)
Response: We thank the reviewer for your careful reading and bringing up the issues in the description of the original plot. Below please find our response to your comments.
First, the "observed" data, i.e. the 'x' axis in the first half of lower panel in Figure S4 (shown here below as Fig 1a) (please note in the revised manuscript the Figure S4 is now updated and referred to as by Fig S5.), refers to the observed output values of the simulations obtained after running the hybrid computer model, whereas the 'y' axis refers to the predicted values obtained from the cross validated model. These diagnostic plots denote the black circles which are the cross validated prediction. Cross validation is in the sense that for predictions made at design point x, all observations at design point x are removed from the training set. The second half of lower panel refers to the standard residual plot wherein the 'x' axis represents the observed values obtained from the simulation and the 'y' axis refers to the residual error ({error (predicted values – observed values) / standard deviation (error)}) obtained.

In fact, the models used for plotting are the cross-validated ones and are not fit using the entire dataset. Cross-Validation (section 7.10 of The Elements of Statistical Learning (Trevor, Robert et al. 2009), is a legitimate approach for model assessment and it is especially suitable in our case because the simulation data is expensive to obtain (each simulation takes ~9-10 minutes to run, thus 267 parameter sets with 20 replicates = 5,340 simulations. The entire simulation dataset took us about 2 months to obtain.

Nevertheless, we would like to show that using separate testing and training dataset for model assessment we obtained similar conclusions as those using the cross validated model.
We randomly split the observed output simulation dataset for one of the datasets (Fig S4 "a"), Helicobacter pylori in Lamina propria into training (80%) and testing (20%) sets and built the Gaussian emulator using the mlegp package. As observed in the Fig 1b and Fig 1c below, we plotted the predicted (values predicted using mlegp) vs. the observed simulation data values for both the training set (top panel of Fig 1b) and testing sets (top panel of Fig 1c). In the top panels of Fig 1b, the black circles denote the cross validated prediction points for the training dataset. Similarly, the top panel of Fig 1c, the black open circles are obtained after plotting the predictions for testing dataset, made using the model trained on the 80% of the randomly split dataset, vs. the observed values (known) for the 20% of the randomly split used as testing dataset here. Additionally, we calculated the standardized residuals for each of the 80% and 20% randomly split datasets and plotted the standardized residual plots in the lower panels of Fig. 1b and Fig 1c respectively.

As observed in the bottom panels of the Fig 1a, 1b and 1c the amount of standard residuals obtained for the cross validated model (Fig S4 a) from the paper and also the one mentioned in previous paragraph), the training dataset (80% randomly split dataset), and testing dataset (20% randomly split dataset) respectively, were similar. Thus, here we demonstrated that the results obtained from the cross-validated model built using mlegp (from Figure S4 a) and as shown here in Fig 1a) were similar to the results obtained using the cross-validation technique by randomly splitting the data into 80% and 20% (shown here in Fig 1b and Fig 1c).

Fig 1a. Original plot from Fig S4 a). The plot shows the predicted vs. observed simulation values for the Cross Validated (CV) model (top panel) and residual error plot for the CV model (bottom panel).

Fig 1b. The plot shows the predicted vs. observed simulation values for the randomly split 80% of the dataset (top panel) and residual error plot for the randomly split 80% of the dataset(bottom panel).

Fig 1b. The plot shows the predicted vs. observed simulation values for the randomly split 20% of the dataset (top panel) and residual error plot for the randomly split 20% of the dataset(bottom panel).

We clarified that the observed data refers to observed simulation values and recreated the Figure S4 (now updated to Fig S5 with updated legends. Please refer to L1045-L1058 in the manuscript.

Reviewer #2: The authors present results from a multi-scale hybrid model of host immune responses to H pylori exposure in the gut. The paper addresses outstanding questions in this complex system and overall the results are interesting. Some comments/questions to be addressed are outlined below.

1. A key component of the introduction ("double edge sword, p 1 line 4") as well as in the discussion (p28 line 672 "dual role as pathogen and beneficial organism") mentions the conflicting roles of H pylori infection - however the results do not clearly connect to help answer this dichotomy. More detailed analysis/discussion of the results should be provided to clarify the conclusion or the focus of the intro/discussion should be adjusted to relate more closely to the results currently presented.
Response: We thank the reviewer for this valuable suggestion. This study addresses the dichotomy in the introduction but mostly focuses on investigating the dynamics that promote the tolerance to the bacterium in the gastrointestinal mucosa and its systemic immunoregulatory effects. We view the dichotomy represented by the beneficial effects of regulatory responses (immune tolerance to the bacterium) in lesion development versus the detrimental actions of effector responses. Since, the majority of H. pylori-colonized individuals, approximately 85%, do not present any detrimental effect, we wanted to contribute towards the further investigation of the dynamics of immunoregulatory mechanisms underlying H. pylori infection using computational modeling. We emphasized the need for investigation of the immunoregulatory role and the adjusted the focus of the introduction and discussion to relate more closely to the results highlighting regulatory immune cells here. We updated the manuscript accordingly, please refer to L4-L7, L52-L54, L62-L65 in introduction and L850-L854 in discussion.

2. Section 3.4 and p 29 line 694 discuss the involvement of regulatory macrophages and tolerogenic DCs on the colonization of H pylori. These conclusions appear to be drawn based on correlation between responses in H pylori and macrophage/DC populations upon epithelial cell proliferation adjustment (Fig 5). A causal connection between the macrophages/DCs and H pylori is not made (or is not clear to me from the text). If such a connection is embedded in the mechanisms included in Table 1 it should be outlined in the results section where the conclusion is made otherwise simulations targeting the macrophage/DC populations would be needed to confirm this hypothesis.
Response: We thank the reviewer for pointing this out. We want to clarify that computational modeling based studies are capable of providing predictive modeling derived insights, however, any definitive causal connection should be validated in an experimental or clinical setting. In this study, based on the results obtained from the metamodel based global SA, the epithelial cell proliferation parameter was shown to an impact on the H. pylori population.

Following these findings which highlighted the importance of epithelial cell proliferation, the biological hypothesis derived from this prediction is that the epithelial cell proliferation parameter is responsible for the higher colonization of H. pylori. Prior to conducting any experimental studies, we wanted to explore the hypothesis using our hybrid computer model in silico and study the model outputs obtained after we changed the epithelial cell proliferation parameter. Thus, we varied the epithelial cell proliferation parameter across a varying range of values (0.9-0.1) and studied its effect on the different output cell population (obtained after running the simulations). These outputs were the ones obtained after running the simulation using the hybrid computational model, as we varied the epithelial cell proliferation parameter. We analyzed the outputs from the hybrid computer model and observed upon decreasing the Epiprolifer from a range of values 0.9-0.1, the output cell populations with regulatory function, namely regulatory macrophages and tolerogenic dendritic cells were found to vary. Overall, these cell populations varied due to the variation in the epithelial cell proliferation parameter.

We want to clarify that such connection was not embedded in the mechanisms included in Table 1 but it represents an emergent behavior from the simulations predicting the involvement of regulatory and tolerogenic dendritic cells in the mechanisms of immunoregulation during H. pylori infection. Finally, the simulations targeting the epithelial cell proliferation caused a change in regulatory and tolerogenic dendritic cell population. This shows that the simulations indirectly targeted the regulatory and tolerogenic dendritic cell population. Thus, we hypothesize that epithelial cell proliferation might be responsible for the higher colonization of H. pylori through a mechanism that involves the regulatory macrophages and tolerogenic cells. This is in line with our own conclusions drawn from a previous paper (Viladomiu, Bassaganya-Riera et al. 2017) where we show that the presence of cells with regulatory phenotype favor higher levels of H. pylori colonization. The results from the sensitivity analysis presented in this paper suggest that epithelial proliferation might be a crucial part of the mechanisms by which these regulatory responses are induced and that there is a link between these parameters. The exact biological process however cannot be inferred from the current model and it will be investigated in follow-up in vivo studies.

We updated the manuscript with the detailed clarification, please refer to L788-826 and L908-L915.

3. Clarity is needed on some parts of the methods description:
3.1 P6, line 131: what are the units of the grid dimensions given. Are these the dimensions of a single grid cell or the entire grid? How are the 4 compartments separated on the grid?
Response : We thank the reviewer for pointing this out. These are the dimensions of the entire grid. An individual grid cell is 1nm x 1nm. The 4 compartments are separated by border implementation such that the dimensions of the 4 compartments are lumen (2nm), epithelium (1nm), lamina propria (5nm) and gastric lymph node (2nm). The following compartments are adjacent to each other:

- Lumen - epithelium
- Epithelium - lamina propria
- Lamina propria – gastric lymph node

We updated the manuscript with detailed model description, please refer to L222-L232. We also added a figure describing the grid in the Additional file Fig S2.

3.2 P6 line 149: what data were the ODEs calibrated to? Is there a reference?
Response: The CD4+ ODE model was calibrated using the experimental data provided in the Table S1 of

the reference - Carbo, Hontecillas et al. 2013. The Particle Swarm algorithm implemented in COPASI was used to determine unknown model parameter values and fully calibrate the model. The intracellular macrophage ODE model was calibrated using a combination of sourced and new data generated from in vitro macrophage differentiation studies, compiled into a dataset provided within S2 file of Leber, Bassaganya-Riera et al. 2016.
We accordingly updated the manuscript, please refer to L155-L166.

3.3 P6 line 150, and p22 line 524: ABM parameters were calibrated to "qualitatively resemble" the patterns observed in in vivo model. What patterns? What is considered to be qualitatively similar enough? Do the simulations reproduce the dynamics as well and the endpoint experimental observations? Inclusion of experimental data alongside the simulations in figure 2 or a description of the key dynamics (e.g. fold-changes, peak values etc.) would go a long way in communicating confidence in the model parameters.

Response: We thank the reviewer for their valuable suggestion. The values of the parameters are obtained based on the qualitative comparison of the model outputs with the experimental results obtained from the mouse model of H. pylori infection. The simulations reproduced similar dynamics as described below -
The results in the mouse model indicated that between weeks 2 and 3 post-infection a decrease in bacterial burden in the stomach of LysMcre mice (lacking PPARg in myeloid cells) was observed as shown in Fig 1A of (Viladomiu, Bassaganya-Riera et al. 2017). The decrease in bacterial burden led to a significant and sustained lower colonization levels when compared to WT and CD4Cre (lacking PPARg in T cells). Similar to the results observed in the mouse model, we observed a significant decrease (Fig 2a,d) in the bacterial burden in the simulated LysMcre group as compared to the simulated WT and CD4cre groups.

Furthermore, the results from the mouse model indicated that a significant increase in numbers of F4/80hiCD11b+ CD64+ CX3CR1+ cells (here referred to as resident macrophages in this paper), was observed in WT mice in comparison with LysMcre mice as shown in Fig. 2A, 2E of (Viladomiu, Bassaganya-Riera et al. 2017). These cells accumulated in the stomach mucosa starting on day 14 post-infection in the WT mice but not in the LysMcre mice. We observed a similar increase (Fig 2b,e and Fig 2c,f) in the number of resident macrophages as well as monocyte derived macrophages in the simulated WT groups in comparison to the simulated LysMcre group. As shown below, the peak of resident macrophages in lamina propria (refer Fig 2b of this paper) was observed at ~16-21 days which was similar to the peak observed in the CD64+F480hi macrophages at day 21, in Fig 2a described in (Viladomiu, Bassaganya-Riera et al. 2017). We included the experimental data alongside the simulation and revised the Fig 2 as shown below.

We accordingly updated the manuscript, please refer to L344-L350, L654-L671 and updated legend for Fig 2, L647-L649.

3.4 P11 line 246: the authors state that they perform global SA of the hybrid computer model. I believe they mean the metamodel here?
Response: We thank the reviewer for pointing this out. Although, a metamodel was built using the hybrid computer model, overall the global SA that included two stages –i) screening the influential inputs using PRCC (which was performed on the outputs from hybrid computer model simulations) and building a metamodel (using the outputs from the hybrid computer model) followed by calculating the Sobol' indices. Hence, we stated that we performed the global SA of the hybrid computer model.

3.5 P 21 line 480 and 484: parameter values were 'reduced' to emulate biological KOs. By how much were the parameters reduced?
Response: We thank the reviewer for pointing this out. We added new columns in Table S1 with the values of the parameters used to emulate the biological KOs. A complete set of parameter for each of the biological KOs are included as separate columns in Table S1.
To simulate the CD4Cre group, the probabilities of a naive T cell transitioning to an iTreg cell (p_nTtoiTreg) and Th17 cell differentiating to iTreg (p_Th17toiTreg) were reduced to 5% and 10% of the baseline (WT) value respectively (refer Table S1). As described in (Carbo, Hontecillas et al. 2013), to simulate the LysMCre experimental conditions, the probabilities of i) a monocyte transitioning to a

regulatory macrophage (p_MonotoMreg) and ii) immature dendritic cells switching to tolerogenic dendritic cells (p_iDCtotDC) were reduced approximately to 60% and 30% of the baseline (WT) value, respectively (refer Table S1).
We updated the manuscript with the above listed values, please refer to L602-615.

3.6 The in vivo model is mentioned several times before it is clarified to be a mouse model.
Response: We thank the reviewer for their valuable suggestion. We updated the manuscript and clarified that the in vivo model is a mouse model.

Comment from the Editor: Further, our series Guest Editor, Paul Macklin has had a quick look at the manuscript from a reproducibility point-of-view and suggests that you include somewhere (e.g., in supporting info) the specific examples for this paper, including detailed instructions on how to create the specific examples presented. Note that our curators also asked for detailed instructions on how to require detailed instructions for usability - not just code.

In addition, please register any new software application in the SciCrunch.org database to receive a RRID (Research Resource Identification Initiative ID) number, and include this in your manuscript. This will facilitate tracking, reproducibility and re-use of your tool.

Response: The RRID (Research Resource Identification Initiative ID) number as assigned by the SciCrunch.org database is SCR_016918. We included this in the manuscript, please refer to L180-L182.

Detailed instructions for the usability are described below and also included in Additional file S1. Detailed instructions on how to create the specific examples presented here are also included. We accordingly updated the manuscript, please refer to L178-180.

Additional file S1

This file contains the detailed instruction to Install ENISI MSM (Step I), Run a simulation (Step II) and Conduct Sensitivity Analysis (Step III). The jupyter (.ipynb) notebooks (Fig2-Code.ipynb, Fig3-Code.ipynb, Fig4-Code.ipynb and Fig5-Code.ipynb) include detailed instructions on how to create the specific figures presented in the paper.

A. How to install ENISI MSM
1. Create a folder for the hybrid computer model: mkdir ENISI
2. Change directory to the newly created folder: cd ENISI
3. Clone the dependencies required from the ENISI-Dependencies from the NIMML GitHub repository -
i. git clone --recursive https://github.com/NIMML/ENISI-Dependencies
4. Change the path to the ENISI-Dependencies folder: cd ENISI-Dependencies
5. Create a directory build within the folder: mkdir build
6. Change directory to the directory created in step 5: cd build
7. Start the installation: cmake ../
make
8. Change the directory cd
9. Change the directory to the one created in step 1: cd ENISI
10. Clone the ENISI-MSM model from the NIMML GitHub repository –
i. git clone —recursive https://github.com/NIMML/ENISI-MSM
11. Change the directory to ENISI-MSM: cd ENISI-MSM
12. Create a directory build within the folder: mkdir build
13. Change the directory to the directory created in step 12: cd build
14. Start the installation:
cmake -DENISI_MSM_DEPENDENCY_DIR=PATH TO ENISI-Dependencies FOLDER/install ..
make

B. How to run a simulation

1. Create a folder FolderName to save the simulation results. It is important to place all the results of every experiment and its respective files in different folders.

2. Place the files i) config.props ii) run.props iii) job.sh (required only if running on cluster) iv) CD4.cps v) MregDiff.cps vi) model.props all in the folder where you want the output files to be saved (i.e FolderName).
3. model.props is the parameter file wherein you can change the parameters.
4. run.props and config.props are the configurable files where you can change the number of TICKS (that is a measure of computational time, i.e stop.at = number of TICKS) and the size of the grid (in the current model that is set to 1nm).
5. For running locally, use run.sh
6. To run on a cluster, use job.sh.
7. For the -output folder path, change the CONFIG variable and provide path to your folder i.e /home/username/FolderName.
8. ENISI executable to be used in the job.sh file is located in /PATH: ENISI/ENISI-MSM/bin folder that is created in the (installation step, Section A).
9. Run your job by typing -> sh run.sh (OR) ./ run.sh "path of the folder where you want the results or sh job.sh (specify the CONFIG variable within).
10. After the job iscompleted, you will have .log files, .tsv files for all the compartments.
11. The .log file will contain debugging statements if there are any issued in the code. Additional statments can be added to the source code for confirmation and monitoring the output.
C. Sensitivity Analysis
Stage 1 Initialization
1. Parameters.xlsx -> Contains the maximum and minimum values of the input parameters and information about which parameters are fixed.
2. Generate the Input parameter design matrix (P1) using - design_matrix_generation.m; (NOTE: Comment out the Stage 2 part of the code).
3. Each row in P1 corresponds to the different values of the parameters to be used in the model.props files.
4. Run the simulation using the hybrid computer code as described in Section B.
Stage 1 Analysis
1. Run the simulations (152 x 20 replicates) for each input parameter setting obtained from P1 (see above, step 2 in the initialization stage).
2. Convert the data into .csv file format:
a. 1st column: time points information (i.e. Ticks),
b. 2nd column mean values and
c. 3rd column standard deviations
All the information will be obtained from the ENISI-MSM output runs.
3. Run Stage1-PRCC.ipynb - Formats the data to be used for the PRCC analysis and calculates the PRCC coefficients. (The code generates a data frame with rows from the Parameters.xlsx file and average of the output obtained for that parameter setting in the last column).
4. Plot the PRCC graphs using Stage1-PRCC_barplots.R
5. Alternatively, use Fig3-Code.ipynb jupyter notebook to recreate the figures in the paper.
6. Create an excel sheet with information about the active and inactive inputs from PRCC - PRCC_activeinactiveinputs-added.xlsx.
Stage 2 Initialization
• Generate the Input parameter design matrix (P2) using – i) design_matrix_generation.m (NOTE: Comment out the Stage 1 part of the code) and ii) information regarding the active and inactive inputs present in PRCC_activeinactiveinputs-added.xlsx file.
• Run the simulation using the hybrid computer code as described in Section B.
Stage 2 Analysis
1. Run the simulations (115 x 20 replicates) for each input parameter setting obtained from P2 (see above, step 1 in the initialization stage).
2. Convert the data into .csv file format:
a. 1st column: time points information (i.e. Ticks),
b. 2nd column mean values and
c. 3rd column standard deviations.
All the information will be obtained from the ENISI-MSM output runs.
3. Combine all the outputs obtained from P2 and P1. (outputs obtained after running simulation for P1 from Stage 1, Section C and for P2 from Stage 2, Section C).
Create folders for each of the cell (cells are represented as agents in each compartment) populations and save the files from step 2, Sage 2, Section C.
4. Run Stage2-inputfilegeneration.m and save the output as .mat file to be used to build a temporal metamodel.
5. Build a temporal metamodel using Stage2-BuildTempMM.R and save the output as .Rdata dataset.

6. Calculate the Sobol Indices using Stage2-SA-temporal6tps.R. The input to the code includes the .Rdata obtained from the previous step 6 (stage 2 Analysis, Section C) and the datasets obtained after running SobolIndex_data_generation.m.

References

Mei, Y., V. Abedi, A. Carbo, X. Zhang, P. Lu, C. Philipson, R. Hontecillas, S. Hoops, N. Liles and J. Bassaganya-Riera (2015). "Multiscale modeling of mucosal immune responses." BMC Bioinformatics 16 Suppl 12: S2.

Trevor, H., T. Robert and F. JH (2009). The elements of statistical learning: data mining, inference, and prediction, New York, NY: Springer.

Viladomiu, M., J. Bassaganya-Riera, N. Tubau-Juni, B. Kronsteiner, A. Leber, C. W. Philipson, V. Zoccoli-Rodriguez and R. Hontecillas (2017). "Cooperation of Gastric Mononuclear Phagocytes with Helicobacter pylori during Colonization." J Immunol 198(8): 3195-3204.

Close
