# Supplementary material for: High-resolution computational modeling of immune responses in the gut
Source: Gigascience. 2019 Jun 11;8(6):giz062. doi: 10.1093/gigascience/giz062 (PMC6559340; doi:10.1093/gigascience/giz062)
Supplement: giz062_Response_to_Reviewer_Comments_Revision_1 [file giz062_response_to_reviewer_comments_revision_1.pdf]

Author's Response To Reviewer Comments

Close

Point by point response to the Reviewer reports

We would like to thank the reviewers and editors for taking time to review our manuscript entitled "High-Resolution Computational Modeling of Immune Responses in the Gut" and for providing valuable and constructive criticism. The review process has been helpful in the improvement of our submission. We have considered the comments that were made and have prepared the following point-by-point response. We hope that the revised version of the manuscript can now be accepted for publication. Thanks in advance.

Reviewer #1: The unit of sizes of the model grid can't be right (e.g. grid is 30nm x 10 nm). Animal cells should have measurements in the order of micrometres instead of nanometres. Please check if these are just typos, or do these errors affect any aspect of the simulation, such as diffusion.
Response: We thank the reviewer for pointing this out. We fixed the typos and the unit size of the model grid are 30 µm x 10 µm. These typos do not affect any aspect of the simulations as these units are only annotations and the model takes the numbers as input. We updated the manuscript and fixed the typos throughout the manuscript. Please refer to L120 – L121, and L216, L220-L221.

Reviewer #2: The authors have made significant improvements to the manuscript and thoroughly responded to reviewer comments. One major concern remains surrounding the authors' response to questions around the grid dimensions. The dimensions for the entire grid are given in nm which is smaller than a single cell. Furthermore they state that there are no limits to cell(agent) occupancy per grid compartment. This is rather confusing and calls into question how much spatial information is really contained in this model (e.g. if cytokines are diffusing over the 30nm grid what does that mean for the concentrations that individual cells (measured in micrometers)are seeing?). Based on the author responses it appears that the model is a multi-compartment model with well-mixed discrete agents in each compartment rather than a spatio-temporal model as they claim.
Response: We thank the reviewer for their comment.
We thank the reviewer for pointing out the concern regarding the dimensions of the grid. The correct dimensions of the grid are 30 µm x 10 µm. We updated the manuscript and fixed the typos. Please refer to L120 – L121, and L216, L220-L221.
The mention regarding no limits to cell (agent) occupancy refers to the cells (agents) having no physical size. Further, once a cell (agent) dies it is removed from the simulation to minimize the computational costs of agents that do not contribute to the biology.
The model output contains information about the x and y co-ordinate of the agents at every time point. The cytokines and internal signaling pathways that drive functional fates of cells are well mixed within a cell, i.e., we have only temporal resolution
within the cell during a time step. However, the production, degradation, and diffusions are cell specific thus the cytokine concentration results are also spatio-temporal.
Since, the model is capable of providing information regarding spatial co-ordinates over time, we claim the model to be a spatio-temporal model. We updated the manuscript, please refer to L163-L170.

Please also ensure that your revised manuscript conforms to the journal style, which can be found in the Instructions for Authors on the journal homepage.
Response: The revised manuscript conforms to the journal style.

Close
