# Supplementary material for: High-resolution computational modeling of immune responses in the gut
Source: Gigascience. 2019 Jun 11;8(6):giz062. doi: 10.1093/gigascience/giz062 (PMC6559340; doi:10.1093/gigascience/giz062)
Supplement: giz062_Response_to_Reviewer_Comments_Revision_2 [file giz062_response_to_reviewer_comments_revision_2.pdf]

Author's Response To Reviewer Comments

Close

Once more, we would like to truly thank the reviewers and editor for the time and effort they are putting in reviewing our work. The fact that they've all brought up concerns about the grid dimensions and structure underlines the importance of computational biology as means of integrating biological concepts with biophysics and mathematics. Most biologists tend to focus on functions (i.e., gene expression or protein properties) and numbers, but they rarely consider the dimensions of the "biological space". The proposed revisions and clarifications better connect the biology, underlying mathematics and spatial/biophysical considerations. In response to the comments raised by the reviewers, we have revised the manuscript and provided explanations to further clarify the description of the grid.

Reviewer reports:

Reviewer #2 Comment 1: I have looked through the authors' responses and remain concerned about the grid dimensions. Redefining the grid in um instead of nm still leaves the entire simulation environment at 30um by 10um which is about on the order of a single cell (e.g. macrophage diameter ~20um https://www.ncbi.nlm.nih.gov/pmc/articles/PMC1470168/). I remain concerned that defining the grid at such a small scale creates a biologically unrealistic disconnect between the diffusing cytokines (on a very small scale) and the cells that they influence (on a much larger and abstract scale). I tried to understand the dimensions of the diffusion constants for the cytokines but the parameter table S1 contains no units to be able to compare diffusion scales to cell movement scales.

Response to Reviewer 2, Comment 1: We thank the reviewer for their concern. We agree that defining the grid in μm leaves the simulation in a smaller scale and want to clarify that even though the units in the model are annotations, we understand that it is crucial to define the grid in a more biologically meaning way.

We define the area in the model being simulated as a simulation environment with 30 mm x 10 mm two-dimensional grid. The size of an individual lattice site (previously referred to as grid cell in the paper) is 1 mm x 1mm. The scales described as in the previous version of ENISI-MSM (Mei et al., 2015) were kept unchanged. The table describing the scales used in (Mei et al., 2015) are also shown here in Table 2.
As described in table shown below (adapted from Table 1) of our previous work (Mei et al., 2015), the spatial properties for cytokine diffusion defined in the range of millimeters were unchanged in the version of ENISI-MSM used in this paper.
Scale
Example scenario
Spatial(m)
Time(s)
Technology
Tool
Intra-cellular
Signaling pathways
Nano (nm)
Nano
ODE
COPASI
Cellular
Cell movement and subtype
Milli (mm)
Tens
ABM
ENISI
Intra-cellular

Cytokine-diffusion
Milli (mm)
Tens
PDE
ValueLayer
Tissue
Inflammation and lesions
Centi (cm)
Thousands
Projection
ENISI
Table 2. The four scales of ENISI models, their spatial and temporal properties and modeling technologies and tools used for each scale. (Table 1 as adapted from (Mei et al., 2015))

We updated the manuscript accordingly, please refer to L213-L223.

We thank the reviewer for pointing this out. We updated Table S1 with units for clarity. Lastly, we want to clarify that the model deals with numbers and the units are annotations in the simulation hence the corrections in the dimensions above do not affect the simulation results in any way. All the values used in the code were internally consistent with the model.

Reviewer #3, Comment 1: In this review, I am looking at the more limited questions on the matter of units and scale, which have been raised by both Reviewer #1 and Reviewer #2.

Generally, I don't think the units matter, so long as they have been handled correctly (i.e., with correct conversion and internal consistency within the computational model). The units should be properly labeled in any parameter tables.
I tend to use microns and minutes (or seconds for some problems) for this scale of problem, but there are others who just use cm and sec for everything. As long as the values are correct in the displayed units (and as long as the code used internally consistent values), I think it's purely aesthetic. That said, if something is being labeled on a multicellular level, then labeling 10 microns will be much more appropriate than 10,000 nm.
Response to Reviewer 3, Comment 1: We thank the reviewer for highlighting this point. We concur that the units don’t matter for the simulation results, but want to best clarify the dimensions to make them relevant with the biology. We have labelled all the units in the parameter Table S1. The values used in the code were internally consistent with the model and handled correctly. For further clarity, we show the scales used in the previous ENISI models and the spatial and temporal properties as described in (Mei et al., 2015) and included the Table 1 from (Mei et al., 2015).

Scale
Example scenario
Spatial(m)
Time(s)
Technology
Tool
Intra-cellular
Signaling pathways
Nano (nm)
Nano
ODE
COPASI
Cellular
Cell movement and subtype
Milli (mm)
Tens
ABM
ENISI
Intra-cellular
Cytokine-diffusion
Milli (mm)

Tens
PDE
ValueLayer
Tissue
Inflammation and lesions
Centi (cm)
Thousands
Projection
ENISI
Table 2. The four scales of ENISI models, their spatial and temporal properties and modeling technologies and tools used for each scale. (Adapted from (Mei et al., 2015)).

We updated the manuscript accordingly, please refer to L213-L223.

Reviewer #3, Comment 2: However, I want to further dig into Reviewer #2's concerns on grid sizes and scales.
As I read this draft, I see at least one potential source of confusion: this team appears to be very focused on mathematical and numerical methods. As such, they are using the word "cell" for both a biological cell and a computational lattice site. This is a really bad idea, and the authors should pick a better nomenclature (e.g., computational mesh or lattice site) to avoid this confusion. They should never use "cell" to mean anything other than a biological cell once they enter computational biology.
Response to Reviewer 3, Comment 2: The focus of the paper was to utilize an already published tool (Mei et al., 2015) to study Helicobacter pylori infection. So, a significant focus of the work was on the biology. However, we agree that the nomenclature should be clarified to avoid confusion from using the term “cell” for both actual biological cells and space/grid units. In the revised version of the manuscript we have substituted “grid cell” for “lattice site” and kept “cell” to refer to biological cells.

Reviewer #3, Comment 3: Next, they need to be clearer about what their grids represent. They should show a picture of the domain and meshing in their main text, and not just supplementary material. They should probably also clarify that they are simulating a cross-section of tissue in their model, rather than 3D or some top-down view. (At least from what I can tell.) (They seem to address this in text, but showing the mesh will provide better clarity.)
Response to Reviewer 3, Comment 3: We thank the reviewer for their valuable suggestion regarding this crucial point. We included a cartoon picture of the domain and mesh (as Fig. 2) in the main text as opposed to in the supplementary material (as shown below).

We highlighted that we are simulating a cross-section of tissue in the model and we redefined it as a simulation environment with 30 mm x 10 mm two-dimensional grid.
The size of an individual lattice site (previously referred to as grid cell in the paper) is 1 mm x 1mm.

We updated the manuscript accordingly, please refer to L231-L235.

Reviewer #3, Comment 4: Assuming the authors now have a 30 micron x 10 micron domain, they can simulate at most one epithelial cell, if it's all in plane. But if it's a cross-section, I suppose they could have more. Perhaps many h pylori (which they size at about 1 micron), but not many mammalian cells. So, their computational domain is still not very clear to me, and they should just show it, with appropriate labeling. They seem to skip straight to population dynamics in their figures, but it would be very helpful if they showed one actual spatiotemporal simulation. This would make the nature and performance of their model much clearer.
Response to Reviewer 3, Comment 4: We thank the reviewer for their concern.
With a redefined computational simulation environment of 30 mm x 10 mm, the epithelium is comprised of hundreds of epithelial cells.
For example, if the initial number of epithelial cells defined by the user is 12, the total number of epithelial cells amounts to = (30 x 1) dimension of epithelial compartment x 12intial number = 360.

In addition to the figure of the grid environment, we included the screenshots of one actual in silico simulation of H. pylori infection to highlight the spatiotemporal aspects of the modeling outputs. The screenshots were created using VisIt version 2.12 (Childs et al., 2012), an interactive visualization and analysis tool. As shown in Additional file Fig. S2 the screenshots represent the spatial distribution of

different agent cells over time points (2, 4, 5 and 6) distributed across the 2D grid. Further, we presented the insets in Fig. S2 showing a zoomed in portion of the respective grids across the time steps 2, 4, 5 and 6.

We also want to clarify that the agents represented in the screenshots below are only for visual representation and do not represent the actual size of the biological cells.

We updated the manuscript accordingly and added Fig 3, please refer to L333-L338.

Fig S2. Time screenshots of a Helicobacter pylori infection modeled in a 30 mm (length) x 10 mm (width) two-dimensional grid. The thickness of the compartment is shown on the y-axis, such that: lumen spans (0 to 2) units, epithelium spans (2 to 3) units, lamina propria spans (3 to 8) units and gastric lymph node across (8-10) units on the scale. Two-dimensional distribution of different cell subsets over the time steps (ticks) 2, 4 (top panels), 5 and 6 (bottom panels) are shown. The insets in each image shows a zoomed in portion of the respective grids across the time steps 2, 4, 5 and 6. The agents represented in the screenshots below are only for visual representation and do not represent the actual size of the biological cells.
Future refinements of the model will create agents of the actual sizes of cells.

Reviewer #3, Comment 5: On sizes and scaling, I fully agree with Reviewer #2: if this is indeed a 30 micron x 10 micron cross section, there's no way there are more than a handful of mammalian cells at any time in any simulation. If they have made a scaling argument (and there are such arguments that could be made if formulated clearly and rigorously), they'd better be clear about it. Any results that show thousands of mammalian cells in a 30 x 10 micron^2 domain are simply beyond biophysical plausibility.

Response Reviewer 3, Comment 5: We defined the simulation environment as a 30 mm x 10 mm two-dimensional grid that represents a cross section area of stomach tissue modeled here.

Reviewer #3, Comment 6: Again, just actually showing a simulation (either a movie, or some time snapshots, but showing locations of all the cell agents and substrate distributions) would help clarify things much more. No limit to the number of cells in a mesh site, while mathematically possible, does not make sense on such a small simulated domain. Even though the authors treated the cell agents as no size (infinitesimal points), there are physical limits, and moreover if each cell is absorbing / secreting things at appropriate rates, then there should be ridiculous amounts of secretion of growth factors and ridiculous depletion of growth substrates, if there is a huge overcrowding of hundreds or thousands of mammalian cells in a 1 micron x 1 micron lattice site.

Response to Reviewer 3, Comment 6: We thank the reviewer for their valuable suggestion and have included time snapshots for the simulation (time points 2, 4, 5 and 6) created using VisIt version 2.12 (Childs et al., 2012), an interactive visualization and analysis tool. Please refer to Additional file Fig S2 also included in the above response to Comment 5.
The hybrid multiscale modeling platform ENISI MSM is currently capable of scaling up to up to 109 agents, at which the memory (on a 32 GB node) was exceeded due to the large number of agents.

Reviewer #3, Comment 7: If that's what's going on, the authors really do need to take a step back and consider domains sufficiently large to capture hundreds or thousands of mammalian cells. This is not simply a matter of relabeling axes: it's a matter of simulating a larger physical domain that is suited to the size of objects (mammalian cells) that they are considering, with biophysically reasonable parameters. The authors need to carefully review all their parameters (e.g., cell densities) to ensure they are correctly scaled and reasonable. If there is nobody on the team with sufficient domain expertise to review these parameters and results to check for reasonableness, it may be time to grow the team. Given that actual mammalian cells are much larger than the computational lattice sites, there must be constraints: if a lattice site is "occupied" by a mammalian cells, so are many (or most!) of the surrounding lattice sites. They would be no-fly zones for further mammalian cells. The common way to solve such problems is to use computational lattice sites that are of comparable size to the largest

biological cells (e.g., 1 biological cell per lattice site, as a cellular automaton model), or use very large lattice sites (e..g, 100 micron x 100 micron) that can truly contain multiple cells.

Response to Reviewer 3, Comment 7: We thank the reviewer for their suggestion on the size of the lattice site.
In the current model, the size of the lattice site (referred as spatial grid previously) is 1mm x 1mm, capable of containing multiple cells.
The total number of agents in a compartment of size (length x width) is calculated as follows: number (agents) x size (compartment),
For example, if the user set the initial number of epithelial cell agents to be 12, the total number of epithelial agents within the epithelium compartment amounts to -
12initial_number x (30 x 1)size_epithelilum_compartment = 360.

Reviewer #3, Comment 8: All is not lost, however. If for some reason that larger domain is computationally infeasible (hard to imagine), the authors really don't need a 1 micron mesh resolution for most of the effects here. The diffusion length scale of most chemokines and diffusing substrates would not require a 1 micron mesh resolution. (And numerical stability will improve if coarser mesh resolutions are used.) If the authors feel a 1 micron mesh is needed for the bacteria, they could easily use a separate mesh. This work looks interesting. I think the team has a great contribution to make, if they pay a bit closer attention to the biophysical limits of their system.

Response to Reviewer 3, Comment 8: The computational domain used here is of 30 x 10 units in size and the individual lattice site is 1 units for the simulation. The lattice site is a configurable run parameter and can be changed without modifying the model.

We thank the reviewer for their valuable suggestions, inputs and concerns and have tried to clarify the questions around the grid dimensions.

With these revisions and clarifications, we believe that the revised manuscript is acceptable for publication.

Thank you for considering this work.

References:

Childs, H., Brugger, E., Whitlock, B., Meredith, J., Ahern, S., Pugmire, D., …Weber, G. (2012). VisIt: An End-User Tool For Visualizing and Analyzing Very Large Data. High Performance Visualization-Enabling Extreme-Scale Scientific Insight. Insight, 357-372.
Mei, Y., Abedi, V., Carbo, A., Zhang, X., Lu, P., Philipson, C., ..Bassaganya-Riera, J. (2015). Multiscale modeling of mucosal immune responses. BMC Bioinformatics, 16 Suppl 12, S2. doi:10.1186/1471-2105-16-s12-s2

Close
