# Supplementary material for: High-resolution computational modeling of immune responses in the gut
Source: Gigascience. 2019 Jun 11;8(6):giz062. doi: 10.1093/gigascience/giz062 (PMC6559340; doi:10.1093/gigascience/giz062)
Supplement: giz062_Response_to_Reviewer_Comments_Revision_3 [file giz062_response_to_reviewer_comments_revision_3.pdf]

Author's Response To Reviewer Comments

Close

Point by point response to reviewers
Reviewer #3: I appreciate the authors' efforts to revise their manuscript to address the editorial and review concerns. However, I'm a little concerned.
This is the second time the authors have "redefined" what their lattice sites represent. This is a not just a matter of crossing out micrometers and writing millimeters. Either the original simulations were actually 30 mm x 10 mm and they just mislabeled twice, or the original simulation was performed on the wrong domain size and the simulations now need to be rerun on the correct 30 mm x 10 mm grid.
I'd like the authors to clearly answer which correction matches reality for this submission:
A) The simulations were originally performed on a 30 nm x 10 nm domain as in the original simulation, but they have now re-run all simulations on a correct 30 mm x 10 mm grid and updated all the results, figures, and as needed, conclusions.
B) The simulations were original performed on a 30 micron x 10 micron domain as in the resubmission, but they have now re-run all simulations on a correct 30 mm x 10 mm grid and updated all the results figures, and as needed, conclusions.
C) The simulations were originally performed on a 30 mm x 10 mm domain, and they were mislabeled twice but at last are correctly labeled now. They have verified and rechecked all code and configuration settings that the simulation runs truly correspond mathematically to a 30 mm x 10 mm domain.
D) Something else that they 100% clearly state, rather than thanking us and redefining axes again.
The reason we need to be careful on this is that in numerics packages, changing a simulation previously run on a small domain to one now corresponding to a big domain is almost never a simple matter of relabeling the prior plots. Rescaling axes without changing the data is equivalent to changing the diffusion coefficient (and other parameters).
Either the original and resubmitted labels were wrong, and they have now corrected. Or their original units were correctly stated, the domain size was wrong, and they must correct by rerunning the simulations on the correct domain. Or space was nondimensionalized, and all the parameters were internally represented in units of lattice sites instead of physical units. (e.g., diffusion coefficients in length units^2 / time units). (But this strikes me as less likely.)
If I simulate a city block with unrealistic parameters, it doesn't automatically become a correct simulation of the entire city by just relabeling axes. The statements about just relabeling units, as well as relying upon "configurable run parameters", gives me pause to be a little cautious before accepting.
An explicit clarification on (A)-(D) (or other) will be helpful. I think the results are probably fine. But I want to be sure, and not just probably fine.
Response: We want to clarify that all the simulations and results were obtained with no units. In the first version of the submitted manuscript, we described the model as a region with a 30 x 10 grid, similar to the area defined in (Mei et al. 2015) as a square region with 100 x 100 2D grid cells. Over the course of the reviews, in order to better describe the model, we included the units. Nevertheless, the simulation runs corresponded mathematically to a 30 mm x 10 mm domain. The numbers (describing the dimension of the compartments) that represent a region being modeled and used to obtain all the simulation results and conclusions; corresponded to ‘mm’ units. Thus, there were no changes equivalent to changing the diffusion coefficient since the original units modeled were mm.
The units in the model were annotations and purely aesthetic to provide a closer biological meaning. The change in the units did not affect the simulation results.
To support our claim that the change in the units (annotations) did not affect the simulations results, we ran the simulations for a Wild type (WT) scenario with - i) no units (black dot-dashed line), ii) nanometer (‘nm’ - green solid line), iii) micrometer (‘μm’ - red dashed line) and iv) millimeter (‘mm’ - blue solid line) as units. The figure below shows: (a) the number of Helicobacter pylori agents over time, and b) number of resident macrophages agents over time. There was no statistically significant difference ($P > 0.05$), observed between the groups for both the cell populations (see - c) and d)).

Also, now that I'm looking through the GitHub repo for the project, I'd like to see a clearer statement on which parameter files to use when running to reproduce the specific results in this paper. If any

additional scripts or configuration files are needed to create the figures in this paper (e.g., parameter sweeps), they should include them somewhere in the github repo with clear instructions. (The instructions are presently a bit vague.)
Response: We included and clarified which parameter files were used to reproduce the results in the README.mkd of the repo for the project (see below pages 3-5). The parameter values were also listed in Table S1.
The scripts needed to create all the figures presented in the paper were provided in the form of Jupyter notebooks (in the FTP directory of Gigascience). The scripts and other processing files and jupyter files are now included in the 'Processing' folder on the GitHub repository as well. The detailed instructions (scripts and files) to reproduce the figures in the paper are included in the GitHub repository ('Processing/Figures'). The instructions were also in listed in the File S1 of the paper (see README.mkd (on pages 3-6) below).
Thank you. I think with a little more clarification, this paper will be acceptable for publication and a great contribution. But relabeling plot axes twice without rerunning anything makes me nervous, and I need more clarity to give a green light.
Response: To summarize, we did not use the units to run the simulations and results, however, the simulation runs corresponded mathematically to a 'mm' domain. To better describe the model, we included units in the consecutive revisions.
We re-ran the simulations for four (WT) scenarios - i) no units, ii) nanometer ('nm'), iii) micrometer ('μm') and iv) millimeter ('mm') as units and demonstrated that the simulations results did not change because the units in the model are annotations.
We believe that with these clarifications the paper should be acceptable for publication.
References:
Mei Y, Abedi V, Carbo A, Zhang X, Lu P, Philipson C, Hontecillas R, Hoops S, Liles N, Bassaganya-Riera J. Multiscale modeling of mucosal immune responses. BMC bioinformatics. 2015 Dec;16(12):S2.

README.mkd on the GitHub repository -
The text includes detailed instructions regarding - i) installing ENISI-MSM and it's dependencies, ii) running the program on a local system and on a server and iii) scripts used for the processing of the outputs generated from the code. Additionally, README.mkd in the /ENISI-MSM/Howtorunasimulation and /ENISI-MSM/Sensitivity-Analysis folders also provides detailed instructions on running simulation and sensitivity analysis respectively.
Installation
```
mkdir ENISI
cd ENISI
git clone https://github.com/NIMML/ENISI-Dependencies
git clone https://github.com/NIMML/ENISI-MSM
```
Building ENISI-Dependencies
```
cd "path-to-ENISI"/ENISI-Dependencies
mkdir build
cd build
cmake ../
make
```
Building ENISI-MSM
```
cd "path-to-ENISI"/ENISI-MSM
mkdir build
cd build
cmake -DENISI_MSM_DEPENDENCY="path-to-ENISI"/ENISI-DEPENDENCIES/install" ..
make
```
Run the program
Running on the local system
1. Change the paths below in the run.sh file (can be located in the "path-to-ENISI"/ENISI-

MSM/Howtorunasimulationfolder).
o Path for mpirun in run.sh to be changed to your "path-to-ENISI"/ENISI-Dependencies/install/bin/ directory.
o Path for ENISI-MSM executable to be changed to the location of your ~/ENISI/ENISI-MSM/bin/ directory
2. Create a folder where the output files are to be saved (for e.g. OutputFolder) with the following file contents :
o config.props, run.props, model.props, CD4.cps and MregDiff.cps (All included in the "path-to-ENISI"/ENISI-MSM/Howtorunasimulation folder).
o Configurable parameter file - model.props.
o run.props and config.props are the configurable files where you can change -
▪ Number of TICKS (that is a measure of computational time, i.e stop.at = number of TICKS)
▪ Size of the grid cell.
3. Run the executable -
../run.sh "path-to-OutputFolder"
Running on server
1. Install and build ENISI-Dependencies and ENISI-MSM on the server.
2. Create a folder to run the simulation and store the output files (for e.g. OutputFolder). The contents include:
o config.props, run.props, model.props, CD4.cps and MregDiff.cps and job.sh files (All included in the "path-to-ENISI"/ENISI-MSM/Howtorunasimulation folder).
o Configurable parameter file - model.props.
o The run.props and config.props are the files where you can change -
▪ Number of TICKS (that is a measure of computational time, i.e stop.at = number of TICKS)
▪ Size of the grid cell.
o The path-to-OutputFolder is provided in the CONFIG variable specified in the job.sh file.
3. Run the executable.
sh job.sh
Scripts
All the scripts and parameter sets are listed in "path-to-ENISI"/ENISI-MSM/Processing folder.
Parameter sets
• "path-to-ENISI"/ENISI-MSM/Processing/ParameterSets folder contains the parameter files (model.props) to be used when running the program (refer to 'Run the program' section above) to reproduce the files used to plot the results in the paper (currently under review).
• The files are named as model_*.props where "*" represents the different condition.
• When running each simulation for the different condition, rename the file to model.props.
Code for figures in the paper
• All the jupyter notebooks that create the figure in the paper are provided in the "path-to-ENISI"/ENISI-MSM/Processing/Figures.
• The files are named as Fig*_Code.ipynb where "*" represents the figure number.
Other scripts
The bash and python scripts are provided in the "path-to-ENISI"/ENISI-MSM/Processing/Others folder. Each script has a comment section that decribes the usage, purpose and required location of the script. The folder structure for the below scrips are as follows:
~/alloutputs/allRuns/setting0/run0
• The alloutputs folder contains the collections of all outputs.
• The allRuns folder (inside the alloutputs folder) contains the settings folder.
• The setting folder corresponds to a different set of parameters.
• The run folder corresponds to the replicates (for e.g. 10) for individual parameter set. (The run folder is similar to the folder created in Step 2 of running the jobs locally and on the server. The run folders include all the files provided in the "path-to-ENISI"/ENISI-MSM/Howtorunasimulation folder)
i. lp_code.py (can be located in any folder; the "path-to-lp_code.py" is required by the tsvcsv.sh).
ii. tsvcsv.sh (to be located in ~/alloutputs/).
iii. average_and_SD.py (to be located in ~/alloutputs/allRuns/setting0/).
The scripts for Sensitivity-Analysis and the steps are detailed in "path-to-ENISI"/ENISI-MSM/Sensitivity-Analysis/.

Close
