# Supplementary material for: High-resolution computational modeling of immune responses in the gut
Source: Gigascience. 2019 Jun 11;8(6):giz062. doi: 10.1093/gigascience/giz062 (PMC6559340; doi:10.1093/gigascience/giz062)
Supplement: giz062_Reviewer_1_Report_Original_Submission — Chang Gong -- 12/9/2018 Reviewed [file giz062_reviewer_1_report_original_submission.pdf]

Reviewer Report

Title: High-Resolution Computational Modeling of Immune Responses in the Gut

Version: Original Submission **Date:** 12/9/2018

Reviewer name: Chang Gong

Reviewer Comments to Author:

In this manuscript, the authors constructed a multi-scale systems biology model of Helicobacter pylori infection to study the interaction between bacterial infection and the immune system. Some modifications could be considered to improve the quality of this manuscript:

1. The model needs to be more clearly described in the text. Some details might be available from the code; nevertheless, it would be helpful for readers to understand if the authors can include more information regarding the model. For example:

a. Agent-based model:

i. What is the spatial discretization? The authors mentioned it's a 30*10 2D grid cell, but resident macrophages are in thousands. So multiple cells are allowed in the same grid location? How many?

ii. What is the time step size?

iii. How is migration implemented for cells and bacteria agents?

b. ODE: What's the COPASI setup for the solver? How is the solver in sync with the ABM?

c. PDE: What package and numerical scheme is used to solve the PDEs? What's the setting?

2. The authors listed the values of parameters in Table S1.

a. However, it's not clear what their units are (the baseline column seems to include characters such as "l^2", "#" or "d". are these units? Please clarify).

b. Also, the sources of the parameter values are not very clear, except for the vague statement "expert judgement" (Saltelli, Tarantola et al. 2000 is cited, but this is an article on SA and does not contain parameters).

c. Please in the table explain what mechanism each parameter corresponds to. Some can be inferred from the name, but it's not very clear.

d. Some parameters are not included in the table. For example, the diffusivity of the cytokines are not listed.

3. In Table 1 and Table 2, there is a T cell class named "Tr", which is not explained in the text. Please clarify.

4. The authors used a Gaussian emulator as surrogate model for the hybrid model. In line 582, the authors mentioned that performance is evaluated using diagnostic plots in Figure S4. Please clarify what the "Observed" data refers to. Are these the same simulations from the training set which the emulator fitted to, or are these new simulations done? If these are the training set results, the authors need to run simulations and emulation on a new testing set and evaluate the performance; if it's already done, please clarify how its done (range of parameters, number of simulations, etc.)

Methods

Are the methods appropriate to the aims of the study, are they well described, and are necessary controls included? Choose an item.

Conclusions

Are the conclusions adequately supported by the data shown? Choose an item.

Reporting Standards

Does the manuscript adhere to the journal's guidelines on minimum standards of reporting? Choose an item.

Choose an item.

Statistics

Are you able to assess all statistics in the manuscript, including the appropriateness of statistical tests used? Choose an item.

Quality of Written English

Please indicate the quality of language in the manuscript: Choose an item.

Declaration of Competing Interests

Please complete a declaration of competing interests, considering the following questions:

- Have you in the past five years received reimbursements, fees, funding, or salary from an organisation that may in any way gain or lose financially from the publication of this manuscript, either now or in the future?
- Do you hold any stocks or shares in an organisation that may in any way gain or lose financially from the publication of this manuscript, either now or in the future?
- Do you hold or are you currently applying for any patents relating to the content of the manuscript?
- Have you received reimbursements, fees, funding, or salary from an organization that holds or has applied for patents relating to the content of the manuscript?
- Do you have any other financial competing interests?
- Do you have any non-financial competing interests in relation to this paper?

If you can answer no to all of the above, write 'I declare that I have no competing interests' below. If your reply is yes to any, please give details below.

I declare that I have no competing interests

I agree to the open peer review policy of the journal. I understand that my name will be included on my report to the authors and, if the manuscript is accepted for publication, my named report including any attachments I upload will be posted on the website along with the authors' responses. I agree for my report to be made available under an Open Access Creative Commons CC-BY license (http://creativecommons.org/licenses/by/4.0/). I understand that any comments which I do not wish to be included in my named report can be included as confidential comments to the editors, which will not be published.

I agree to the open peer review policy of the journal

To further support our reviewers, we have joined with Publons, where you can gain additional credit to further highlight your hard work (see: https://publons.com/journal/530/gigascience). On publication of this paper, your review will be automatically added to Publons, you can then choose whether or not to claim your Publons credit. I understand this statement.

Yes Choose an item.
