# Supplementary material for: High-resolution computational modeling of immune responses in the gut
Source: Gigascience. 2019 Jun 11;8(6):giz062. doi: 10.1093/gigascience/giz062 (PMC6559340; doi:10.1093/gigascience/giz062)
Supplement: giz062_Reviewer_2_Report_Original_Submission — Elsje Pienaar -- 12/17/2018 Reviewed [file giz062_reviewer_2_report_original_submission.pdf]

Reviewer Report

Title: High-Resolution Computational Modeling of Immune Responses in the Gut

Version: Original Submission **Date:** 12/17/2018

Reviewer name: Elsje Pienaar

Reviewer Comments to Author:

The authors present results from a multi-scale hybrid model of host immune responses to H pylori exposure in the gut. The paper addresses outstanding questions in this complex system and overall the results are interesting. Some comments/questions to be addressed are outlined below.

1. A key component of the introduction ("double edge sword, p 1 line 4") as well as in the discussion (p28 line 672 "dual role as pathogen and beneficial organism") mentions the conflicting roles of H pylori infection - however the results do not clearly connect to help answer this dichotomy. More detailed analysis/discussion of the results should be provided to clarify the conclusion or the focus of the intro/discussion should be adjusted to relate more closely to the results currently presented.

2. Section 3.4 and p 29 line 694 discuss the involvement of regulatory macrophages and tolerogenic DCs on the colonization of H pylori. These conclusions appear to be drawn based on correlation between responses in H pylori and macrophage/DC populations upon epithelial cell proliferation adjustment (Fig 5). A causal connection between the macrophages/DCs and H pylori is not made (or is not clear to me from the text). If such a connection is embedded in the mechanisms included in Table 1 it should be outlined in the results section where the conclusion is made otherwise simulations targeting the macrophage/DC populations would be needed to confirm this hypothesis

3. Clarity is needed on some parts of the methods description:

3.1 P6, line 131: what are the units of the grid dimensions given. Are these the dimensions of a single grid cell or the entire grid? How are the 4 compartments separated on the grid?

3.2 P6 line 149: what data were the ODEs calibrated to? Is there a reference?

3.3 P6 line 150, and p22 line 524: ABM parameters were calibrated to "qualitatively resemble" the patterns observed in in vivo model. What patterns? What is considered to be qualitatively similar enough? Do the simulations reproduce the dynamics as well and the endpoint experimental observations? Inclusion of experimental data alongside the simulations in figure 2 or a description of the key dynamics (e.g. fold-changes, peak values etc.) would go a long way in communicating confidence in the model parameters.

3.4 P11 line 246: the authors state that they perform global SA of the hybrid computer model. I believe they mean the metamodel here?

3.5 P 21 line 480 and 484: parameter values were 'reduced' to emulate biological KOs. By how much were the parameters reduced?

3.6 The in vivo model is mentioned several times before it is clarified to be a mouse model.

Methods

Are the methods appropriate to the aims of the study, are they well described, and are necessary controls included? Choose an item.

Conclusions

Are the conclusions adequately supported by the data shown? Choose an item.

Reporting Standards

Does the manuscript adhere to the journal's guidelines on minimum standards of reporting? Choose an item.

Choose an item.

Statistics

Are you able to assess all statistics in the manuscript, including the appropriateness of statistical tests used? Choose an item.

Quality of Written English

Please indicate the quality of language in the manuscript: Choose an item.

Declaration of Competing Interests

Please complete a declaration of competing interests, considering the following questions:

- Have you in the past five years received reimbursements, fees, funding, or salary from an organisation that may in any way gain or lose financially from the publication of this manuscript, either now or in the future?
- Do you hold any stocks or shares in an organisation that may in any way gain or lose financially from the publication of this manuscript, either now or in the future?
- Do you hold or are you currently applying for any patents relating to the content of the manuscript?
- Have you received reimbursements, fees, funding, or salary from an organization that holds or has applied for patents relating to the content of the manuscript?
- Do you have any other financial competing interests?
- Do you have any non-financial competing interests in relation to this paper?

If you can answer no to all of the above, write 'I declare that I have no competing interests' below. If your reply is yes to any, please give details below.

I declare that I have no competing interests

I agree to the open peer review policy of the journal. I understand that my name will be included on my report to the authors and, if the manuscript is accepted for publication, my named report including any attachments I upload will be posted on the website along with the authors' responses. I agree for my

report to be made available under an Open Access Creative Commons CC-BY license (http://creativecommons.org/licenses/by/4.0/). I understand that any comments which I do not wish to be included in my named report can be included as confidential comments to the editors, which will not be published.

I agree to the open peer review policy of the journal

To further support our reviewers, we have joined with Publons, where you can gain additional credit to further highlight your hard work (see: https://publons.com/journal/530/gigascience). On publication of this paper, your review will be automatically added to Publons, you can then choose whether or not to claim your Publons credit. I understand this statement.

Yes Choose an item.
