# Supplementary material for: High-resolution computational modeling of immune responses in the gut
Source: Gigascience. 2019 Jun 11;8(6):giz062. doi: 10.1093/gigascience/giz062 (PMC6559340; doi:10.1093/gigascience/giz062)
Supplement: giz062_Reviewer_3_Report_Revision_2 — Paul Macklin, Ph.D. -- 2/25/2019 Reviewed [file giz062_reviewer_3_report_revision_2.pdf]

Reviewer Report

Title: High-Resolution Computational Modeling of Immune Responses in the Gut

Version: Revision 2 **Date:** 2/25/2019

Reviewer name: Paul Macklin, Ph.D.

Reviewer Comments to Author:

In this review, I am looking at the more limited questions on the matter of units and scale, which have been raised by both Reviewer #1 and Reviewer #2.

Generally, I don't think the units matter, so long as they have been handled correctly (i.e., with correct conversion and internal consistency within the computational model). The units should be properly labeled in any parameter tables. I tend to use microns and minutes (or seconds for some problems) for this scale of problem, but there are others who just use cm and sec for everything. As long as the values are correct in the displayed units (and as long as the code used internally consistent values), I think it's purely aesthetic. That said, if something is being labeled on a multicellular level, then labeling 10 microns will be much more appropriate than 10,000 nm.

However, I want to further dig into Reviewer #2's concerns on grid sizes and scales.

As I read this draft, I see at least one potential source of confusion: this team appears to be very focused on mathematical and numerical methods. As such, they are using the word "cell" for both a biological cell and a computational lattice site. This is a really bad idea, and the authors should pick a better nomenclature (e.g., computational mesh or lattice site) to avoid this confusion. They should never use "cell" to mean anything other than a biological cell once they enter computational biology.

Next, they need to be clearer about what their grids represent. They should show a picture of the domain and meshing in their main text, and not just supplementary material. They should probably also clarify that they are simulating a cross-section of tissue in their model, rather than 3D or some top-down view. (At least from what I can tell.) (They seem to address this in text, but showing the mesh will provide better clarity.)

Assuming the authors now have a 30 micron x 10 micron domain, they can simulate at most one epithelial cell, if it's all in plane. But if it's a cross-section, I suppose they could have more. Perhaps many h pylori (which they size at about 1 micron), but not many mammalian cells. So, their computational domain is still not very clear to me, and they should just show it, with appropriate labeling. They seem to skip straight to population dynamics in their figures, but it would be very helpful if they showed one actual spatiotemporal simulation. This would make the nature and performance of their model much clearer.

On sizes and scaling, I fully agree with Reviewer #2: if this is indeed a 30 micron x 10 micron cross section, there's no way there are more than a handful of mammalian cells at any time in any simulation. If they have made a scaling argument (and there are such arguments that could be made if formulated clearly and rigorously), they'd better be clear about it. Any results that show thousands of mammalian cells in a 30 x 10 micron^2 domain are simply beyond biophysical plausibility.

Again, just actually showing a simulation (either a movie, or some time snapshots, but showing locations

of all the cell agents and substrate distributions) would help clarify things much more. No limit to the number of cells in a mesh site, while mathematically possible, does not make sense on such a small simulated domain. Even though the authors treated the cell agents as no size (infinitesimal points), there are physical limits, and moreover if each cell is absorbing / secreting things at appropriate rates, then there should be ridiculous amounts of secretion of growth factors and ridiculous depletion of growth substrates, if there is a huge overcrowding of hundreds or thousands of mammalian cells in a 1 micron x 1 micron lattice site.

If that's what's going on, the authors really do need to take a step back and consider domains sufficiently large to capture hundreds or thousands of mammalian cells. This is not simply a matter of relabeling axes: it's a matter of simulating a larger physical domain that is suited to the size of objects (mammalian cells) that they are considering, with biophysically reasonable parameters. The authors need to carefully review all their parameters (e.g., cell densities) to ensure they are correctly scaled and reasonable. If there is nobody on the team with sufficient domain expertise to review these parameters and results to check for reasonableness, it may be time to grow the team.

Given that actual mammalian cells are much larger than the computational lattice sites, there must be constraints: if a lattice site is "occupied" by a mammalian cells, so are many (or most!) of the surrounding lattice sites. They would be no-fly zones for further mammalian cells. The common way to solve such problems is to use computational lattice sites that are of comparable size to the largest biological cells (e.g., 1 biological cell per lattice site, as a cellular automaton model), or use very large lattice sites (e..g, 100 micron x 100 micron) that can truly contain multiple cells.

All is not lost, however. If for some reason that larger domain is computationally infeasible (hard to imagine), the authors really don't need a 1 micron mesh resolution for most of the effects here. The diffusion length scale of most chemokines and diffusing substrates would not require a 1 micron mesh resolution. (And numerical stability will improve if coarser mesh resolutions are used.) If the authors feel a 1 micron mesh is needed for the bacteria, they could easily use a separate mesh.

This work looks interesting. I think the team has a great contribution to make, if they pay a bit closer attention to the biophysical limits of their system.

Methods

Are the methods appropriate to the aims of the study, are they well described, and are necessary controls included? Choose an item.

Conclusions

Are the conclusions adequately supported by the data shown? Choose an item.

Reporting Standards

Does the manuscript adhere to the journal’s guidelines on [minimum standards of reporting?]() Choose an item.

Choose an item.

Statistics

Are you able to assess all statistics in the manuscript, including the appropriateness of statistical tests used? Choose an item.

Quality of Written English

Please indicate the quality of language in the manuscript: Choose an item.

Declaration of Competing Interests

Please complete a declaration of competing interests, considering the following questions:

- Have you in the past five years received reimbursements, fees, funding, or salary from an organisation that may in any way gain or lose financially from the publication of this manuscript, either now or in the future?
- Do you hold any stocks or shares in an organisation that may in any way gain or lose financially from the publication of this manuscript, either now or in the future?
- Do you hold or are you currently applying for any patents relating to the content of the manuscript?
- Have you received reimbursements, fees, funding, or salary from an organization that holds or has applied for patents relating to the content of the manuscript?
- Do you have any other financial competing interests?
- Do you have any non-financial competing interests in relation to this paper?

If you can answer no to all of the above, write 'I declare that I have no competing interests' below. If your reply is yes to any, please give details below.

I declare that I have no competing interests.

I agree to the open peer review policy of the journal. I understand that my name will be included on my report to the authors and, if the manuscript is accepted for publication, my named report including any attachments I upload will be posted on the website along with the authors' responses. I agree for my report to be made available under an Open Access Creative Commons CC-BY license (http://creativecommons.org/licenses/by/4.0/). I understand that any comments which I do not wish to be included in my named report can be included as confidential comments to the editors, which will not be published.

I agree to the open peer review policy of the journal

To further support our reviewers, we have joined with Publons, where you can gain additional credit to further highlight your hard work (see: https://publons.com/journal/530/gigascience). On publication of this paper, your review will be automatically added to Publons, you can then choose whether or not to claim your Publons credit. I understand this statement.

Yes Choose an item.
