# Supplementary material for: High-resolution computational modeling of immune responses in the gut
Source: Gigascience. 2019 Jun 11;8(6):giz062. doi: 10.1093/gigascience/giz062 (PMC6559340; doi:10.1093/gigascience/giz062)
Supplement: giz062_Reviewer_3_Report_Revision_3 — Paul Macklin, Ph.D. -- 3/21/2019 Reviewed [file giz062_reviewer_3_report_revision_3.pdf]

Reviewer Report

Title: High-Resolution Computational Modeling of Immune Responses in the Gut

Version: Revision 3 **Date:** 3/21/2019

Reviewer name: Paul Macklin, Ph.D.

Reviewer Comments to Author:

I appreciate the authors' efforts to revise their manuscript to address the editorial and review concerns. However, I'm a little concerned.
This is the second time the authors have "redefined" what their lattice sites represent.
This is a not just a matter of crossing out micrometers and writing millimeters. Either the original simulations were actually 30 mm x 10 mm and they just mislabeled twice, or the original simulation was performed on the wrong domain size and the simulations now need to be rerun on the correct 30 mm x 10 mm grid.
I'd like the authors to clearly answer which correction matches reality for this submission:
A) The simulations were originally performed on a 30 nm x 10 nm domain as in the original simulation, but they have now re-run all simulations on a correct 30 mm x 10 mm grid and updated all the results, figures, and as needed, conclusions.
B) The simulations were original performed on a 30 micron x 10 micron domain as in the resubmission, but they have now re-run all simulations on a correct 30 mm x 10 mm grid and updated all the results figures, and as needed, conclusions.
C) The simulations were originally performed on a 30 mm x 10 mm domain, and they were mislabeled twice but at last are correctly labeled now. They have verified and rechecked all code and configuration settings that the simulation runs truly correspond mathematically to a 30 mm x 10 mm domain.
D) Something else that they 100% clearly state, rather than thanking us and redefining axes again.
The reason we need to be careful on this is that in numerics packages, changing a simulation previously run on a small domain to one now corresponding to a big domain is almost never a simple matter of relabeling the prior plots. Rescaling axes without changing the data is equivalent to changing the diffusion coefficient (and other parameters).
Either the original and resubmitted labels were wrong, and they have now corrected. Or their original units were correctly stated, the domain size was wrong, and they must correct by rerunning the simulations on the correct domain. Or space was nondimensionalized, and all the parameters were internally represented in units of lattice sites instead of physical units. (e.g., diffusion coefficients in length units^2 / time units). (But this strikes me as less likely.)
If I simulate a city block with unrealistic parameters, it doesn't automatically become a correct simulation of the entire city by just relabeling axes. The statements about just relabeling units, as well as relying upon "configurable run parameters", gives me pause to be a little cautious before accepting.
An explicit clarification on (A)-(D) (or other) will be helpful. I think the results are probably fine. But I want to be sure, and not just probably fine.
Also, now that I'm looking through the GitHub repo for the project, I'd like to see a clearer statement on

which parameter files to use when running to reproduce the specific results in this paper. If any additional scripts or configuration files are needed to create the figures in this paper (e.g., parameter sweeps), they should include them somewhere in the github repo with clear instructions. (The instructions are presently a bit vague.)
Thank you. I think with a little more clarification, this paper will be acceptable for publication and a great contribution. But relabeling plot axes twice without rerunning anything makes me nervous, and I need more clarity to give a green light.

Methods

Are the methods appropriate to the aims of the study, are they well described, and are necessary controls included? Choose an item.

Conclusions

Are the conclusions adequately supported by the data shown? Choose an item.

Reporting Standards

Does the manuscript adhere to the journal's guidelines on [minimum standards of reporting?](#) Choose an item.

Choose an item.

Statistics

Are you able to assess all statistics in the manuscript, including the appropriateness of statistical tests used? Choose an item.

Quality of Written English

Please indicate the quality of language in the manuscript: Choose an item.

Declaration of Competing Interests

Please complete a declaration of competing interests, considering the following questions:

- Have you in the past five years received reimbursements, fees, funding, or salary from an organisation that may in any way gain or lose financially from the publication of this manuscript, either now or in the future?
- Do you hold any stocks or shares in an organisation that may in any way gain or lose financially from the publication of this manuscript, either now or in the future?
- Do you hold or are you currently applying for any patents relating to the content of the manuscript?

- Have you received reimbursements, fees, funding, or salary from an organization that holds or has applied for patents relating to the content of the manuscript?
- Do you have any other financial competing interests?
- Do you have any non-financial competing interests in relation to this paper?

If you can answer no to all of the above, write 'I declare that I have no competing interests' below. If your reply is yes to any, please give details below.

I declare that I have no competing interests.

I agree to the open peer review policy of the journal. I understand that my name will be included on my report to the authors and, if the manuscript is accepted for publication, my named report including any attachments I upload will be posted on the website along with the authors' responses. I agree for my report to be made available under an Open Access Creative Commons CC-BY license (http://creativecommons.org/licenses/by/4.0/). I understand that any comments which I do not wish to be included in my named report can be included as confidential comments to the editors, which will not be published.

I agree to the open peer review policy of the journal

To further support our reviewers, we have joined with Publons, where you can gain additional credit to further highlight your hard work (see: https://publons.com/journal/530/gigascience). On publication of this paper, your review will be automatically added to Publons, you can then choose whether or not to claim your Publons credit. I understand this statement.

Yes Choose an item.
